# Genome Regions Associated with Functional Performance of Soybean Stem Fibers in Polypropylene Thermoplastic Composites

**DOI:** 10.1371/journal.pone.0130371

**Published:** 2015-07-13

**Authors:** Yarmilla Reinprecht, Muhammad Arif, Leonardo C. Simon, K. Peter Pauls

**Affiliations:** 1 University of Guelph, Department of Plant Agriculture, Guelph, ON, Canada; 2 University of Waterloo, Department of Chemical Engineering, Waterloo, ON, Canada; The Chinese University of Hong Kong, HONG KONG

## Abstract

Plant fibers can be used to produce composite materials for automobile parts, thus reducing plastic used in their manufacture, overall vehicle weight and fuel consumption when they replace mineral fillers and glass fibers. Soybean stem residues are, potentially, significant sources of inexpensive, renewable and biodegradable natural fibers, but are not curretly used for biocomposite production due to the functional properties of their fibers in composites being unknown. The current study was initiated to investigate the effects of plant genotype on the performance characteristics of soybean stem fibers when incorporated into a polypropylene (PP) matrix using a selective phenotyping approach. Fibers from 50 lines of a recombinant inbred line population (169 RILs) grown in different environments were incorporated into PP at 20% (wt/wt) by extrusion. Test samples were injection molded and characterized for their mechanical properties. The performance of stem fibers in the composites was significantly affected by genotype and environment. Fibers from different genotypes had significantly different chemical compositions, thus composites prepared with these fibers displayed different physical properties. This study demonstrates that thermoplastic composites with soybean stem-derived fibers have mechanical properties that are equivalent or better than wheat straw fiber composites currently being used for manufacturing interior automotive parts. The addition of soybean stem residues improved flexural, tensile and impact properties of the composites. Furthermore, by linkage and *in silico* mapping we identified genomic regions to which quantitative trait loci (QTL) for compositional and functional properties of soybean stem fibers in thermoplastic composites, as well as genes for cell wall synthesis, were co-localized. These results may lead to the development of high value uses for soybean stem residue.

## Introduction

Composite materials are produced from two or more components, which have different physical and chemical properties. In biocomposites, one or more phases have a biological origin [1]. Increasing demands by automotive parts manufacturers and OEMs (original equipment manufacturers) for light weight, low cost materials coupled with an enhanced societal interest in reducing environmental impacts of manufacturing and transportation are driving interest in the development of new composite materials containing plant fibers as reinforcing fillers [2]. Natural plant fibers have good specific strength and modulus, high sound absorption, low density, reduced tool wear, enhanced energy recovery, reduced dermal and respiratory irritation, good biodegradability and are economically viable [3]. Plant fibers can be used to manufacture automobile parts as replacements for glass fibers in composites with plant- or petroleum-based plastics.

Composite characteristics are dictated by their end uses. For example, thermoplastic composites are often processed by injection molding, which allows a good balance between design requirements and manufacturing costs. The selection of materials is based on processing (molding) costs and performance (application) requirements. The major challenge associated with using composites with natural fibers for injection molding is the limited thermal stability (up to 200°C) of the fibers, whereas, the major challenge associated with the performance stage is formulating to meet OEM specifications. This requires developing a composite formulation with the appropriate balance of specific modulus and impact strength properties.

The major sources of plant biomass/natural fibers are wood residues (sawdust, wood chips and wood waste), energy crops (hybrid poplar, switch grass and willow) and agricultural residues (wheat straw, soybean straw and maize stem). Composites made with agro-based fibers typically have lower performance characteristics than those made with wood fibers [4], which may be due to the relatively low cellulose and lignin contents of some agro-based fibers [5]. Generally, low cost agro-based flours have filler properties that enhance the tensile and flexural moduli of composite materials but have little effect on their strength [6].

Soybean stem biomass is an abundant agricultural byproduct of soybean seed production worldwide, with no current significant industrial use. Its incorporation into composites would lead to new, value-added, non-food uses for this agricultural residue [2]. The world production of soybean in 2011 was 251.5 million metric tons (www.soystats.com/2012/page_30.htm), which resulted in potentially over 100 million tons of soybean stems available for biofiber production. In 2013, approximately one million hectares of Ontario land was devoted to soybean production (Statistics Canada: Field Crop Reporting Series; available at: http://www.omafra.gov.on.ca/english/stats/crops/index.html). Over 200 soybean cultivars can be grown in Ontario in five different heat areas (Ontario Oil and Protein Seed Crop Committee; http://www.gosoy.ca/soyhome.php). The genetic and environmental influences on the functional properties of soybean stem fibers in composites has not been determined, but could be highly variable [7].

The plant cell wall is the main source of lignocellulosic fibers in plant biomass. Dicots have type I primary cell walls [8], which consists of cellulose microfibrils buried in the matrix of hemicellulose (xyloglucan), pectin (homogalacturonan and rhamnogalacturonan) and proteins (extensins and atabinogalactans). The interactions between the different polysaccharides ensure that cell walls are strong, flexible and dynamic [9]. Secondary cell walls (synthesized after cell stops growing) are composed mostly of cellulose, hemicellulose, pectin and lignin [10, 11]. Cellulose is a long, linear polymer of (1–4)-β-linked glucose. Its degree of polymerization varies widely among plant species and it usually contains 3,000–5,000 glucose units per chain in secondary cell walls of woody fibers but up to 15,000 units in cotton fibers [12], organized into crystalline and amorphous regions. In addition, cellulose can be converted from its native form (predominantly form I) to three other crystalline forms by chemical treatments [13, 14]. Hemicellulose has shorter chains but has a more complex structure compared to cellulose. It is a species-specific, highly branched polymer of five- (xylose and arabinose) and six-carbon sugars (galactose, glucose and mannose). Lignin is a complex aromatic polymer composed of monolignol units (*p*-coumaryl alcohol, coniferyl alcohol and sinapyl alcohol) with different levels of methoxylation. The ratios of the monolignols in lignin vary among plant species, tissues and cell wall layers [15]. Cellulose and lignin contribute to the mechanical properties of plant stems. Hemicellulose usually acts as filler between cellulose and lignin and mechanically contributes minimally to the strength and stiffness of fibers [16]. The lignin content plays a large role in the fiber structure, morphology and flexibility. In general, higher lignin contents are associated with finer and more flexible fibers [17].

The composition of plant cell walls is genetically controlled and varies among species, cultivars, tissues, developmental stages and environments [18, 19]. It is also highly dependent on the technique(s) used to make the measurements and variation exists between different evaluations. Cell wall components are often determined with a well established detergent analysis procedure used to measure the components and digestibility of fibers in forages and animal feeds [20]. It is performed in sequential manner, by employing a series of extractions, and measures the content of neutral detergent fiber (NDF, measures cellulose, hemicellulose and lignin), acid detergent fiber (ADF, measures cellulose and lignin) and acid detergent lignin (ADL, lignin). It also provides estimates of hemicellulose (NDF—ADF) and cellulose (ADF—ADL) contents.

Identification of quantitative trait loci (QTL) associated with phenotypic variation for cell wall components and genes underlying these QTL would result in a better understanding of genetic bases of these traits, while the development of molecular markers for the QTL would simplify and accelerate breeding for these traits. A number of fiber QTL studies have been conducted and numerous QTL have been mapped for cell wall components in various plant species. For example, QTL that explained significant variation for NDF (cellulose, hemicellulose and lignin), ADF (cellulose and lignin) and ADL (lignin) contents in maize stems have been identified on all ten chromosomes in different mapping populations {recombinant inbred lines (RIL) B73 x B52, n = 200; [21]; F_3_ B73 x De811, n = 150; [22]; RIL (F_6_) B73 x De811, n = 200; [23]}. In Arabidopsis, QTL for stem fiber length and lignin content were identified on chromosomes 2 and 5 in Col-4 x Ler-0 RIL population (n = 98) and annotated genes within the QTL intervals were investigated [24]. Numerous QTL for seed cell wall polysaccharides have been identified in soybean Minsoy x Archer RIL population {n = 108; [25]} however, no QTL information is available for soybean stem cell wall components. All these QTL for cell wall components were identified in populations of different types and sizes, usually designed to segregate specifically for the trait(s) of interest. However, in a mapping population not all segregants are equally informative and selective mapping was proposed as an alternative approach, that is valid especially for the traits that are expensive or difficult to phenotype [26, 27]. In selective mapping, the focus can be on lines at the extreme (high and low) ends of the trait distribution, which have a tendency to contain more positive and negative alleles. By selecting the most informative recombinants from the mapping (random) population, selective mapping can be as effective as whole population mapping. In general, the detection of major QTL is not affected by selective mapping. However, selective mapping may have reduced power to detect minor QTL (<10%), since the selected population contains only a portion of variance explained by the whole population and may result in a reduced linkage map [28, 29].

The clustering of QTL associated with fiber traits was reported for a number of plant species, including cotton [30, 31] and maize [21, 22] and has been attributed to linkage or pleiotropy. It was proposed that the underlying genetic basis for this observation is clustering of developmentally-related genes in plant genomes {reviewed in Nȕtzman and Osbourn [32]}. In particular, clusters of cotton fiber quality QTL may represent groups of coordinately regulated genes and/or groups of small gene families that have undergone proximal duplication followed by sub- or neo-functionalization [33]. However, the biosynthetic pathways for cellulose, hemicellulose and lignin are different and complex and involve the synthesis of numerous diverse products [34, 35]. It was estimated that over 2,000 genes are involved in cell wall biosynthesis and modification in Arabidopsis stems [36].

Cellulose is synthesized by plasma membrane-associated cellulose synthase complexes. Cellulose synthase catalytic subunits are encoded by the *CesA* gene superfamily [37], with ten members in rice and Arabidopsis and 20 members in maize [38]. In Arabidopsis, different sets of genes are required for the synthesis of a functional primary (*CesA1*, *CesA3* and *CesA6* or *CesA6-like*) and secondary cell walls {*CesA4*, *CesA7* and *CesA8* complexes [39]}. In maize, three *CesA* genes (*ZmCesA10–12*) clustered with homologs of Arabidopsis *CesA4*, *CesA7* and *CesA8*, have roles in the formation of cellulose in secondary cell walls [40]. Currently, 17 genes have been annotated as cellulose synthase (EC:2.4.1.12) in the soybean genome {*Glycine max* Wm82.a2.v1, Phytozome v9.1, accessed 13 Mar 2015; [41]}.

Cellulose and hemicellulose are closely associated in cell walls by enzymatic modifications and chemical crosslinking [42]. Hemicellulose synthesis occurs in the Golgi bodies [43]. Enzymes for hemicellulose biosynthesis are encoded by a cellulose synthase-like (*Csl*) gene superfamily classified into eight families (*CslA* to *CslH*). In Arabidopsis, 29 *Csl* genes are classified into six families (no *CslF* and *CslH* families); in rice, 36 *Csl* genes have been identified in six families (no *CslB* and *CslG* families); and 33 *Csl* genes identified in maize belong to five families {no *CslB*, *CslG* and *CslH* [38]}. The current version of the soybean genome contains over 60 sequences annotated as *Csl* genes {*G*. *max* Wm82.a2.v1, Phytozome v9.1, accessed 13 Mar 2015; [41]}.

The two-stage biosynthesis of lignin through the phenylpropanoid pathway is relatively well understood. The first stage is the synthesis of monolignols, which starts from the amino acid phenylalanine and proceeds through number of ring and side-chain modifications catalyzed by dozens of enzymes encoded by small gene families [including: phenylalanine ammonia lyase (*PAL*), cinnamate 4-hydroxylase (*C4H*); 4-coumarate:coenzyme A ligase (*4CL*), hydroxycinnamoyl transferase (*HCT*), coumarate 3’-hydroxylase (*C3’H*), caffeic acid O-methyltransferase (*COMT*), cinnamoyl-CoA reductase (*CCR*), cinnamyl alcohol dehydrogenase (*CAD*), caffeoyl-CoA O-methyltransferase (*CCoAOMT*), ferulate 5-hydroxylase (*F5H*)] [44]. In total, 81 genes in Arabidopsis, 86 genes in rice and 102 genes in maize are involved in phenylpropanoid biosynthesis [38]. The monolignols are polymerized into lignin as *p*-hydroxyphenyl (H lignin), guaiacyl (G lignin) and syringyl (S lignin) phenylpropanoids. These steps are catalyzed by numerous peroxidases, laccases [45] and dirigent proteins. The soybean genome is characterized by high redundancy and gene duplications that resulted from at least two rounds of polyploidization [46]. Most of the low copy sequences are present in more than two copies. For example, soybean has eight *PAL* genes compared to four *PAL* genes in Arabidopsis; laccase (EC:1.10.3.2) is encoded by 17 genes (*LAC1* to *LAC17*) in Arabidopsis and 54 *LAC* genes in soybean {*Glycine max* Wm82.a2.v1, Phytozome v9.1, accessed 13 Mar 2015; [41]}.

Biosynthesis of secondary cell walls is complex and requires coordinated expression of secondary wall structural genes and targeted secretion, deposition and assembly of wall components {cellulose, hemicellulose and lignin; [47–49]}. A transcription factor network, composed of secondary cell wall NAC domain and R2R3 MYBs, regulates secondary cell wall biosynthesis in Arabidopsis. Several NAC domain transcription factors (SND1, NST1, NST2, VDN6 and VND7) act as master switches and can directly activate the expression of secondary cell wall specific biosynthetic genes and downstream transcription factors (such as: SND2, SND3, KNAT, AtMYB46, AtMYB52, AtMYB54, AtMYB58, AtMYB63, AtMYB85 and AtMYB103) that also directly regulate secondary cell wall biosynthetic genes.

Biomass from crop production is a large source of natural fibers. In North America several thousand soybean varieties are grown on over 32 million hectares (http://www.agcensus.usda.gov/Publications/2012/Full_Report/Volume_1,_Chapter_1_US/usv1.pdf), spanning 13 maturity zones [50]. This production leaves more than 15 million tons of residue in the fields annually (http://www.ofa.on.ca/uploads/userfiles/files/biomass_crop_residues_availability_for_bioprocessing_final_oct_2_2012.pdf). Approximately one third of the residues could be safely removed from the fields and used in various industrial applications. However, the relative contributions of the genetic backgrounds and environments the plants are grown in to the compositional and functional properties of soybean stem fibers are unknown. The current study characterized the chemical compositions of mature soybean stems grown in different environments, measured the genetic and environmental effects on the performance properties of stem fibers after incorporation into a low-cost polypropylene (PP) thermoplastic matrix and identified chromosomal locations conditioning these unique stem fiber traits in soybean. The work identified novel QTL for this traditional food and feed crop that could lead to the development of high value uses for the stem residue.

## Materials and Methods

### Plant Material

A selective phenotyping approach, with a set of 50 RILs, representing approximately 30% from an existing, well characterized RG10 x OX948 mapping population of 169 RILs [51, 52] was used in this study. A height per unit of lodging (H/L) was derived from the plant height and lodging measurements made previously on these lines [51] and used to select two groups of lines with contrasting stem characteristics from both ends of the trait distribution (Fig A in S1 File). Two parents (RG10 and OX948), 50 RILs and four check cultivars (OAC Kent, OAC Huron, OAC Champion and OAC Prodigy) were grown in three Ontario locations {Harrow [Woodslee (3410 CHU, crop heat units)], Ridgetown (3340 CHU) and Woodstock (2890 CHU), ON Canada} in two years [2008 (F_8_ generation) and 2009 (F_9_ generation)] using a rectangular (7 x 8) lattice design with two replications. The 2008 trials were machine planted on 29 May in Harrow (Woodslee), on 30 May in Ridgetown and on 2 June in Woodstock; the 2009 trials were planted on 21 May in Ridgetown, on 26 May in Harrow (Woodslee) and on 16 June in Woodstock. The Harrow (Woodslee, Brokstoon Clay) trials were planted in 5-row plots with 0.45 m row-to-row spacing and 2.5 m long in 2008 and 4 m long in 2009. The Ridgetown (Brookston Clay) trials were planted as 5-row plots, 4 m long and 0.43 cm row-to-row spacing; three center rows were harvested. The Woodstock (Guelph Loam) trials were planted as 4-rows plots, 4 m long and 1.65 m wide. Standardized cultural practices were performed as needed. Daily weather data for the three locations and two experimental years (Fig B in S1 File) were collected at the nearest weather stations (available at: http://climate.weather.gc.ca/climateData/dailydata_e.html). Days to maturity were expressed as the number of days from planting to harvest maturity when 95% of plants had mature color. At physiological maturity, ten randomly selected plants were characterized for the plant height and lodging. Plant height was measured as the height (cm) of plant from the soil surface to the tip of the primary stem. Lodging was determined as standability of plants in plot at maturity using a scale of 1 to 5 (1, erect plants; 5, prostrate plants). Height per unit of lodging was derived from the height and lodging measurements. The plots were harvested [Harrow (Woodslee)- 7 October 2008 and 14 October 2009; Ridgetown—21 October 2008 and 8 October 2009; Woodstock—7 November 2008 and 10 November 2009] with a plot combine and threshed separately. Stems were collected from each plot, dried and ground with a Thomas Wiley Mill Model 4 (Thomas Scientific, Swedesboro, NJ) to pass through a 2 mm sieve and stored at -10°C until use.

### Chemical Analysis of Fibers

A three-step detergent fiber analysis [20] was used to characterize ground dry soybean stems (a single 0.5 g sample from each replication/location/year) for neutral detergent fiber [NDF, isolates cell wall (hemicellulose, cellulose and lignin)], acid detergent fiber (ADF, estimates cellulose and lignin) and acid detergent lignin (ADL, isolates lignin). Sequential analysis using a filter bag method was performed with the Ankom 200 fiber analyzer (Ankom technology, Macedon, NY) according to the manufacturer’s instructions (http://www.ankom.com/analytical-procedures.aspx). Hemicellulose and cellulose contents were calculated from NDF, ADF and ADL values (hemicellulose = NDF—ADF, cellulose = ADF—ADL) and expressed in % on a dry weight basis.

The content of free phenolics in ground soybean stems was determined with a 50% Folin-Ciocalteu’s phenol reagent (Sigma Chemicals Company, St. Louis, USA) using a microwave-based protocol [53] and determined at 725 nm with the SpectraMax Plus384 absorbance microplate reader using SOFTmax PRO 4.0 controller software (Molecular Devices Corporation, Sunnyvale, CA, USA). Gallic acid (Acros Chemical Company, NJ, USA) in 50% ethanol was used as a standard and the quantities of free phenolics were expressed as an average of three (10 mg) subsamples measurements (from each replication/location/year) in μg mg^-1^ phenolics on a dry weight basis.

### Thermogravimetric Analysis

To optimize the processing conditions, the thermal stability of the fibers was determined by thermal gravimetric analysis (TGA), prior to compounding in the PP matrix. The fiber onset degradation temperature (°C) was measured by heating samples (one per pooled replication from each location/year) from 35°C to 700°C at a heating rate of 10°C min^-1^ in a nitrogen environment (flow rate of 50 ml min^-1^) with the TGA Q500 instrument (TA Instruments, New Castle, DE, USA). The weight loss as a function of temperature was measured with the Universal Analysis 2000 instrument (TA Instruments, New Castle, DE, USA). TGA thermographs were used to measure weight losses for fibers from the different RILs. Onset degradation temperatures were recorded as the temperatures (°C) at which the samples showed 1% weight loss.

### Soybean Stem Fiber/Polypropylene (SS/PP) Composites

Composite formulations consisted of: homopolypropylene matrix [Pro-fax 6301(A. Schuman Inc., OH, USA) with 12 melt flow index (MI)], individual soybean stem fiber samples from each of the 50 RILs and two parental genotypes grown in four environments (Harrow and Woodstock in 2008 and 2009, pooled replications), a coupling agent [Fusabond P-353, maleic anhydride grafted polypropylene (DuPont, Mississauga, ON, Canada)] and antioxidants [Irganox 1010, Phenolic and Irgafos 168, Phosphate (Ciba, Inc., Mississauga, ON, Canada)]. The materials were homogenized with a melt blend process. Soybean stem residue (20 wt-%), polypropylene (77.5 wt-%), coupling agent (2 wt-%) and antioxidants (0.25 wt-% each Irganox 1010 and Irgaphos 168) were hand mixed to get a uniform mixture and extruded using a conical twin-screw micro-extruder (Haake MiniLab, Thermo Electron Corporation, Waltham, MA, USA) with optimized processing conditions (190°C, 40 rpm). The extruded composites (208 formulations) were hand cut into pellets and molded to produce 15 test bars using an injection molding RR/TSMP machine (Ray-Ran, Warwickshire, UK) with the barrel temperature at 190°C, mold tool temperature at 50°C, 15 sec hold time at 100 psi pressure. The test specimens were annealed in an air circulating oven GC 5890A (Hewlett Packard, Ramsey, MN, USA) at 150°C for 10 min at a temperature rate of 10°C min^-1^ and cooled down to room temperature. Ten test bars (63.5±0.2 12.5±0.2 x 3.10±0.2 mm) produced with stem fibers of 50 RILs and two parental genotypes were used for each mechanical test, including: five test bars for flexural strength and flexural modulus, and five test bars for impact strength (Izod test), according to ASTM (the American Society for Testing and Materials; ASTM International) standards. The tests for flexural strength and flexural modulus (five test bars) were performed according to the ASTM Standard D790-10 [54] using the TestResources machine Model 120Q1000 (TestResources, Inc., Shakopee, MN, USA). The Izod test (five test bars) was performed according to ASTM Standard D256-10 [55] for measuring impact strength using the TMI impact testing machine (TMI Testing Machine, Inc., New Castle, DE, USA). Five test specimen bars (37.4.0±0.2 x 5.0±0.2 x 2±2.0 mm) as described by ASTM Standard D1708-10 [56] were used for tensile strength, tensile modulus and ultimate tensile strength measurements with the same machine as used for testing flexural properties.

### Trait Data Analysis

Analysis of variance was performed using the Proc Mixed procedure in SAS (Statistical Analysis System) v.9.2 software [57]. Data were analyzed separately for each location and year, combined across locations for each year, and combined over two years. Genotypes and locations were considered as fixed effects and all other effects were considered to be random. The homogeneity of error variances were tested before pooling data for combined analyses using a residual analysis in SAS. The relationships among agronomical traits, fiber chemical traits and the physical traits of the composites were analyzed by correlation (Spearman) in SAS and principal components analysis (PCA) using STATISTICA v.9 software [58]. Heritability in standard units [59] for fiber traits and composite mechanical traits was estimated by determining correlations between the values for 2009 RILs (F_9_) and 2008 RILs (F_8_).

### Mapping and Marker Development

Over 100 genes involved in cell wall biosynthesis and modifications were selected from databases (NCBI, DFCI) and microarray literature and more than 200 gene-specific PCR primers were designed (Table A in S1 File). Genomic DNA was isolated from the young leaves (100 mg) of growth room-grown plants with the DNeasy plant mini kit (Qiagen Inc.—Canada, Mississauga, ON, Canada) according to manufacturer's protocols. Cell wall gene-specific primers were screened with parental (RG10 and OX948) genomic DNA. PCRs were performed in 20 μl volumes containing 1x PCR buffer (supplied with enzyme), 3 mM MgCl_2_ (supplied with enzyme), 0.1 mM each of dNTPs (Invitrogen, Life Technologies, Inc., Burlington, ON, Canada), 1.6 U *Taq* DNA polymerase (Invitrogen), 5 μM each of the forward and reverse primer and 24 ng of soybean genomic DNA, with a PTC- 100 Programmable Thermal Controller (MJ Research, Inc., Watertown, MA, USA). The amplification program consisted of an initial 2 min denaturation step at 94°C, followed by 35 cycles of denaturation at 94°C for 30 s, annealing at 55–60°C for 45 s and extension at 72°C for 1 min, with a final extension at 72°C for 10 min. The PCR products were separated by electrophoresis on 1% w/v agarose gel containing ethidium bromide in a 1xTBE buffer at 100 V for 2 h and visualized under ultraviolet light. Polymorphic primers were used to screen 169 RILs from the RG10 x OX948 population. Monomorphic PCR products were purified and used as a template for cycle sequencing (CEQTM 8000 genetic analysis system; Beckman Coulter Inc., Fullerton, CA, USA). Sequences were compared to existing soybean sequences by BLAST searches at NCBI (http://www.ncbi.nlm.nih.gov/BLAST/) to ensure that the target genes had been cloned. Newly produced single nucleotide polymorphism (SNP) markers were used to screen the complete RG10 x OX948 population. Fiber genes were isolated and gene-specific PCR-based markers for several key enzymes in cellulose, hemicellulose and lignin biosynthetic pathways were developed.

Mapmaker/Exp 3.0b [60] was used to add the newly developed fiber gene-based markers to the previously created RG10 x OX948 linkage map, which contained 120 markers [simple sequence repeat (SSR), random amplified polymorphic DNA (RAPD) and gene (omega-3 fatty acid desaturase and seed lipoxygenase)-based sequence-tagged sites (STS) and cleaved amplified polymorphic sequences (CAPS)] on 26 linkage groups (18 chromosomes) and covered 1,247.5 cM [51]. A minimum LOD score of 3.0 and maximum distance between two markers of 50.0 cM were used to assign new fiber gene-based loci into linkage groups. Recombination frequencies were converted to cM distances using Kosambi’s mapping function [61]. QTL were identified with composite interval mapping (CIM) using Windows QTL Cartographer version 2.5 [62] with the following settings: map function Kosambi, a walk speed of 2 cM, five control markers, model 6 (standard), forward and backward regression (method 3) and probabilities of 0.05. Genome-wide scans were performed for each trait and QTL. The 1,000 permutation test at 0.05 significance level for CIM was used to determine LOD thresholds for each trait (QTL group 1). Because of the novelty of some of the mapping traits, QTL at LOD threshold values ≥2.5 (program's default; QTL group 2) and ≥2.0 {LOD threshold used in Reinprecht et al [51]; QTL group 3} were also considered as putative QTL. The map positions of these QTL were detected using the option for automatic QTL location (using program's default parameters). Several QTL not automatically detected were also marked as putative when they exceeded threshold LOD scores (QTL group 4). Additive effects at each significant QTL and the percentages of phenotypic variation (R^2^) explained by QTL for each trait were acquired directly from the CIM output.

A soybean *in silico* map was generated by a two-step process. Initially, all gene sequences used to design fiber gene-based primers were BLASTed against soybean Williams 82 (Wm82) genome (*G*. *max* Wm82.a2.v1) in Phytozome v9.1 {available at: www.phytozome.net; [41]}. Flanking markers of the newly identified fiber compositional and composite performance QTL intervals were used to position these QTL on soybean physical map. Subsequently, genomic regions containing these QTL were scanned for additional candidate genes that might be involved in cell wall biosynthesis and modification. The maps were drawn with the MapChart 2.2 software[63]. For each chromosome, the genetic and *in silico* maps were aligned and connected by common SSR markers.

## Results and Discussion

### Quantitative Traits Variability

In this work we used 50 RILs, approximately 30% of the existing soybean RG10 x OX948 mapping population (n = 169) created from the parents with different agronomic and seed characteristics with available extensive molecular genetic information [51, 52]. The selection of RILs was based on the height per unit of lodging (H/L), a trait derived from the plant height and lodging measurements [51]. Cell wall composition was associated with stem strength and standability (lodging) in wheat [64, 65]. In pea, lodging was negatively correlated with lignin and cellulose contents in stems [66].

Significant genotype by environment (GxE) interactions were detected for the most of the analyzed traits (Table B in S1 File). Therefore, QTL analysis was performed separately for each environment. Frequency distributions for all traits (raw data) are shown in Fig C-a to C-c in S1 File.

#### Agronomic traits

RILs were different for all agronomic traits (days to maturity, plant height, lodging and derived height per unit of lodging trait) evaluated in three locations over two years (Fig D-a in S1 File). Maturity varied from 109 days (Harrow, 2008) to 146 days (Ridgetown. 2009). Variability for plant height was higher in the second year (2009) and ranged from 58.5 cm (Ridgetown) to 115 cm (Harrow) and was likely associated with greater moisture availability and higher temperatures during the second growing season compared to the first (Fig B in S1 File). However, variability for plant height was less in this data set when compared to the values for this trait evaluated for the whole population in a different set of environments [51].

#### Chemical composition of soybean stem fibers

The fibers obtained from mature stems of the parental genotypes (RG10 and OX948) and 50 RILs grown in three locations over two seasons, after cutter milling and sieving through a 2.0 mm pore-size screen showed difference in color, and varied from light (Fig E-a in S1 File) to a deep brown (Fig E-b in S1 File). The stem fibers also had significantly different chemical compositions. Transgressive segregants were detected for all fiber compositional traits among RILs grown in six environments (Table 1; Fig D-b in S1 File). Stem fibers of RILs contained 27.0% (Woodstock, 2008) to 41.0% (Harrow, 2009) cellulose, 13.1% (Harrow, 2009) to 20.1% (Ridgetown, 2009) hemicellulose, 8.7% (Woodstock, 2008) to 19.2% (Woodstock, 2009) lignin and 1.31 μg mg^-1^ (Ridgetown, 2009) to 3.33 μg mg^-1^ (Ridgetown, 2008) free phenolics (Table 1; Fig D-b in S1 File). Johnson et al [67] reported slightly higher values of these cell wall components [cellulose 526 g kg^-1^, hemicellulose 289 g kg^-1^, and lignin 168 g kg^-1^ (acid-insoluble) + 5 g kg^-1^ plant material (acid-soluble)] for soybean line NK S14-M7. Reddy and Yang [68] reported wide ranges of cellulose (44–83%, ADF method) and lignin (5–14%, Klason lignin) contents in soybean straw. The low performance of plant fibers in composites is associated, in part, with the degradation of fiber components caused by the high temperatures necessary to melt resins (~ 200°C)[69]. The stem fibers of the RILs used in this study showed significant variation (17.3°C) in their onset degradation temperatures. They ranged from 188.2°C (Woodstock, 2009) to 205.5°C (Woodstock, 2008), but were generally lower than wheat fibers (208.8°C) and pure PP (350.0°C)(data not shown).

**Table 1:** Chemical composition of soybean stem fibers from parental genotypes and 50 selected RG10 x OX948 recombinant inbred lines (RILs).

Trait	Year	Location	Genotypes					
			Parents		RILs			
			RG10	OX948	Mean ± SD	Range	CV (%)	H^2^
Cellulose (%)	2008	Harrow	39.3	37.9	37.5 ± 1.59	32.6–40.0	4.25	32.6
		Ridgetown	37.6	38.4	36.7 ± 1.62	32.7–40.0	4.40	
		Woodstock	40.4	34.0	35.8 ± 2.76	27.0–40.8	7.70	
	2009	Harrow	39.6	38.2	37.9 ± 1.95	33.0–41.0	5.15	
		Ridgetown	35.2	35.0	34.7 ± 2.40	28.6–40.1	6.93	
		Woodstock	34.5	36.1	36.3 ± 2.08	29.8–40.4	5.73	
Hemicellulose (%)	2008	Harrow	18.2	17.2	17.6 ± 0.43	16.7–18.6	2.42	1.2
		Ridgetown	17.2	18.9	17.4 ± 0.57	16.0–18.6	3.25	
		Woodstock	16.5	16.9	17.1 ± 0.65	15.1–18.9	3.79	
	2009	Harrow	15.7	16.8	16.7 ± 0.98	13.1–19.1	5.83	
		Ridgetown	16.2	14.2	16.3 ± 1.13	13.5–20.1	6.90	
		Woodstock	16.0	16.7	16.6 ± 0.95	14.7–19.0	5.76	
Lignin (%)	2008	Harrow	12.2	11.0	11.7 ± 0.98	9.7–14.1	8.31	13.1
		Ridgetown	14.2	12.2	12.9 ± 1.23	10.5–16.4	9.53	
		Woodstock	13.0	13.1	11.5 ± 1.35	8.7–16.1	11.67	
	2009	Harrow	12.6	13.5	13.3 ± 0.21	10.1–18.4	11.31	
		Ridgetown	14.0	13.0	12.7 ± 0.26	9.0–18.1	14.19	
		Woodstock	11.7	11.9	12.6 ± 1.93	9.9–19.2	15.36	
Free phenolics (μg mg^-1^)	2008	Harrow	2.27	2.28	2.23 ± 2.243	1.83–2.90	10.89	37.1
		Ridgetown	2.22	2.38	2.34 ± 0.300	1.66–3.33	12.85	
		Woodstock	2.43	2.73	2.19 ± 0.257	1.63–2.93	11.74	
	2009	Harrow	1.40	2.05	1.99 ± 0.382	1.32–3.22	19.22	
		Ridgetown	1.86	2.39	2.09 ± 0.335	1.31–2.75	16.03	
		Woodstock	2.16	2.02	2.08 ± 0.320	1.51–3.11	15.42	

#### Mechanical properties of soybean stem fiber/polypropylene (SS/PP) composites

In the current study, a total of 208 soybean straw formulations were molded into test specimens with PP and analyzed for their mechanical properties. The results showed that both genotype and environment had significant effects on the performance (mechanical) properties of the composites (Table B in S1 File). Fibers from different RILs, when incorporated into PP matrix, were significantly different for their flexural, tensile and impact properties. Furthermore, transgressive segregants were identified for all the traits, which indicates that these traits could be improved through breeding (Table 2; Fig D-c in S1 File). To the best of our knowledge, this is the first demonstration that the genetic background of the source of plant fibers influences the performance characteristics of the composite materials manufactured from them.

**Table 2:** Mechanical properties of soybean stem fiber/polypropylene (SS/PP)^a^ composites.

Trait	Year	Location	Genotypes					
			Parents		RILs			
			RG10	OX948	Mean ± SD	Range	CV (%)	H^2^
Flexural strength (MPa)	2008	Harrow	46.7	49.7	49.3 ± 1.68	46.7–51.6	3.42	14.4
		Woodstock	49.6	46.6	49.2 ± 1.99	45.8–52.5	4.05	
	2009	Harrow	48.1	49.5	49.3 ± 1.87	45.8–53.0	3.80	
		Woodstock	46.2	48.4	48.6 ± 2.25	42.5–51.6	4.64	
Flexural modulus (MPa)	2008	Harrow	1293	1248	1347 ± 54.9	1215–1466	5.86	21.2
		Woodstock	1324	1218	1349 ± 64.1	1218–1490	6.30	
	2009	Harrow	1404	1323	1361 ± 57.0	1236–1563	6.99	
		Woodstock	1243	1371	1327 ± 70.6	1109–1428	7.29	
Ultimate tensile strength (MPa)	2008	Harrow	34.4	35.9	35.7 ± 1.84	31.4–38.4	5.15	9.9
		Woodstock	37.2	36.1	35.5 ± 1.13	31.1–37.9	6.00	
	2009	Harrow	34.0	34.4	34.7 ± 2.58	30.9–38.8	7.44	
		Woodstock	34.6	35.3	34.4 ± 2.20	30.7–37.9	6.39	
Tensile strength (MPa)	2008	Harrow	31.9	33.7	32.8 ± 1.83	29.8–35.9	5.58	9.8
		Woodstock	34.8	33.7	32.5 ± 2.05	29.4–34.9	6.29	
	2009	Harrow	32.8	32.1	32.3 ± 2.35	28.5–35.9	7.26	
		Woodstock	31.4	32.4	32.2 ± 2.02	28.9–35.4	6.29	
Tensile modulus (MPa)	2008	Harrow	223	254	259 ± 29.2	177–318	16.16	25.0
		Woodstock	243	247	261 ± 31.4	173–315	16.77	
	2009	Harrow	239	256	256 ± 23.5	202–321	14.94	
		Woodstock	238	251	250 ± 25.1	207–330	14.83	
Impact strength (J m^-1^)	2008	Harrow	25.6	23.9	26.5 ± 4.68	22.9–33.9	17.64	0.6
		Woodstock	23.0	26.4	26.8 ± 4.81	23.0–33.2	17.93	
	2009	Harrow	30.8	26.4	26.4 ± 4.43	21.7–33.6	16.49	
		Woodstock	24.8	26.0	26.0 ± 4.78	17.0–33.6	18.38	

^a^SS/PP composites were developed from stem fibers (20.0 wt %) of 50 soybean RILs and two parental genotypes (RG10 and OX948), which were grown in two Ontario locations over two years

In general, the addition of soybean stem fibers to the PP matrix resulted in composite materials that had improved tensile and flexural properties relative to pure PP. The values for flexural strength, flexural modulus and tensile modulus were 17%, 33% and 15% higher, compared to pure PP (Table C in S1 File; Fig D-c in S1 File). In addition, all SS/PP composites had higher ultimate tensile strength and impact strength values compared to the WS/PP composites. Average tensile strength values were similar to the WS/PP composite and the flexural strength, flexural modulus and tensile modulus values of the SS/PP composites were lower than the values for WS/PP composite (Table C in S1 File; Fig D-c in S1 File). However, there were lines from which fibers were obtained that produced composites with similar or higher values of flexural strength (53.0 MPa), flexural modulus (1563 MPa) and tensile modulus (330 MPa) than the WS/PP composite. Similarly, composites produced with fibers of some RILs had higher values of ultimate tensile strength (38.8 MPa), tensile strength (35.9 MPa) and impact strength (33.9 J m^-1^) compared to pure PP (Table C in S1 File; Fig D-c in S1 File). These results indicate that soybean stem residues, when incorporated into a PP matrix, behave as enforcing materials. These data support previously reported results with composites with soybean stem flour [70, 71]. The addition of plant fibers to polymer matrix increases tensile and flexural properties of composites. The onset degradation temperature of soybean stem fibers incorporated into composites was 225°C, which was considerably lower compared to pure PP (350°C). However, pure PP degrades completely at 425°C while soybean fiber composites degrade at 462°C in nitrogen gas environment suggesting that the incorporation of soybean fibers retards the process of degradation at higher temperatures (data not shown). The superior characteristics of the soybean/PP matrices, compared to the wheat fiber/PP matrix, is significant because a wheat straw fiber-filled PP (WS/PP) composite is currently being used in automotive parts production (http://corporate.ford.com/news-center/press-releases-detail/pr-ford-teams-up-to-develop-wheat-31391).

### Associations Between Fiber Composition and Composite Performance

Significant negative correlations were found between height per unit of lodging (derived selection trait) and plant height and lodging measurements. This trait was also correlated with some fiber and composite traits in some environments (Table 3). The relationships among fiber compositional traits were complex, as indicated by inconsistent low to moderate correlations in samples from different locations/years (S1 Table). For example, a significant positive correlation was detected between cellulose and lignin contents of the soybean stem fibers [R = 0.39 (p = 0.0058) Woodstock, 2008], cellulose and hemicellulose contents in samples from some environments [R = 0.39 (p = 0.0051) Woodstock, 2008; R = 0.28 (p = 0.0498) Ridgetown, 2009] or hemicellulose and lignin contents [R = 0.37 (p = 0.0091) Woodstock, 2008]. Cellulose content of the stem fibers grown in some environments was significantly and positively correlated with some physical traits of the composites they were incorporated into, including: flexural strength [R = 0.30 (p = 0.0323)] and flexural modulus [R = 0.29 (p = 0.0443)] in samples from Woodstock (2008) or tensile modulus [R = 0.29 (p = 0.0398)] in samples from Harrow (2009). On the other hand, content of free phenolics was negatively correlated with impact strength [R = -0.36 (p = 0.0095)] in samples from Woodstock (2008) (S1 Table).

**Table 3:** Significant correlations between height per unit of lodging (H/L) and agronomic, fiber compositional and composite mechanical traits in 50 selected RG10 x OX948 recombinant inbred lines (RILs) in different environments.

Trait	Environment	Height per unit of lodging (H/L)					
		Harrow 2008	Ridgetown 2008	Woodstock 2008	Harrow 2009	Ridgetown 2009	Woodstock 2009
Days to maturity	Harrow 2008	-0.47***	-0.34*	NS^a^	NS	-0.31*	NS
	Ridgetown 2008	-0.38**	-0.46***	-0.40**	NS	-0.29*	-0.29*
	Woodstock 2008	-0.28*	-0.32*	-0.34*	NS	NS	NS
	Harrow 2009	NS	-0.40**	-0.28*	-0.39*	-0.35*	-0.32*
	Ridgetown 2009	-0.42**	-0.51***	-0.40**	-0.28*	-0.30*	-0.35*
	Woodstock 2009	NS	NS	NS	NS	NS	NS
Plant height	Harrow 2008	-0.28*	-0.36*	-0.30*	NS	NS	-0.34*
	Ridgetown 2008	-0.33*	NS	-0.39**	NS	-0.34*	-0.33*
	Woodstock 2008	NS	-0.32*	-0.29*	-0.28*	NS	-0.29*
	Ridgetown 2009	NS	-0.28*	NS	NS	NS	NS
	Woodstock 2009	-0.38**	-0.43**	-0.34*	NS	-0.31*	-0.31*
Lodging	Harrow 2008	-0.92***	-0.59***	-0.72***	-0.47***	-0.42**	-0.52***
	Ridgetown 2008	-0.59***	-0.92***	-0.70***	-0.61***	-0.58***	-0.56***
	Woodstock 2008	-0.72***	-0.65***	-0.96***	-0.69***	-0.57***	-0.66***
	Harrow 2008	-0.41**	-0.55***	-0.65***	-0.96***	-0.66***	-0.58***
	Ridgetown 2008	-0.46***	-0.64***	-0.56***	-0.62***	-0.88***	-0.46***
	Woodstock 2008	-0.59***	-0.56***	-0.72***	-0.60***	-0.49***	-0.88***
Cellulose	Ridgetown 2009	NS	-0.29*	NS	NS	NS	NS
	Woodstock 2009	NS	0.37**	NS	NS	NS	0.35*
Lignin	Harrow 2008	NS	NS	NS	0.28*	NS	NS
Free phenolics	Ridgetown 2008	NS	NS	NS	-0.31*	NS	NS
Flexural modulus	Woodstock 2009	NS	NS	NS	0.29*	NS	0.28*
Ultimate tensile strength	Woodstock 2009	NS	NS	NS	-0.28*	NS	NS
Tensile strength	Woodstock 2009	NS	NS	NS	-0.31*	NS	NS
Tensile modulus	Harrow 2009	NS	NS	NS	NS	NS	-0.32*

^a^Not significant

*Significant at P = 0.05

**Significant at P = 0.01

***Significant at P = 0.001

**Fig 1:**
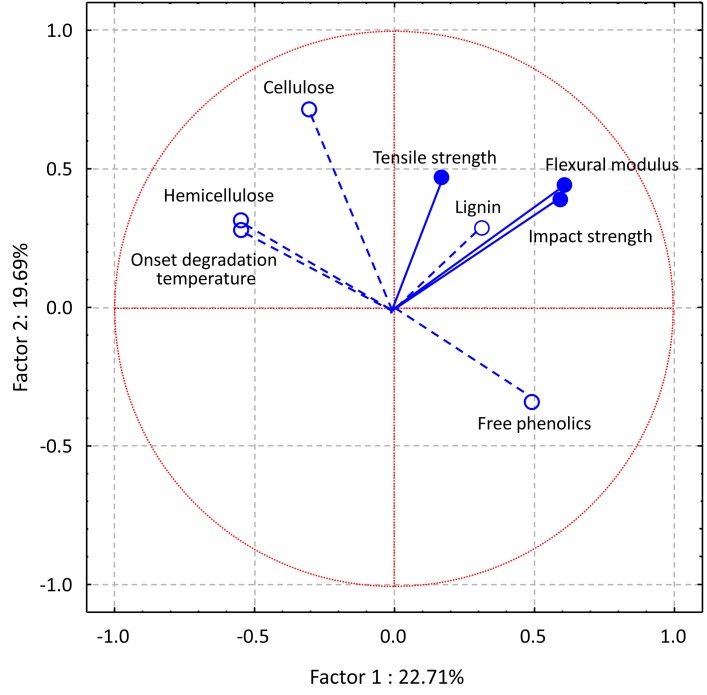
Projection of the physico-chemical variables of soybean stem fibers and soybean stem fiber/polypropylene (SS/PP) composite materials. Factors 1x2 explained 42.4% of the variability.

PCA was used to further examine potential relationships (average of four environments—Harrow and Woodstock in 2008 and 2009) among fiber and three main composite traits (tensile strength, flexural modulus and impact strength). Four factors explained 68% of the total fiber and composite traits variation among the RILs (data not shown). A biplot of the first two factors explained 42% of the variation in the RILs (Fig 1). With the exeption of lignin composition, which was grouped with composite mechanical properties (tensile strength, flexural modulus and impact strength), fiber composition and composite traits were separated into two groups. In particular, onset degradation temperature, hemicellulose and cellulose content grouped together with no significant effects on the composite traits. In addition, the free phenolic content of the fibers did not belong to any group and negatively affected hemicellulose content, cellulose content and onset degradation temperature (fiber traits). This is in agreement with the negative correlation values observed between free phenolics and these traits in some environments (S1 Table).

Some significant associations of the fiber compositional traits with mechanical properties of their composites indicates that the chemical compositions of soybean stem fibers affect their performance in SS/PP composites. This was expected because the major components of plant cell walls (cellulose, hemicellulose and lignin) were present in soybean stem fibers in highly various quantities among the different RILs. In addition, they have very different chemical structures [72, 73]. Cellulose is a simple homopolymer, composed of linear D-anhydroglucopyranose units joined together by β-1,4-glycosidic linkages, that is the same structure in all plants but present in different quantities. Hemicellulose is a more complex heteropolymer, consisting of pentoses, hexoses and sugar acids that differs among plants. It is branched and has molecular sizes that are 10 to 100 times smaller than cellulose and it is soluble in alkali and strong acids. Lignin is a complex hydrocarbon polymer with both aliphatic and aromatic components that differs, not only among plant species, but also, among cell types. It is soluble in alkali and readily oxidized with phenol [73]. Lignin functions as reinforcing agent in plant cell. It crosslinks various cell polysaccharides and provides mechanical strength to the cell wall. This variation in chemical structure would be expected to have different effects on composite mechanical properties. Cellulose has previously been associated with impovement of tensile strength and modulus in cellulose fiber-filled polypropylene composites [74]. Because of its complex chemical structure, hemicellulose has not been isolated from fibers and tested in composites. Toriz et al [75] reported lower tensile, flexural and unnotched impact strength in unmodified lignin-filled polypropylene composites compared to pure PP.

Heritability (in standard units) for fiber compositional and composite mechanical traits was low to moderate (Tables 1 and 2). Cellulose and free phenolics had moderate heritabilities (33% and 37%, respectively). Heritabilities for lignin and hemicellulose were low (13% and 1%, respectively). Lorenz et al [76] reported 0.79 and 0.24 broad-sense heritability for lignin content in two maize populations but to the best of our knowledge this has not been determined previously for soybean stem fibers and SS/PP composites.

### Selection of the Best Performing RILs

**Fig 2:**
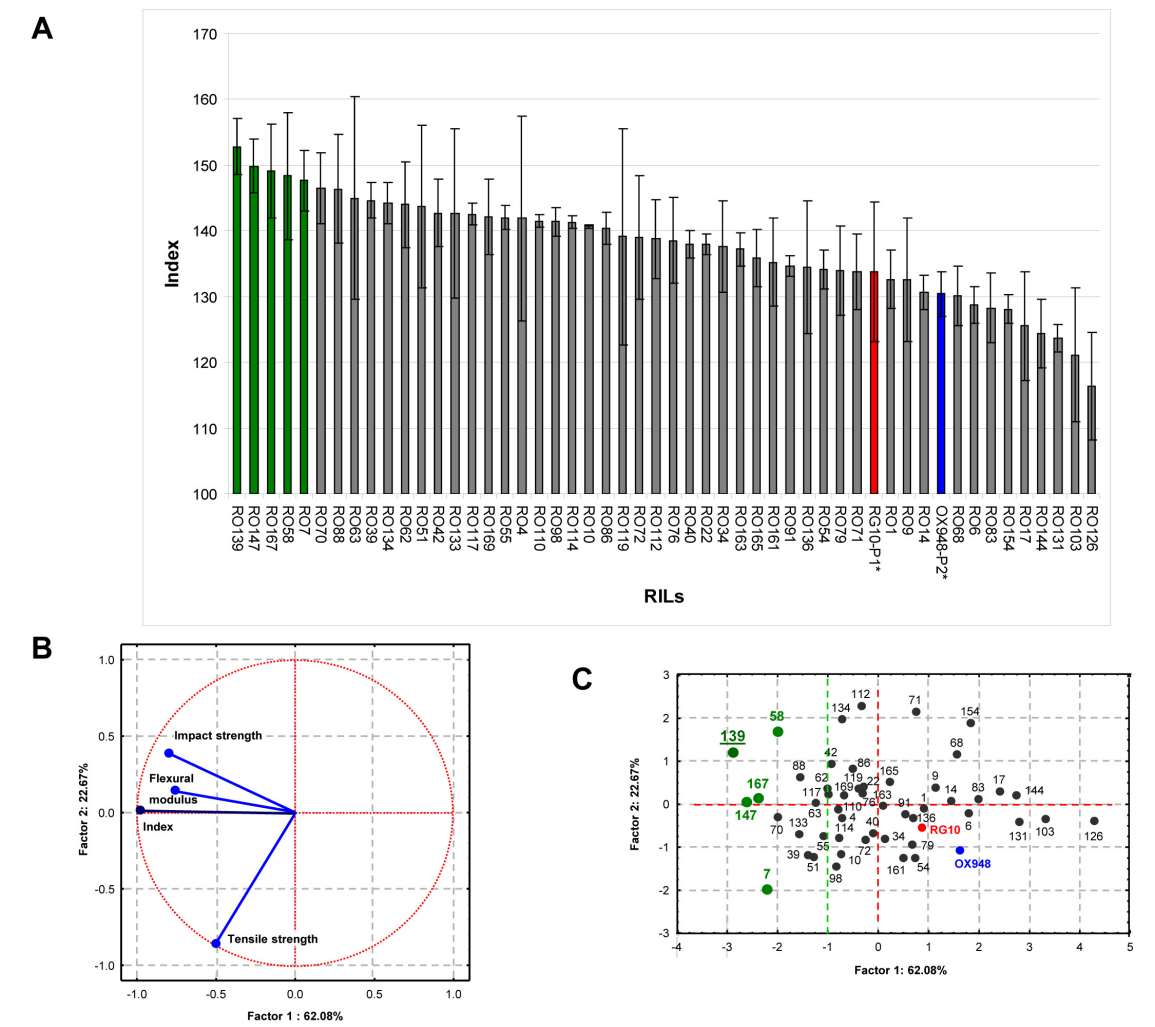
Mechanical properties of soybean stem fiber/polypropylene (SS/PP) composite materials. A. Index distribution. The index was calculated as Ʃ the percent difference for tensile strength from pure PP, the percent difference for flexural modulus from pure PP and the percent difference for impact strength from pure PP; B. Projection of the mechanical traits and index: factors 1x2; C. Projection of the soybean genotypes on the factor-plane: factors 1x2 explained 84.75% of the variability.

To identify RILs that can produce fibers for thermoplastic composites suitable for automotive parts manufacture, we developed a performance index based on three mechanical traits of the composites, namely: tensile strength (resistance to tension in material to break), flexural modulus (resistance of material to deformation under stress) and impact strength (resistance of material to withstand shock/ force). The index = Ʃ percent difference for tensile strength from pure PP, percent difference for flexural modulus from pure PP and percent difference for impact strength from pure PP. These parameters were chosen because they show the strength of material against the most common forces [tension (tensile), compression (flexural) and hammering (impact)] and can act together on a piece of composite material or its end product. The index values for the RILs ranged from 115–152 and the majority had higher values than the best parent (RG10 = 132). The line RO139 had the highest index value of 152.8 (Fig 2A). A principal component analysis (PCA) of the index data indicated that the most of the variation in the mechanical performance index was explained by three factors and a biplot with the first two factors explained 85% of the variation (Fig 2B). The plot shows that the index was highly aligned with impact strength and flexural modulus, but the index and the other two parameters were nearly independent of the tensile strength parameter.

**Fig 3:**
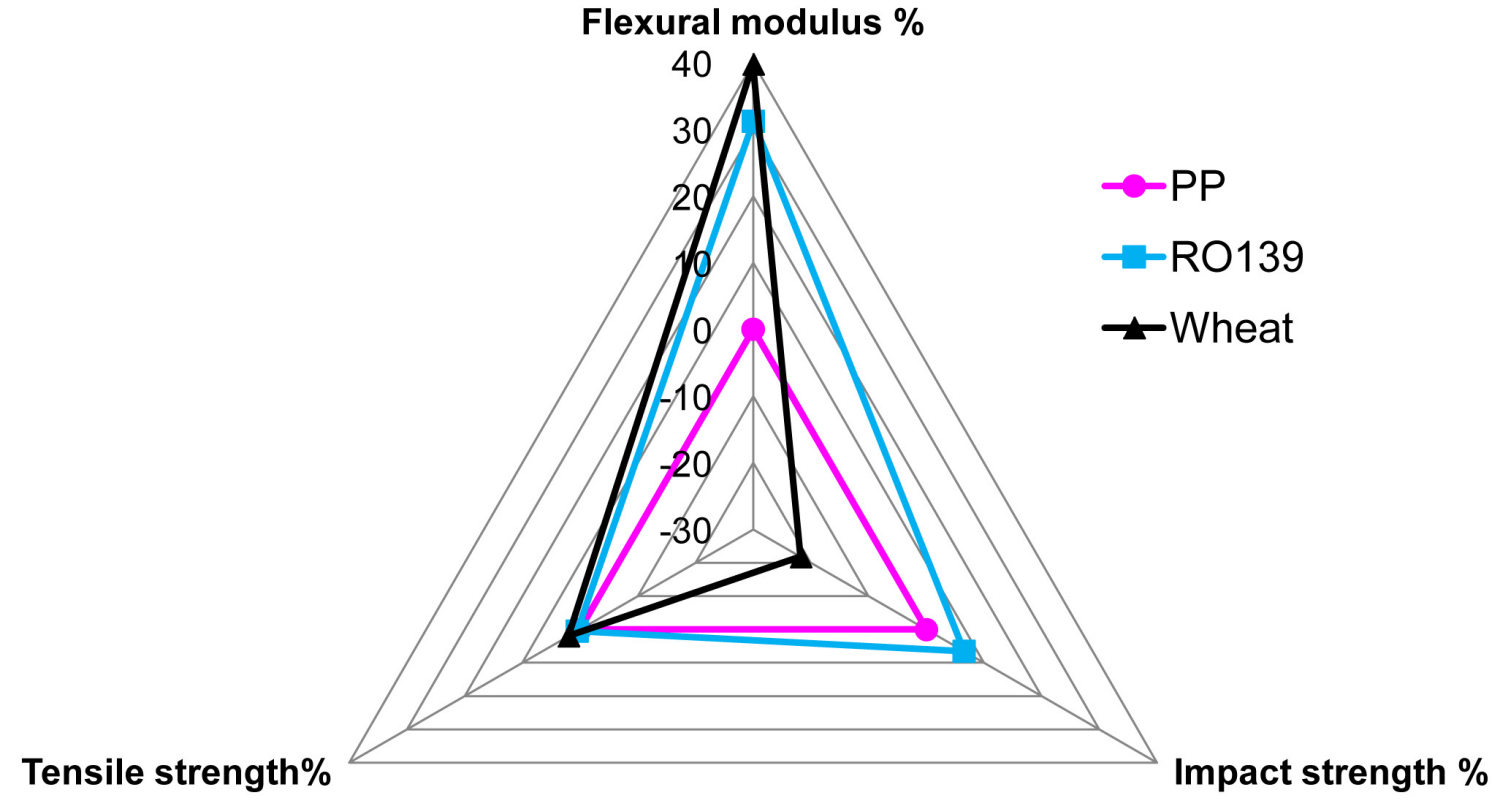
Mechanical trait-based index comparison of the best performing soybean (RO139) stem fiber/polypropylene (SS/PP) composite with wheat straw/polypropylene (WS/PP) composite and pure polypropylene (PP). Composites from RO139 (blue) were better than pure PP (magenta) for all three mechaical traits and outperformed WS/PP (black) for impact strength.

A PCA projection of genotype index values (Fig 2C; Table C in S1 File) identified a number of SS/PP composites (prepared with fibers from different RILs) that had superior index values, suggesting that they would be suitable for manufacturing various parts where impact strength and flexural modulus were important attributes. This analysis would also suggest that line RO139 is the best candidate, since it had the furthest distance from the origin in the double positive quadrant. A radar diagram indicated that composites from this line outperformed pure PP for all three mechanical traits (Fig 3). When compared to WS/PP composites, which already have application in auto industry, SS/PP composites produced from RO139 were better for impact strength and had similar values for tensile strangth. If the application required a composite material with more tensile strength then lines from the posive negative quadrant could be selected, such as RO7 (Fig 2C).

### QTL Identification

**Fig 4:**
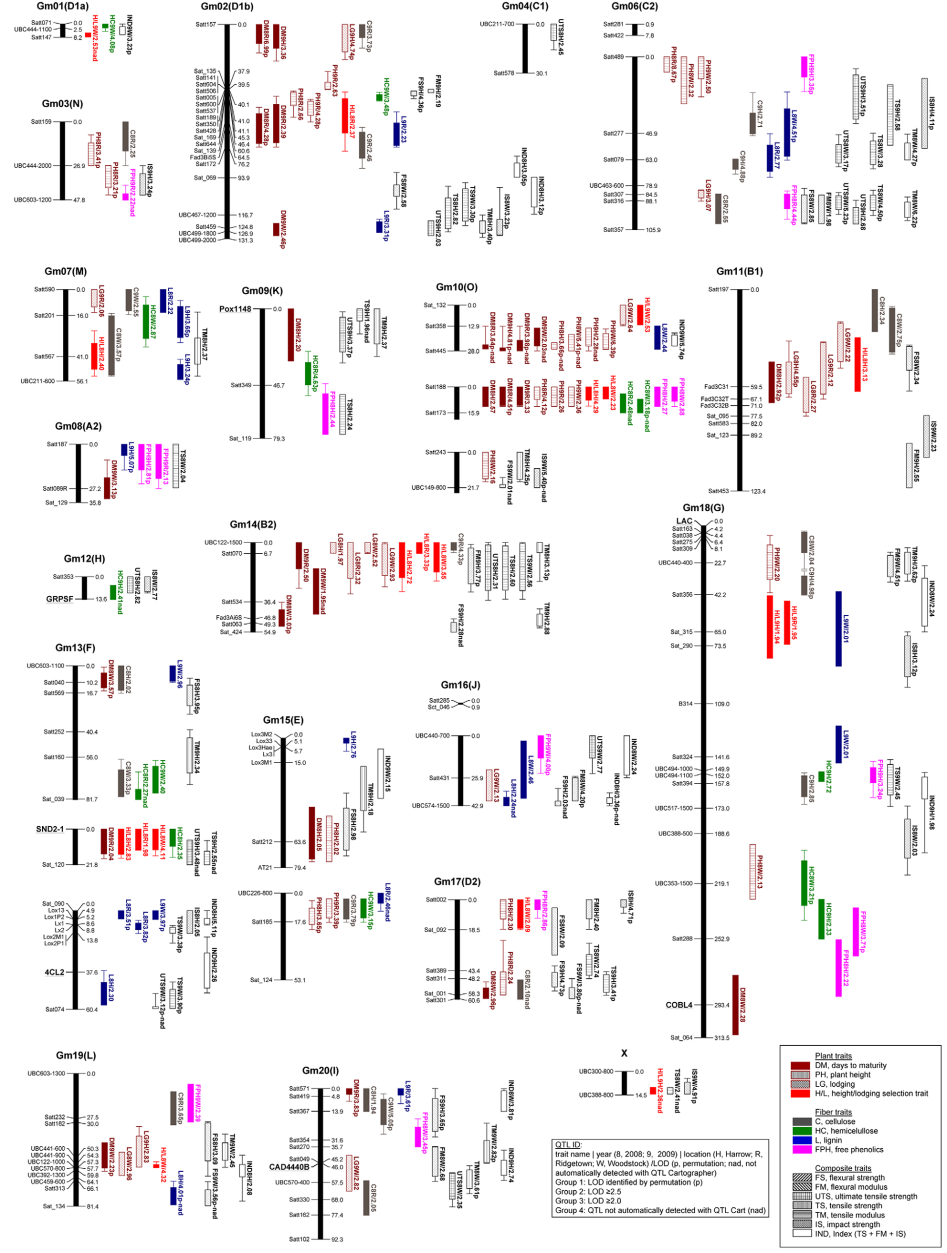
Soybean RG10 x OX948 stem fiber-based composite QTL map. QTL were detected using the Composite Interval Mapping (CIM) with Windows QTL Cartographer v.2.5_009 [The settings used: map function Kosambi, a walk speed of 2 cM, five control markers, model 6 (standard), forward and backward regression (method 3), and probabilities of 0.05]. The 1,000 permutation test at 0.05 significance level for CIM was used to determine LOD thresholds for each trait. Because of the novelty of some of the mapping traits, QTL at LOD threshold values ≥2.0 were also considered as putative QTL. The map positions of these QTL were detected using the option for automatic QTL location (using program's default parameters). Several QTL not automatically detected (nad) were also marked as putative when they exceeded threshold LOD scores.

The availability of a linkage map data for the soybean RG10 x OX948 population [51] that included the RILs used in the current study provided a unique opportunity to partition the genetic effects on fiber composition and composite physical traits among specific chromosomal locations through a QTL analysis. In addition, we were also able to locate the mapped SSRs and cell wall gene-specific markers in the soybean genome [46] to identify potential candidate genes associated with the QTL (Table D in S1 File). Furthermore, we defined and identified a novel type of QTL, we termed fiber composite performance QTL, which are soybean genomic regions associated with the functional properties of soybean stem fibers (flexural, impact and tensile) in thermoplastic composites. Because of the significant GxE effects (Table B in S1 File) QTL analyses were performed separately for each environment. Based on CIM analysis {a LOD [logarithm (base 10) of odds] score threshold, determined by 1,000 permutations, ranged from 2.2 to 4.7}, at least one QTL was identified for all 15 traits in at least one of six environments (Fig 4).We also considered as putative QTL those identified at LOD threshold ≥2. In total, 247 QTL were identified for four fiber traits, seven mechanical traits and four agronomic traits including a height/lodging (H/L) selection trait on 27 linkage groups that corresponded to 19 chromosomes (soybean reference genetic map—Gm composite_2003) and a linkage group X (which had only two UBC RAPD markers not mapped on the soybean 2003_composite map) (Fig F in S1 File). The inclusion of QTL identified at a relatively low threshold (LOD ≥2) could be justified by the novelty of this type of QTL. Since this was the first study of this nature, the focus of this work was to identify any genomic region potentially associated with fiber compositional and composite performance traits in soybean. Also, this would allow direct comparison with the QTL identified previously for some agronomic traits using the whole mapping population {n = 169, [51]}. The number of QTL identified per chromosome ranged from a single fiber composite performance QTL (UTS8H) on chromosome Gm04 (C1) to 28 QTL (agronomic, fiber compositional and fiber composite performance) on chromosome Gm10 (O). The highest number of QTL was identified for the days to maturity (25 QTL on 12 chromosomes) and the lowest number of QTL was identified for flexural modulus (eight QTL on eight chromosomes). In total, 96 and 99 QTL were identified for all 15 traits in Harrow and Woosdtock, respectively, in both years. Ridgetown was excluded from the QTL study of composite mechanical properties, which resulted in the smallest number QTL identified in this location. Fifty two QTL were mapped in Ridgetown for nine agronomic and fiber traits; no QTL for hemicellulose was detected in this location in the second year of the study. Genomic regions associated with analyzed traits accounted for 9% to 44% of total phenotypic variability for the specific trait (Fig 4; Tables 4 and 5).

**Table 4:** Quantitative trait loci (QTL) for agronomic and fiber compositional traits in 50 selected RG10 x OX948 recombinant inbred lines (RILs) in six environments.

Trait	Year	Chromosome (Linkage group)	Marker interval	Location								
				Harrow			Ridgetown			Woodstock		
				LOD	Additive	R^2^	LOD	Additive	R^2^	LOD	Additive	R^2^
Days to maturity (days)	2008	Gm02 (D1b)	Satt157-Sat_135				6.99	2.36	0.36			
		Gm02 (D1b)	Sat_169-Satt172				4.28	-1.87				
		Gm09 (K)	Pox1148-Satt349	2.20	-1.97	0.24						
		Gm10 (O)	Satt358-Satt445				3.64nad^a^	1.46	0.16			
		Gm10 (O)	Satt188-Satt173	**2.57**	**-1.49**	**0.14**	**4.51**	**-1.74**	**0.20**			
		Gm11 (B1)	Satt197-Fad3C	2.92	-1.74	0.19						
		Gm13 (F)	UBC603_.1100_-Satt569							3.57	-1.63	0.18
		Gm14 (B2)	Satt534-Sat_424							3.03	-1.66	0.16
		Gm15 (E)	Satt212-AT21	2.05	1.09	0.12						
		Gm17 (D2)	Satt311-Satt301							2.96	-1.55	0.14
		Gm18 (G)	COBL4-Sat_064							2.28	1.75	0.32
	2009	Gm02 (D1b)	Satt157-Sat_135	3.36	1.50	0.27						
		Gm02 (D1b)	Sat_169-Satt172				2.39	-1.62	0.12			
		Gm02 (D1b)	Satt459-UBC499_.2000_							2.46	0.37	0.14
		Gm08 (A2)	Satt187-Sat_129							3.13	0.50	0.20
		Gm10 (O)	Satt358-Satt445	**4.81nad**	**1.88**	**0.33**	**3.98nad**	**2.21**	**0.21**	**2.03nad**	**0.34**	**0.12**
		Gm10 (O)	Satt188-Satt173				3.33	-2.12	0.18			
		Gm13 (F)	SND2-Sat_120				2.04	-1.47	0.11			
		Gm14 (B2)	UBC122_.1500_-Satt070				2.50	-1.75	0.13			
		Gm14 (B2)	Satt070-Satt534							1.95	-1.29	0.14
		Gm19 (L)	Satt182-UBC122_.1000_							2.23	0.37	0.11
		Gm20 (I)	Satt571-Satt367				3.83	-2.15	0.17			
Plant height (cm)	2008	Gm02 (D1b)	Satt189-Sat_139				2.66	-3.30	0.13			
		Gm03 (N)	Satt159-UBC444_.2000_				3.41	4.12	0.22			
		Gm03 (N)	UBC444_.2000_-UBC603_.1200_				3.21	4.05				
		Gm06 (C2)	Satt489-Satt277				8.67	5.84	0.44	2.32	3.93	0.40
		Gm10 (O)	Satt358-Satt445	3.66nad	3.20	0.20				5.41nad	5.95	0.36
		Gm10 (O)	Satt188-Satt173				4.12	-3.52	0.17			
		Gm10 (O)	Satt243-UBC149_.800_							2.16	3.90	0.16
		Gm15 (E)	Satt212-AT21	2.02	2.91	0.26						
		Gm15 (E)	UBC226_.800_-Satt185	3.65	-3.65	0.38						
		Gm17 (D2)	Satt002-Sat_092	2.30	-2.19	0.11						
		Gm17 (D2)	Satt389-Satt001				2.40	-2.47	0.09			
		Gm18 (G)	UBC388_.500_-Satt288							2.13	5.98	0.34
	2009	Gm02 (D1b)	Sat_135-Satt189				2.63	-4.68	0.39			
		Gm02 (D1b)	Sat_169-Sat_139				4.28	-7.47				
		Gm06 (C2)	Satt489-Satt277							2.50	3.52	0.15
		Gm10 (O)	Satt358-Satt445	**2.28nad**	**4.38**	**0.20**				**6.39**	**6.27**	**0.39**
		Gm10 (O)	Satt188-Satt173				**2.26**	**-4.34**	**0.14**	**2.36**	**-3.26**	**0.13**
		Gm15 (E)	Satt185-UBC226_.800_				3.39	-5.96	0.28			
		Gm18 (G)	Satt309-Satt356							2.20	3.11	0.20
Lodging (scale)	2008	Gm11 (B1)	Satt197-Fad3C	**4.55**	**-0.29**	**0.42**	**2.62**	**-0.44**	**0.42**			
		Gm14 (B2)	UBC122_.1500_-Satt534	**1.97**	**-0.15**	**0.12**	**2.32**	**-0.34**	**0.18**	2.52	-0.54	0.16
		Gm19 (L)	UBC441_.600_-Satt313							2.96ad	-0.51	0.14
	2009	Gm02 (D1b)	Satt157-Sat_135	4.74	0.57	0.44						
		Gm06 (C2)	UBC463_.600_-Satt316	3.07	0.34	0.18						
		Gm07 (M)	SAtt590-Satt201				2.06	-0.24	0.11			
		Gm10 (O)	Sat_132-Satt358							2.64	0.32	0.16
		Gm11 (B1)	Satt197-Fad3C				**2.12**	**-0.45**	**0.38**	**2.22**	**-0.51**	**0.33**
		Gm14 (B2)	UBC122_.1500_-Satt070							2.93	-0.34	0.21
		Gm16 (J)	Satt431-UBC574_.1500_							2.13	-0.33	0.19
		Gm19 (L)	Satt182-UBC392_.1300_	2.83	-0.30	0.16						
		Gm20 (I)	Satt270-Satt049							2.82	0.32	0.16
Height per unit of lodging	2008	Gm02 (D1b)	Sat_169-Satt172				2.37	10.9	0.26			
		Gm07 (M)	Satt201-UBC211_.600_	2.40	-4.12	0.11						
		Gm10 (O)	Satt188-Satt173	4.29	6.14	0.25						
		Gm11 (B1)	Satt197-Fad3C	3.13	7.17	0.32						
		Gm13 (F)	SND2-Sat_120	**2.83**	**6.00**	**0.20**	**1.98**	**7.63**	**0.13**	**4.11**	**11.32**	**0.28**
		Gm14 (B2)	UBC122_.1500_-Satt070	**2.72**	**5.15**	**0.16**	**3.33**	**9.10**	**0.26**	**3.55**	**9.83**	**0.22**
		Gm17 (D2)	Satt002-Sat_092							2.09	8.62	0.15
	2009	Gm01 (D1a)	UBC444_.1100_-Satt147							2.53nad	6.86	0.17
		Gm10 (O)	Sat_132-Satt358							2.53	-7.27	0.20
		Gm18 (G)	Satt356-B314	**1.94**	**-9.92**	**0.19**	**1.95**	**-7.26**	**0.16**			
		X	UBC300_.800_-UBC388_.800_	2.36nad	-9.31	0.13						
Cellulose (ADF-ADL, %)	2008	Gm03 (N)	Satt159-UBC444_.2000_				2.25	0.62	0.22			
		Gm06 (C2)	Satt307-Satt357				2.55	-0.57	0.15			
		Gm07 (M)	Satt201-UBC211_.600_							3.57	1.40	0.25
		Gm11 (B1)	Satt197-Fad3C	2.34	1.00	0.30				2.75	1.57	0.34
		Gm13 (F)	Satt160-Sat)039							3.33	-1.39	0.29
		Gm13 (F)	UBC603_.1100_-Satt569	2.02	0.59	0.14						
		Gm17 (D2)	Satt001-Satt301				2.10nad	-0.61	0.14			
		Gm18 (G)	LAC-UBC440_.400_							2.04	-0.78	0.34
		Gm20 (I)	Satt571-Satt367	1.94	-0.58	0.12						
		Gm13 (F)	UBC570_.400_-Satt162				2.05	-0.55	0.13			
	2009	Gm02 (D1b)	Satt157-Sat_135				3.73	0.95	0.19			
		Gm02 (D1b)	Fad3B-Sat_069				2.46	-0.87				
		Gm06 (C2)	Satt489-Satt277	2.71	-0.87	0.35						
		Gm06 (C2)	Satt079-UBC463_.600_	4.88	1.32							
		Gm07 (M)	Satt590-Satt201							2.55	0.73	0.16
		Gm14 (B2)	UBC122_.1500_-Satt070				4.33	-1.04	0.23			
		Gm15 (E)	UBC226_.800_-Satt185				3.79	-1.04	0.24			
		Gm18 (G)	UBC440_.400_-Satt356	4.98	-1.07	0.36						
		Gm18 (G)	UBC494_.1000_-UBC517_.1500_	2.85	0.71							
		Gm19 (L)	UBC603_.1300_-Satt182				3.65	1.00	0.25			
		Gm20 (I)	SAtt571-Satt354							5.05	-1.02	0.30
Hemicellulose (NDF-ADF, %)	2008	Gm07 (M)	Satt590-Satt567							2.87	0.35	0.29
		Gm09 (K)	Pox1148-Satt349				4.63	0.40	0.44			
		Gm10 (O)	Satt188-Satt173				**2.48nad**	**0.22**	**0.15**	**3.18nad**	**0.30**	**0.21**
		Gm13 (F)	Satt160-Sat_039				2.27nad	0.24	0.26			
		Gm13 (F)	SND2-Sat_120	2.35	-0.19	0.20						
		Gm18 (G)	UBC388_.500_-Satt288							3.21	0.28	0.27
	2009	Gm01 (D1a)	Satt071-UBC444_.1100_							4.08	-0.39	0.20
		Gm02 (D1b)	Satt189-Sat_169							3.48	0.42	0.22
		Gm12 (H)	Satt353-GRPSF	2.41	0.28	0.19						
		Gm13 (F)	Satt160-Sat_039							2.40	0.44	0.25
		Gm15 (E)	UBC226_.800_- Satt185							3.15	0.45	0.27
		Gm18 (G)	UBC494_.1000_-Satt394	2.72	0.33	0.32						
		Gm18 (G)	UBC353_.1500_-Satt288	2.33	-0.40							
Lignin (ADL, %)	2008	Gm06 (C2)	Satt489-Satt079							**4.51**	**0.73**	**0.41**
		Gm06 (C2)	Satt277-UBC463_.600_				**2.77**	**0.36**	**0.16**			
		Gm07 (M)	Satt590-Satt201				2.22	0.35	0.15			
		GM10 (O)	Satt358-Satt445							2.44	0.50	0.17
		Gm13 (F)	Sat_090-Lox13				3.51	0.52	0.27			
		Gm13 (F)	Lx1-Lox2P1				3.82	0.41				
		Gm13 (F)	4CL2-Sat_074	2.30	0.44	0.22						
		Gm15 (E)	UBC226_.800_-Satt185				2.46	-0.36	0.15			
		Gm16 (J)	Satt431-UBC574_.1500_	2.24nad	0.38	0.14						
		Gm16 (J)	UBC440_.700_-UBC574_.1500_							**2.46**	**-0.58**	**0.20**
		Gm19 (L)	Satt313-Sat_134	**4.01nad**	**-0.64**	**0.38**						
	2009	Gm02 (D1b)	Sat_169-Satt175				2.23	-0.37	0.29			
		Gm02 (D1b)	UBC467_.1200_-Satt459				3.31	0.50				
		Gm07 (M)	Satt201-Satt567	3.65	0.64	0.33						
		Gm07 (M)	Satt567-UBC211_.600_	3.24	-0.46							
		Gm08 (A2)	Satt187-Satt089R	5.07	0.54	0.27						
		Gm13 (F)	UBC603_.1100_-Satt040							2.96	-0.60	0.20
		Gm13 (F)	Sat_090-Lox13							3.97	-0.70	0.27
		Gm15 (E)	Lox3M2-Lox3M1	2.76	0.35	0.12						
		Gm18 (G)	Satt356-Sat_290							2.01	-0.55	0.25
		Gm18 (G)	B314-UBC494_.1000_							2.01	0.52	
		Gm20 (I)	Satt571-Satt367				3.61	-0.49	0.23			
Free phenolics (μg mg^-1^)	2008	Gm06 (C2)	Satt307-Satt357				4.44	0.20	0.30			
		Gm09 (K)	Satt349-Sat_119	2.44	-0.14	0.30						
		Gm10 (O)	Satt188-Satt173	**2.27**	**0.10**	**0.15**				**2.88**	**-0.11**	**0.16**
		Gm17 (D2)	Satt002-Sat_092				2.86	-0.12	0.16			
		Gm18 (G)	Satt288-Sat_064	**2.22**	**-0.13**	**0.38**						
		Gm18 (G)	UBC353_.1500_-COBL4							**3.71**	**0.12**	**0.31**
		Gm20 (I)	Satt367-Satt270							3.45	0.13	0.22
	2009	Gm03 (N)	UBC444_.2000_-UBC603_.1200_				2.22	0.15	0.18			
		Gm06 (C2)	Satt489-Satt277	**3.35**	**-0.18**	**0.27**	**2.13**	**-0.15**	**0.21**			
		Gm08 (A2)	Satt187-Satt089R	2.81	0.18	0.20						
		Gm16 (J)	UBC440_.700_-Satt431							4.00	0.16	0.26
		Gm18 (G)	Satt324-Satt394	3.24	0.16	0.20						
		Gm19 (L)	UBC603_.1300_-Satt182							2.39	0.12	0.19

^a^QTL not automatically identified by QTL Cartographer

QTL identified in multiple environments are shown in bold

**Table 5:** Quantitative trait loci (QTL) for fiber mechanical performance in composites (fiber composite performance QTL) in 50 selected RG10 x OX948 recombinant inbred lines (RILs) in four environments.

Trait	Chromosome (Linkage group)	Marker interval	Environment											
			Harrow 2008			Woodstock 2008			Harrow 2009			Woodstock 2009		
			LOD	Additive	R^2^	LOD	Additive	R^2^	LOD	Additive	R^2^	LOD	Additive	R^2^
Flexural strength (MPa)	Gm02 (D1b)	Sat_135-Sat_169							4.36	0.66	0.20			
	Gm02 (D1b)	Sat_069-UBC467_.1200_				2.58	-1.12	0.40						
	Gm06 (C2)	Satt307-Satt357				2.85	0.79	0.22						
	Gm10 (O)	Satt243-UBC149_.800_										2.01nad^a^	0.66	0.12
	Gm11 (B1)	Satt197-Fad3C				2.34	-1.11	0.40						
	Gm13 (F)	Satt040-Satt252	3.95	-0.55	0.22									
	Gm14 (B2)	Fad3Ai6-Sat_424							2.28nad	0.52	0.12			
	Gm15 (E)	Lox3M1-AT21	2.98	0.46	2.98									
	Gm16 (J)	Satt431-UBC57_.1500_							2.03nad	0.43	0.10			
	Gm17 (D2)	Satt002-Satt389				2.09	-0.62	0.16						
	Gm17 (D2)	Satt389-Sat_001							**4.73**	**-0.62**	**0.21**			
	Gm17 (D2)	Satt311-Satt301										**3.80nad**	**0.87**	**0.20**
	Gm19 (L)	Satt182-UBC441_.900_	3.09	0.56	0.21									
	Gm19 (L)	Satt313-Sat_134										3.56nad	0.90	0.21
	Gm20 (I)	Satt419-Satt354							3.65	0.60	0.17			
Flexural modulus (MPa)	Gm02 (D1b)	Sat_135-Sat_169							2.19	18.81	0.11			
	Gm06 (C2)	Satt307-Satt357				1.98	28.71	0.21						
	Gm11 (B1)	Sat_123-Satt453							2.55	-30.78	0.29			
	Gm14 (B2)	UBC122_.1500_-Satt534							3.77	-28.01	0.31			
	Gm16 (J)	Satt431-UBC574_.1500_				4.20	-33.30	0.29						
	Gm17 (D2)	Satt002-Sat_092	2.40	-21.44	0.18									
	Gm18 (G)	Satt309-Satt356										4.51	-31.99	0.26
	Gm20 (I)	Satt270-UBC570_.400_				2.68	27.39	0.18						
Ultimate tensile strength (MPa)	Gm02 (D1b)	UBC467_.1200_-UBC499_.2000_										2.03	0.70	0.10
	Gm04 (C1)	UBC211_.700_-Satt578	2.45	-0.55	0.24									
	Gm06 (C2)	Satt489-Satt277							3.51	1.36	0.36			
	Gm06 (C2)	Satt277-Satt079				3.17	-0.64	0.31						
	Gm06 (C2)	Satt307-Satt357				**5.23**	**1.05**							
	Gm06 (C2)	Satt316-Satt357							**2.68**	**1.04**				
	Gm09 (K)	Pox1148-Satt349							3.37	1.46	0.36			
	Gm12 (H)	Satt353-GRPSF	2.82	-0.58	0.17									
	Gm13 (F)	SND2-Sat_120							3.48nad	1.20	0.27			
	Gm13 (F)	4CL2-Sat_074										3.12nad	-0.88	0.21
	Gm14 (B2)	UBC122_.1500_-SAtt534	2.31	-0.56	0.17									
	Gm16 (J)	UBC440_.700_-Satt431										2.77	-0.72	0.18
	Gm20 (I)	Satt049-Satt330				2.35	0.63	0.12						
Tensile strength (MPa)	Gm02 (D1b)	Sat_069-UBC499_.1800_	**2.82**	**-0.54**	**0.18**									
	Gm02 (D1b)	Sat_069-Satt459										**3.30**	**-0.88**	**0.28**
	Gm06 (C2)	Satt489-Satt079							**2.58**	**0.74**	**0.37**			
	Gm06 (C2)	Satt277-Satt079				**3.28**	**-0.64**	0.29						
	Gm06 (C2)	Satt307-Satt357				4.50	0.87							
	Gm08 (A2)	Satt187-Satt089R				2.04	-0.53	0.14						
	Gm09 (K)	Pox1148-Satt349							1.96	0.57	0.34			
	Gm09 (K)	Satt349-Sat_119	2.24	-0.63	0.20									
	Gm13 (F)	SND2-Sat_120							2.55	0.92	0.20			
	Gm13 (F)	Lx1-Lox2M1										3.38	0.88	0.26
	Gm13 (F)	4CL2-Sat_074										3.90	-0.84	
	Gm14 (B2)	UBC122_.1500_-Satt534	2.60	-0.68	0.27							2.56	0.67	0.19
	Gm17 (D2)	Sat_092-Satt311				2.74	-0.49	0.15						
	Gm17 (D2)	Satt389-Satt001							3.41	-0.89	0.20			
	Gm18 (G)	Satt324-UBC517_.1500_										2.45	-0.54	0.11
	X	UBC300_.800_-UBC388_.800_				2.41nad	-0.51	0.12						
Tensile modulus (MPa)	Gm02 (D1b)	UBC467_.1200_-UBC499_.1800_	3.40	-12.70	0.17									
	Gm06 (C2)	Satt277-Satt079				4.27	-18.69	0.45						
	Gm06 (C2)	Satt307-Satt357				6.22	25.07							
	Gm07 (M)	Satt201-Satt567	2.37	-15.83	0.28									
	Gm09 (K)	Pox1148-Satt349							2.37	13.14	0.33			
	Gm10 (O)	Satt243-UBC149_.800_	4.25	-15.04	0.31									
	Gm13 (F)	Satt252-Sat_039							2.34	9.94	0.16			
	Gm14 (B2)	UBC122_.1500_-Satt070	3.13	-12.61	0.20									
	Gm14 (B2)	Satt534-Satt063							2.88	-9.85	0.24			
	Gm15 (E)	Lox33-Satt212							2.18	-12.18	0.26			
	Gm18 (G)	Satt309-Satt356							3.62	11.06	0.26			
	Gm19 (L)	Satt182-UBC122_.1000_										2.45	-9.90	0.19
	Gm20 (I)	Satt354-Satt049										2.82	11.71	0.19
	Gm20 (I)	Satt049-Satt330				3.61	14.78	0.21						
Impact strength (μg mg^-1^)	Gm02 (D1b)	UBC467_.1200_-UBC499_.2000_				3.23	1.24	0.21						
	Gm03 (N)	UBC444_.2000_-UBC603_.1200_							3.24	-1.37	0.30			
	Gm06 (C2)	Satt489-Satt277	4.11	1.40	0.40									
	Gm10 (O)	Satt243-UBC149_.800_										5.40nad	2.25	0.39
	Gm11 (B1)	Sat_095-Satt453										2.23	1.14	0.15
	Gm12 (H)	Satt353-GRPSF				2.77	1.10	0.16						
	Gm13 (F)	Sat_090-Lox2M1	2.05	0.77	0.12									
	Gm17 (D2)	Satt002-Sat_092	4.71	-1.19	0.23									
	Gm18 (G)	Sat_315-B314	3.12	-1.34	0.36									
		UBC517_.1500_-UBC353_.1500_				2.03	-1.01	0.19						
	X	UBC300_.800_-UBC388_.800_										4.91	2.02	0.35
Index	Gm01 (D1a)	Satt071-Satt147										3.23	8.62	0.18
	Gm02 (D1b)	Satt172-Sat_069	3.05	-7.29	0.34									
	Gm02 (D1b)	Sat_069-UBC467_.1200_	3.12	-6.62										
	Gm10 (O)	Satt358-Satt445										5.74	18.4	0.40
	Gm13 (F)	Lox13-Lx1	5.11	7.99	0.33									
	Gm13 (F)	Lox2M1-4CL2							2.26	6.07	0.17			
	Gm15 (E)	Lox33-Satt212										2.15	-6.09	0.15
	Gm16 (J)	UBC440_.700_-Satt431				2.24	-3.63	0.13						
	Gm16 (J)	Satt431-UBC574_.1500_	3.36nad	6.36	0.23									
	Gm18 (G)	UBC440_.400_-Sat_315				2.24	-3.92	0.18						
	Gm18 (G)	Satt394-UBC517_.1500_							1.98	5.16	0.29			
	Gm19 (L)	UBC441_.900_-Satt313	2.08	-4.63	0.14									
	Gm20 (I)	Satt571-Satt367				3.81	-4.96	0.21						
	Gm20 (I)	Satt270-UBC570_.400_							2.74	5.86	0.17			

^a^QTL not automatically identified by QTL Cartographer

QTL identified in multiple environments are shown in bold

#### QTL for agronomic traits

Currently, 487 QTL for maturity, plant height, lodging and height per unit of lodging [159 maturity QTL (20 chromosomes, 32 studies), 187 plant height QTL (20 chromosomes, 35 studies), 80 lodging QTL (19 chromosomes, 27 studies) and 11 height per unit of lodging QTL (four chromosomes, four studies)] have been identified in different populations and are deposited in SoyBase (available at www.soybase.org; accessed 14 Mar 2015). Current work identified 82 QTL for these traits using a set (50 RILs) of the RG10 x OX948 population (n = 169) in three locations over two years. Twenty five QTL were detected for plant height on 12 chromosomes and explained up to 36% of the phenotypic variability for the trait. Twenty three QTL were identified for plant height on seven chromosomes. These QTL explained up to 44% of total phenotypic variability for the trait. Sixteen QTL for lodging were mapped on nine chromosomes and explained 11% to 44% variability for the trait. Using the set of 50 RILs, this study confirmed several maturity QTL (on chromosomes Gm02, Gm08, Gm10, Gm11 and Gm13), plant height QTL (chromosomes Gm03, Gm06, Gm10 and Gm18) and lodging QTL (on chromosomes Gm11 and Gm19) identified previously with the whole population {n = 169, [51]}. Eighteen QTL for the plant height to lodging ratio (H/L) selection trait were mapped on nine chromosomes and linkage group X and explained up to 32% phenotypic variability for the trait. Eleven QTL for this trait were identified in soybean previously {SoyBase, available at www://soybase.org; accessed 14 Mar 2015; [77]} and were mapped on four chromosomes (Gm07, Gm13, Gm18 and Gm19) in four independent mapping populations [78, 79]. Although some H/L QTL identified in the current study mapped to the same regions as QTL identified earlier (eg. QTL on chromosomes Gm07, Gm13, Gm18 and Gm19), the majority of the H/L QTL mapped to new genomic regions on six different chromosomes (Gm01, Gm02, Gm10, Gm11, Gm14 and Gm17) and linkage group X (Fig 4; Table 4).

#### Fiber compositional QTL

Seventy three QTL were identified for fiber compositional traits in six environments. They mapped to 18 chromosomes and explained significant portions of the phenotypic variation for individual fiber traits. Twenty two QTL for cellulose (C) content were mapped on 12 chromosomes and explained 12% to 36% variability for the trait. Fourteen QTL for hemicellulose (HC) content were identified on nine chromosomes and phenotypic variation explained by them ranged from 15% to 44%. Twenty two QTL for lignin (L) content explained 12% to 41% of the trait variabilty and were mapped on 11 chromosomes. Fifteen QTL for free phenolics (FPH) were identified on ten chromosomes and explained 15% to 38% phenotypic variability of the trait (Fig 4; Table 4). No QTL for these traits were mapped in soybean previously. Some clustering of QTL for soybean stem fiber cellulose, hemicellulose, lignin and free phenolics contents was observed, especially hemicellulose, cellulose, and lignin QTL on chromosomes Gm02 (HC9W, C9R, L9R), Gm07 (HC8W, C8W, C8W, L8R, L9H), Gm13 (HC8R, HC9W, C8W) and Gm18 (HC9H, C9H, L9W), respectively. In addition, our ability to connect the linkage map with the annotated genome sequence for soybean through the SSR markers identified several examples where these QTL were associated with the genome regions containing among others cell wall regulatory and biosynthetic genes (Fig G in S1 File). For example, under the cellulose QTL C9H on chromosome Gm06, two cellulose synthase genes [Glyma.06g225400-CesA(TC219882c) and Glyma.06g225500-CCR2(TC219882a)], the COBRA-like gene (Glyma.06g205500-COBL4) and several Myb transcription factors (Glyma.06g213400, Glyma.06g248200) were observed. Similarly, the lignin QTL L9H region on chromosome Gm07 contained, among others, a cinnamoyl CoA reductase gene [Glyma.07g026300-CCR2(TC219427)] involved in lignin biosynthesis and several regulatory genes (such as NAM transcription factor Glyma.07g050600 or Myb transcription factor Glyma.07g037700) (Fig G in S1 File).

#### Fiber composite performance QTL

Ninety two QTL were detected for the composite mechanical traits in four environments. The QTL were distributed across the entire soybean genome and explained significant portion of phenotypic variability for individual composite mechanical traits. Fifteen QTL for flexural strength (FS) were mapped on 11 chromosomes and explained 10% to 40% of the phenotypic variability for the trait. Eight QTL for flexural modulus (FM) were mapped on eight chromosomes and explained 11% to 31% phenotypic variability for the trait. Fifteen QTL for ultimate tensile strength (UTS) were mapped on ten chromosomes and explained 12% to 36% phenotypic variability for the trait. Fifteen QTL for tensile strength (TS) were mapped on seven chromosomes and linkage group X. These QTL explained 11% to 37% phenotypic variability for the trait. Fourteen QTL for tensile modulus (TM) were mapped on 11 chromosomes and explained 16% to 31% variability for the trait. Eleven QTL for impact strength (IS) were mapped on nine chromosomes and linkage group X and explained 12% to 40% phenotypic variability for the trait. Fourteen QTL for mechanical index (IND) were mapped on nine chromosomes and explained 13% to 40% phenotypic variability for the trait. Some QTL for flexural strength and modulus, tensile strength and modulus and impact strength were clustered on several chromosomes (Fig 4; Table 5). No QTL for composite mechanical traits were mapped in plants previously.

### QTL Consistency Across Environments

Significant GxE interactions were identified for the majority of the traits analyzed in this study (Table B in S1 File). Therefore, trait values could not be averaged over environments and QTL analysis was performed for each environment separately. The variability in the expression of these QTL was consistent with the low correlations observed among the fiber compositional and composite mechanical performance properties in different locations and years (S1 Table). The enzymes and regulatory proteins involved in the biosynthesis and modification of cellulose, hemicellulose and lignin are encoded by hundreds of genes, which often belong to large gene families (http://cellwall.genomics.purdue.edu/families/index.html), whose expression is affected by environmental conditions [10, 80, 81]. To be useful for breeding, the consistency of these QTL need to be tested in different environments and/or genetic backgrounds.

In general, the major effect QTL that were identified in the current study for four agronomic traits were stable over locations and/or years (Fig 4; Tables 4 and 5). Moreover, using a set of 50 RILs, this work confirmed positions and effects of several QTL for days to maturity, plant height and lodging, identified previously with the complete RG10 x OX948 population (n = 169), performed under a different set of environments [51]. These QTL, which are associated with the genes in some cases, have the greatest potential for application in plant breeding.

A number of fiber compositional QTL were consistently identified in several environments, including: lignin QTL (L8W and L8R) on chromosome Gm06, free phenolics QTL (FPH9R and FPH9H) on chromosome Gm08, hemicellulose QTL (HC8W and HC8R) on chromosome Gm10, and cellulose QTL (C8H and C8W) on chromosome Gm11. These QTL may be important in breeding for cell wall compositional traits. Because of the complexities involved in assesing the newly defined fiber composite performance QTL, they were assayed with fibers obtained from the two most divergent locations (Harrow and Woodstck) over two years. These QTL were sensitive to environmental conditions and the most of them were location or year specific. These QTL might be easier to detect if a number of environments were increased [82]. Also, by averaging trait values over environments specific environmental effects would likely be avoided [79]. The inconsistencies of these QTL over environments could be the major limitation of their application in MAS for a specific trait. Also, because of some large gaps in the linkage map and incomplete genome coverage [51] it is possible that some QTL were not identified. In addition, by using a subset of 50 RILs from the mapping population (n = 169), only major effect QTL could be potentially detected. By increasing number of markers and /or population size some of the missing QTL might be identified.

### Co-localization of Fiber Composite Performance QTL, Fiber Compositional QTL and Cell Wall Biosynthesis Genes

The level of polymorphism in mapping populations depends on the diversity of the genetic background of parental genotypes used to develop the population and type of the markers used in the study. We found relatively low levels of polymorphism for random (RAPD and SSR) markers in the RG10 x OX948 mapping population previously [51]. Similarly, only a few cell wall biosynthetic (regulatory and structural) gene-based markers (Table A in S1 File) were polymorphic between parents (RG10 and OX948) in this study.

Nevertheless, several fiber traits were associated with specific cell wall biosynthetic genes mapped in the RG10 x OX948 population. For example, impact strength QTL IS8W on chromosome Gm12 (Fig 4; Fig G in S1 File) co-localized with fiber QTL HC9H (hemicellulose) and both were associated with the gene coding for glycine-rich protein (GRP). This gene-spercific marker explained 13% phenotypic variability for the impact strength in Woodstock in 2008. The superfamily of plant glycine-rich proteins (GRPs) is characterized by the presence of semi-repetitive glycine-rich motifs and diversity in structure, expression and localization. GRPs may act as scaffolds for the deposition of cell wall constituents, during protoxylem development and in cell wall fortification by connecting lignin rings [83, 84]. In addition, it was shown that Arabidopsis *AtGRP9* interacts with *AtCAD5* (which catalyzes the last step in monolignol biosynthesis [85]–the reduction of cinnamaldehydes into cinnamyl alcohols) suggesting the involvement of GRPs in lignin biosynthesis/deposition [86].

Similarly, the flexural modulus QTL FM8W on chromosome Gm20 co-localized with six composite performance QTL [two index QTL (IND8W and IND9H), ultimate tensile strength QTL (UTS8W), flexural strength QTL (FS8W) and two tensile modulus QTL (TM8W and TM9W)] and two fiber composition QTL [free phenolics QTL (FPH8W) and cellulose QTL (C8R)]. These QTL were associated with the cinnamyl alcohol dehydrogenase (*CAD*) gene, which explained 10% of the phenotypic variability for the flexural modulus in Woodstock in 2008. CAD catalyses the conversion of cinnamyl aldehydes, with a NADPH cofactor, to cinnamyl alcohols, which are precursors to lignin [85]. Therefore, markers developed for these genes could assist breeding efforts for soybean lines with higher stem lignin content with potential application in composite manufacturing. However, because fiber composition and fiber performance in composite materials traits are quantitative in nature, encoded by a number of genes with small effects, more work is required in this area.

An example of overlapping fiber composite performance QTL occurred on chromosome Gm06 (Fig 4; Fig G in S1 File) between markers UBC463-600 and Satt357 (27 cM) where a QTL for flexural modulus FM8W overlapped with several other composite performance QTL, including: two ultimate tensile stregth QTL (UTS9H and UTS8W), a flexural sterngth QTL (FS8W), a flexural modulus QTL (FM8W) and a tensile strength QTL (TS8W). In addition, it co-localized with two fiber composition QTL [C8R (cellulose) and FPH8R (free phenolics)] and a lodging QTL (LG9H). Because of low polymorphism in the RG10 x OX948 population, we were not able to map any of the candidate cell wall synthesis genes directly in this region. However, the *in silico* map (Fig G in S1 File) contains numerous potential cell wall candidate genes in this region, including: glycosyl hydrolase 5 (Cell.GlyH5), cell wall protein (CWall), xyloglucan endo-transglycosylase (XET), cytochrome P450 (P450), peroxidase (Pox), cellulose synthase (CesA) and a number of transcription factors.

Similarly, two QTL for tensile strength (TS8W and TS9H) on chromosome Gm06 overlaped with a QTL for impact strength (IS8H), a QTL for tensile modulus (TM8W) and two QTL for ultimate tensile sterngth (UTS9H and UTS8W), and these co-localized with a number of fiber composition QTL for lignin (L8W and L8R), cellulose (C9H) and free phenolics (FPH9H)) and three plant height QTL (PH9W, PH8W and PH8R). The *in silico* map for this region contains cell wall-related genes, such as genes coding for expansin (EXP), Myb305-like transcription factor (Myb305), glycosyl-phosphatidyl inositol anchored protein [COBRA(TC216062)], flavonoid 3'-hydroxylase (F3'H), peroxidase (Pox), ABC transporter (ABCtr) or cellulose synthase (CesA). A gene coding for a member of a COBRA-like protein family (COBL4) was mapped on chromosome Gm18 (Fig 4; Fig G in S1 File). Maize brittle stalk2 (*bk2*) encodes a COBRA-like protein (similar to phytochelatin synthetase), which is involved in secondary cell wall biosynthesis [87]. It is expressed in early organ development but, it is also required for tissue flexibility at maturity. Mutants make smaller plants with reduced levels of cellulose and cell wall sugars. In general, cell wall biosynthetic (structural and regulatory) genes are encoded by gene families. In addition, extensive duplication exists in the soybean genome [88].

Several genomic regions contained clusters of linked and/or peiotropic loci that affected a number of traits (Fig 4; Tables 4 and 5). For example, SND2-Sat_120 marker interval on chromosome Gm13 (F) was associated with QTL for days to maturity (DM9R), height per unit of lodging (HL08H, HL08R, HL08W), ultimate tensile strength (UTS9H) and tensile strength (TS9H). Similarly, marker interval (UBC122_-1500_-Satt070-Satt534) on chromosome Gm14 (B2) was associated with QTL for several traits, inclusing days to maturity (DM9R), lodging (LG8H, LG8R, LG8W, LG9W), height per unit of lodging (HL8H, HL8R, HL8W), cellulose (C9R), flexural modulus (FM9H), ultimate tensile strength (UTS8H), tensile strength (TS8H, TS9W) and tensile modulus (TM8H). Pleoitropy is usually associated with major gene effects [89]. A common genetic basis might explain some correlations between agronomic, fiber compositional and composite traits. Alternatively, clustering of genes for different traits might be the basis for overlapping QTL. Mansur et al [90] and Orf et al [79] observed clustering of QTL with strong effects on maturity, plant height and lodging. However, the marker density in the current map was not sufficient to determine if the regions significant for more than one trait were the result of pleiotropy or gene linkage. Currently, a work to add microarray-based single feature polymorphism (SFP) markers to the map is underway. A more saturated map would help to resolve this ambiguity.

## Conclusion

The use of plant fibers in automotove parts is limited by their variability and poor performance when incorporated into composites. Our work indentified connections between the structure and chemistry of soybean stem fibers and their performance in thermoplastic composites. The results demonstrate that the performance of soybean stem fibers in composites was signifcantly affected by both genotypes and evironments. This study provides an understanding of the cell wall compositional traits that are important for the use of soybean stem fibers in composites. In particular, the lignin content of stem fibers (from some environments) was positively correlated with certain composite mechanical traits. For example, higher lignin contents in the fibers used to produce composites will enhance their mechanical properties such as flexural modulus, tensile strength and impact strength and determine their use in specific applications. We also developed SS/PP composites from a number of RILs that performed better than pure PP. Above all, most of the SS/PP composites had the values of flexural modulus higher than pure PP. The superior mechanical performance characterisitcs of SS/PP composites indicated that the soybean stem fibers have structural (reinforcing) roles in the composites and are not simply fillers. For the production of automotive interior parts, composite materials need to be strong and flexible to maintain durability. Moreover, the finding that the SS/PP composites were in some cases better that the WS/PP composites that are already used in the production of automotive interior parts suggests that the SS/PP materials could also be commercialized. With the additional adavantage that the SS/PP composites are relatively easy to manufacture and mold, compared to glass fiber composites, the current results make these materials potentially useful in injection-moulded or directly-formed automotive parts. Numerous QTL for fiber compositional and composite mechanical traits were detected across the entire soybean genome that explained significant portions of phenotypic variation for a specific trait. Co-segregation analysis with fiber compositional QTL as well as *in silico* mapping resulted in the identification of cell wall biosynthesis genes that co-localize with fiber composite performance QTL. Furthermore, several gene-based markers that were developed might allow rapid introgression of genes related to good fiber quality into elite germplasm. However, more work in this area, including QTL confirmation, fine mapping and functional analysis of candidate genes underlying QTL are needed.

## Supporting Information

S1 File Appendix. Distribution of height per unit of lodging (H/L) selection trait in 50 recombinant inbred lines (RILs) from the RG10 x OX948 cross. Lines were selected from an existing, well characterized population of n = 169 RILs **(Fig A)**. Mean temperature (^o^C) and total precipitation (mm) for soybean growing seasons in Harrow, Ridgetown and Woodstock (ON, Canada) in 2008 and 2009. a) 2008; b) 2009 **(Fig B)**. Frequency distribution of traits (raw data) analyzed in parental genotypes (RG10 and OX948) and 50 selected RG10 x OX948 recombinant inbred lines (RILs). a) agronomic traits; b) fiber compositional traits; c) composite mechanical properties **(Fig C)**. Distribution of 15 traits in parental genotypes (RG10 and OX948) and 50 selected RG10 x OX948 recombinant inbred lines (RILs). a) agronomic traits; b) fiber compositional traits; c) composite mechanical properties **(Fig D)**. Ground dry stem fibers from selected soybean lines. a) light brown color; b) deep brown color **(Fig E)**. Distribution of quantitative trait loci (QTL) LOD scores. a) QTL for agronomic and fiber compositional traits in six environments; b) QTL for fiber mechanical performance in four environments. QTL were detected using the Composite Interval Mapping (CIM) with Windows QTL Cartographer v.2.5_009. The settings used: map function Kosambi, a walk speed of 2cM, five control markers, model 6 (standard), forward and backward regression (method 3), and probabilities of 0.05. The 1,000 permutation test at 0.05 significancs level for CIM was used to determine LOD thresholds for each trait **(Fig F)**. Comparison of the soybean RG10 x OX948 stem fiber-based composite QTL map (right) with the *G*. *max* Wm82.a2.v1 sequence map (left). Linkage map—QTL were detected using the Composite Interval Mapping with Windows QTL Cartographer v.2.5_009 [The settings used: map function Kosambi, a walk speed of 2cM, five control markers, model 6 (standard), forward and backward regression (method 3), and probabilities of 0.05]. Sequence (*in silico*) map—Initial mapping was done by BLASTing cell wall gene sequences against soybean genome (*G*. *max* Wm82.a2.v1) in Phytozome 9.1; additional sequences were then added to newly identified QTL regions [by scaning (200 kb walk) the soybean genome for genes potentially involved in cell wall biosynthesis/modifcation in Phytozome 9.1 and/or using *G*. *max* Wm82.a2.v1 annotation and feature coordinate files from SoyBase]. Maps were linked by common SSR markers. Mapped fiber genes are indicated in bold **(Fig G)**. Cell wall-related gene-based PCR primers **(Table A)**. Analysis of variance (Fisher test values) for agronomic, fiber compositional and composite mechanical traits **(Table B)**. Comparison of mechanical performance of stem fibers in soybean/polypropylene (SS/PP) composites with pure polypropylene (PP) and wheat straw/polypropylene (WS/PP) composites **(Table C)**. Soybean (*Glycine max* Wm82.a2.v1) sequence map (partial, Phytozome v9.1) **(Table D)**.
(PDF)

S1 Table Correlations between agronomic, fiber compositional and composite performance traits in 50 selected RG10 x OX948 recombinant inbred lines (RILs) in different environments. (XLSX)
