# Supplementary material for: Genome Regions Associated with Functional Performance of Soybean Stem Fibers in Polypropylene Thermoplastic Composites
Source: PLoS One. 2015 Jul 13;10(7):e0130371. doi: 10.1371/journal.pone.0130371 (PMC4500502; doi:10.1371/journal.pone.0130371)
Supplement: S1 File — Distribution of height per unit of lodging (H/L) selection trait in 50 recombinant inbred lines (RILs) from the RG10 x OX948 cross. Lines were selected from an existing, well characterized population of n = 169 RILs (Fig A). Mean temperature (oC) and total precipitation (mm) for soybean growing seasons in Harrow, Ridgetown and Woodstock (ON, Canada) in 2008 and 2009. a) 2008; b) 2009 (Fig B). Frequency distribution of traits (raw data) analyzed in parental genotypes (RG10 and OX948) and 50 selected RG10 x OX948 recombinant inbred lines (RILs). a) agronomic traits; b) fiber compositional traits; c) composite mechanical properties (Fig C). Distribution of 15 traits in parental genotypes (RG10 and OX948) and 50 selected RG10 x OX948 recombinant inbred lines (RILs). a) agronomic traits; b) fiber compositional traits; c) composite mechanical properties (Fig D). Ground dry stem fibers from selected soybean lines. a) light brown color; b) deep brown color (Fig E). Distribution of quantitative trait loci (QTL) LOD scores. a) QTL for agronomic and fiber compositional traits in six environments; b) QTL for fiber mechanical performance in four environments. QTL were detected using the Composite Interval Mapping (CIM) with Windows QTL Cartographer v.2.5_009. The settings used: map function Kosambi, a walk speed of 2cM, five control markers, model 6 (standard), forward and backward regression (method 3), and probabilities of 0.05. The 1,000 permutation test at 0.05 significancs level for CIM was used to determine LOD thresholds for each trait (Fig F). Comparison of the soybean RG10 x OX948 stem fiber-based composite QTL map (right) with the G. max Wm82.a2.v1 sequence map (left). Linkage map—QTL were detected using the Composite Interval Mapping with Windows QTL Cartographer v.2.5_009 [The settings used: map function Kosambi, a walk speed of 2cM, five control markers, model 6 (standard), forward and backward regression (method 3), and probabilities of 0.05]. Sequence (in silico) [file pone.0130371.s001.pdf]

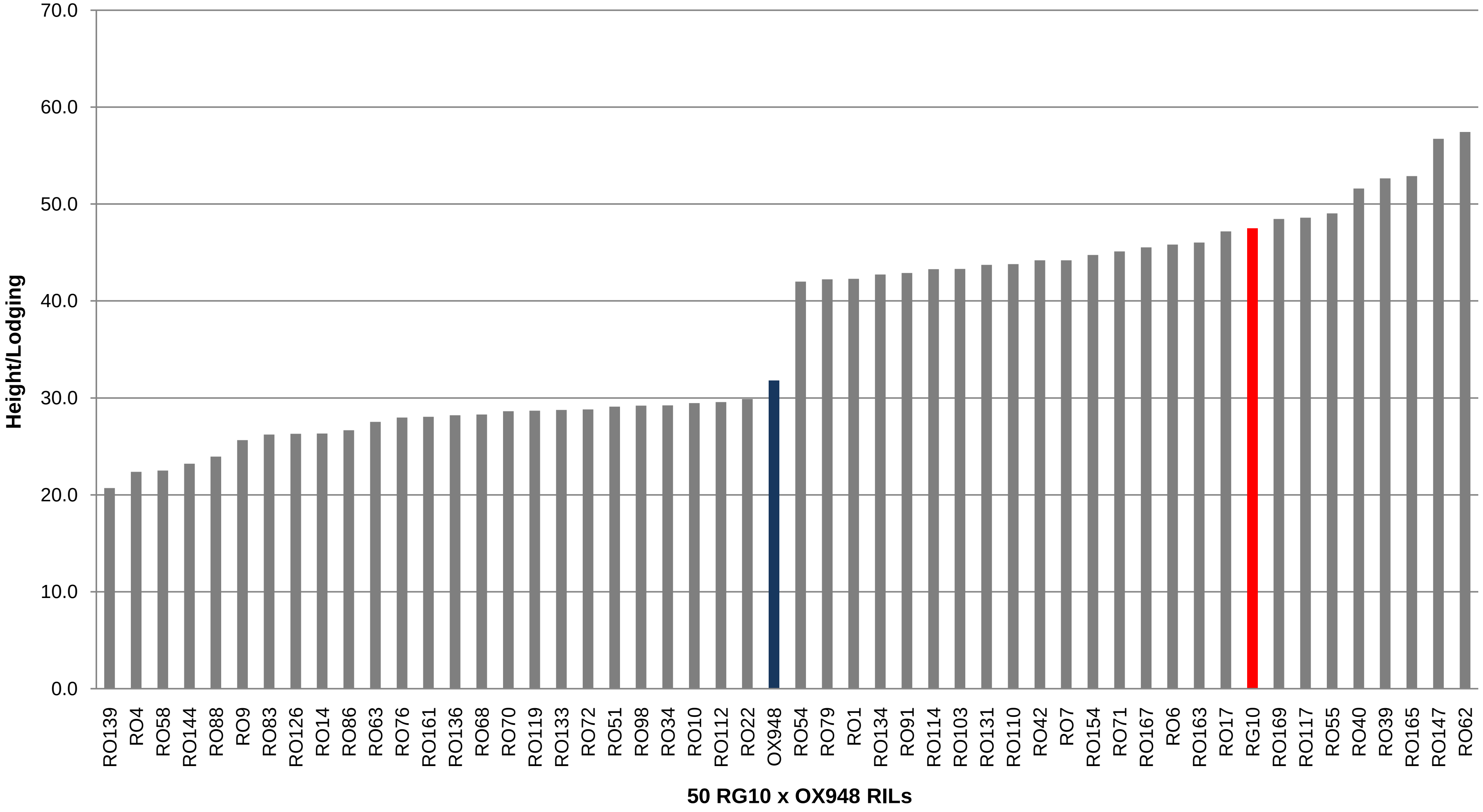

Fig. A in S1 File. Distribution of height per unit of lodging (H/L) selection trait in 50 recombinant inbred lines (RILs) from the RG10 x OX948 cross. Lines were selected from an existing, well characterized population of n = 169 RILs.

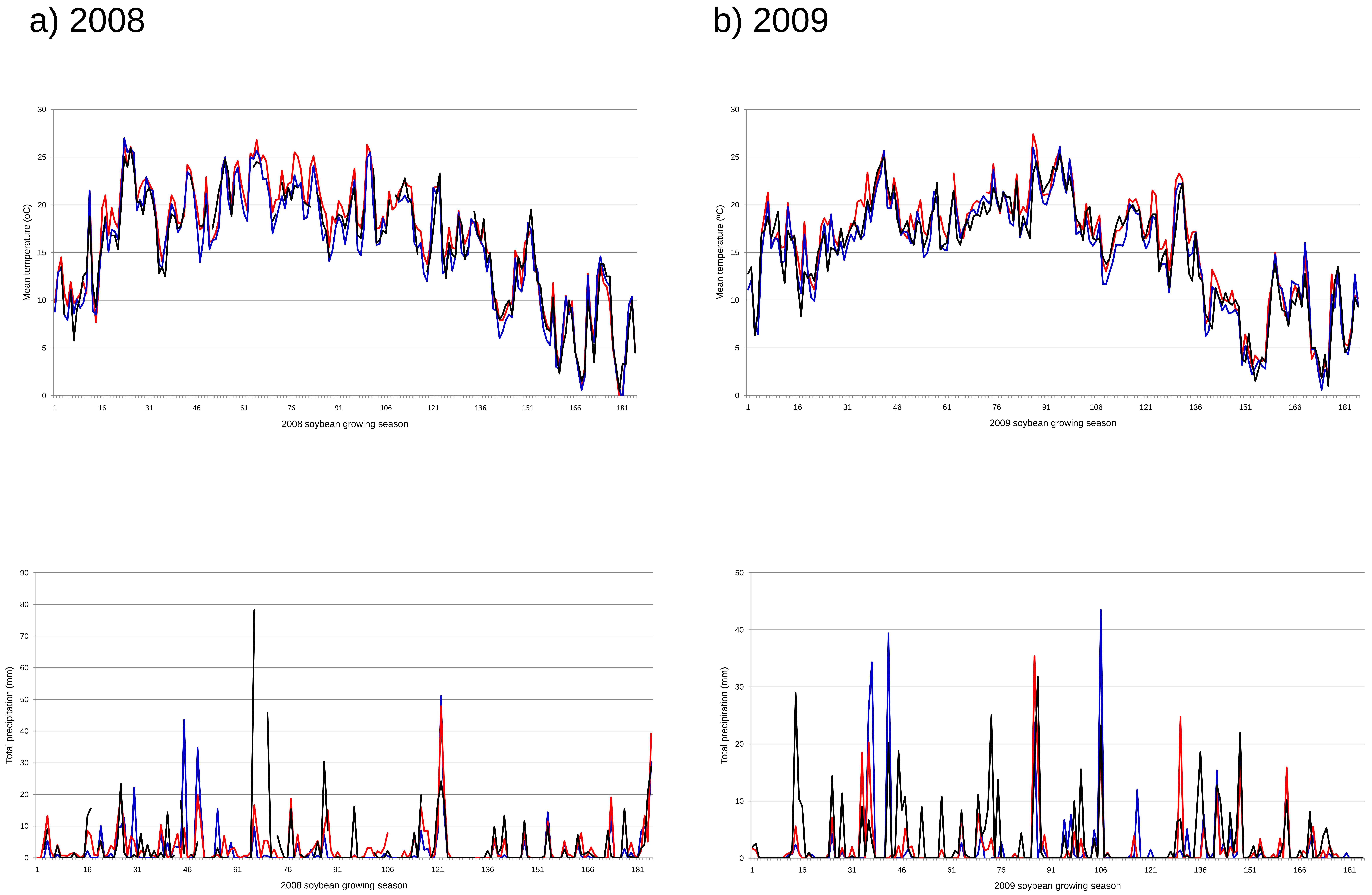

Fig. B in S1 File. Mean temperature (ºC) and total precipitation (mm) for soybean growing seasons in Harrow, Ridgetown and Woodstock (ON, Canada) in 2008 and 2009. a) 2008; b) 2009.

a) Plant (soybean) stems

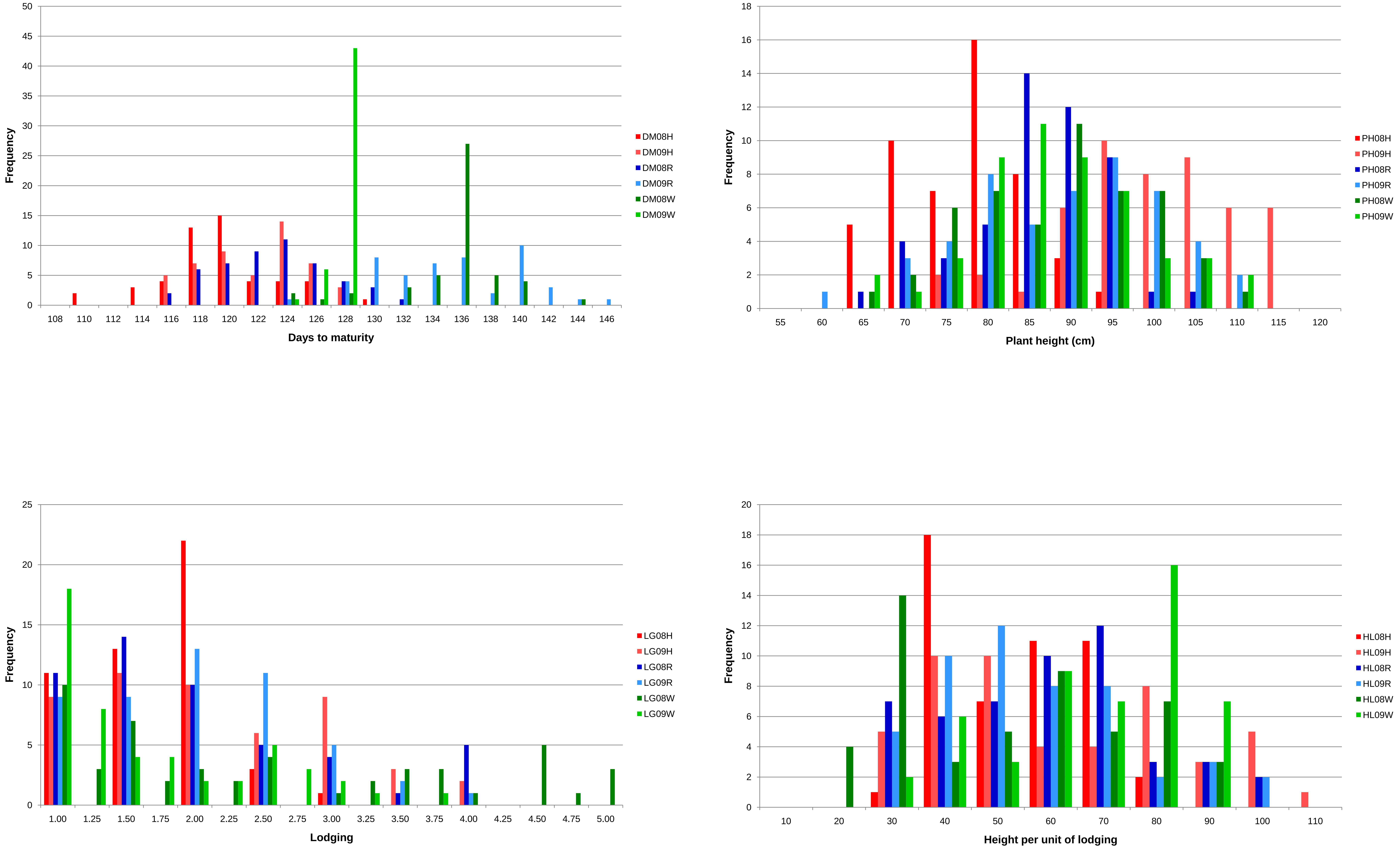

Fig. C in S1 File. Frequency distribution of traits (raw data) analyzed in parental genotypes (RG10 and OX948) and 50 selected RG10 x OX948 recombinant inbred lines (RILs). a) agronomic traits; b) fiber compositional traits; c) composite mechanical properties

b) Stem fibers

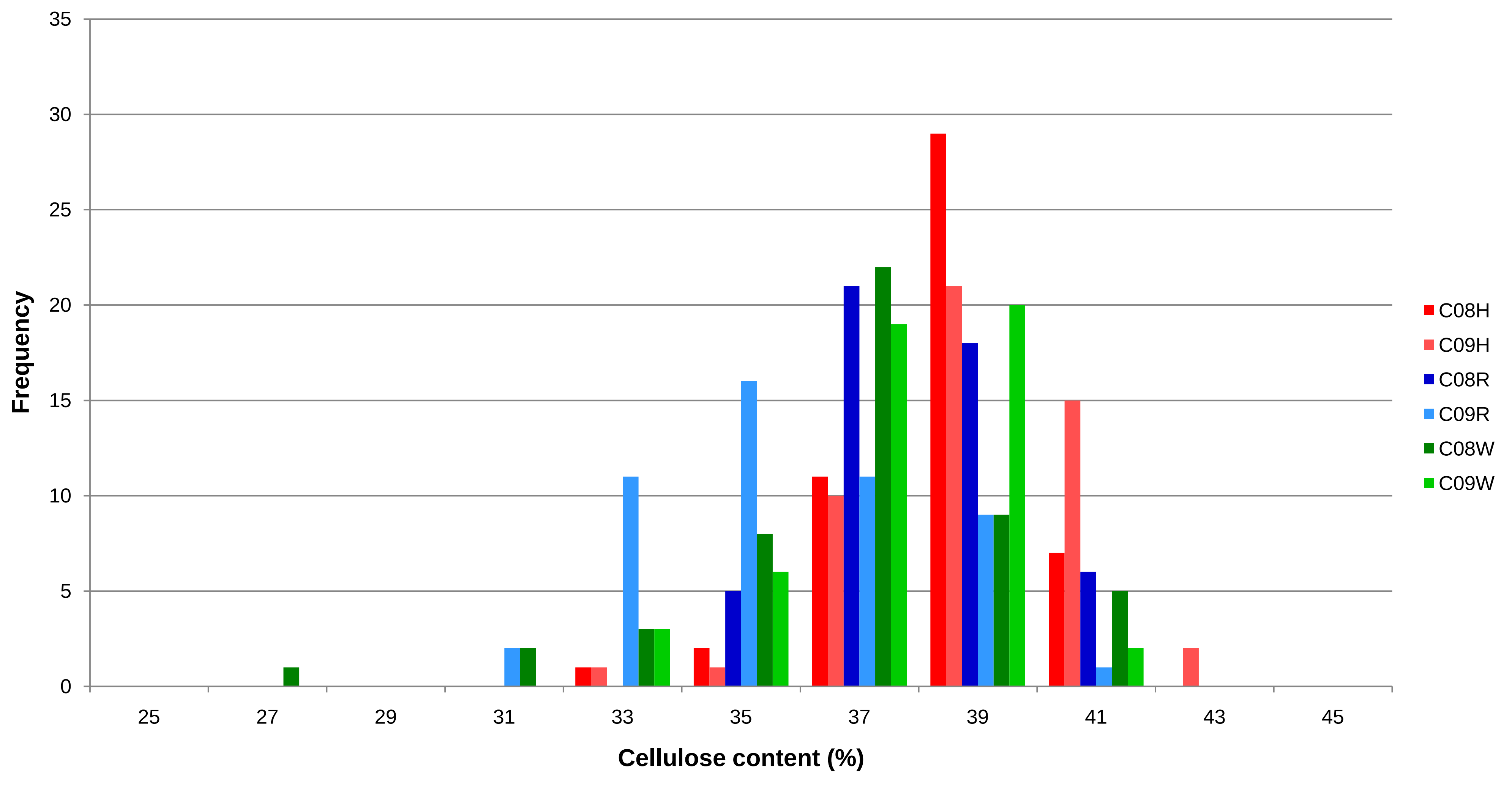

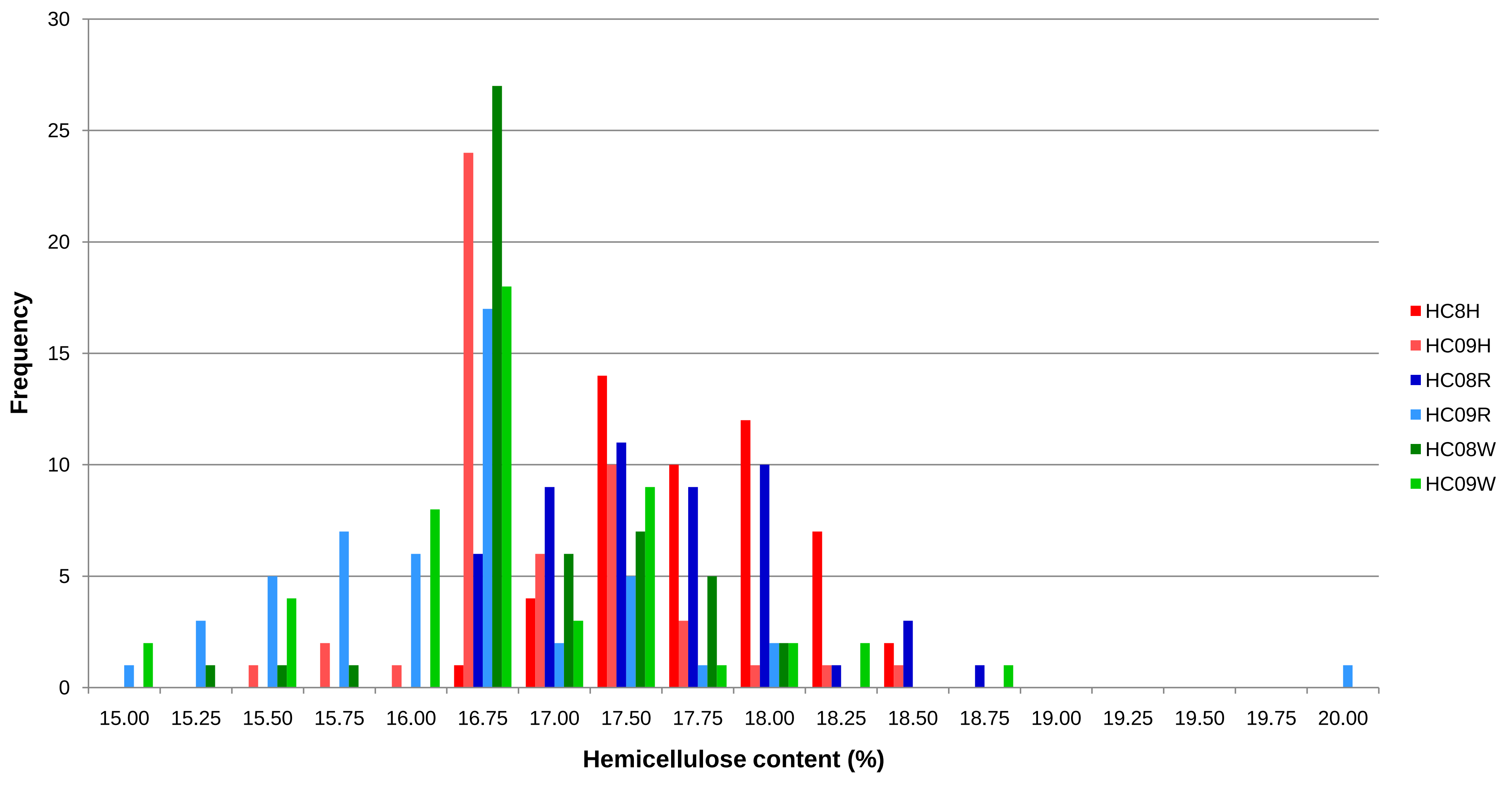

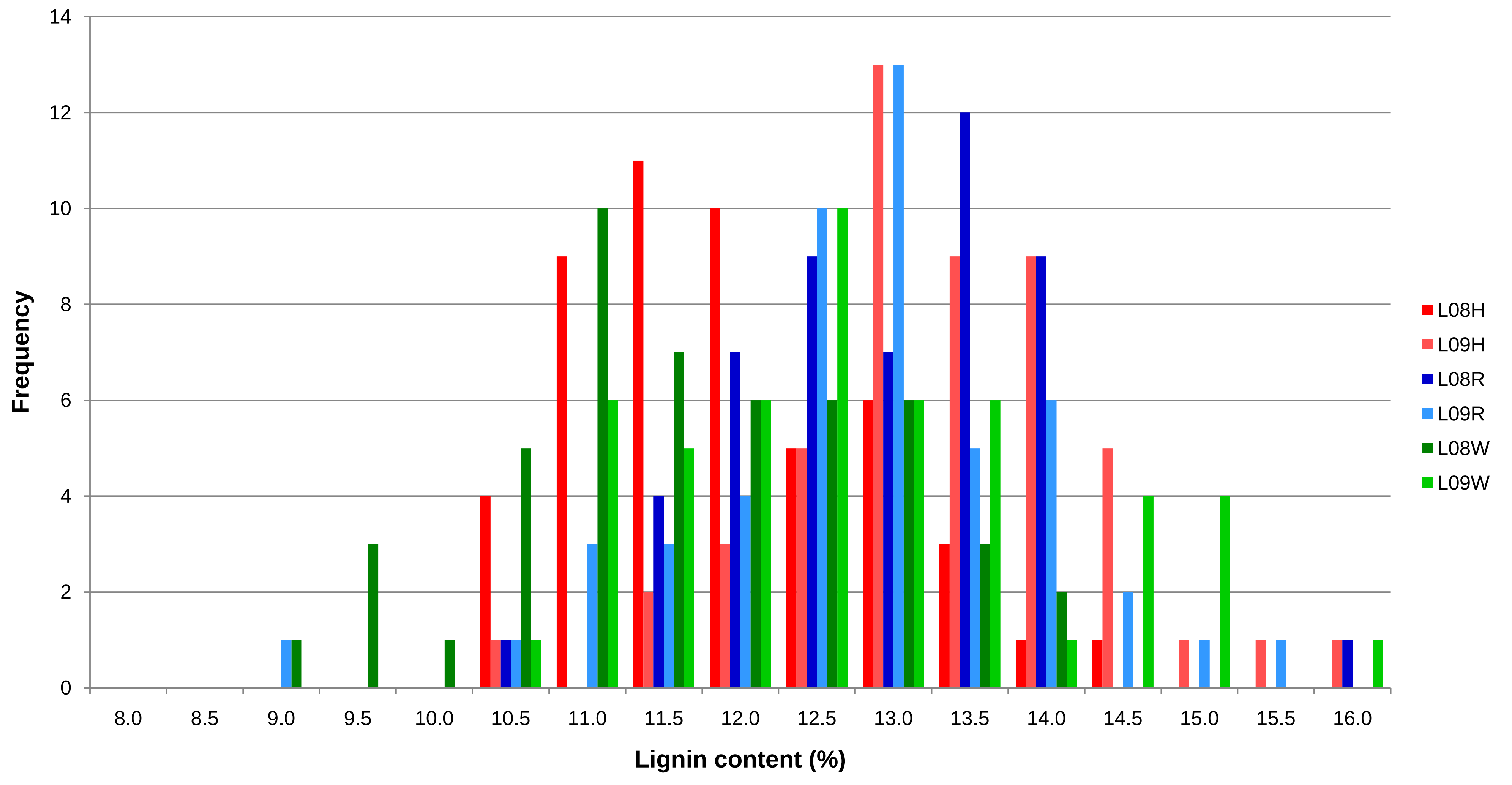

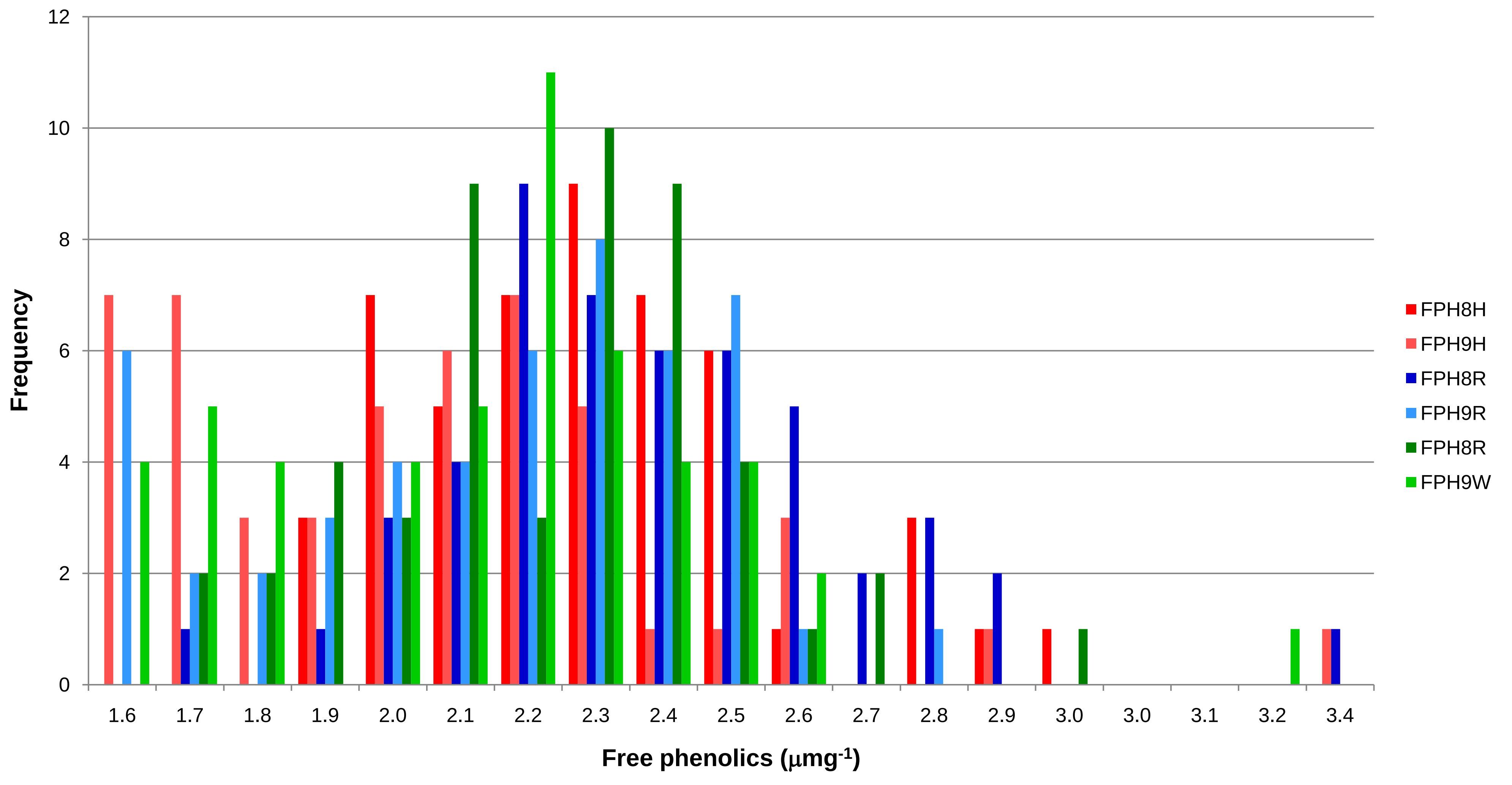

c) Composites

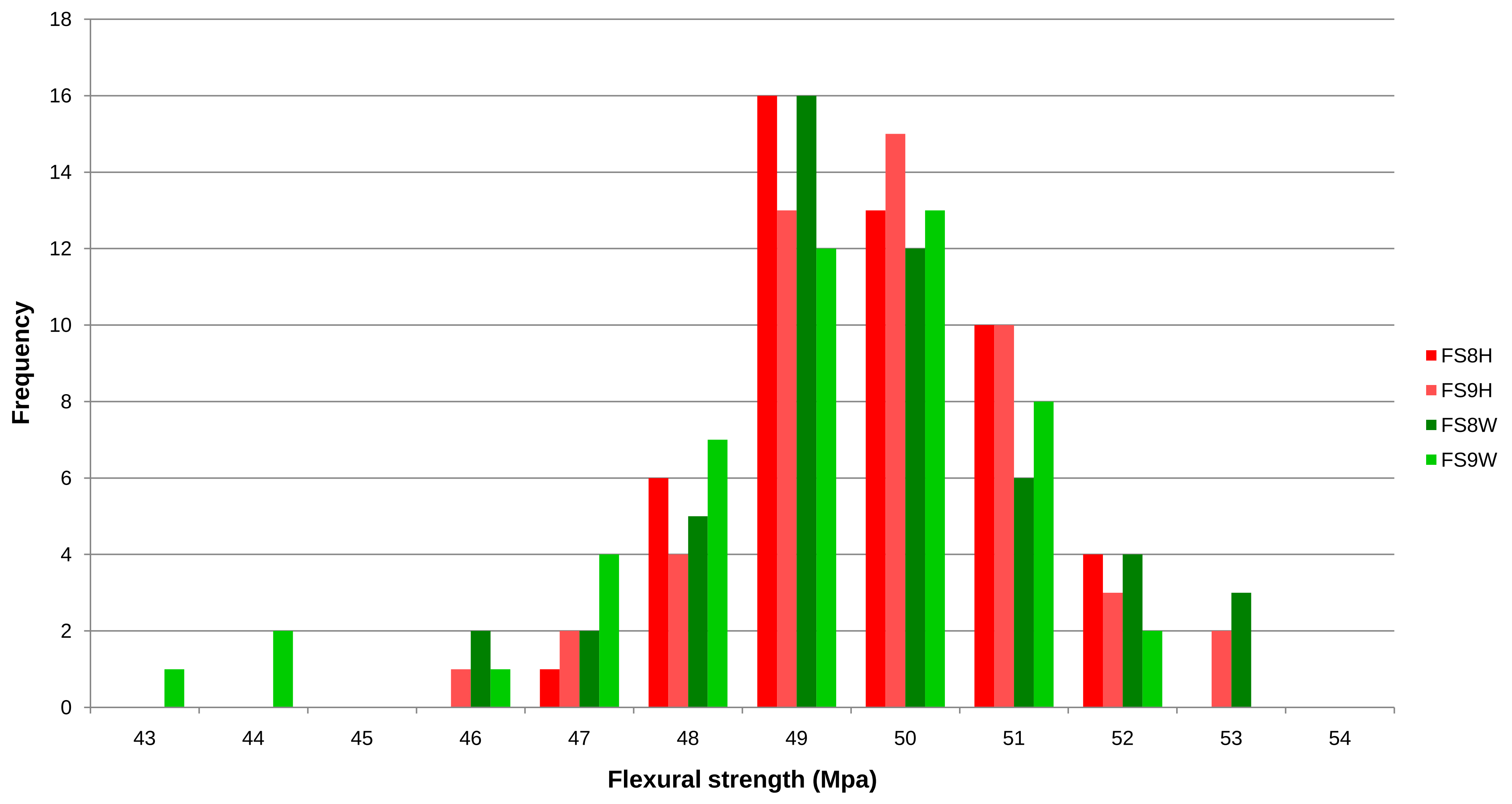

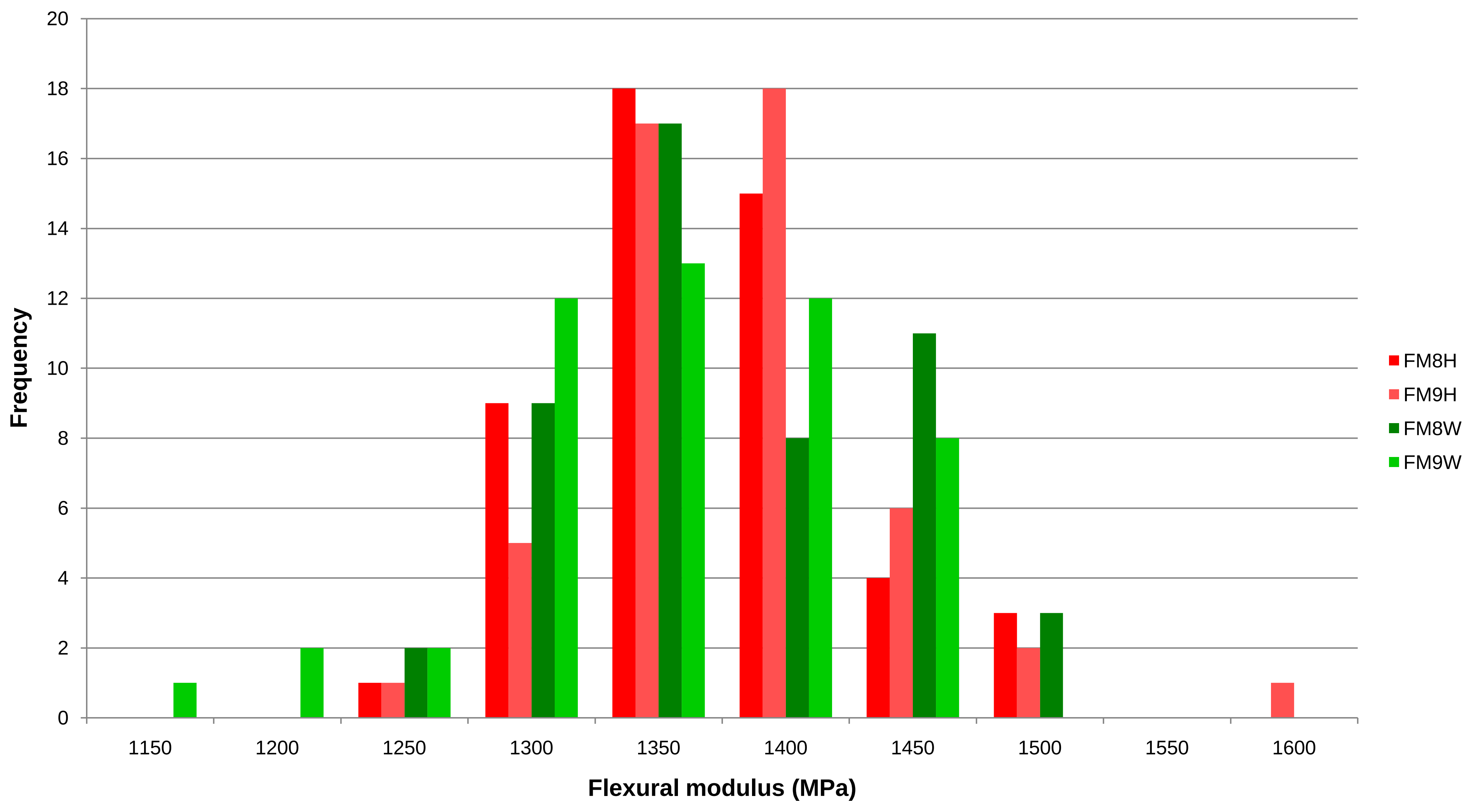

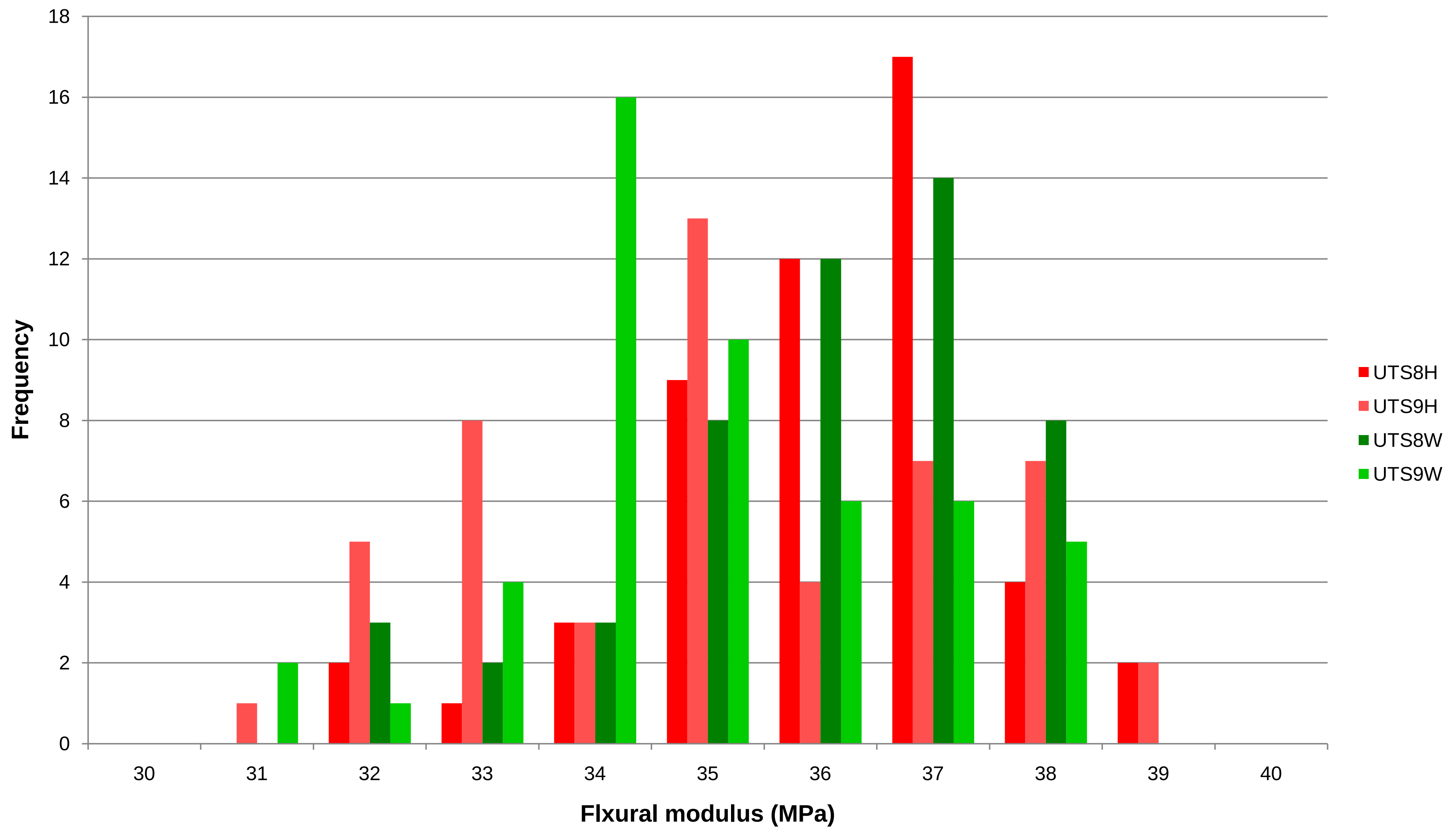

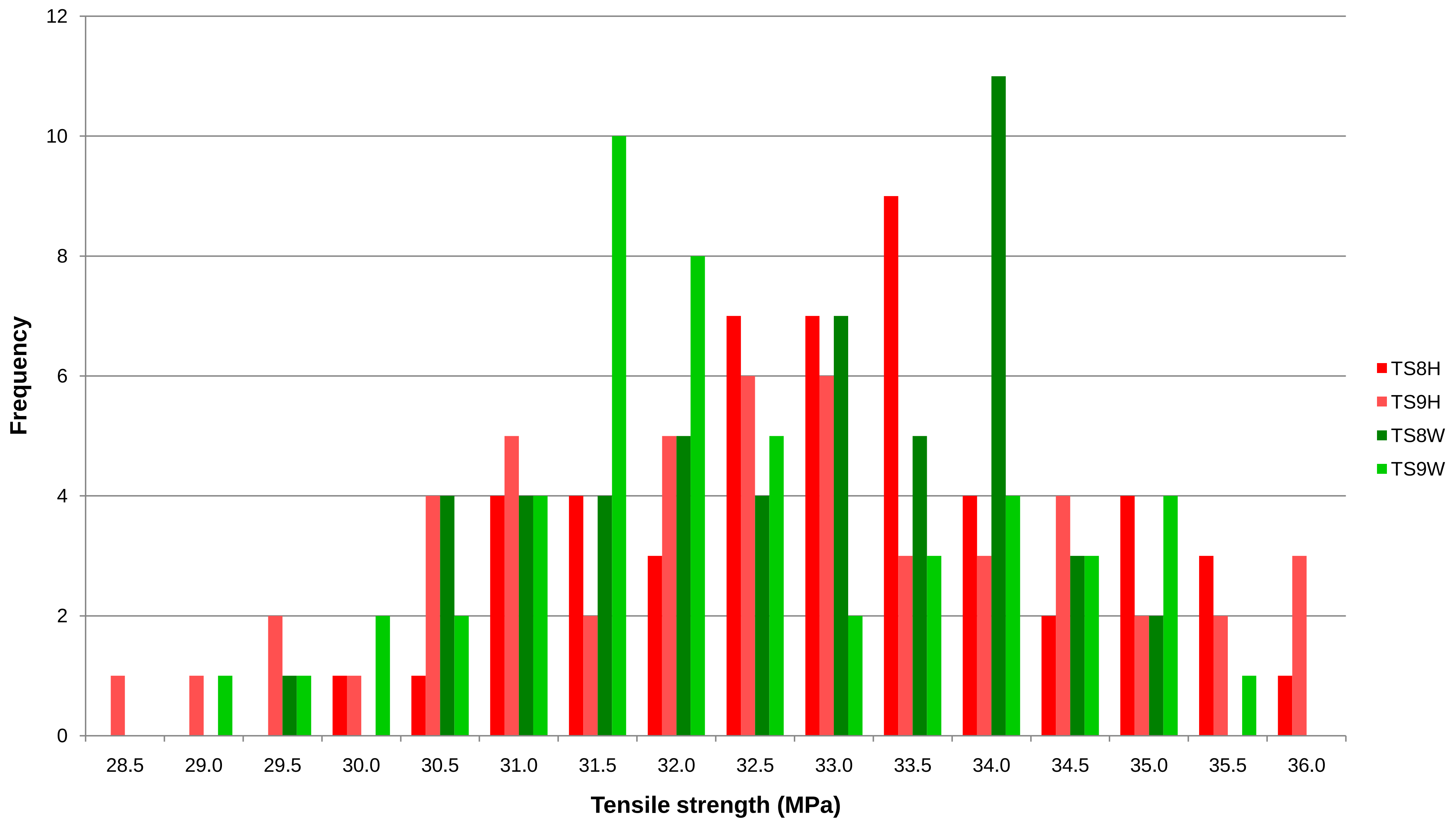

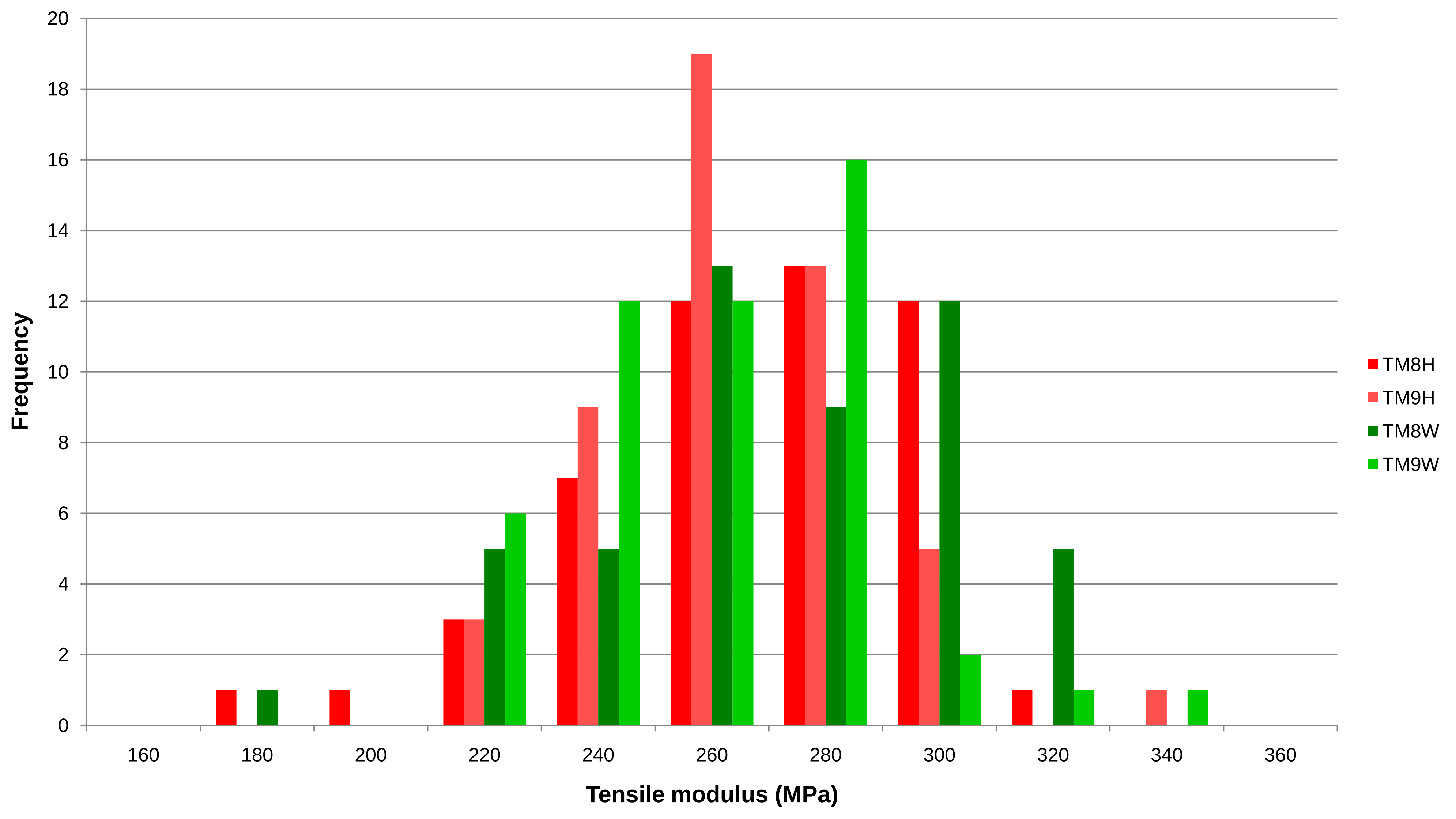
Frequency
Tensile modulus (MPa)
TM8H
TM9H
TM8W
TM9W

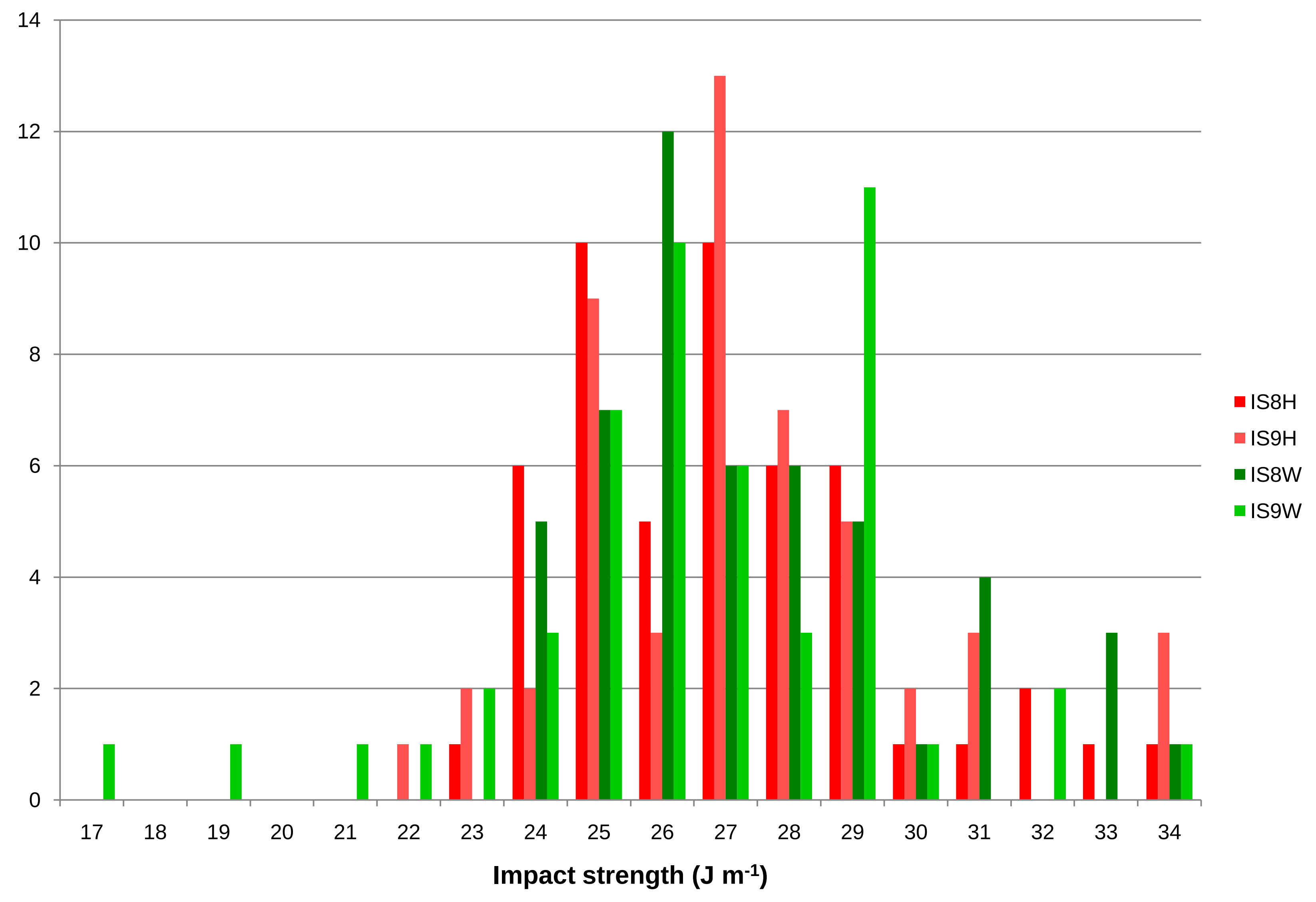
Impact strength (J m⁻¹)
IS8H
IS9H
IS8W
IS9W

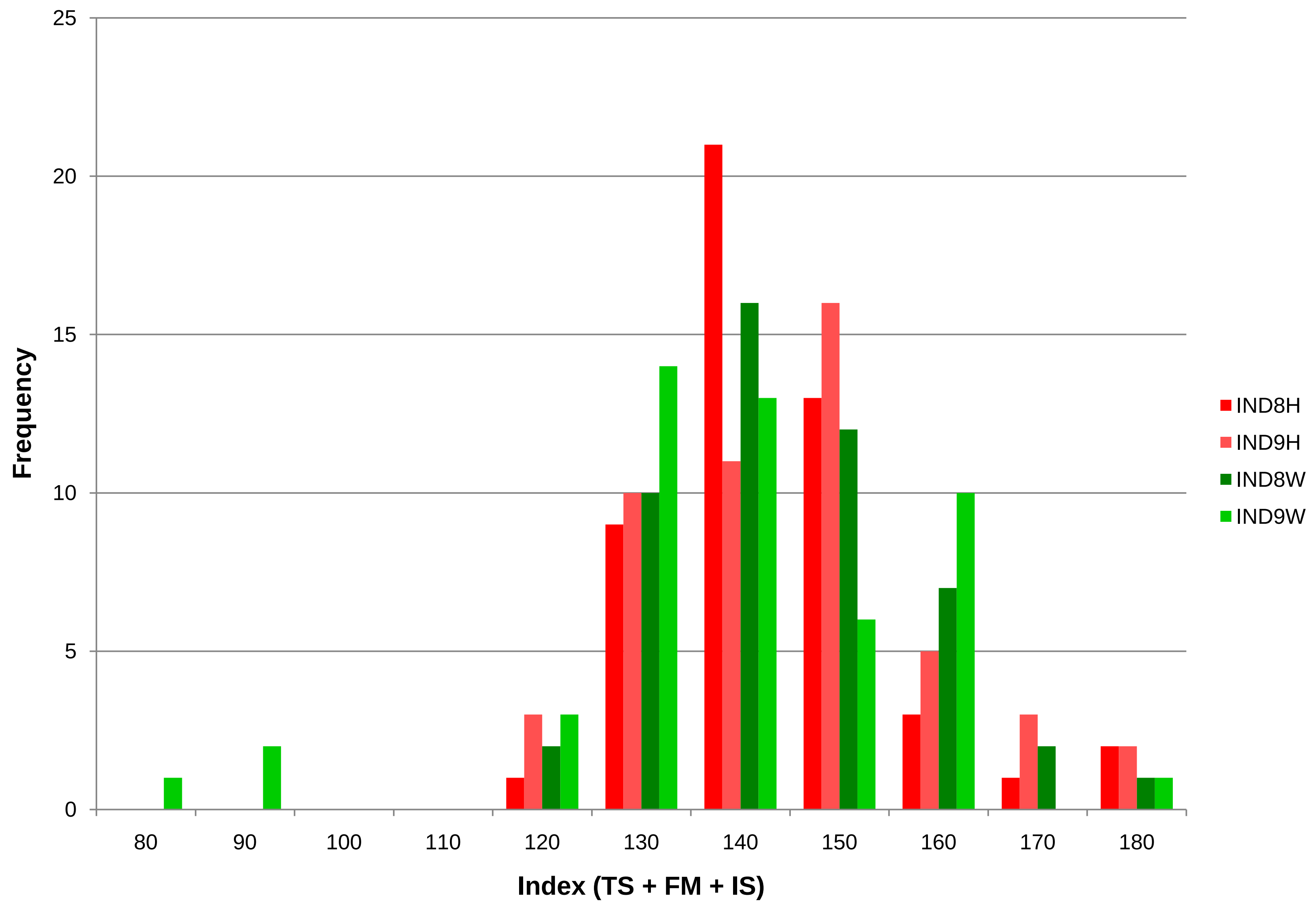
Frequency
Index (TS + FM + IS)
IND8H
IND9H
IND8W
IND9W

a) Plant (soybean) stems

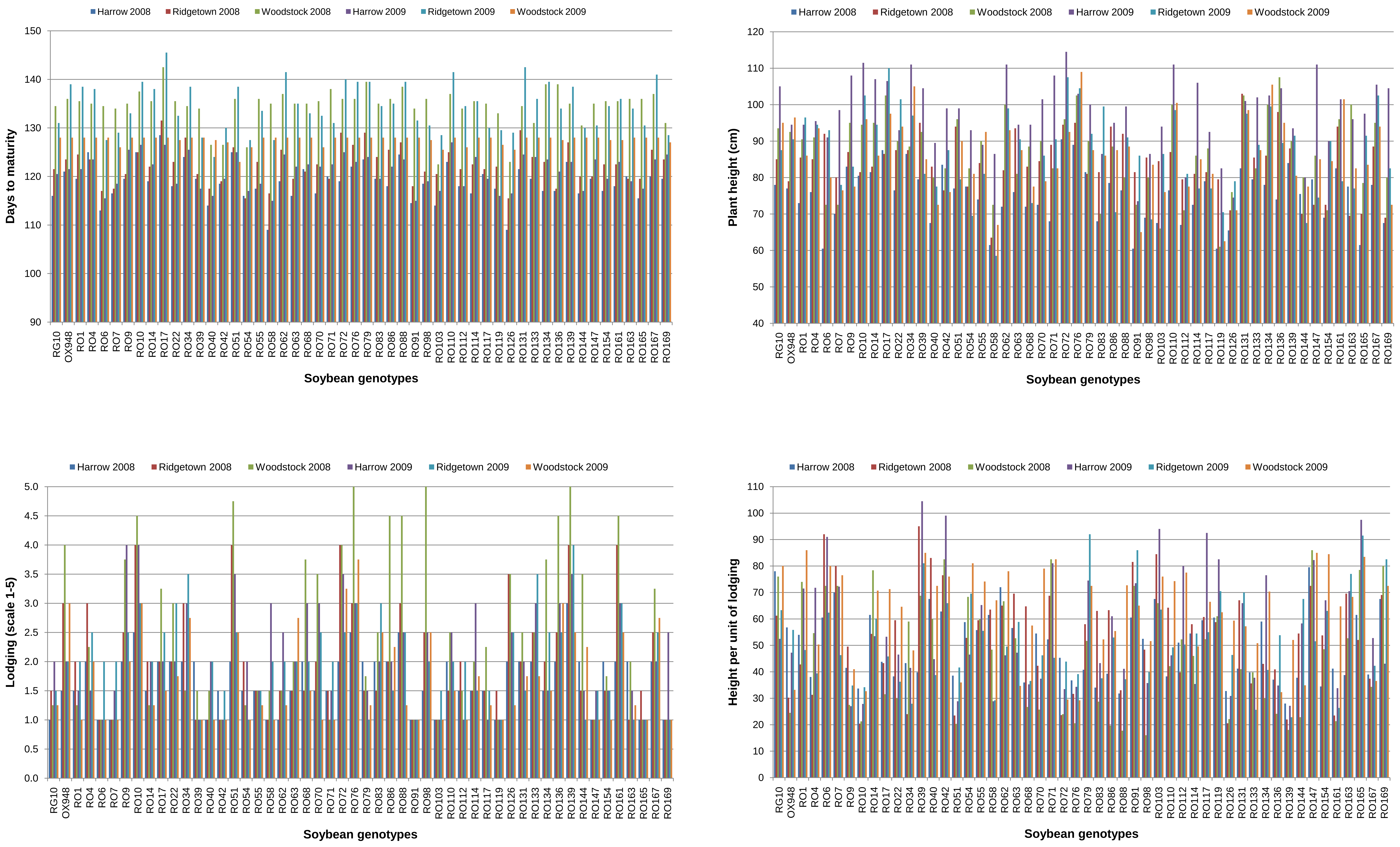

Fig. D in S1 File. Distribution of 15 traits in parental genotypes (RG10 and OX948) and 50 selected RG10 x OX948 recombinant inbred lines (RILs). a) agronomic traits; b) fiber compositional traits; c) composite mechanical properties

b) Stem fibers

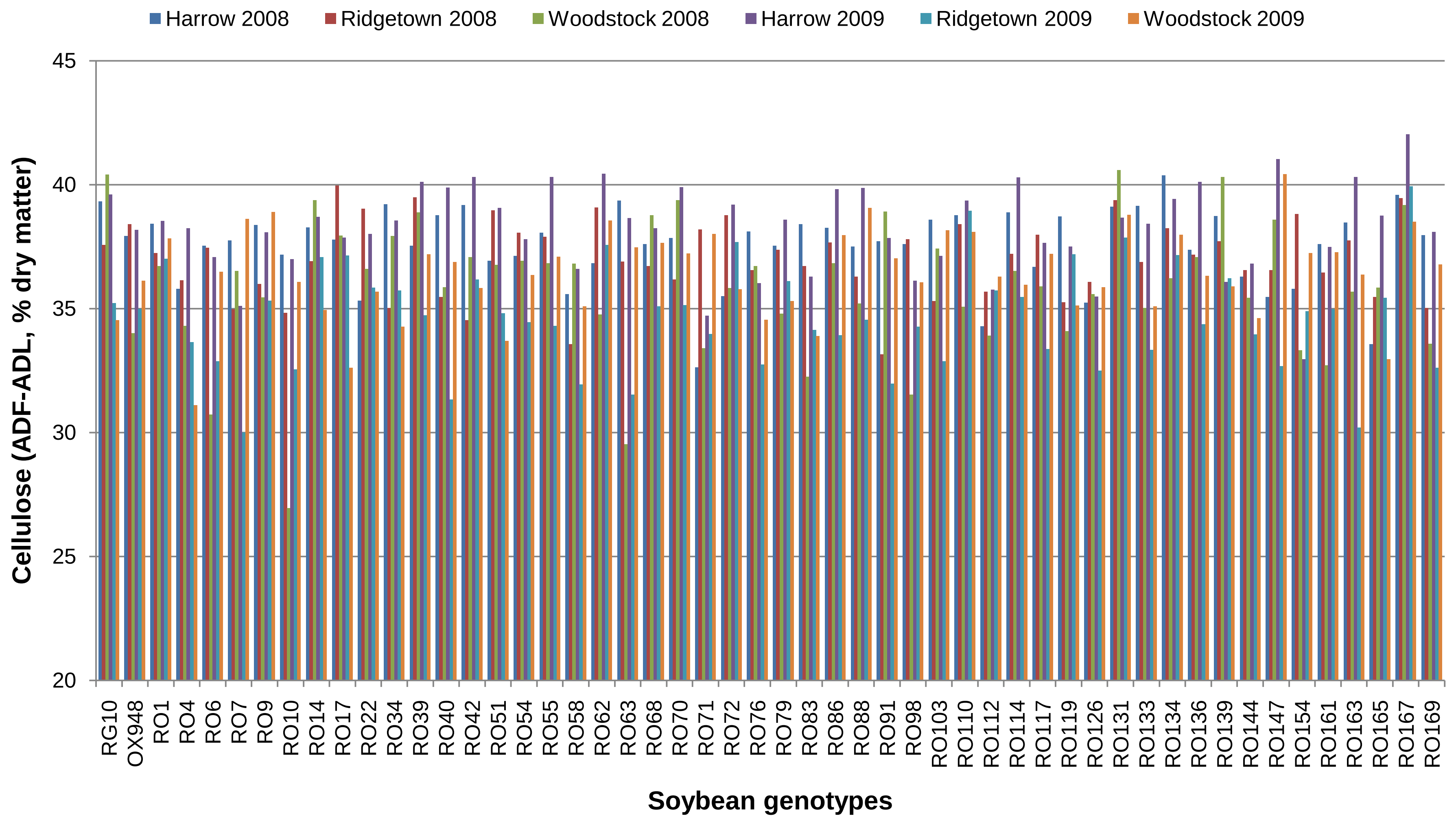

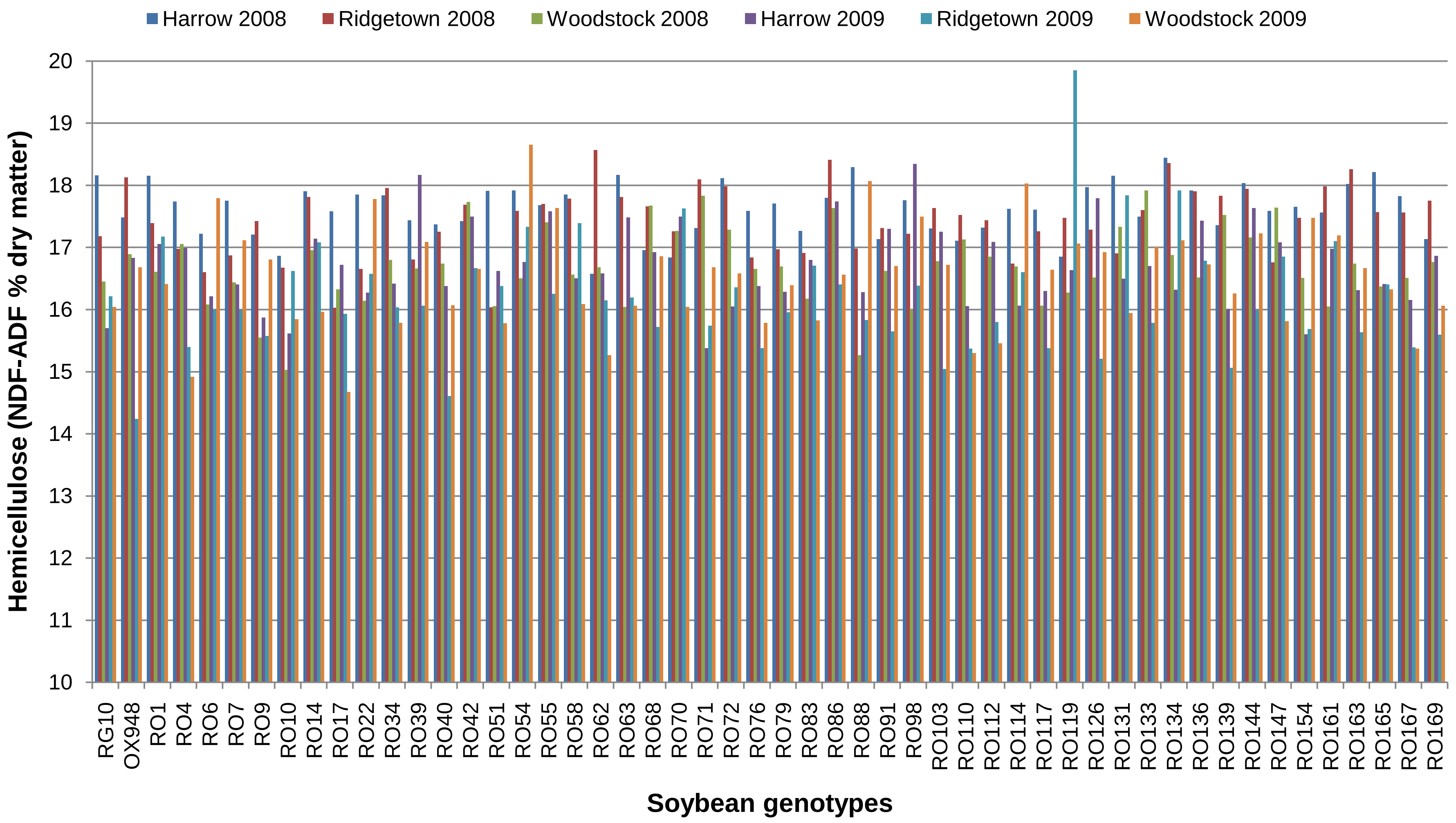

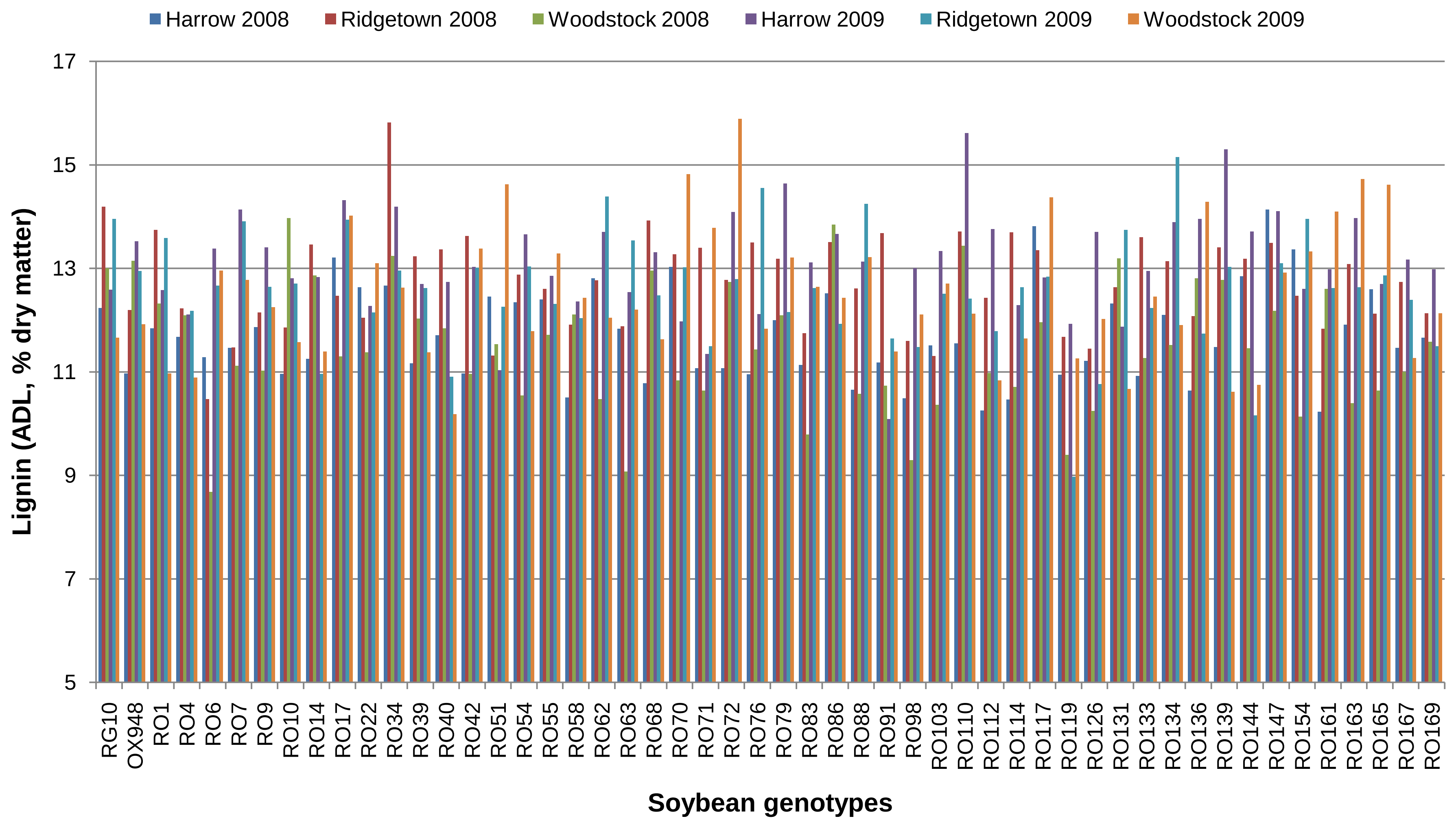

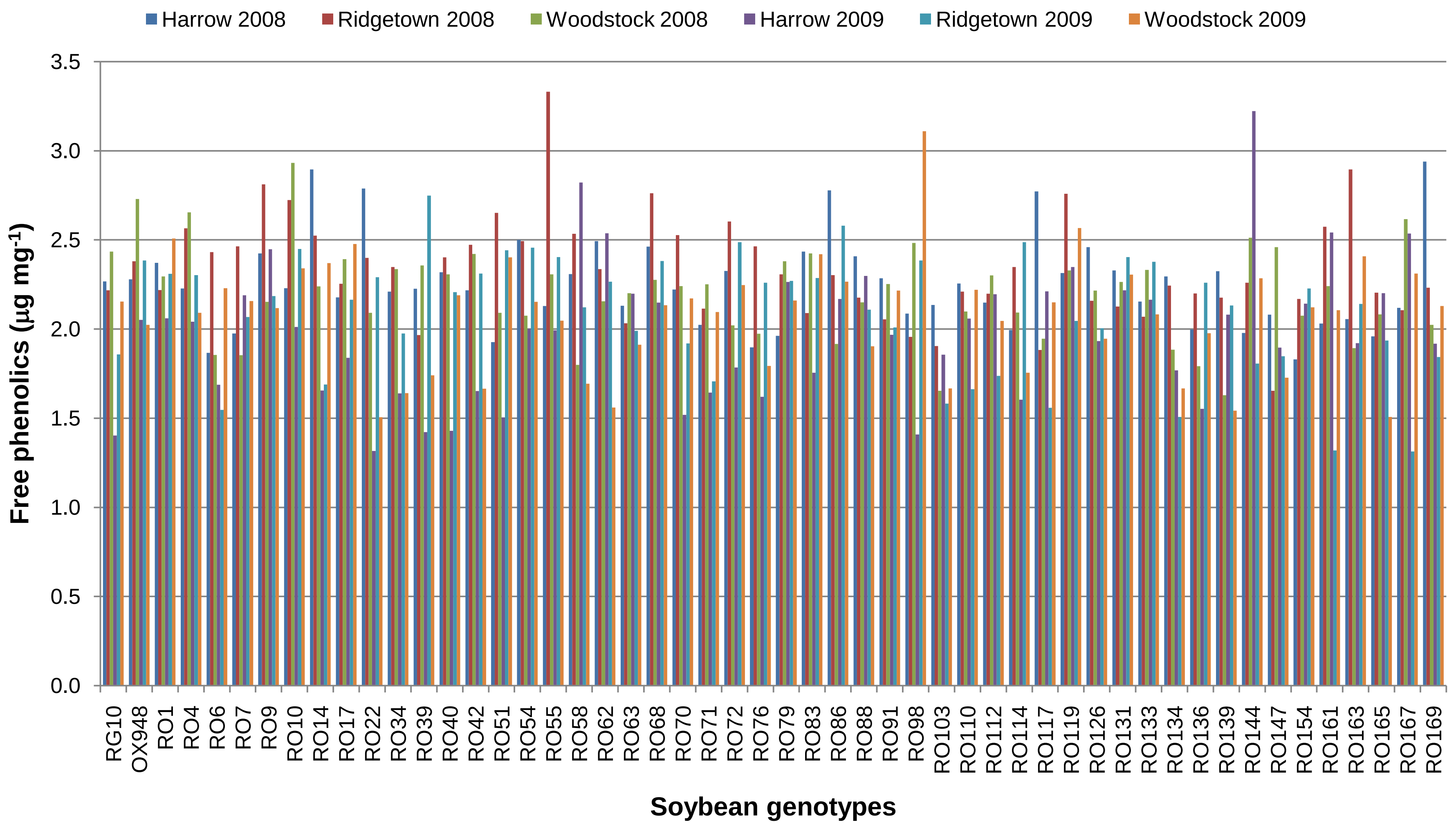

c) Composites

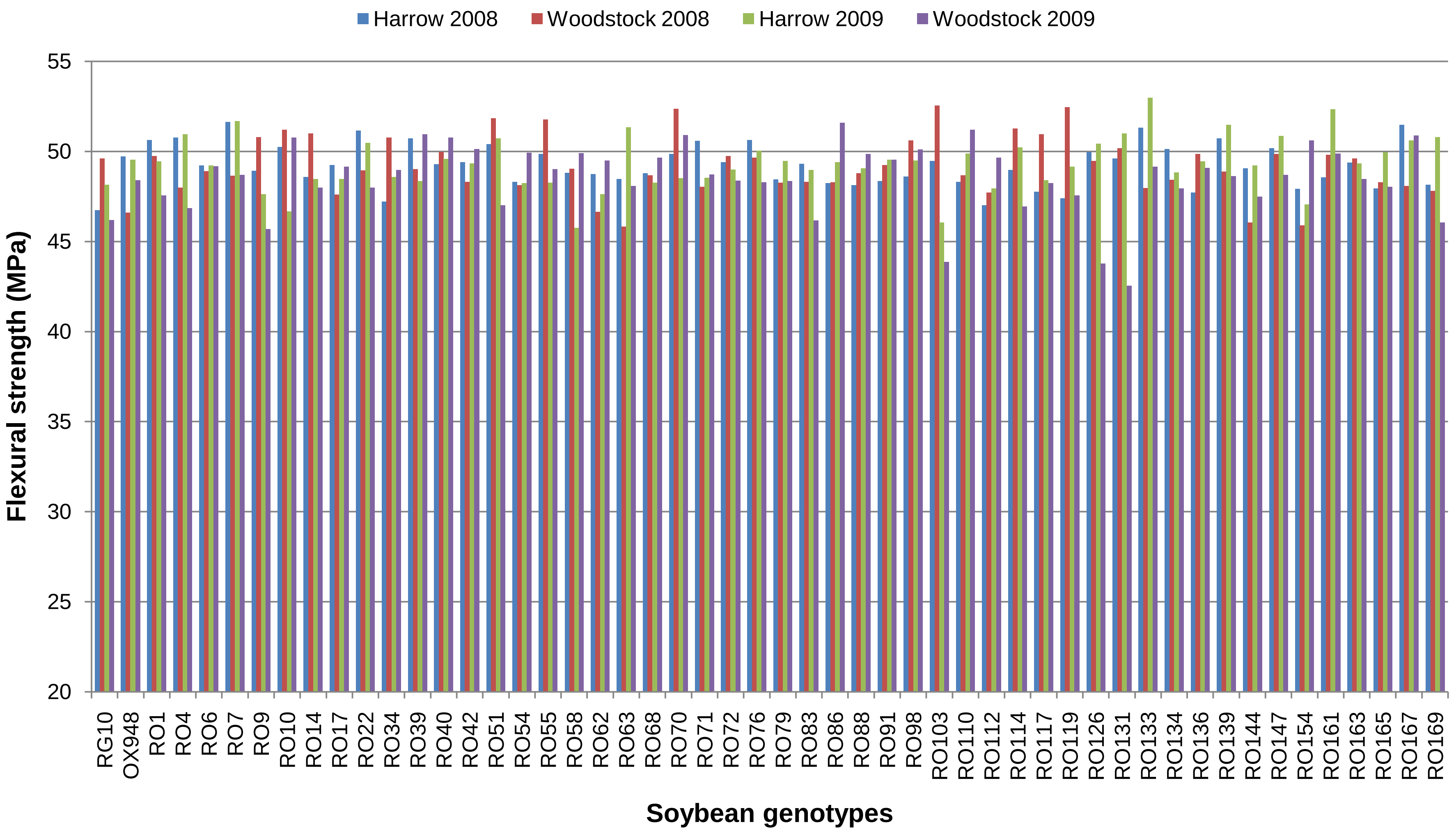

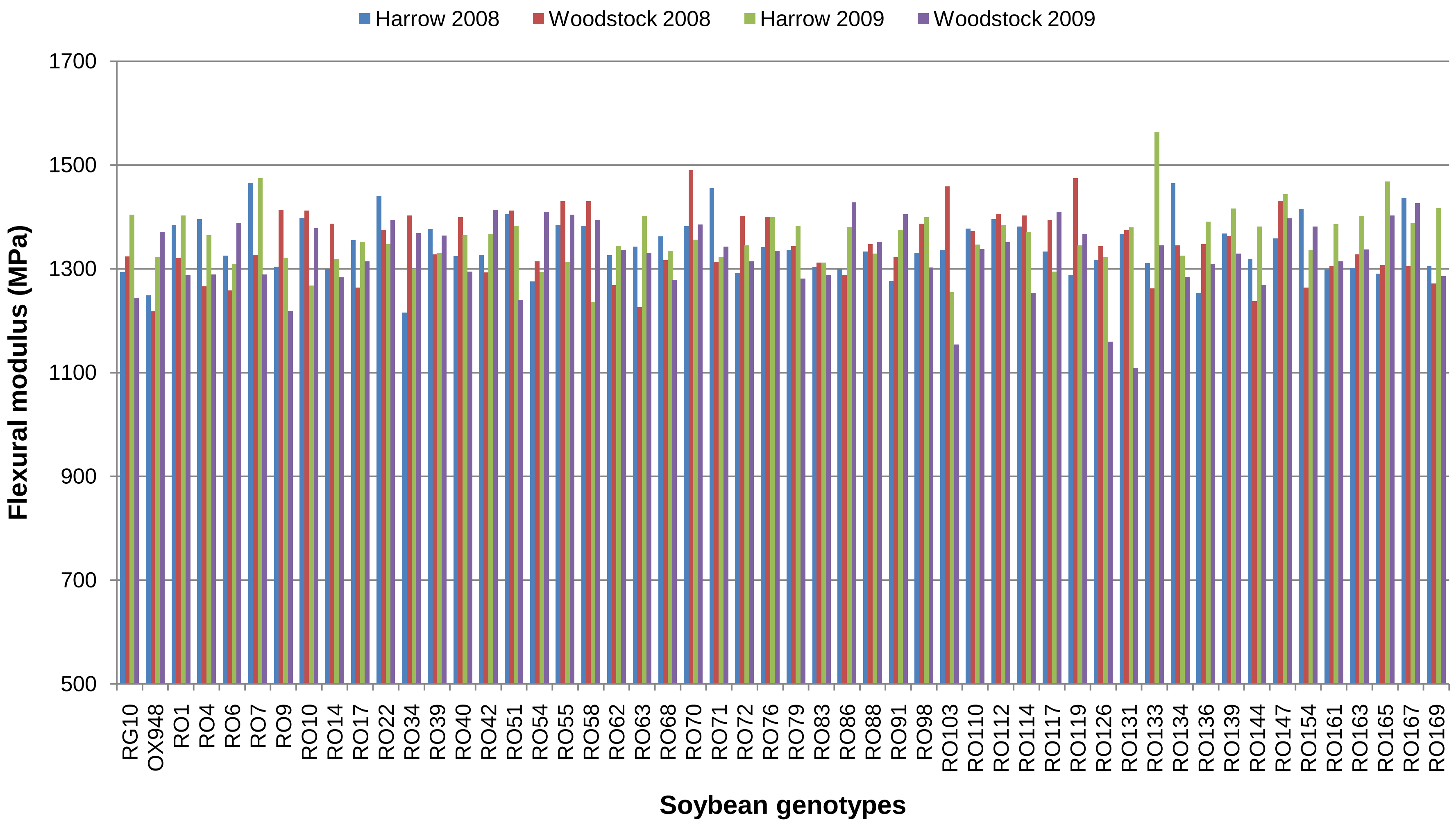

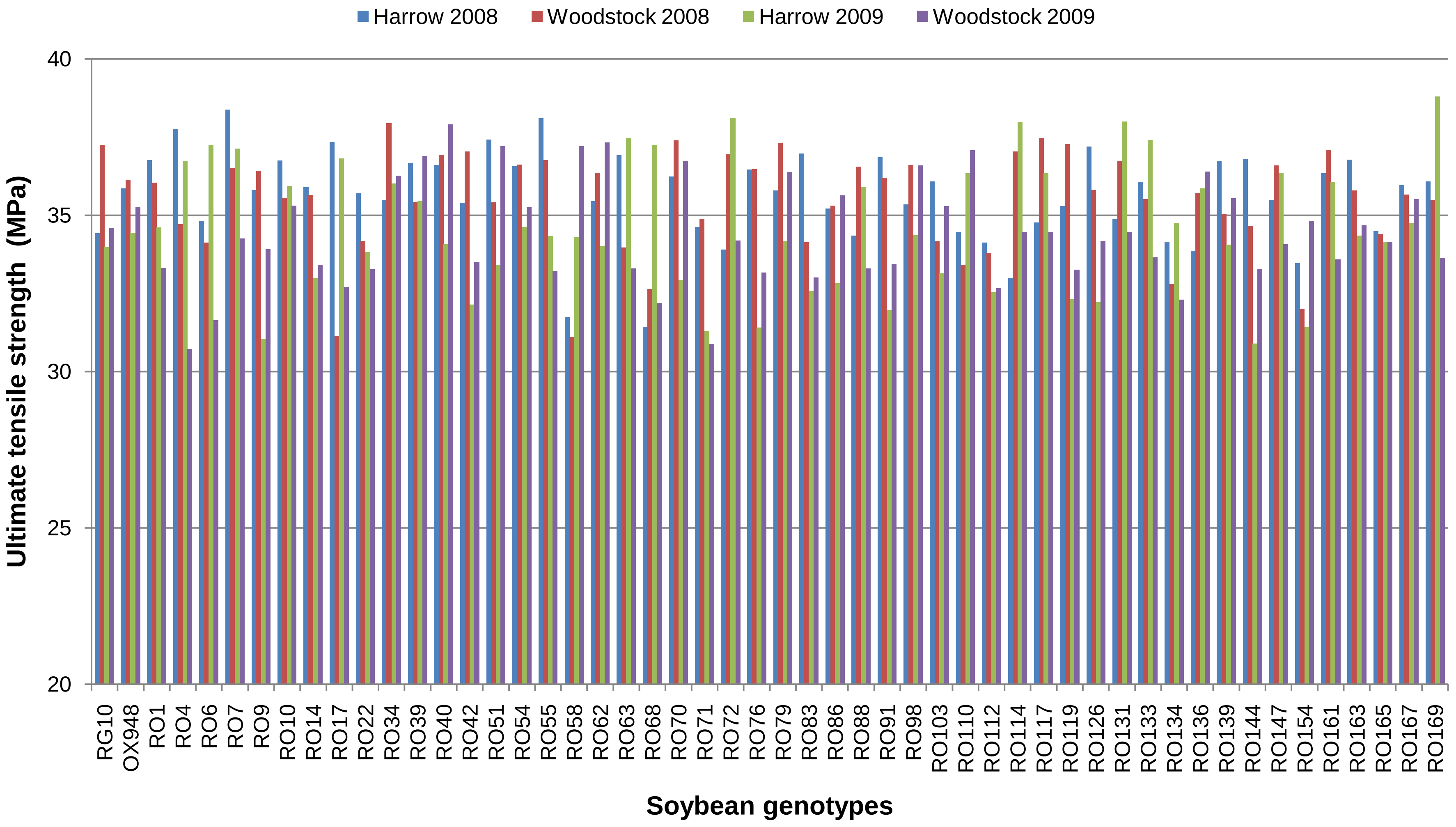

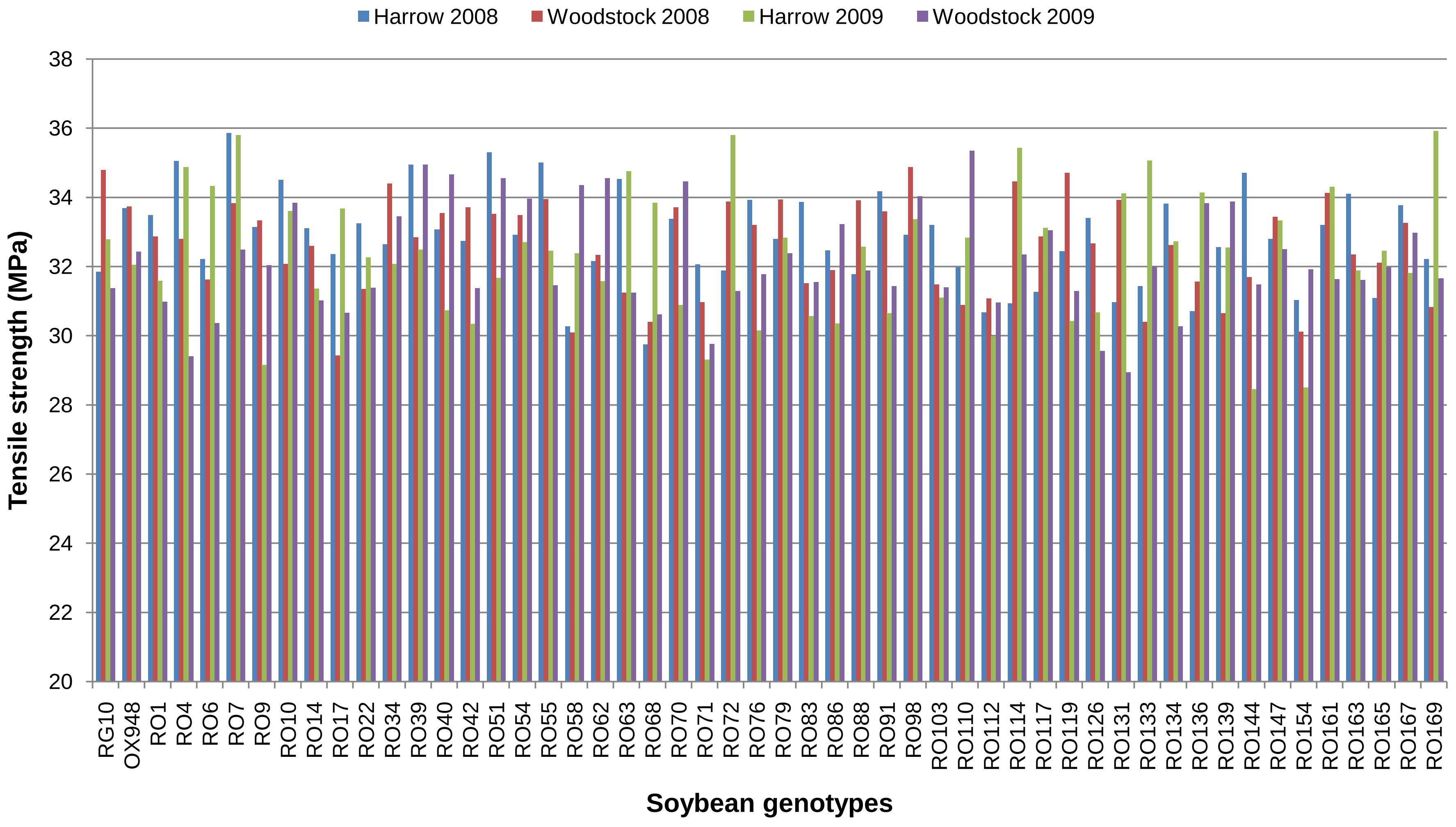

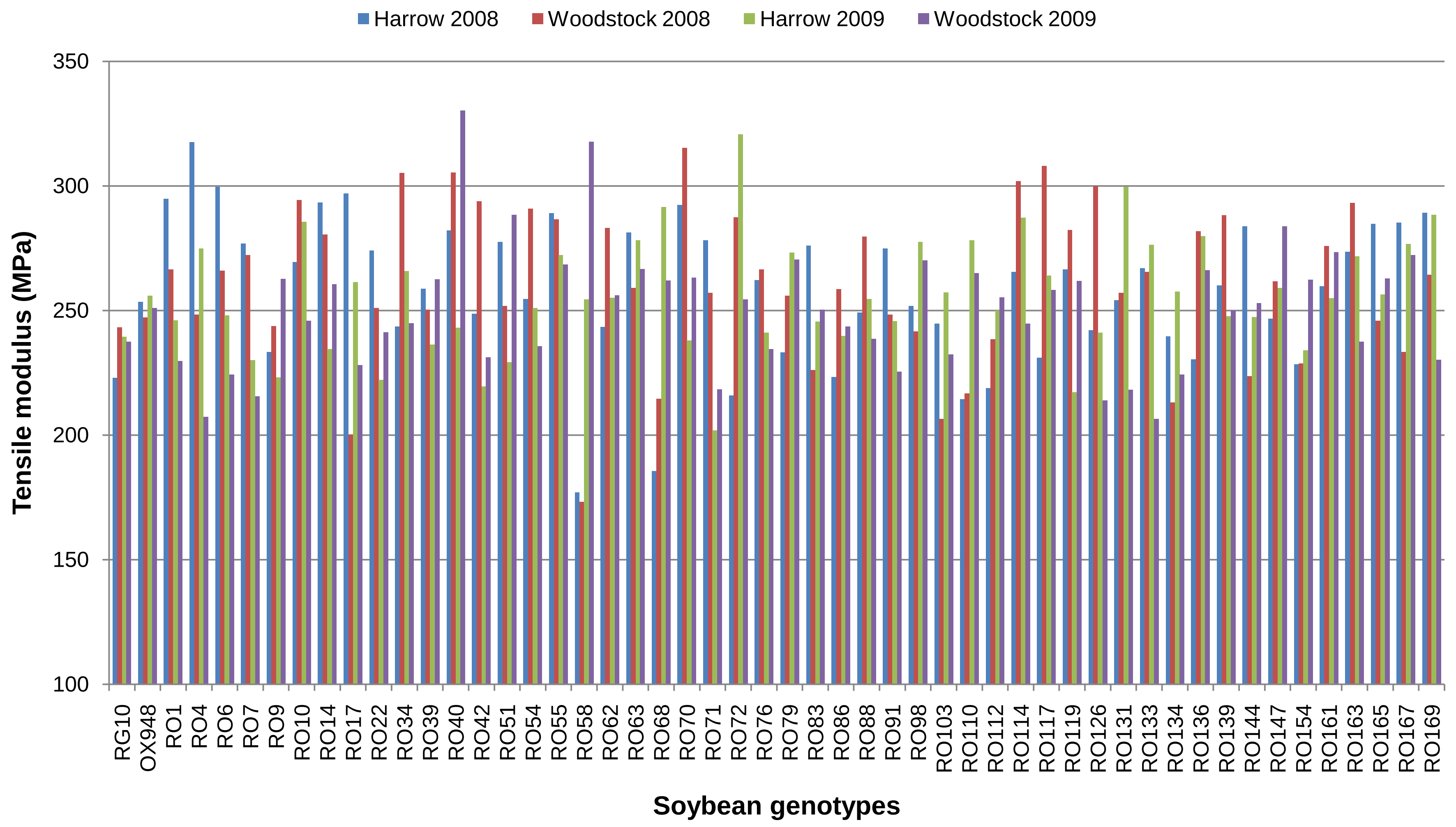
Harrow 2008
Woodstock 2008
Harrow 2009
Woodstock 2009
Tensile modulus (MPa)
Soybean genotypes

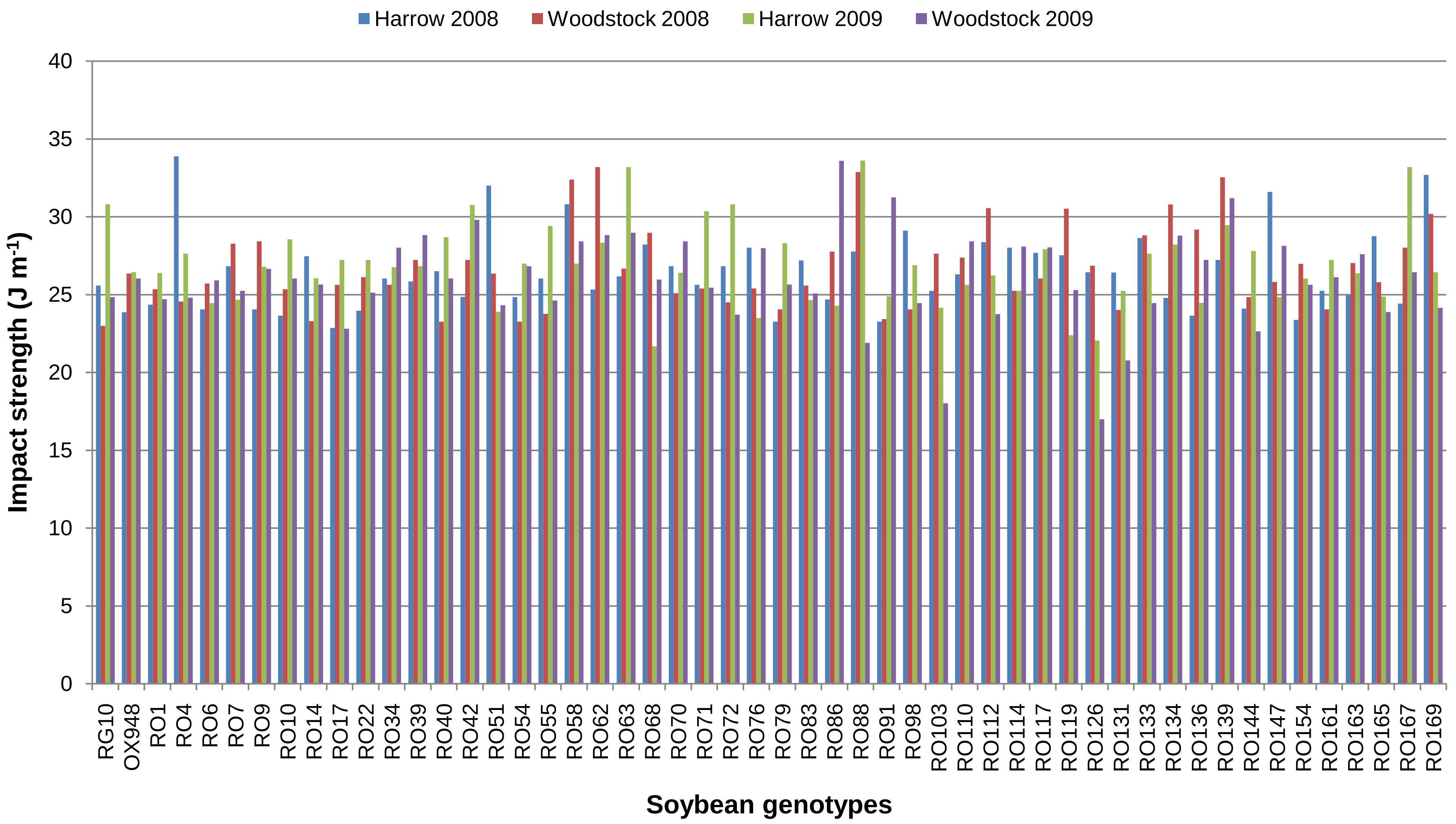
Harrow 2008
Woodstock 2008
Harrow 2009
Woodstock 2009
Impact strength (J m-1)
Soybean genotypes

a)

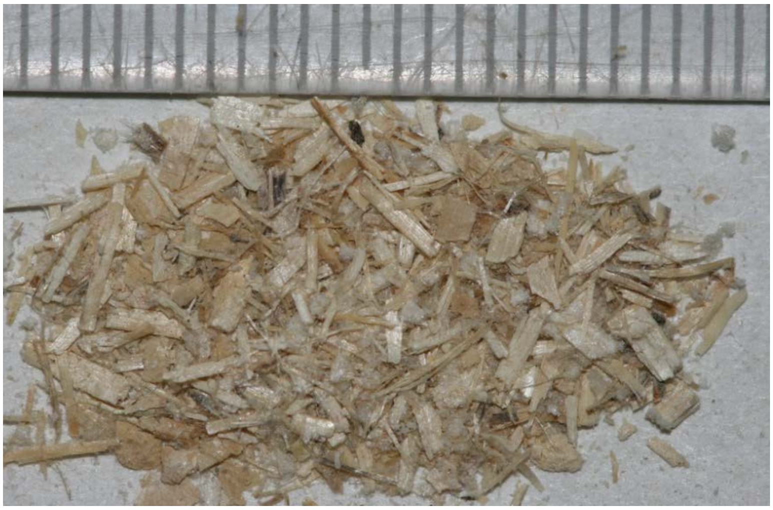

b)

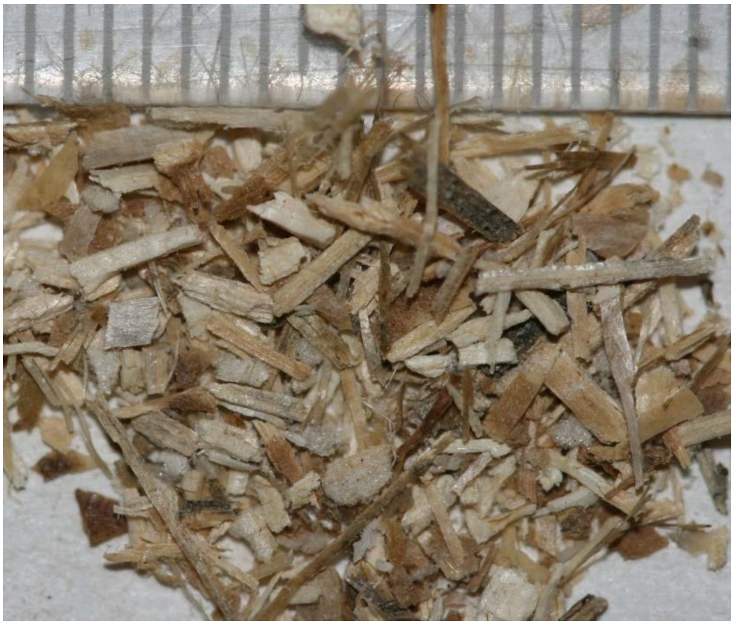

Fig. E in S1 File. Ground dry stem fibers from selected soybean lines. a) light brown color; b) deep brown color.

a) QTL for agronomic and fiber compositional traits in six environments

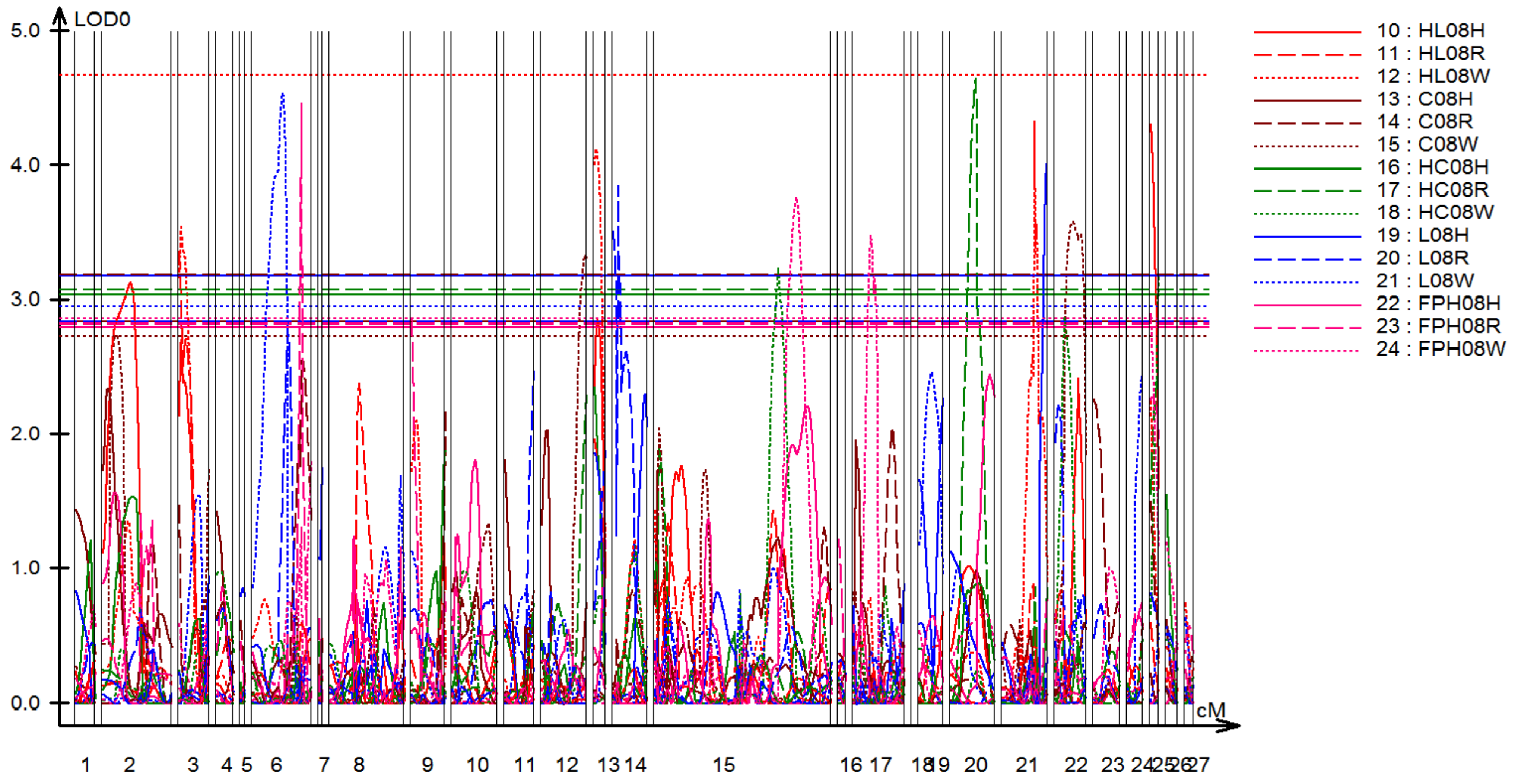

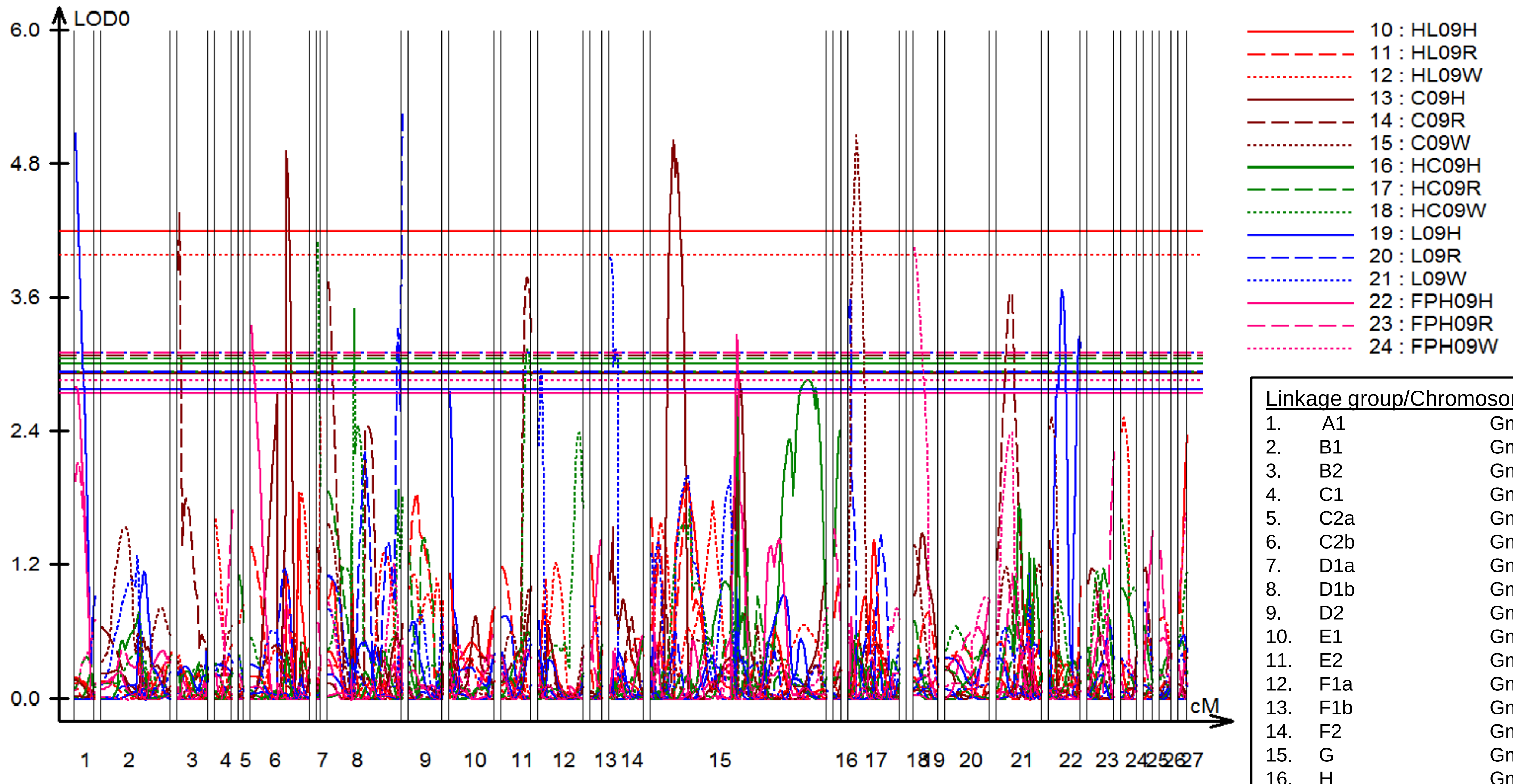

Linkage group/Chromosome:

1.	A1	Gm08
2.	B1	Gm11
3.	B2	Gm14
4.	C1	Gm04
5.	C2a	Gm06
6.	C2b	Gm06
7.	D1a	Gm01
8.	D1b	Gm02
9.	D2	Gm17
10.	E1	Gm15
11.	E2	Gm15
12.	F1a	Gm13
13.	F1b	Gm13
14.	F2	Gm13
15.	G	Gm18
16.	H	Gm12
17.	I	Gm20
18.	J1	Gm16
19.	J2	Gm16
20.	K	Gm09
21.	L	Gm19
22.	M	Gm07
23.	N	Gm03
24.	O1	Gm10
25.	O2	Gm10
26.	O3	Gm10
27.	X	-

Fig. F in S1 File. Distribution of quantitative trait loci (QTL) LOD scores. a) QTL for agronomic and fiber compositional traits in six environments; b) QTL for fiber mechanical performance in four environments.

b) QTL for fiber mechanical performance in four environments

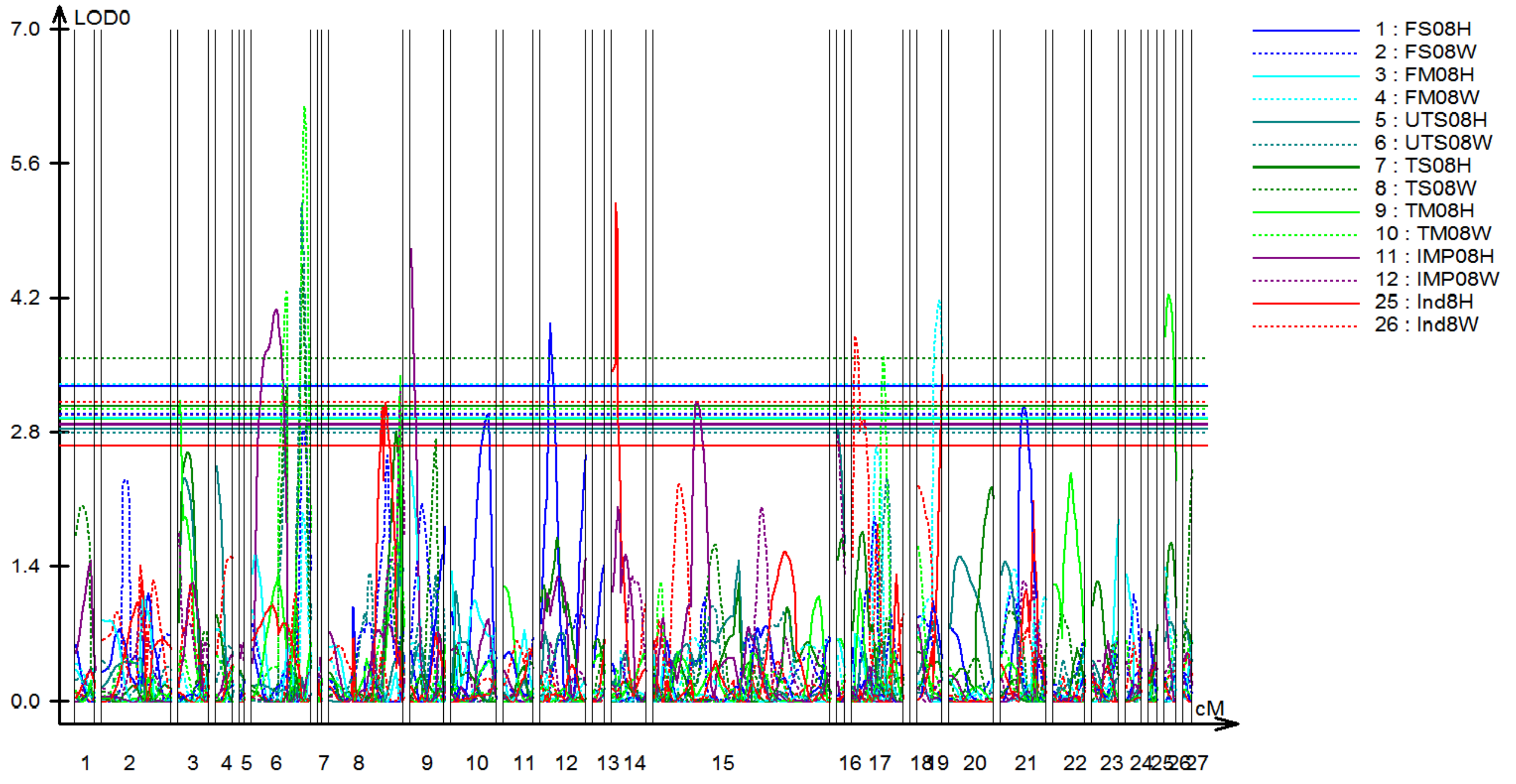

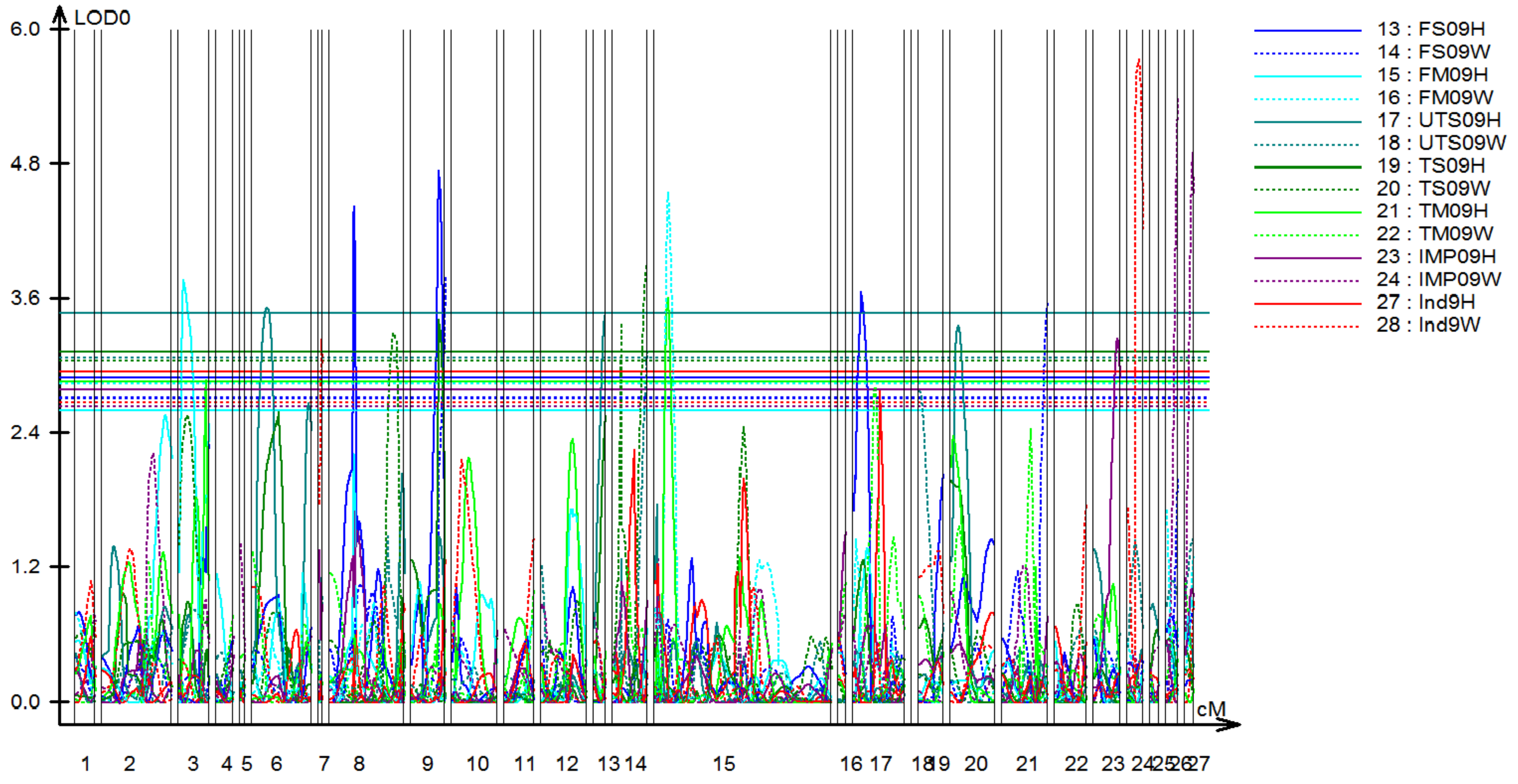

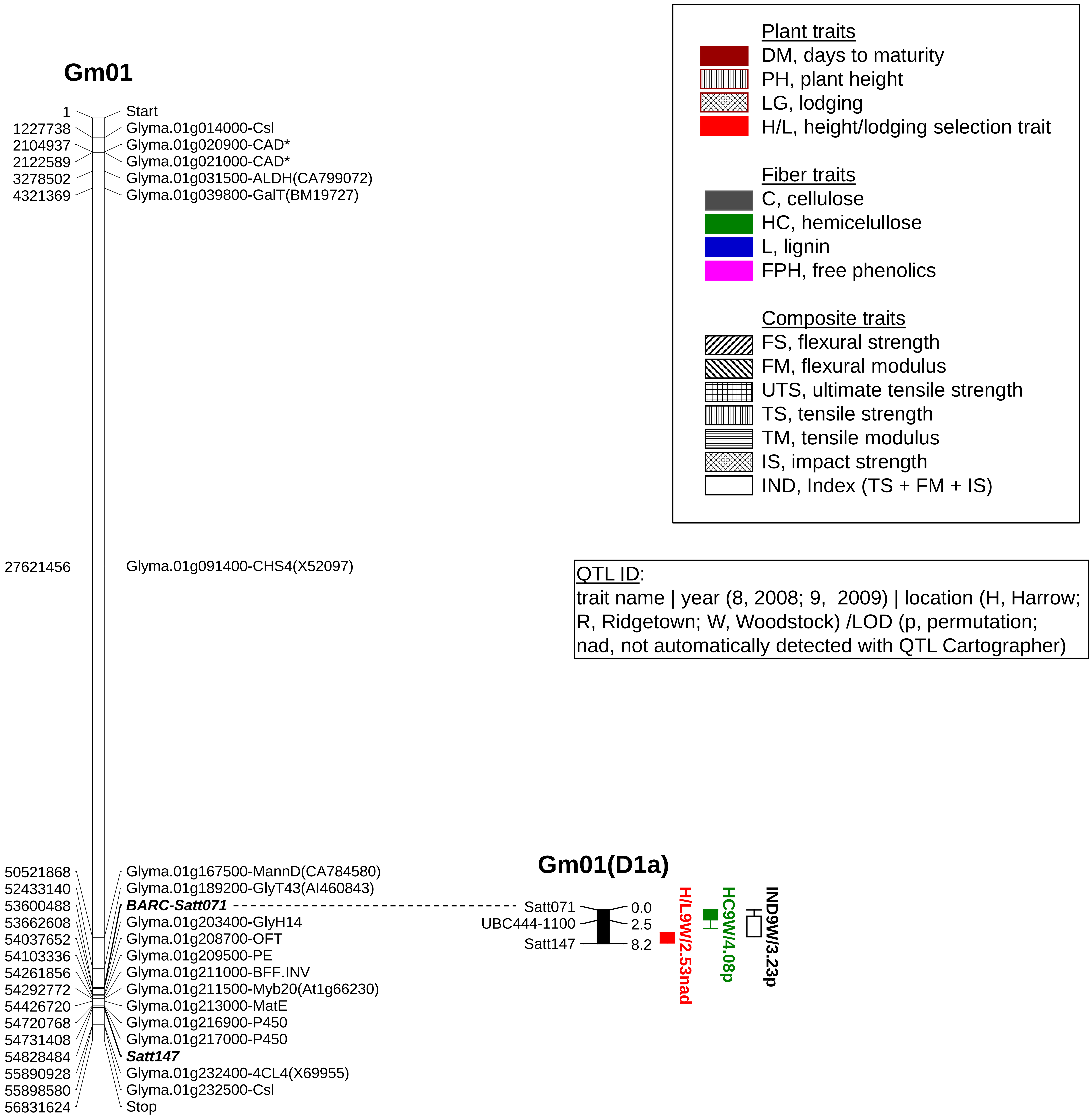

Fig. G in S1 File. Comparison of the soybean RG10 x OX948 stem fiber-based composite QTL map (right) with the *G. max* Wm82.a2.v1 sequence map (left). Linkage map - QTL were detected using the Composite Interval Mapping with Windows QTL Cartographer v.2.5_009 [The settings used: map function Kosambi, a walk speed of 2cM, five control markers, model 6 (standard), forward and backward regression (method 3), and probabilities of 0.05]. Sequence (*in silico*) map - Initial mapping was done by BLASTing cell wall gene sequences against soybean genome (*G. max* Wm82.a2.v1) in Phytozome 9.1; additional sequences were then added to newly identified QTL regions [by scaning (200 kb walk) the soybean genome for genes potentially involved in cell wall biosynthesis/modifcation in Phytozome 9.1 and/or using *G. max* Wm82.a2.v1 annotation and feature coordinate files from SoyBase]. Maps were linked by common SSR markers. Mapped fiber genes are indicated in bold.

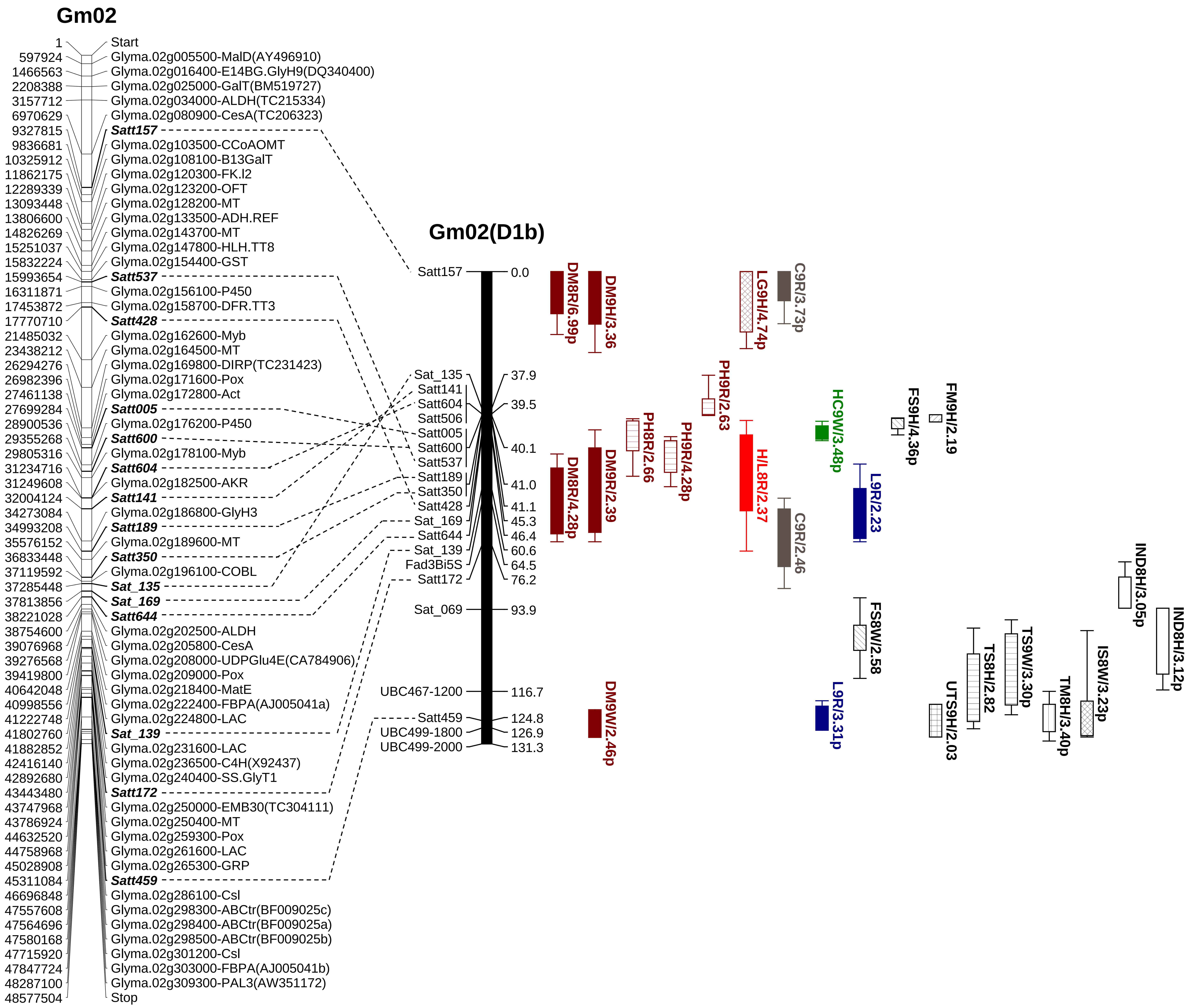
Gm02
1 Start
597924 Glyma.02g005500-MalD(AY496910)
1466563 Glyma.02g016400-E14BG.GlyH9(DQ340400)
2208388 Glyma.02g025000-GalT(BM519727)
3157712 Glyma.02g034000-ALDH(TC215334)
6970629 Glyma.02g080900-CesA(TC206323)
9327815 Satt157
9836681 Glyma.02g103500-CCoAOMT
10325912 Glyma.02g108100-B13GalT
11862175 Glyma.02g120300-FK.I2
12289339 Glyma.02g123200-OFT
13093448 Glyma.02g128200-MT
13806600 Glyma.02g133500-ADH.REF
14826269 Glyma.02g143700-MT
15251037 Glyma.02g147800-HLH.TT8
15832224 Glyma.02g154400-GST
15993654 Satt537
16311871 Glyma.02g156100-P450
17453872 Glyma.02g158700-DFR.TT3
17770710 Satt428
21485032 Glyma.02g162600-Myb
23438212 Glyma.02g164500-MT
26294276 Glyma.02g169800-DIRP(TC231423)
26982396 Glyma.02g171600-Pox
27461138 Glyma.02g172800-Act
27699284 Satt005
28900536 Glyma.02g176200-P450
29355268 Satt600
29805316 Glyma.02g178100-Myb
31234716 Satt604
31249608 Glyma.02g182500-AKR
32004124 Satt141
34273084 Glyma.02g186800-GlyH3
34993208 Satt189
35576152 Glyma.02g189600-MT
36833448 Satt350
37119592 Glyma.02g196100-COBL
37285448 Sat_135
37813856 Sat_169
38221028 Satt644
38754600 Glyma.02g202500-ALDH
39076968 Glyma.02g205800-CesA
39276568 Glyma.02g208000-UDPGlu4E(CA784906)
39419800 Glyma.02g209000-Pox
40642048 Glyma.02g218400-MatE
40998556 Glyma.02g222400-FBPA(AJ005041a)
41222748 Glyma.02g224800-LAC
41802760 Sat_139
41882852 Glyma.02g231600-LAC
42416140 Glyma.02g236500-C4H(X92437)
42892680 Glyma.02g240400-SS.GlyT1
43443480 Satt172
43747968 Glyma.02g250000-EMB30(TC304111)
43786924 Glyma.02g250400-MT
44632520 Glyma.02g259300-Pox
44758968 Glyma.02g261600-LAC
45028908 Glyma.02g265300-GRP
45311084 Satt459
46696848 Glyma.02g286100-Csl
47557608 Glyma.02g298300-ABCtr(BF009025c)
47564696 Glyma.02g298400-ABCtr(BF009025a)
47580168 Glyma.02g298500-ABCtr(BF009025b)
47715920 Glyma.02g301200-Csl
47847724 Glyma.02g303000-FBPA(AJ005041b)
48287100 Glyma.02g309300-PAL3(AW351172)
48577504 Stop
Gm02(D1b)
Satt157 0.0
Sat_135 37.9
Satt141
Satt604 39.5
Satt506
Satt005 40.1
Satt600
Satt537
Satt189 41.0
Satt350
Satt428 41.1
Sat_169 45.3
Satt644 46.4
Sat_139 60.6
Fad3Bi5S 64.5
Satt172 76.2
Sat_069 93.9
UBC467-1200 116.7
Satt459 124.8
UBC499-1800 126.9
UBC499-2000 131.3
DM8R/6.99p
DM9H/3.36
LG9H/4.74p
C9R/3.73p
PH9R/2.63
PH8R/2.66
PH9R/4.28p
HC9W/3.48p
FS9H/4.36p
FM9H/2.19
DM8R/4.28p
DM9R/2.39
HIL8R/2.37
C9R/2.46
L9R/2.23
IND8H/3.05p
FS8W/2.58
IND8H/3.12p
TS9W/3.30p
TS8H/2.82
IS8W/3.23p
TM8H/3.40p
UTS9H/2.03
L9R/3.31p
DM9W/2.46p

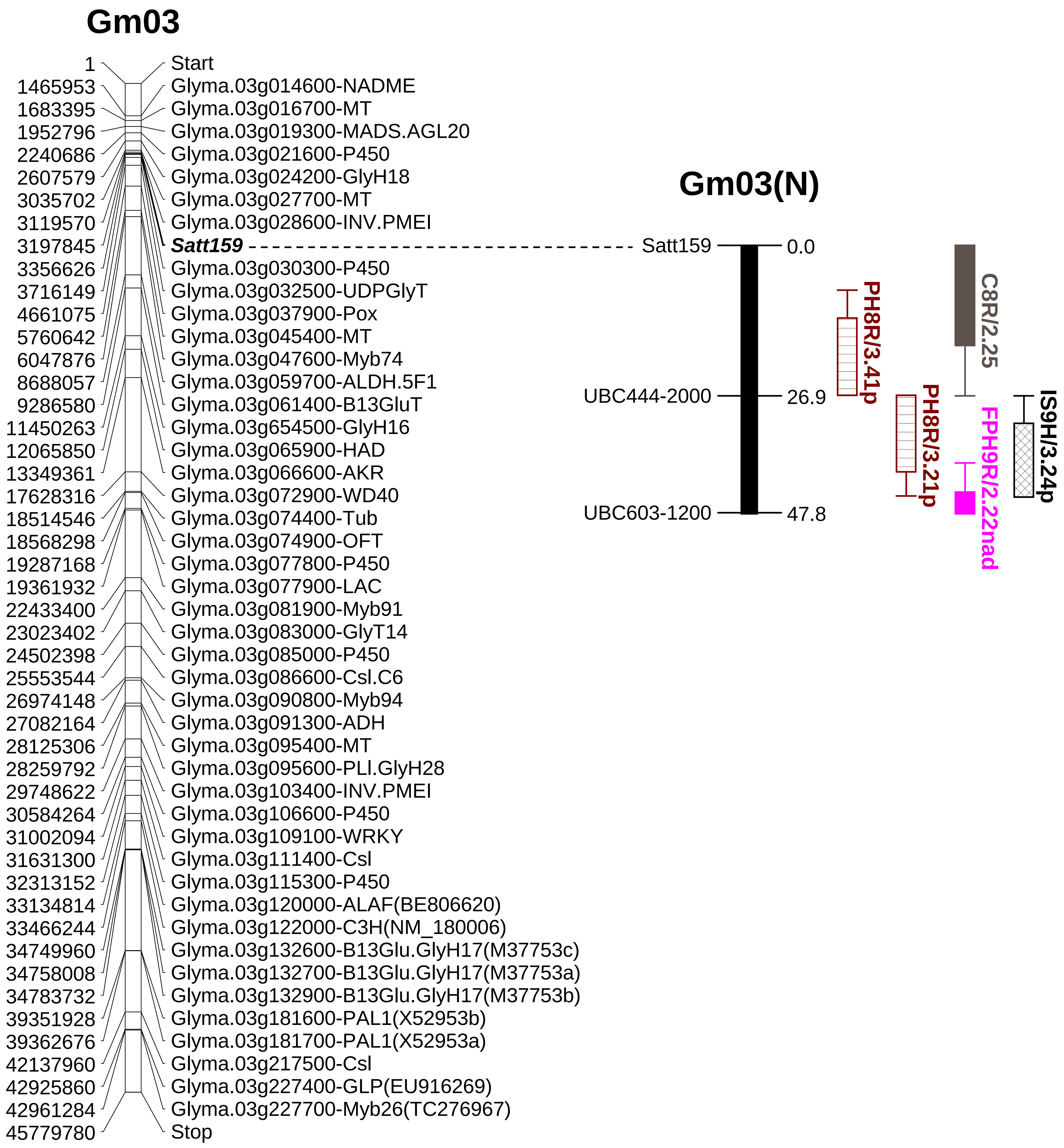
Gm03
1 Start
1465953 Glyma.03g014600-NADME
1683395 Glyma.03g016700-MT
1952796 Glyma.03g019300-MADS.AGL20
2240686 Glyma.03g021600-P450
2607579 Glyma.03g024200-GlyH18
3035702 Glyma.03g027700-MT
3119570 Glyma.03g028600-INV.PMEI
3197845 Satt159
3356626 Glyma.03g030300-P450
3716149 Glyma.03g032500-UDPGlyT
4661075 Glyma.03g037900-Pox
5760642 Glyma.03g045400-MT
6047876 Glyma.03g047600-Myb74
8688057 Glyma.03g059700-ALDH.5F1
9286580 Glyma.03g061400-B13GluT
11450263 Glyma.03g654500-GlyH16
12065850 Glyma.03g065900-HAD
13349361 Glyma.03g066600-AKR
17628316 Glyma.03g072900-WD40
18514546 Glyma.03g074400-Tub
18568298 Glyma.03g074900-OFT
19287168 Glyma.03g077800-P450
19361932 Glyma.03g077900-LAC
22433400 Glyma.03g081900-Myb91
23023402 Glyma.03g083000-GlyT14
24502398 Glyma.03g085000-P450
25553544 Glyma.03g086600-Csl.C6
26974148 Glyma.03g090800-Myb94
27082164 Glyma.03g091300-ADH
28125306 Glyma.03g095400-MT
28259792 Glyma.03g095600-PLI.GlyH28
29748622 Glyma.03g103400-INV.PMEI
30584264 Glyma.03g106600-P450
31002094 Glyma.03g109100-WRKY
31631300 Glyma.03g111400-Csl
32313152 Glyma.03g115300-P450
33134814 Glyma.03g120000-ALAF(BE806620)
33466244 Glyma.03g122000-C3H(NM_180006)
34749960 Glyma.03g132600-B13Glu.GlyH17(M37753c)
34758008 Glyma.03g132700-B13Glu.GlyH17(M37753a)
34783732 Glyma.03g132900-B13Glu.GlyH17(M37753b)
39351928 Glyma.03g181600-PAL1(X52953b)
39362676 Glyma.03g181700-PAL1(X52953a)
42137960 Glyma.03g217500-Csl
42925860 Glyma.03g227400-GLP(EU916269)
42961284 Glyma.03g227700-Myb26(TC276967)
45779780 Stop
Gm03(N)
Satt159 0.0
UBC444-2000 26.9
UBC603-1200 47.8
PH8R/3.41p
PH8R/3.21p
C8R/2.25
FPH9R/2.22nad
IS9H/3.24p

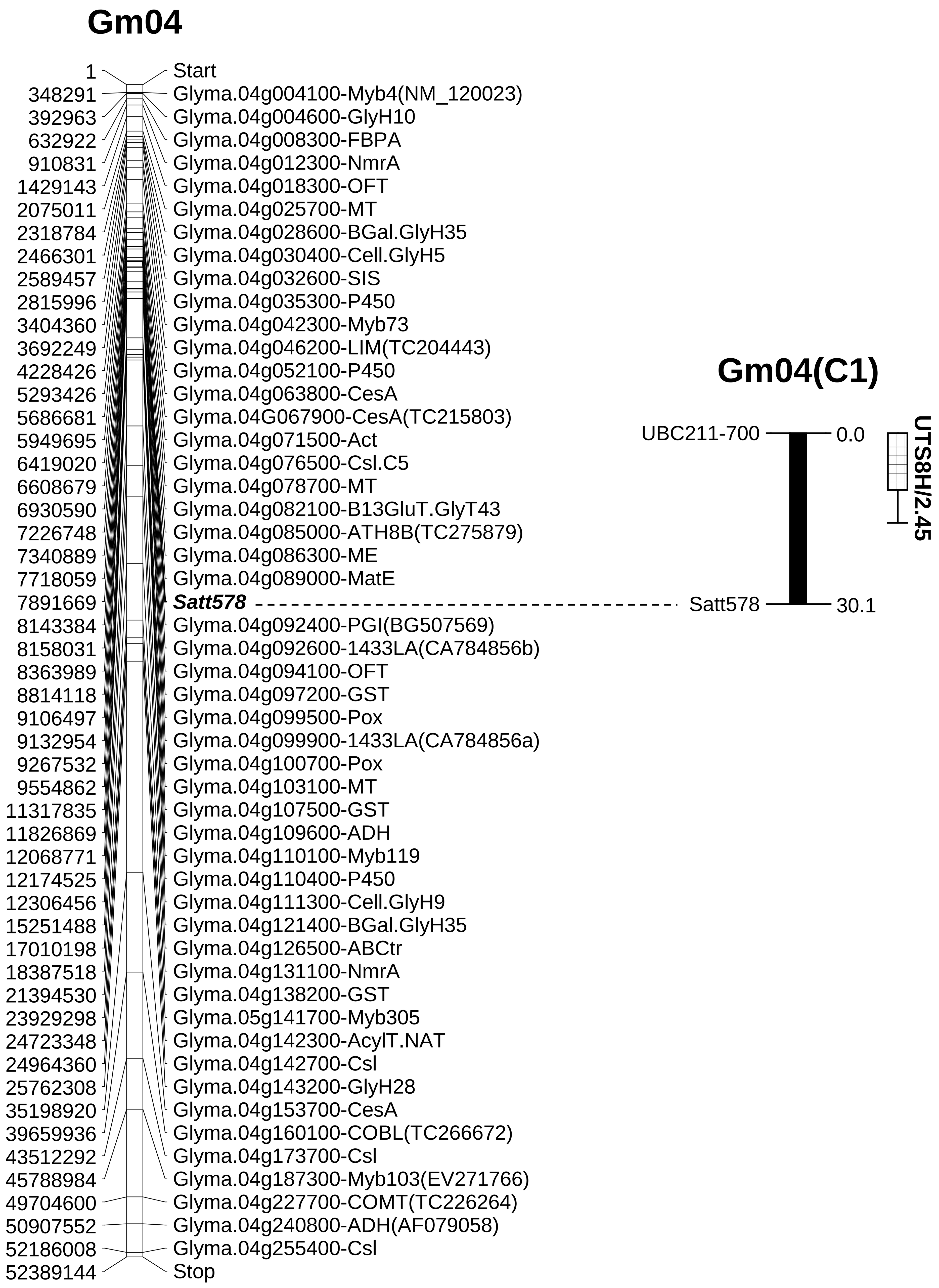

Gm04
1 Start
348291 Glyma.04g004100-Myb4(NM_120023)
392963 Glyma.04g004600-GlyH10
632922 Glyma.04g008300-FBPA
910831 Glyma.04g012300-NmrA
1429143 Glyma.04g018300-OFT
2075011 Glyma.04g025700-MT
2318784 Glyma.04g028600-BGal.GlyH35
2466301 Glyma.04g030400-Cell.GlyH5
2589457 Glyma.04g032600-SIS
2815996 Glyma.04g035300-P450
3404360 Glyma.04g042300-Myb73
3692249 Glyma.04g046200-LIM(TC204443)
4228426 Glyma.04g052100-P450
5293426 Glyma.04g063800-CesA
5686681 Glyma.04G067900-CesA(TC215803)
5949695 Glyma.04g071500-Act
6419020 Glyma.04g076500-Csl.C5
6608679 Glyma.04g078700-MT
6930590 Glyma.04g082100-B13GluT.GlyT43
7226748 Glyma.04g085000-ATH8B(TC275879)
7340889 Glyma.04g086300-ME
7718059 Glyma.04g089000-MatE
7891669 Satt578
8143384 Glyma.04g092400-PGI(BG507569)
8158031 Glyma.04g092600-1433LA(CA784856b)
8363989 Glyma.04g094100-OFT
8814118 Glyma.04g097200-GST
9106497 Glyma.04g099500-Pox
9132954 Glyma.04g099900-1433LA(CA784856a)
9267532 Glyma.04g100700-Pox
9554862 Glyma.04g103100-MT
11317835 Glyma.04g107500-GST
11826869 Glyma.04g109600-ADH
12068771 Glyma.04g110100-Myb119
12174525 Glyma.04g110400-P450
12306456 Glyma.04g111300-Cell.GlyH9
15251488 Glyma.04g121400-BGal.GlyH35
17010198 Glyma.04g126500-ABCtr
18387518 Glyma.04g131100-NmrA
21394530 Glyma.04g138200-GST
23929298 Glyma.05g141700-Myb305
24723348 Glyma.04g142300-AcylT.NAT
24964360 Glyma.04g142700-Csl
25762308 Glyma.04g143200-GlyH28
35198920 Glyma.04g153700-CesA
39659936 Glyma.04g160100-COBL(TC266672)
43512292 Glyma.04g173700-Csl
45788984 Glyma.04g187300-Myb103(EV271766)
49704600 Glyma.04g227700-COMT(TC226264)
50907552 Glyma.04g240800-ADH(AF079058)
52186008 Glyma.04g255400-Csl
52389144 Stop
Gm04(C1)
UBC211-700 0.0
Satt578 30.1
UTS8H/2.45

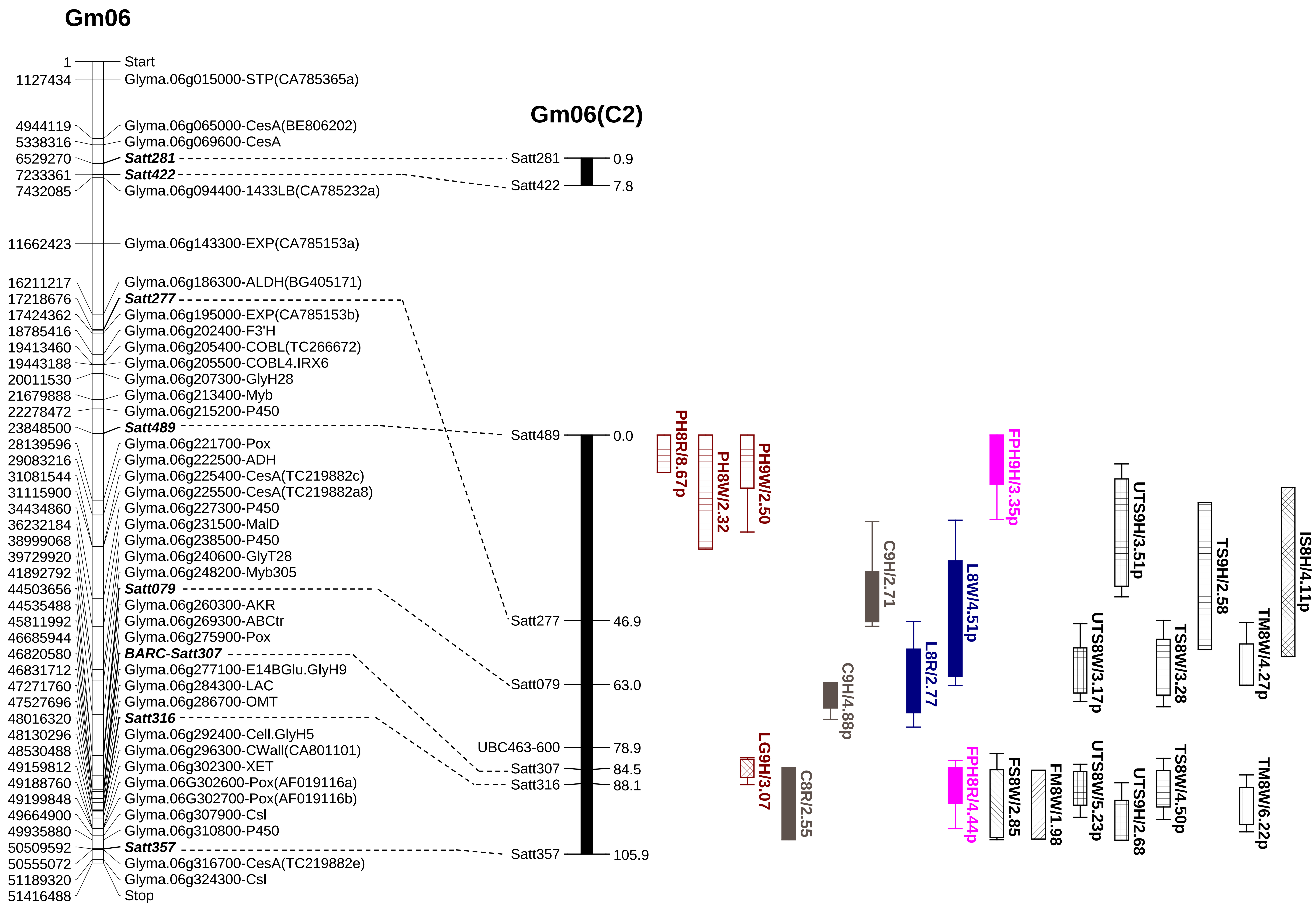

Gm06
1 Start
1127434 Glyma.06g015000-STP(CA785365a)
4944119 Glyma.06g065000-CesA(BE806202)
5338316 Glyma.06g069600-CesA
6529270 Satt281
7233361 Satt422
7432085 Glyma.06g094400-1433LB(CA785232a)
11662423 Glyma.06g143300-EXP(CA785153a)
16211217 Glyma.06g186300-ALDH(BG405171)
17218676 Satt277
17424362 Glyma.06g195000-EXP(CA785153b)
18785416 Glyma.06g202400-F3'H
19413460 Glyma.06g205400-COBL(TC266672)
19443188 Glyma.06g205500-COBL4.IRX6
20011530 Glyma.06g207300-GlyH28
21679888 Glyma.06g213400-Myb
22278472 Glyma.06g215200-P450
23848500 Satt489
28139596 Glyma.06g221700-Pox
29083216 Glyma.06g222500-ADH
31081544 Glyma.06g225400-CesA(TC219882c)
31115900 Glyma.06g225500-CesA(TC219882a8)
34434860 Glyma.06g227300-P450
36232184 Glyma.06g231500-MalD
38999068 Glyma.06g238500-P450
39729920 Glyma.06g240600-GlyT28
41892792 Glyma.06g248200-Myb305
44503656 Satt079
44535488 Glyma.06g260300-AKR
45811992 Glyma.06g269300-ABCtr
46685944 Glyma.06g275900-Pox
46820580 BARC-Satt307
46831712 Glyma.06g277100-E14BGlu.GlyH9
47271760 Glyma.06g284300-LAC
47527696 Glyma.06g286700-OMT
48016320 Satt316
48130296 Glyma.06g292400-Cell.GlyH5
48530488 Glyma.06g296300-CWall(CA801101)
49159812 Glyma.06g302300-XET
49188760 Glyma.06G302600-Pox(AF019116a)
49199848 Glyma.06G302700-Pox(AF019116b)
49664900 Glyma.06g307900-Csl
49935880 Glyma.06g310800-P450
50509592 Satt357
50555072 Glyma.06g316700-CesA(TC219882e)
51189320 Glyma.06g324300-Csl
51416488 Stop
Gm06(C2)
Satt281 0.9
Satt422 7.8
Satt489 0.0
Satt277 46.9
Satt079 63.0
UBC463-600 78.9
Satt307 84.5
Satt316 88.1
Satt357 105.9
PH8R/8.67p
PH8W/2.32
PH9W/2.50
LG9H/3.07
C8R/2.55
C9H/4.88p
C9H/2.71
L8R/2.77
L8W/4.51p
FPH8R/4.44p
FPH9H/3.35p
FS8W/2.85
FM8W/1.98
UTS8W/5.23p
UTS8W/3.17p
UTS9H/3.51p
UTS9H/2.68
TS8W/3.28
TS8W/4.50p
TS9H/2.58
TM8W/4.27p
TM8W/6.22p
IS8H/4.11p

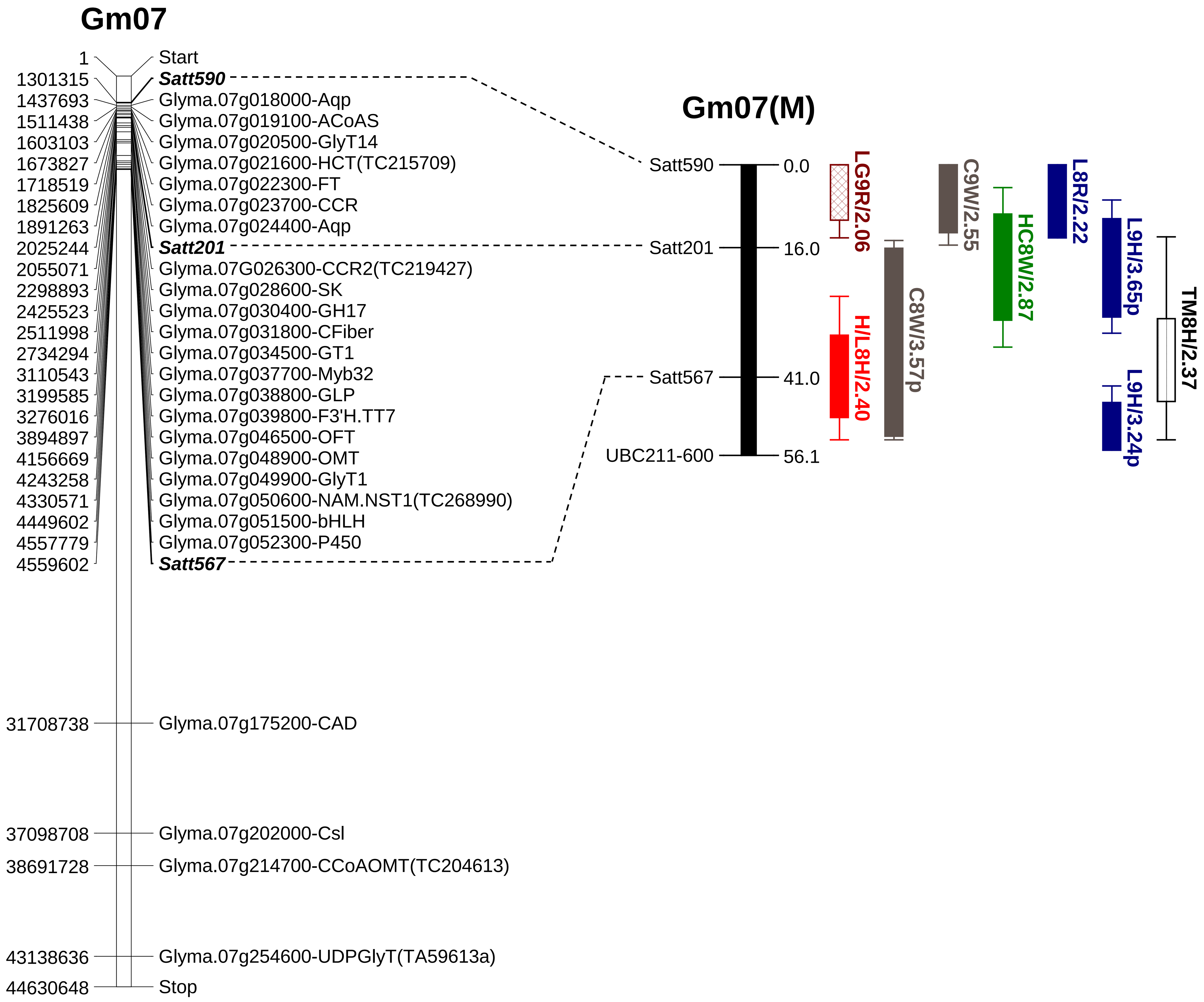
Gm07
1
1301315
1437693
1511438
1603103
1673827
1718519
1825609
1891263
2025244
2055071
2298893
2425523
2511998
2734294
3110543
3199585
3276016
3894897
4156669
4243258
4330571
4449602
4557779
4559602
31708738
37098708
38691728
43138636
44630648
Start
Satt590
Glyma.07g018000-Aqp
Glyma.07g019100-ACoAS
Glyma.07g020500-GlyT14
Glyma.07g021600-HCT(TC215709)
Glyma.07g022300-FT
Glyma.07g023700-CCR
Glyma.07g024400-Aqp
Satt201
Glyma.07G026300-CCR2(TC219427)
Glyma.07g028600-SK
Glyma.07g030400-GH17
Glyma.07g031800-CFiber
Glyma.07g034500-GT1
Glyma.07g037700-Myb32
Glyma.07g038800-GLP
Glyma.07g039800-F3'H.TT7
Glyma.07g046500-OFT
Glyma.07g048900-OMT
Glyma.07g049900-GlyT1
Glyma.07g050600-NAM.NST1(TC268990)
Glyma.07g051500-bHLH
Glyma.07g052300-P450
Satt567
Glyma.07g175200-CAD
Glyma.07g202000-Csl
Glyma.07g214700-CCoAOMT(TC204613)
Glyma.07g254600-UDPGlyT(TA59613a)
Stop
Gm07(M)
Satt590
Satt201
Satt567
UBC211-600
0.0
16.0
41.0
56.1
LG9R/2.06
H/L8H/2.40
C8W/3.57p
C9W/2.55
HC8W/2.87
L8R/2.22
L9H/3.65p
L9H/3.24p
TM8H/2.37

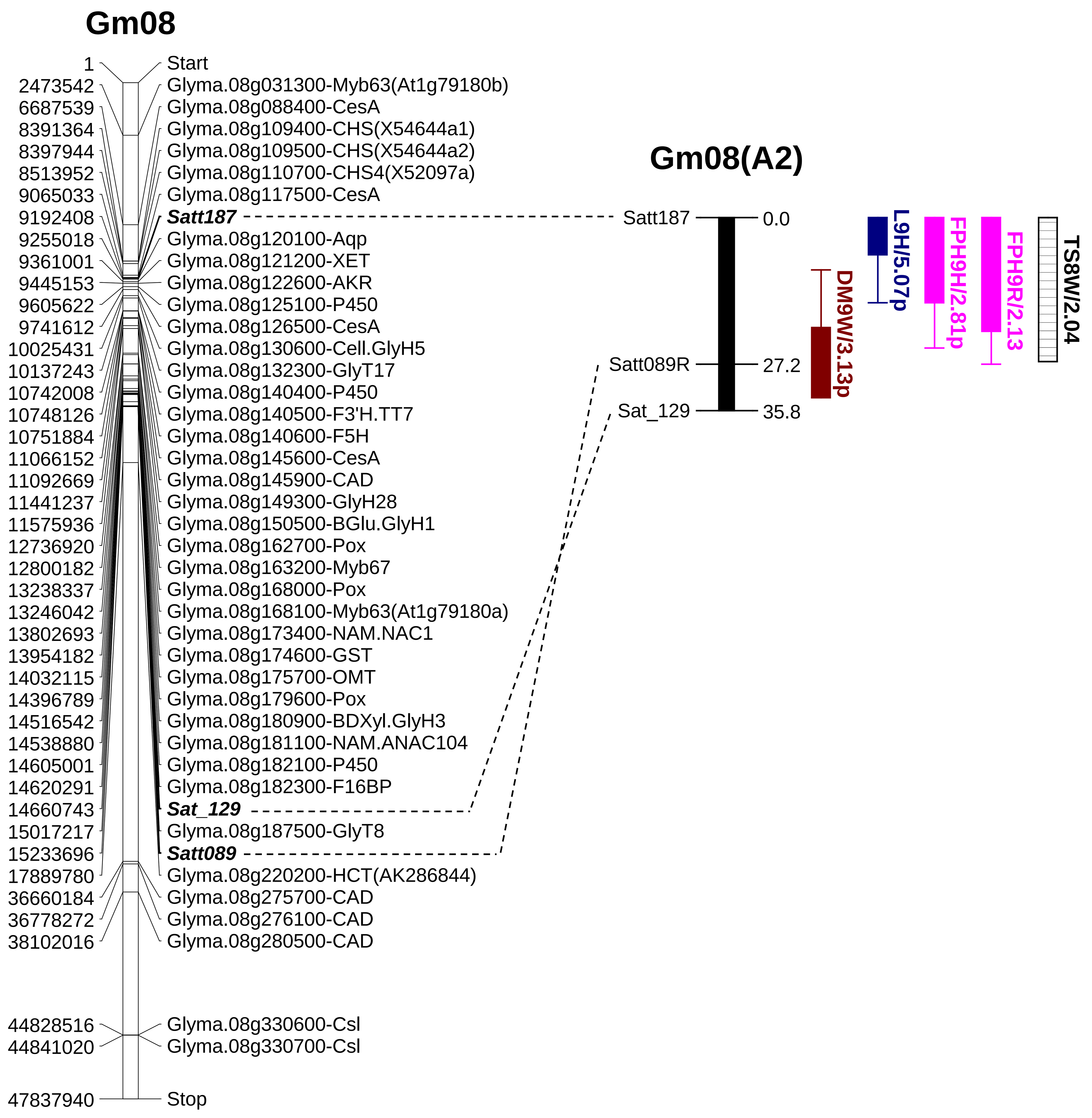
Gm08
1 Start
2473542 Glyma.08g031300-Myb63(At1g79180b)
6687539 Glyma.08g088400-CesA
8391364 Glyma.08g109400-CHS(X54644a1)
8397944 Glyma.08g109500-CHS(X54644a2)
8513952 Glyma.08g110700-CHS4(X52097a)
9065033 Glyma.08g117500-CesA
9192408 Satt187
9255018 Glyma.08g120100-Aqp
9361001 Glyma.08g121200-XET
9445153 Glyma.08g122600-AKR
9605622 Glyma.08g125100-P450
9741612 Glyma.08g126500-CesA
10025431 Glyma.08g130600-Cell.GlyH5
10137243 Glyma.08g132300-GlyT17
10742008 Glyma.08g140400-P450
10748126 Glyma.08g140500-F3'H.TT7
10751884 Glyma.08g140600-F5H
11066152 Glyma.08g145600-CesA
11092669 Glyma.08g145900-CAD
11441237 Glyma.08g149300-GlyH28
11575936 Glyma.08g150500-BGlu.GlyH1
12736920 Glyma.08g162700-Pox
12800182 Glyma.08g163200-Myb67
13238337 Glyma.08g168000-Pox
13246042 Glyma.08g168100-Myb63(At1g79180a)
13802693 Glyma.08g173400-NAM.NAC1
13954182 Glyma.08g174600-GST
14032115 Glyma.08g175700-OMT
14396789 Glyma.08g179600-Pox
14516542 Glyma.08g180900-BDXyl.GlyH3
14538880 Glyma.08g181100-NAM.ANAC104
14605001 Glyma.08g182100-P450
14620291 Glyma.08g182300-F16BP
14660743 Sat_129
15017217 Glyma.08g187500-GlyT8
15233696 Satt089
17889780 Glyma.08g220200-HCT(AK286844)
36660184 Glyma.08g275700-CAD
36778272 Glyma.08g276100-CAD
38102016 Glyma.08g280500-CAD
44828516 Glyma.08g330600-Csl
44841020 Glyma.08g330700-Csl
47837940 Stop
Gm08(A2)
Satt187 0.0
Satt089R 27.2
Sat_129 35.8
DM9W/3.13p
L9H/5.07p
FPH9H/2.81p
FPH9R/2.13
TS8W/2.04

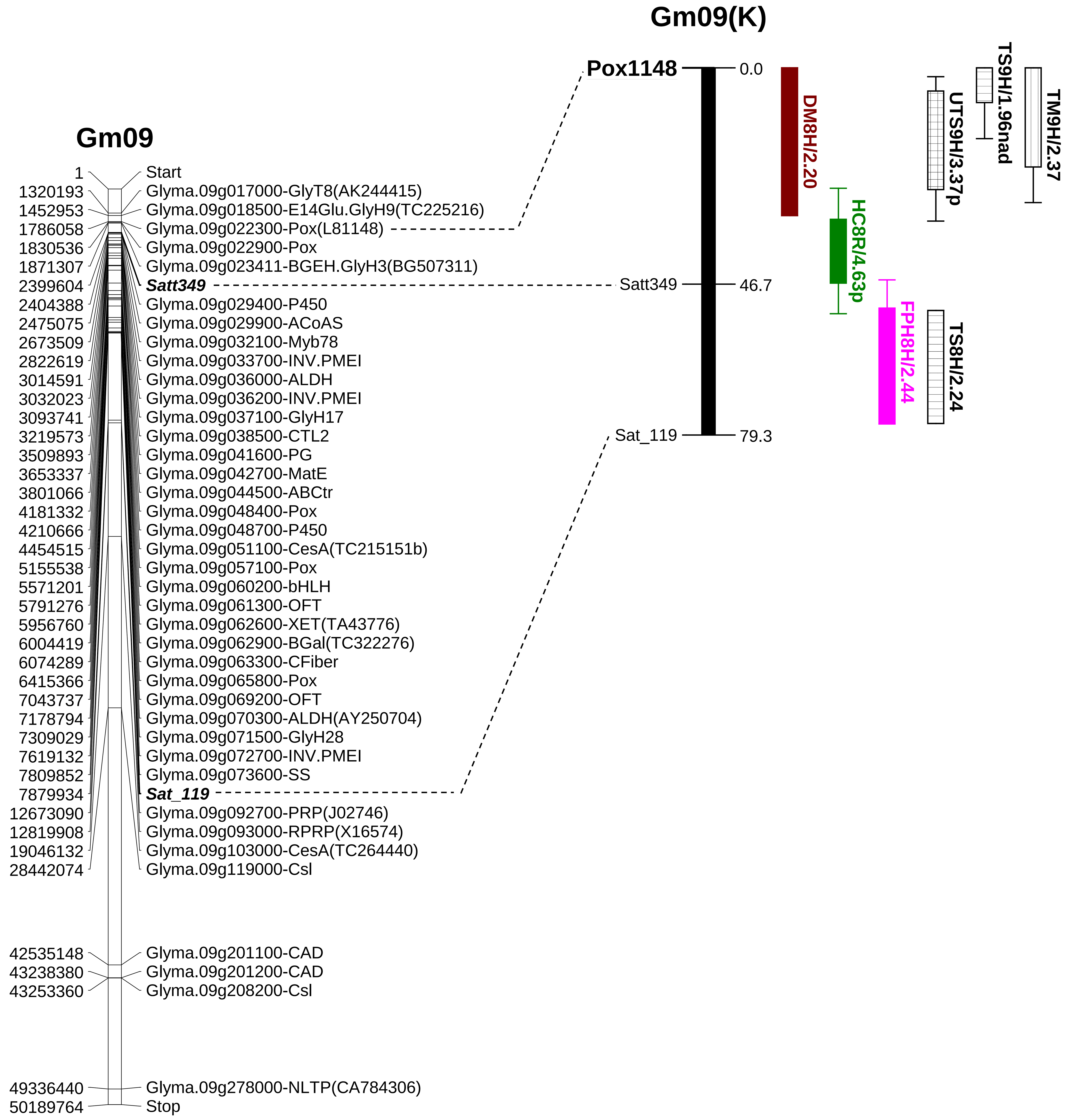
Gm09(K)
Pox1148
0.0
Satt349
46.7
Sat_119
79.3
DM8H/2.20
HC8R/4.63p
FPH8H/2.44
UTS9H/3.37p
TS9H/1.96nad
TM9H/2.37
TS8H/2.24
Gm09
1 Start
1320193 Glyma.09g017000-GlyT8(AK244415)
1452953 Glyma.09g018500-E14Glu.GlyH9(TC225216)
1786058 Glyma.09g022300-Pox(L81148)
1830536 Glyma.09g022900-Pox
1871307 Glyma.09g023411-BGEH.GlyH3(BG507311)
2399604 Satt349
2404388 Glyma.09g029400-P450
2475075 Glyma.09g029900-ACoAS
2673509 Glyma.09g032100-Myb78
2822619 Glyma.09g033700-INV.PMEI
3014591 Glyma.09g036000-ALDH
3032023 Glyma.09g036200-INV.PMEI
3093741 Glyma.09g037100-GlyH17
3219573 Glyma.09g038500-CTL2
3509893 Glyma.09g041600-PG
3653337 Glyma.09g042700-MatE
3801066 Glyma.09g044500-ABCtr
4181332 Glyma.09g048400-Pox
4210666 Glyma.09g048700-P450
4454515 Glyma.09g051100-CesA(TC215151b)
5155538 Glyma.09g057100-Pox
5571201 Glyma.09g060200-bHLH
5791276 Glyma.09g061300-OFT
5956760 Glyma.09g062600-XET(TA43776)
6004419 Glyma.09g062900-BGal(TC322276)
6074289 Glyma.09g063300-CFiber
6415366 Glyma.09g065800-Pox
7043737 Glyma.09g069200-OFT
7178794 Glyma.09g070300-ALDH(AY250704)
7309029 Glyma.09g071500-GlyH28
7619132 Glyma.09g072700-INV.PMEI
7809852 Glyma.09g073600-SS
7879934 Sat_119
12673090 Glyma.09g092700-PRP(J02746)
12819908 Glyma.09g093000-RPRP(X16574)
19046132 Glyma.09g103000-CesA(TC264440)
28442074 Glyma.09g119000-Csl
42535148 Glyma.09g201100-CAD
43238380 Glyma.09g201200-CAD
43253360 Glyma.09g208200-Csl
49336440 Glyma.09g278000-NLTP(CA784306)
50189764 Stop

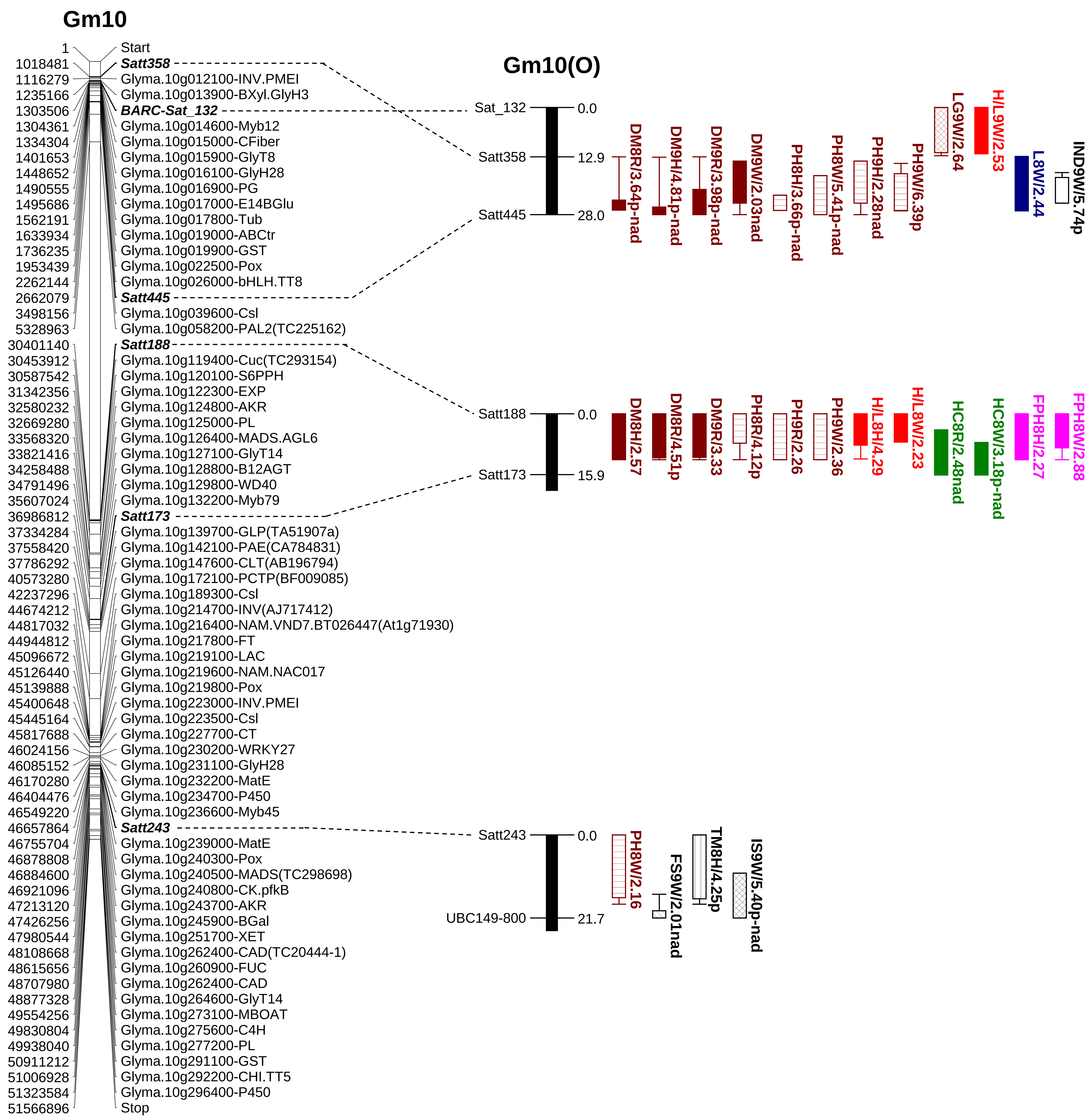

Gm10
1 Start
1018481 Satt358
1116279 Glyma.10g012100-INV.PMEI
1235166 Glyma.10g013900-BXyl.GlyH3
1303506 BARC-Sat_132
1304361 Glyma.10g014600-Myb12
1334304 Glyma.10g015000-CFiber
1401653 Glyma.10g015900-GlyT8
1448652 Glyma.10g016100-GlyH28
1490555 Glyma.10g016900-PG
1495686 Glyma.10g017000-E14BGlu
1562191 Glyma.10g017800-Tub
1633934 Glyma.10g019000-ABCtr
1736235 Glyma.10g019900-GST
1953439 Glyma.10g022500-Pox
2262144 Glyma.10g026000-bHLH.TT8
2662079 Satt445
3498156 Glyma.10g039600-Csl
5328963 Glyma.10g058200-PAL2(TC225162)
30401140 Satt188
30453912 Glyma.10g119400-Cuc(TC293154)
30587542 Glyma.10g120100-S6PPH
31342356 Glyma.10g122300-EXP
32580232 Glyma.10g124800-AKR
32669280 Glyma.10g125000-PL
33568320 Glyma.10g126400-MADS.AGL6
33821416 Glyma.10g127100-GlyT14
34258488 Glyma.10g128800-B12AGT
34791496 Glyma.10g129800-WD40
35607024 Glyma.10g132200-Myb79
36986812 Satt173
37334284 Glyma.10g139700-GLP(TA51907a)
37558420 Glyma.10g142100-PAE(CA784831)
37786292 Glyma.10g147600-CLT(AB196794)
40573280 Glyma.10g172100-PCTP(BF009085)
42237296 Glyma.10g189300-Csl
44674212 Glyma.10g214700-INV(AJ717412)
44817032 Glyma.10g216400-NAM.VND7.BT026447(At1g71930)
44944812 Glyma.10g217800-FT
45096672 Glyma.10g219100-LAC
45126440 Glyma.10g219600-NAM.NAC017
45139888 Glyma.10g219800-Pox
45400648 Glyma.10g223000-INV.PMEI
45445164 Glyma.10g223500-Csl
45817688 Glyma.10g227700-CT
46024156 Glyma.10g230200-WRKY27
46085152 Glyma.10g231100-GlyH28
46170280 Glyma.10g232200-MatE
46404476 Glyma.10g234700-P450
46549220 Glyma.10g236600-Myb45
46657864 Satt243
46755704 Glyma.10g239000-MatE
46878808 Glyma.10g240300-Pox
46884600 Glyma.10g240500-MADS(TC298698)
46921096 Glyma.10g240800-CK.pfkB
47213120 Glyma.10g243700-AKR
47426256 Glyma.10g245900-BGal
47980544 Glyma.10g251700-XET
48108668 Glyma.10g262400-CAD(TC20444-1)
48615656 Glyma.10g260900-FUC
48707980 Glyma.10g262400-CAD
48877328 Glyma.10g264600-GlyT14
49554256 Glyma.10g273100-MBOAT
49830804 Glyma.10g275600-C4H
49938040 Glyma.10g277200-PL
50911212 Glyma.10g291100-GST
51006928 Glyma.10g292200-CHI.TT5
51323584 Glyma.10g296400-P450
51566896 Stop
Gm10(O)
Sat_132 0.0
Satt358 12.9
Satt445 28.0
DM8R/3.64p-nad
DM9H/4.81p-nad
DM9R/3.98p-nad
DM9W/2.03nad
PH8H/3.66p-nad
PH8W/5.41p-nad
PH9H/2.28nad
PH9W/6.39p
LG9W/2.64
H/L9W/2.53
L8W/2.44
IND9W/5.74p
Satt188 0.0
Satt173 15.9
DM8H/2.57
DM8R/4.51p
DM9R/3.33
PH8R/4.12p
PH9R/2.26
PH9W/2.36
H/L8H/4.29
H/L8W/2.23
HC8R/2.48nad
HC8W/3.18p-nad
FPH8H/2.27
FPH8W/2.88
Satt243 0.0
UBC149-800 21.7
PH8W/2.16
FS9W/2.01nad
TM8H/4.25p
IS9W/5.40p-nad

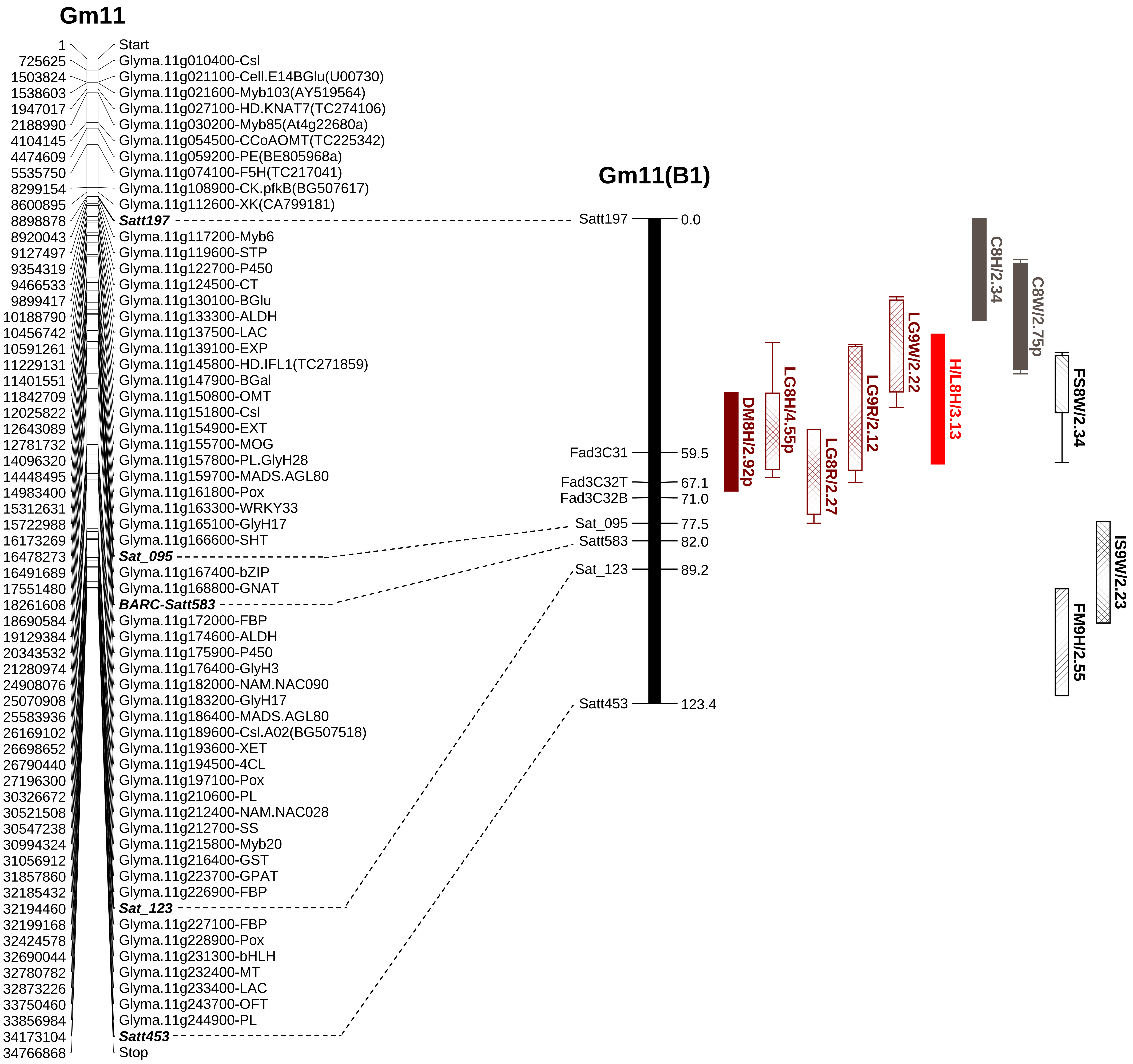
Gm11
1 Start
725625 Glyma.11g010400-CsI
1503824 Glyma.11g021100-Cell.E14BGlu(U00730)
1538603 Glyma.11g021600-Myb103(AY519564)
1947017 Glyma.11g027100-HD.KNAT7(TC274106)
2188990 Glyma.11g030200-Myb85(At4g22680a)
4104145 Glyma.11g054500-CCoAOMT(TC225342)
4474609 Glyma.11g059200-PE(BE805968a)
5535750 Glyma.11g074100-F5H(TC217041)
8299154 Glyma.11g108900-CK.pfkB(BG507617)
8600895 Glyma.11g112600-XK(CA799181)
8898878 Satt197
8920043 Glyma.11g117200-Myb6
9127497 Glyma.11g119600-STP
9354319 Glyma.11g122700-P450
9466533 Glyma.11g124500-CT
9899417 Glyma.11g130100-BGlu
10188790 Glyma.11g133300-ALDH
10456742 Glyma.11g137500-LAC
10591261 Glyma.11g139100-EXP
11229131 Glyma.11g145800-HD.IFL1(TC271859)
11401551 Glyma.11g147900-BGal
11842709 Glyma.11g150800-OMT
12025822 Glyma.11g151800-CsI
12643089 Glyma.11g154900-EXT
12781732 Glyma.11g155700-MOG
14096320 Glyma.11g157800-PL.GlyH28
14448495 Glyma.11g159700-MADS.AGL80
14983400 Glyma.11g161800-Pox
15312631 Glyma.11g163300-WRKY33
15722988 Glyma.11g165100-GlyH17
16173269 Glyma.11g166600-SHT
16478273 Sat_095
16491689 Glyma.11g167400-bZIP
17551480 Glyma.11g168800-GNAT
18261608 BARC-Satt583
18690584 Glyma.11g172000-FBP
19129384 Glyma.11g174600-ALDH
20343532 Glyma.11g175900-P450
21280974 Glyma.11g176400-GlyH3
24908076 Glyma.11g182000-NAM.NAC090
25070908 Glyma.11g183200-GlyH17
25583936 Glyma.11g186400-MADS.AGL80
26169102 Glyma.11g189600-Csl.A02(BG507518)
26698652 Glyma.11g193600-XET
26790440 Glyma.11g194500-4CL
27196300 Glyma.11g197100-Pox
30326672 Glyma.11g210600-PL
30521508 Glyma.11g212400-NAM.NAC028
30547238 Glyma.11g212700-SS
30994324 Glyma.11g215800-Myb20
31056912 Glyma.11g216400-GST
31857860 Glyma.11g223700-GPAT
32185432 Glyma.11g226900-FBP
32194460 Sat_123
32199168 Glyma.11g227100-FBP
32424578 Glyma.11g228900-Pox
32690044 Glyma.11g231300-bHLH
32780782 Glyma.11g232400-MT
32873226 Glyma.11g233400-LAC
33750460 Glyma.11g243700-OFT
33856984 Glyma.11g244900-PL
34173104 Satt453
34766868 Stop
Gm11(B1)
Satt197 0.0
Fad3C31 59.5
Fad3C32T 67.1
Fad3C32B 71.0
Sat_095 77.5
Satt583 82.0
Sat_123 89.2
Satt453 123.4
C8H/2.34
C8W/2.75p
LG9W/2.22
H/L8H/3.13
FS8W/2.34
LG9R/2.12
LG8H/4.55p
LG8R/2.27
DM8H/2.92p
IS9W/2.23
FM9H/2.55

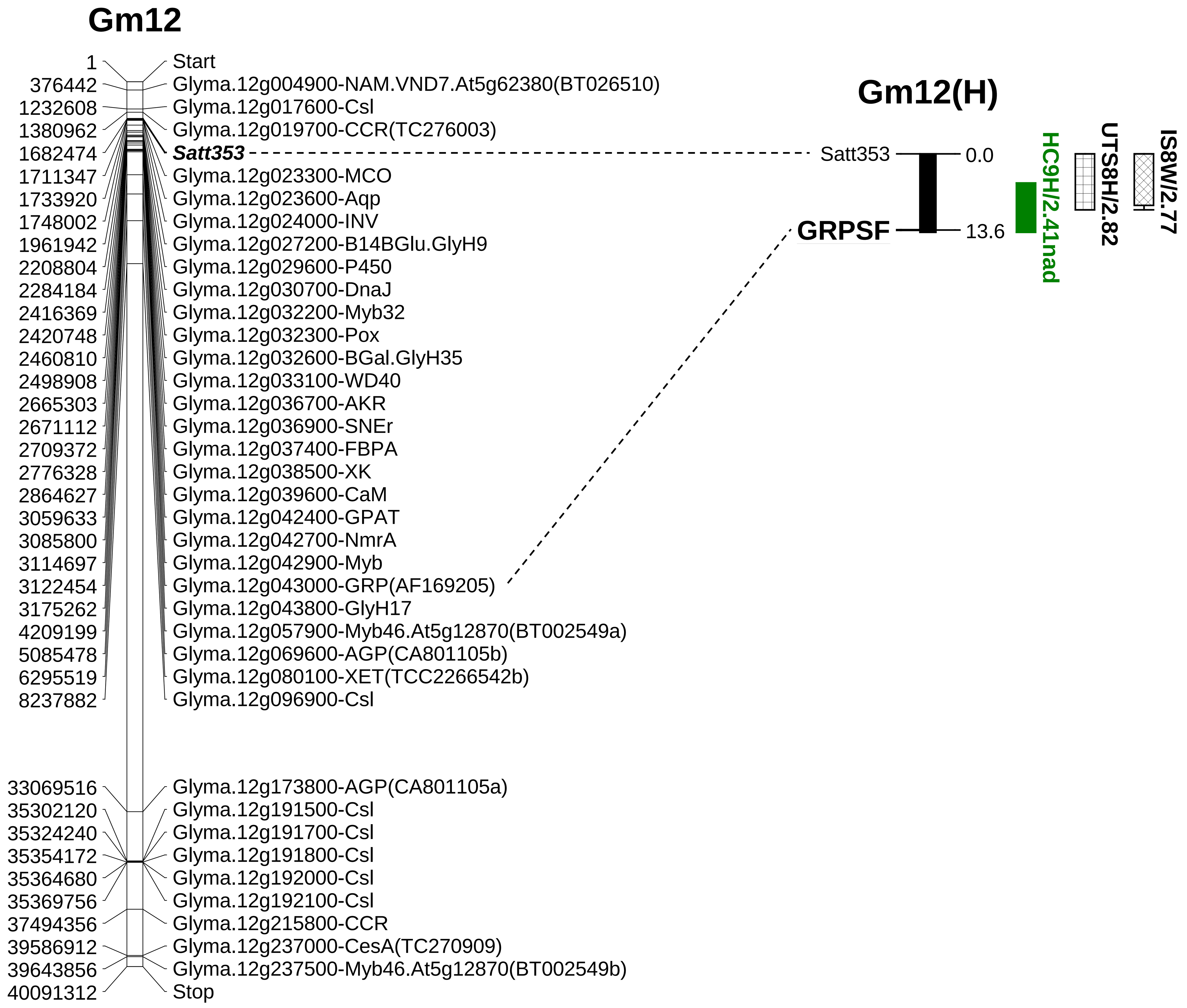

Gm12
1
376442
1232608
1380962
1682474
1711347
1733920
1748002
1961942
2208804
2284184
2416369
2420748
2460810
2498908
2665303
2671112
2709372
2776328
2864627
3059633
3085800
3114697
3122454
3175262
4209199
5085478
6295519
8237882
33069516
35302120
35324240
35354172
35364680
35369756
37494356
39586912
39643856
40091312
Start
Glyma.12g004900-NAM.VND7.At5g62380(BT026510)
Glyma.12g017600-Csl
Glyma.12g019700-CCR(TC276003)
Satt353
Glyma.12g023300-MCO
Glyma.12g023600-Aqp
Glyma.12g024000-INV
Glyma.12g027200-B14BGlu.GlyH9
Glyma.12g029600-P450
Glyma.12g030700-DnaJ
Glyma.12g032200-Myb32
Glyma.12g032300-Pox
Glyma.12g032600-BGal.GlyH35
Glyma.12g033100-WD40
Glyma.12g036700-AKR
Glyma.12g036900-SNEr
Glyma.12g037400-FBPA
Glyma.12g038500-XK
Glyma.12g039600-CaM
Glyma.12g042400-GPAT
Glyma.12g042700-NmrA
Glyma.12g042900-Myb
Glyma.12g043000-GRP(AF169205)
Glyma.12g043800-GlyH17
Glyma.12g057900-Myb46.At5g12870(BT002549a)
Glyma.12g069600-AGP(CA801105b)
Glyma.12g080100-XET(TCC2266542b)
Glyma.12g096900-Csl
Glyma.12g173800-AGP(CA801105a)
Glyma.12g191500-Csl
Glyma.12g191700-Csl
Glyma.12g191800-Csl
Glyma.12g192000-Csl
Glyma.12g192100-Csl
Glyma.12g215800-CCR
Glyma.12g237000-CesA(TC270909)
Glyma.12g237500-Myb46.At5g12870(BT002549b)
Stop
Gm12(H)
Satt353
0.0
GRPSF
13.6
HC9H/2.41nad
UTS8H/2.82
IS8W/2.77

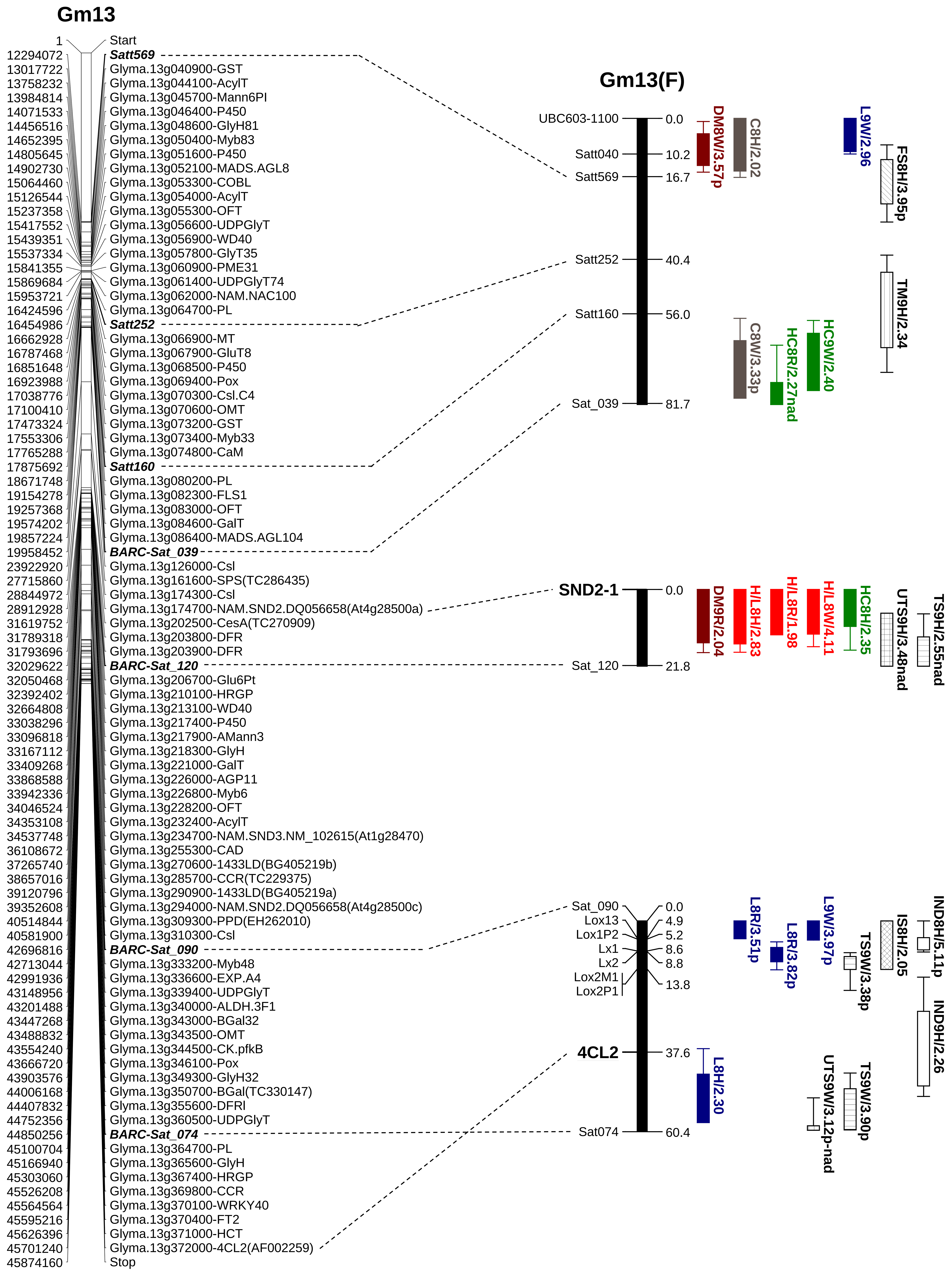

Gm13
1 Start
12294072 Satt569
13017722 Glyma.13g040900-GST
13758232 Glyma.13g044100-AcylT
13984814 Glyma.13g045700-Mann6PI
14071533 Glyma.13g046400-P450
14456516 Glyma.13g048600-GlyH81
14652395 Glyma.13g050400-Myb83
14805645 Glyma.13g051600-P450
14902730 Glyma.13g052100-MADS.AGL8
15064460 Glyma.13g053300-COBL
15126544 Glyma.13g054000-AcylT
15237358 Glyma.13g055300-OFT
15417552 Glyma.13g056600-UDPGlyT
15439351 Glyma.13g056900-WD40
15537334 Glyma.13g057800-GlyT35
15841355 Glyma.13g060900-PME31
15869684 Glyma.13g061400-UDPGlyT74
15953721 Glyma.13g062000-NAM.NAC100
16424596 Glyma.13g064700-PL
16454986 Satt252
16662928 Glyma.13g066900-MT
16787468 Glyma.13g067900-GluT8
16851648 Glyma.13g068500-P450
16923988 Glyma.13g069400-Pox
17038776 Glyma.13g070300-Csl.C4
17100410 Glyma.13g070600-OMT
17473324 Glyma.13g073200-GST
17553306 Glyma.13g073400-Myb33
17765288 Glyma.13g074800-CaM
17875692 Satt160
18671748 Glyma.13g080200-PL
19154278 Glyma.13g082300-FLS1
19257368 Glyma.13g083000-OFT
19574202 Glyma.13g084600-GalT
19857224 Glyma.13g086400-MADS.AGL104
19958452 BARC-Sat_039
23922920 Glyma.13g126000-Csl
27715860 Glyma.13g161600-SPS(TC286435)
28844972 Glyma.13g174300-Csl
28912928 Glyma.13g174700-NAM.SND2.DQ056658(At4g28500a)
31619752 Glyma.13g202500-CesA(TC270909)
31789318 Glyma.13g203800-DFR
31793696 Glyma.13g203900-DFR
32029622 BARC-Sat_120
32050468 Glyma.13g206700-Glu6Pt
32392402 Glyma.13g210100-HRGP
32664808 Glyma.13g213100-WD40
33038296 Glyma.13g217400-P450
33096818 Glyma.13g217900-AMann3
33167112 Glyma.13g218300-GlyH
33409268 Glyma.13g221000-GalT
33868588 Glyma.13g226000-AGP11
33942336 Glyma.13g226800-Myb6
34046524 Glyma.13g228200-OFT
34353108 Glyma.13g232400-AcylT
34537748 Glyma.13g234700-NAM.SND3.NM_102615(At1g28470)
36108672 Glyma.13g255300-CAD
37265740 Glyma.13g270600-1433LD(BG405219b)
38657016 Glyma.13g285700-CCR(TC229375)
39120796 Glyma.13g290900-1433LD(BG405219a)
39352608 Glyma.13g294000-NAM.SND2.DQ056658(At4g28500c)
40514844 Glyma.13g309300-PPD(EH262010)
40581900 Glyma.13g310300-Csl
42696816 BARC-Sat_090
42713064 Glyma.13g333200-Myb48
42991936 Glyma.13g336600-EXP.A4
43148956 Glyma.13g339400-UDPGlyT
43201488 Glyma.13g340000-ALDH.3F1
43447268 Glyma.13g343000-BGal32
43488832 Glyma.13g343500-OMT
43554240 Glyma.13g344500-CK.pfkB
43666720 Glyma.13g346100-Pox
43903576 Glyma.13g349300-GlyH32
44006168 Glyma.13g350700-BGal(TC330147)
44407832 Glyma.13g355600-DFRI
44752356 Glyma.13g360500-UDPGlyT
44850256 BARC-Sat_074
45100704 Glyma.13g364700-PL
45166940 Glyma.13g365600-GlyH
45303060 Glyma.13g367400-HRGP
45526208 Glyma.13g369800-CCR
45564564 Glyma.13g370100-WRKY40
45595216 Glyma.13g370400-FT2
45626396 Glyma.13g371000-HCT
45701240 Glyma.13g372000-4CL2(AF002259)
45874160 Stop
Gm13(F)
UBC603-1100 0.0
Satt040 10.2
Satt569 16.7
Satt252 40.4
Satt160 56.0
Sat_039 81.7
DM8W/3.57p
C8H/2.02
L9W/2.96
FS8H/3.95p
TM9H/2.34
C8W/3.33p
HC8R/2.27nad
HC9W/2.40
SND2-1 0.0
Sat_120 21.8
DM9R/2.04
H/L8H/2.83
H/L8R/1.98
H/L8W/4.11
HC8H/2.35
UTS9H/3.48nad
TS9H/2.55nad
Sat_090 0.0
Lox13 4.9
Lox1P2 5.2
Lx1 8.6
Lx2 8.8
Lox2M1 13.8
Lox2P1
4CL2 37.6
Sat074 60.4
L8R/3.51p
L8R/3.82p
L9W/3.97p
TS9W/3.38p
IS8H/2.05
IND8H/5.11p
IND9H/2.26
L8H/2.30
UTS9W/3.12p-nad
TS9W/3.90p

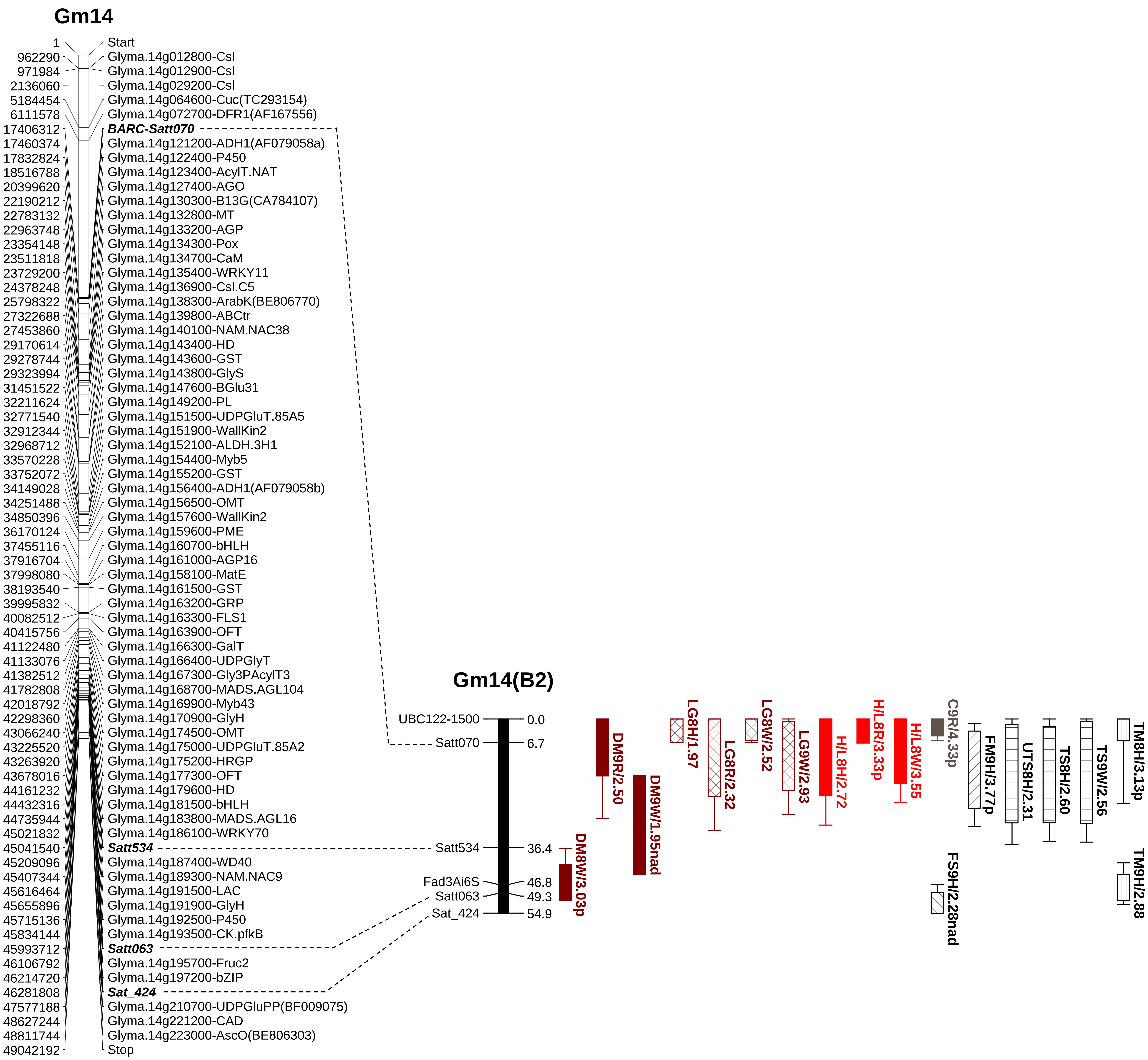

Gm14
1 Start
962290 Glyma.14g012800-Csl
971984 Glyma.14g012900-Csl
2136060 Glyma.14g029200-Csl
5184454 Glyma.14g064600-Cuc(TC293154)
6111578 Glyma.14g072700-DFR1(AF167556)
17406312 BARC-Satt070
17460374 Glyma.14g121200-ADH1(AF079058a)
17832824 Glyma.14g122400-P450
18516788 Glyma.14g123400-AcylT.NAT
20399620 Glyma.14g127400-AGO
22190212 Glyma.14g130300-B13G(CA784107)
22783132 Glyma.14g132800-MT
22963748 Glyma.14g133200-AGP
23354148 Glyma.14g134300-Pox
23511818 Glyma.14g134700-CaM
23729200 Glyma.14g135400-WRKY11
24378248 Glyma.14g136900-Csl.C5
25798322 Glyma.14g138300-ArabK(BE806770)
27322688 Glyma.14g139800-ABCtr
27453860 Glyma.14g140100-NAM.NAC38
29170614 Glyma.14g143400-HD
29278744 Glyma.14g143600-GST
29323994 Glyma.14g143800-GlyS
31451522 Glyma.14g147600-BGlu31
32211624 Glyma.14g149200-PL
32771540 Glyma.14g151500-UDPGluT.85A5
32912344 Glyma.14g151900-WallKin2
32968712 Glyma.14g152100-ALDH.3H1
33570228 Glyma.14g154400-Myb5
33752072 Glyma.14g155200-GST
34149028 Glyma.14g156400-ADH1(AF079058b)
34251488 Glyma.14g156500-OMT
34850396 Glyma.14g157600-WallKin2
36170124 Glyma.14g159600-PME
37455116 Glyma.14g160700-bHLH
37916704 Glyma.14g161000-AGP16
37998080 Glyma.14g158100-MatE
38193540 Glyma.14g161500-GST
39995832 Glyma.14g163200-GRP
40082512 Glyma.14g163300-FLS1
40415756 Glyma.14g163900-OFT
41122480 Glyma.14g166300-GalT
41133076 Glyma.14g166400-UDPGlyT
41382512 Glyma.14g167300-Gly3PAcylT3
41782808 Glyma.14g168700-MADS.AGL104
42018792 Glyma.14g169900-Myb43
42298360 Glyma.14g170900-GlyH
43066240 Glyma.14g174500-OMT
43225520 Glyma.14g175000-UDPGluT.85A2
43263920 Glyma.14g175200-HRGP
43678016 Glyma.14g177300-OFT
44161232 Glyma.14g179600-HD
44432316 Glyma.14g181500-bHLH
44735944 Glyma.14g183800-MADS.AGL16
45021832 Glyma.14g186100-WRKY70
45041540 Satt534
45209096 Glyma.14g187400-WD40
45407344 Glyma.14g189300-NAM.NAC9
45616464 Glyma.14g191500-LAC
45655896 Glyma.14g191900-GlyH
45715136 Glyma.14g192500-P450
45834144 Glyma.14g193500-CK.pfkB
45993712 Satt063
46106792 Glyma.14g195700-Fruc2
46214720 Glyma.14g197200-bZIP
46281808 Sat_424
47577188 Glyma.14g210700-UDPGluPP(BF009075)
48627244 Glyma.14g221200-CAD
48811744 Glyma.14g223000-AscO(BE806303)
49042192 Stop
Gm14(B2)
UBC122-1500 0.0
Satt070 6.7
Satt534 36.4
Fad3Ai6S 46.8
Satt063 49.3
Sat_424 54.9
DM9R/2.50
DM9W/1.95nad
DM8W/3.03p
LG8H/1.97
LG8R/2.32
LG8W/2.52
LG9W/2.93
H/L8H/2.72
H/L8R/3.33p
H/L8W/3.55
C9R/4.33p
FM9H/3.77p
FS9H/2.28nad
UTS8H/2.31
TS8H/2.60
TS9W/2.56
TM8H/3.13p
TM9H/2.88

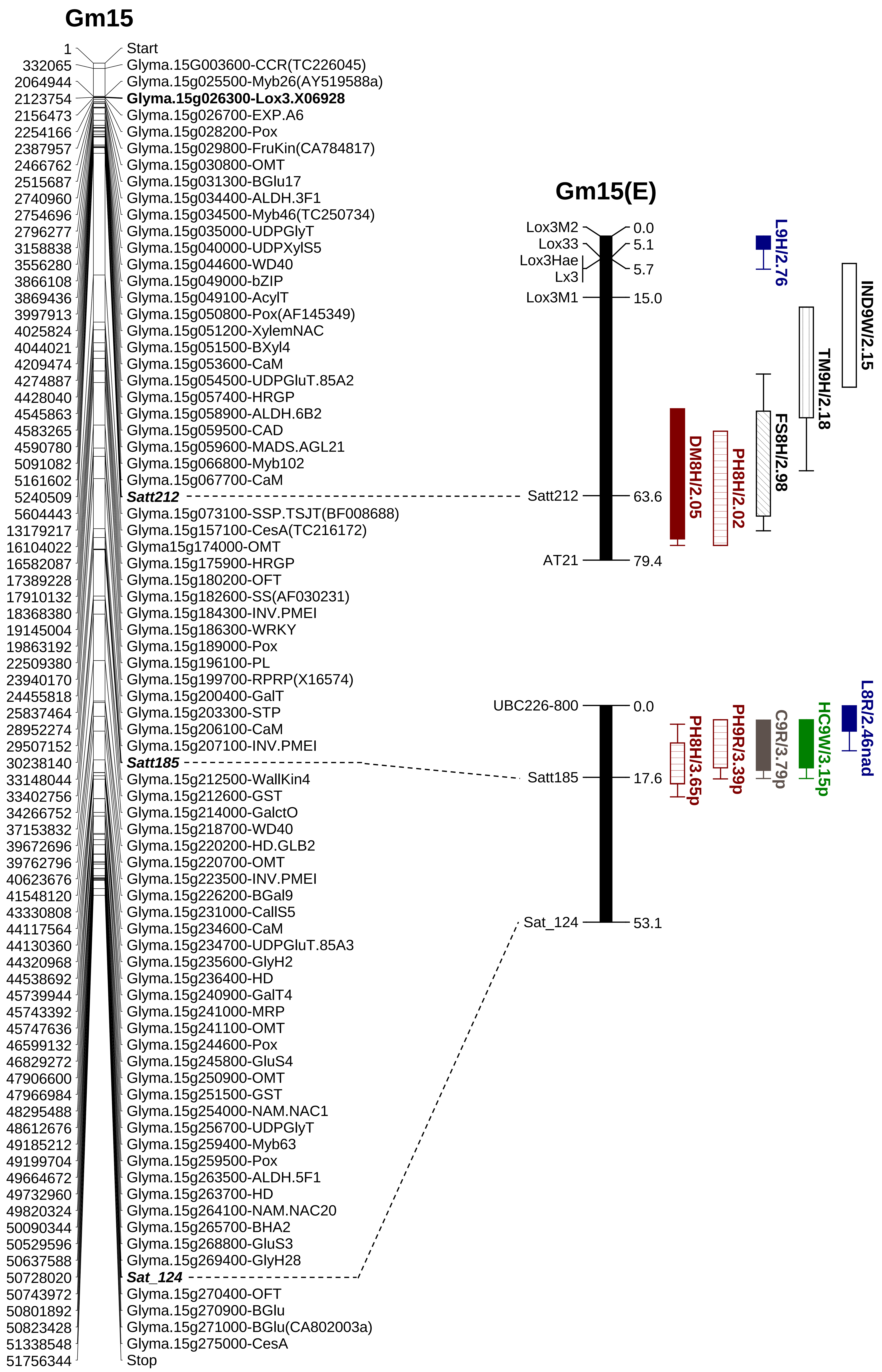

Gm15
1 Start
332065 Glyma.15G003600-CCR(TC226045)
2064944 Glyma.15g025500-Myb26(AY519588a)
2123754 Glyma.15g026300-Lox3.X06928
2156473 Glyma.15g026700-EXP.A6
2254166 Glyma.15g028200-Pox
2387957 Glyma.15g029800-FruKin(CA784817)
2466762 Glyma.15g030800-OMT
2515687 Glyma.15g031300-BGlu17
2740960 Glyma.15g034400-ALDH.3F1
2754696 Glyma.15g034500-Myb46(TC250734)
2796277 Glyma.15g035000-UDPGlyT
3158838 Glyma.15g040000-UDPXylS5
3556280 Glyma.15g044600-WD40
3866108 Glyma.15g049000-bZIP
3869436 Glyma.15g049100-AcylT
3997913 Glyma.15g050800-Pox(AF145349)
4025824 Glyma.15g051200-XylemNAC
4044021 Glyma.15g051500-BXyl4
4209474 Glyma.15g053600-CaM
4274887 Glyma.15g054500-UDPGluT.85A2
4428040 Glyma.15g057400-HRGP
4545863 Glyma.15g058900-ALDH.6B2
4583265 Glyma.15g059500-CAD
4590780 Glyma.15g059600-MADS.AGL21
5091082 Glyma.15g066800-Myb102
5161602 Glyma.15g067700-CaM
5240509 Satt212
5604443 Glyma.15g073100-SSP.TSJT(BF008688)
13179217 Glyma.15g157100-CesA(TC216172)
16104022 Glyma15g174000-OMT
16582087 Glyma.15g175900-HRGP
17389228 Glyma.15g180200-OFT
17910132 Glyma.15g182600-SS(AF030231)
18368380 Glyma.15g184300-INV.PMEI
19145004 Glyma.15g186300-WRKY
19863192 Glyma.15g189000-Pox
22509380 Glyma.15g196100-PL
23940170 Glyma.15g199700-RPRP(X16574)
24455818 Glyma.15g200400-GalT
25837464 Glyma.15g203300-STP
28952274 Glyma.15g206100-CaM
29507152 Glyma.15g207100-INV.PMEI
30238140 Satt185
33148044 Glyma.15g212500-WallKin4
33402756 Glyma.15g212600-GST
34266752 Glyma.15g214000-GalctO
37153832 Glyma.15g218700-WD40
39672696 Glyma.15g220200-HD.GLB2
39762796 Glyma.15g220700-OMT
40623676 Glyma.15g223500-INV.PMEI
41548120 Glyma.15g226200-BGal9
43330808 Glyma.15g231000-CallS5
44117564 Glyma.15g234600-CaM
44130360 Glyma.15g234700-UDPGluT.85A3
44320968 Glyma.15g235600-GlyH2
44538692 Glyma.15g236400-HD
45739944 Glyma.15g240900-GalT4
45743392 Glyma.15g241000-MRP
45747636 Glyma.15g241100-OMT
46599132 Glyma.15g244600-Pox
46829272 Glyma.15g245800-GluS4
47906600 Glyma.15g250900-OMT
47966984 Glyma.15g251500-GST
48295488 Glyma.15g254000-NAM.NAC1
48612676 Glyma.15g256700-UDPGlyT
49185212 Glyma.15g259400-Myb63
49199704 Glyma.15g259500-Pox
49664672 Glyma.15g263500-ALDH.5F1
49732960 Glyma.15g263700-HD
49820324 Glyma.15g264100-NAM.NAC20
50090344 Glyma.15g265700-BHA2
50529596 Glyma.15g268800-GluS3
50637588 Glyma.15g269400-GlyH28
50728020 Sat_124
50743972 Glyma.15g270400-OFT
50801892 Glyma.15g270900-BGlu
50823428 Glyma.15g271000-BGlu(CA802003a)
51338548 Glyma.15g275000-CesA
51756344 Stop
Gm15(E)
Lox3M2 0.0
Lox33 5.1
Lox3Hae
Lx3 5.7
Lox3M1 15.0
Satt212 63.6
AT21 79.4
L9H/2.76
IND9W/2.15
TM9H/2.18
FS8H/2.98
PH8H/2.02
DM8H/2.05
UBC226-800 0.0
Satt185 17.6
Sat_124 53.1
L8R/2.46nad
HC9W/3.15p
C9R/3.79p
PH9R/3.39p
PH8H/3.65p

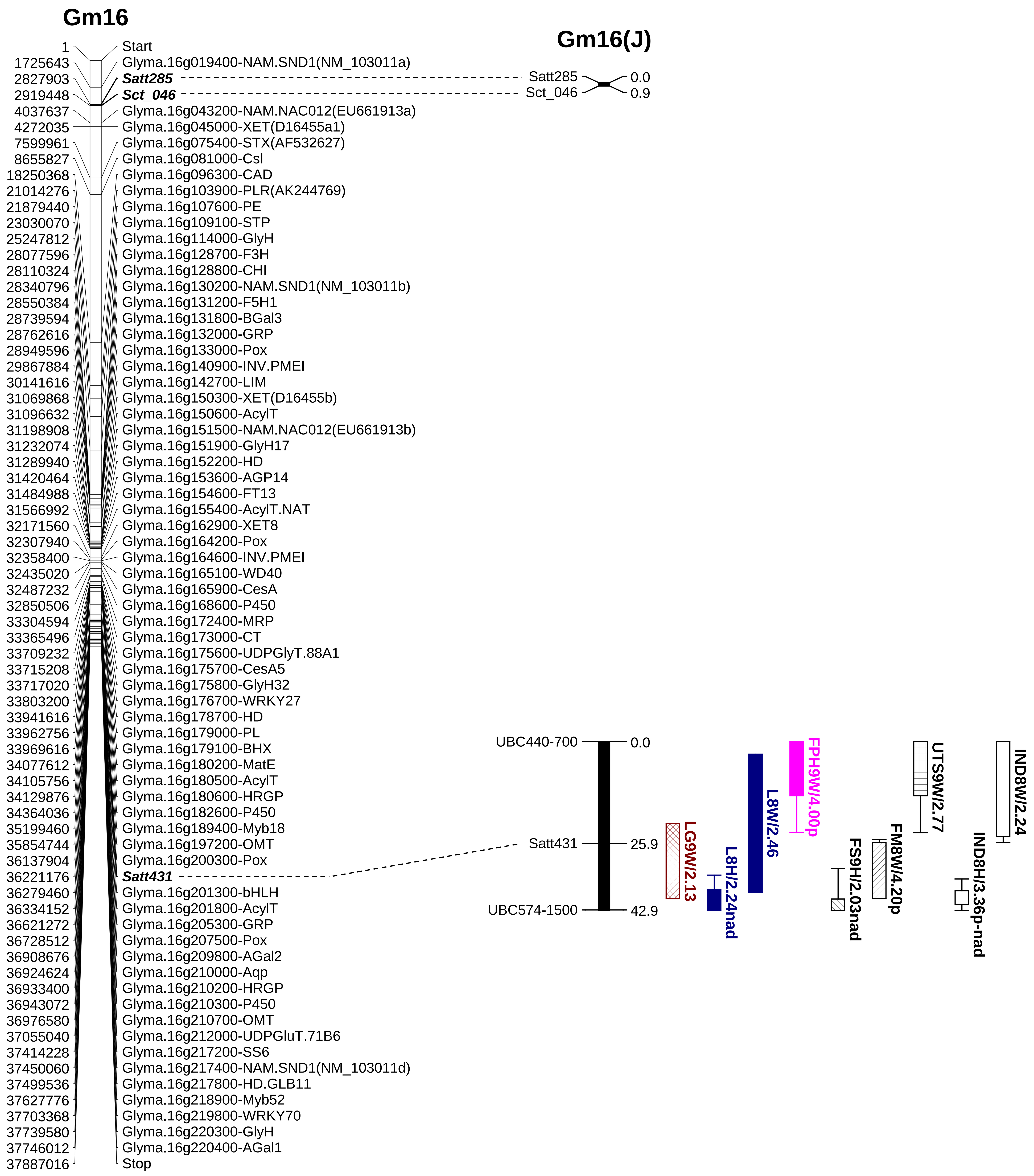

Gm16
Gm16(J)
1 Start
1725643 Glyma.16g019400-NAM.SND1(NM_103011a)
2827903 Satt285
2919448 Sct_046
4037637 Glyma.16g043200-NAM.NAC012(EU661913a)
4272035 Glyma.16g045000-XET(D16455a1)
7599961 Glyma.16g075400-STX(AF532627)
8655827 Glyma.16g081000-Csl
18250368 Glyma.16g096300-CAD
21014276 Glyma.16g103900-PLR(AK244769)
21879440 Glyma.16g107600-PE
23030070 Glyma.16g109100-STP
25247812 Glyma.16g114000-GlyH
28077596 Glyma.16g128700-F3H
28110324 Glyma.16g128800-CHI
28340796 Glyma.16g130200-NAM.SND1(NM_103011b)
28550384 Glyma.16g131200-F5H1
28739594 Glyma.16g131800-BGal3
28762616 Glyma.16g132000-GRP
28949596 Glyma.16g133000-Pox
29867884 Glyma.16g140900-INV.PMEI
30141616 Glyma.16g142700-LIM
31069868 Glyma.16g150300-XET(D16455b)
31096632 Glyma.16g150600-AcylT
31198908 Glyma.16g151500-NAM.NAC012(EU661913b)
31232074 Glyma.16g151900-GlyH17
31289940 Glyma.16g152200-HD
31420464 Glyma.16g153600-AGP14
31484988 Glyma.16g154600-FT13
31566992 Glyma.16g155400-AcylT.NAT
32171560 Glyma.16g162900-XET8
32307940 Glyma.16g164200-Pox
32358400 Glyma.16g164600-INV.PMEI
32435020 Glyma.16g165100-WD40
32487232 Glyma.16g165900-CesA
32850506 Glyma.16g168600-P450
33304594 Glyma.16g172400-MRP
33365496 Glyma.16g173000-CT
33709232 Glyma.16g175600-UDPGlyT.88A1
33715208 Glyma.16g175700-CesA5
33717020 Glyma.16g175800-GlyH32
33803200 Glyma.16g176700-WRKY27
33941616 Glyma.16g178700-HD
33962756 Glyma.16g179000-PL
33969616 Glyma.16g179100-BHX
34077612 Glyma.16g180200-MatE
34105756 Glyma.16g180500-AcylT
34129876 Glyma.16g180600-HRGP
34364036 Glyma.16g182600-P450
35199460 Glyma.16g189400-Myb18
35854744 Glyma.16g197200-OMT
36137904 Glyma.16g200300-Pox
36221176 Satt431
36279460 Glyma.16g201300-bHLH
36334152 Glyma.16g201800-AcylT
36621272 Glyma.16g205300-GRP
36728512 Glyma.16g207500-Pox
36908676 Glyma.16g209800-AGal2
36924624 Glyma.16g210000-Aqp
36933400 Glyma.16g210200-HRGP
36943072 Glyma.16g210300-P450
36976580 Glyma.16g210700-OMT
37055040 Glyma.16g212000-UDPGluT.71B6
37414228 Glyma.16g217200-SS6
37450060 Glyma.16g217400-NAM.SND1(NM_103011d)
37499536 Glyma.16g217800-HD.GLB11
37627776 Glyma.16g218900-Myb52
37703368 Glyma.16g219800-WRKY70
37739580 Glyma.16g220300-GlyH
37746012 Glyma.16g220400-AGal1
37887016 Stop
Satt285 0.0
Sct_046 0.9
UBC440-700 0.0
Satt431 25.9
UBC574-1500 42.9
LG9W/2.13
L8H/2.24nad
L8W/2.46
FPH9W/4.00p
FS9H/2.03nad
FM8W/4.20p
UTS9W/2.77
IND8H/3.36p-nad
IND8W/2.24

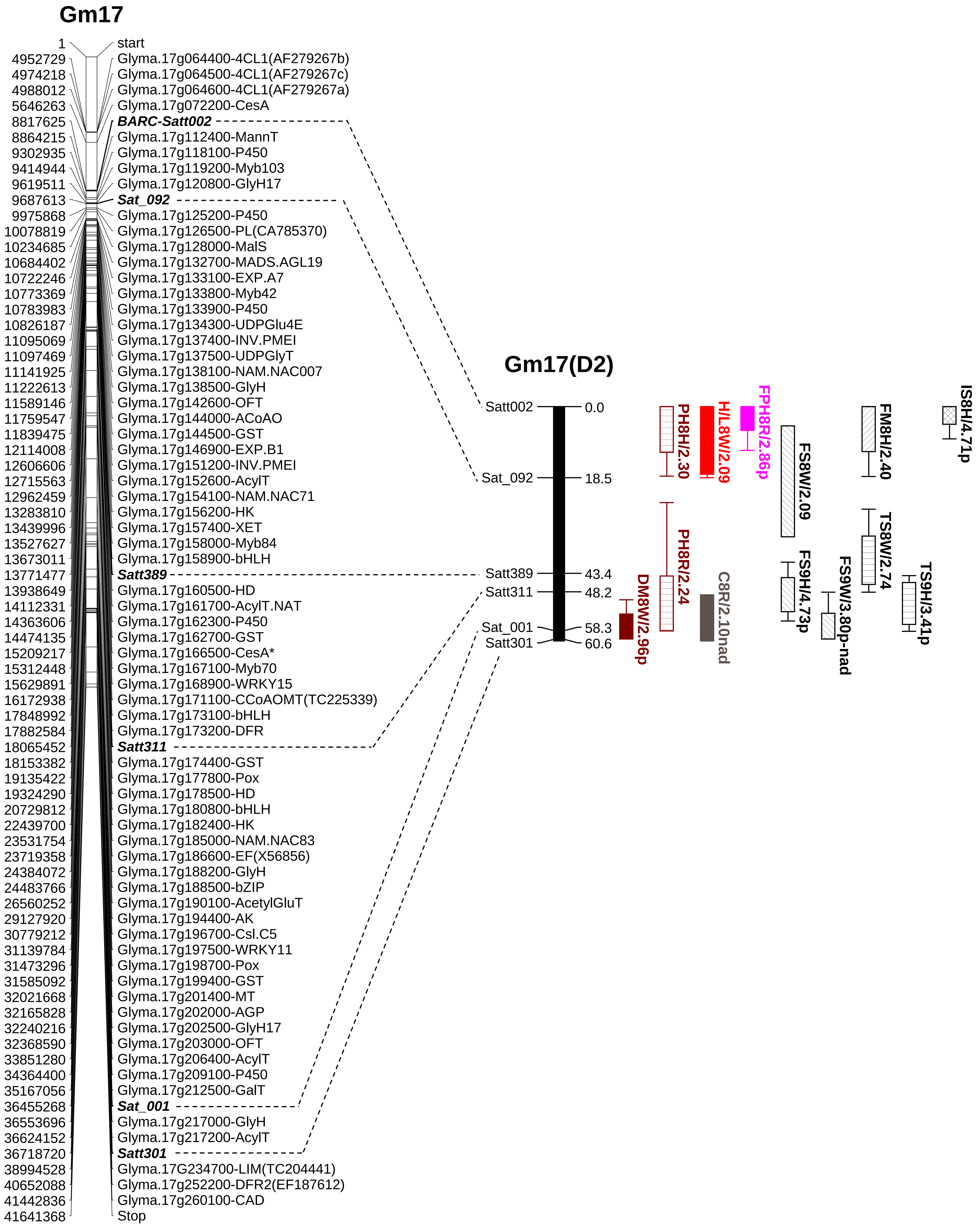
Gm17
1 start
4952729 Glyma.17g064400-4CL1(AF279267b)
4974218 Glyma.17g064500-4CL1(AF279267c)
4988012 Glyma.17g064600-4CL1(AF279267a)
5646263 Glyma.17g072200-CesA
8817625 BARC-Satt002
8864215 Glyma.17g112400-MannT
9302935 Glyma.17g118100-P450
9414944 Glyma.17g119200-Myb103
9619511 Glyma.17g120800-GlyH17
9687613 Sat_092
9975868 Glyma.17g125200-P450
10078819 Glyma.17g126500-PL(CA785370)
10234685 Glyma.17g128000-MalS
10684402 Glyma.17g132700-MADS.AGL19
10722246 Glyma.17g133100-EXP.A7
10773369 Glyma.17g133800-Myb42
10783983 Glyma.17g133900-P450
10826187 Glyma.17g134300-UDPGlu4E
11095069 Glyma.17g137400-INV.PMEI
11097469 Glyma.17g137500-UDPGlyT
11141925 Glyma.17g138100-NAM.NAC007
11222613 Glyma.17g138500-GlyH
11589146 Glyma.17g142600-OFT
11759547 Glyma.17g144000-ACoAO
11839475 Glyma.17g144500-GST
12114008 Glyma.17g146900-EXP.B1
12606606 Glyma.17g151200-INV.PMEI
12715563 Glyma.17g152600-AcylT
12962459 Glyma.17g154100-NAM.NAC71
13283810 Glyma.17g156200-HK
13439996 Glyma.17g157400-XET
13527627 Glyma.17g158000-Myb84
13673011 Glyma.17g158900-bHLH
13771477 Satt389
13938649 Glyma.17g160500-HD
14112331 Glyma.17g161700-AcylT.NAT
14363606 Glyma.17g162300-P450
14474135 Glyma.17g162700-GST
15209217 Glyma.17g166500-CesA*
15312448 Glyma.17g167100-Myb70
15629891 Glyma.17g168900-WRKY15
16172938 Glyma.17g171100-CCoAOMT(TC225339)
17848992 Glyma.17g173100-bHLH
17882584 Glyma.17g173200-DFR
18065452 Satt311
18153382 Glyma.17g174400-GST
19135422 Glyma.17g177800-Pox
19324290 Glyma.17g178500-HD
20729812 Glyma.17g180800-bHLH
22439700 Glyma.17g182400-HK
23531754 Glyma.17g185000-NAM.NAC83
23719358 Glyma.17g186600-EF(X56856)
24384072 Glyma.17g188200-GlyH
24483766 Glyma.17g188500-bZIP
26560252 Glyma.17g190100-AcetylGluT
29127920 Glyma.17g194400-AK
30779212 Glyma.17g196700-Csl.C5
31139784 Glyma.17g197500-WRKY11
31473296 Glyma.17g198700-Pox
31585092 Glyma.17g199400-GST
32021668 Glyma.17g201400-MT
32165828 Glyma.17g202000-AGP
32240216 Glyma.17g202500-GlyH17
32368590 Glyma.17g203000-OFT
33851280 Glyma.17g206400-AcylT
34364400 Glyma.17g209100-P450
35167056 Glyma.17g212500-GalT
36455268 Sat_001
36553696 Glyma.17g217000-GlyH
36624152 Glyma.17g217200-AcylT
36718720 Satt301
38994528 Glyma.17G234700-LIM(TC204441)
40652088 Glyma.17g252200-DFR2(EF187612)
41442836 Glyma.17g260100-CAD
41641368 Stop
Gm17(D2)
Satt002 0.0
Sat_092 18.5
Satt389 43.4
Satt311 48.2
Sat_001 58.3
Satt301 60.6
PH8H/2.30
H/L8W/2.09
FPH8R/2.86p
FS8W/2.09
FM8H/2.40
IS8H/4.71p
PH8R/2.24
C8R/2.10nad
DM8W/2.96p
FS9H/4.73p
FS9W/3.80p-nad
TS8W/2.74
TS9H/3.41p

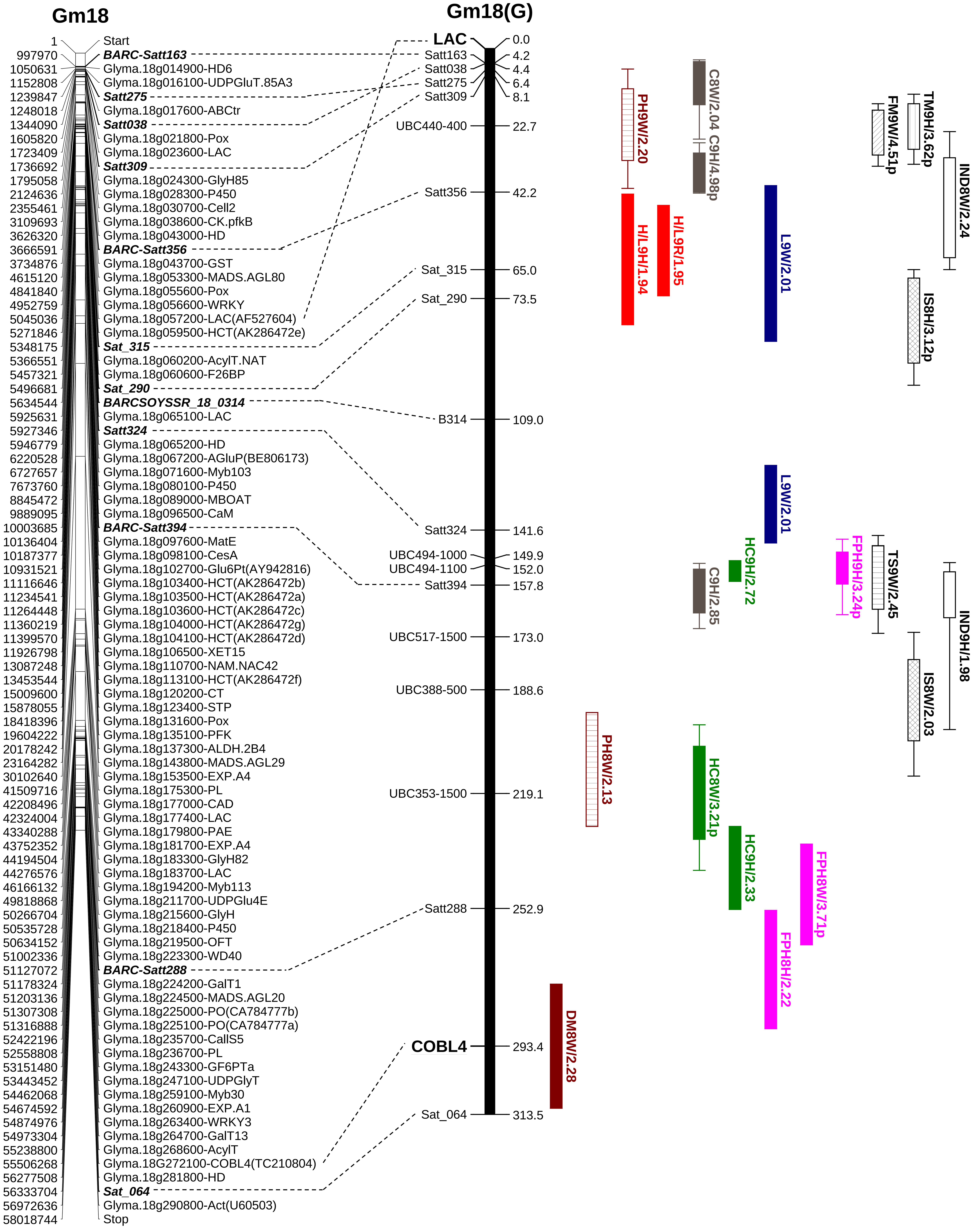

Gm18
Gm18(G)
1 Start
997970 BARC-Satt163
1050631 Glyma.18g014900-HD6
1152808 Glyma.18g016100-UDPGluT.85A3
1239847 Satt275
1248018 Glyma.18g017600-ABCtr
1344090 Satt038
1605820 Glyma.18g021800-Pox
1723409 Glyma.18g023600-LAC
1736692 Satt309
1795058 Glyma.18g024300-GlyH85
2124636 Glyma.18g028300-P450
2355461 Glyma.18g030700-Cell2
3109693 Glyma.18g038600-CK.pfkB
3626320 Glyma.18g043000-HD
3666591 BARC-Satt356
3734876 Glyma.18g043700-GST
4615120 Glyma.18g053300-MADS.AGL80
4841840 Glyma.18g055600-Pox
4952759 Glyma.18g056600-WRKY
5045036 Glyma.18g057200-LAC(AF527604)
5271846 Glyma.18g059500-HCT(AK286472e)
5348175 Sat_315
5366551 Glyma.18g060200-AcylT.NAT
5457321 Glyma.18g060600-F26BP
5496681 Sat_290
5634544 BARCSOYSSR_18_0314
5925631 Glyma.18g065100-LAC
5927346 Satt324
5946779 Glyma.18g065200-HD
6220528 Glyma.18g067200-AGluP(BE806173)
6727657 Glyma.18g071600-Myb103
7673760 Glyma.18g080100-P450
8845472 Glyma.18g089000-MBOAT
9889095 Glyma.18g096500-CaM
10003685 BARC-Satt394
10136404 Glyma.18g097600-MatE
10187377 Glyma.18g098100-CesA
10931521 Glyma.18g102700-Glu6Pt(AY942816)
11116646 Glyma.18g103400-HCT(AK286472b)
11234541 Glyma.18g103500-HCT(AK286472a)
11264448 Glyma.18g103600-HCT(AK286472c)
11360219 Glyma.18g104000-HCT(AK286472g)
11399570 Glyma.18g104100-HCT(AK286472d)
11926798 Glyma.18g106500-XET15
13087248 Glyma.18g110700-NAM.NAC42
13453544 Glyma.18g113100-HCT(AK286472f)
15009600 Glyma.18g120200-CT
15878055 Glyma.18g123400-STP
18418396 Glyma.18g131600-Pox
19604222 Glyma.18g135100-PFK
20178242 Glyma.18g137300-ALDH.2B4
23164282 Glyma.18g143800-MADS.AGL29
30102640 Glyma.18g153500-EXP.A4
41509716 Glyma.18g175300-PL
42208496 Glyma.18g177000-CAD
42324004 Glyma.18g177400-LAC
43340288 Glyma.18g179800-PAE
43752352 Glyma.18g181700-EXP.A4
44194504 Glyma.18g183300-GlyH82
44276576 Glyma.18g183700-LAC
46166132 Glyma.18g194200-Myb113
49818868 Glyma.18g211700-UDPGlu4E
50266704 Glyma.18g215600-GlyH
50535728 Glyma.18g218400-P450
50634152 Glyma.18g219500-OFT
51002336 Glyma.18g223300-WD40
51127072 BARC-Satt288
51178324 Glyma.18g224200-GalT1
51203136 Glyma.18g224500-MADS.AGL20
51307308 Glyma.18g225000-PO(CA784777b)
51316888 Glyma.18g225100-PO(CA784777a)
52422196 Glyma.18g235700-CallS5
52558808 Glyma.18g236700-PL
53151480 Glyma.18g243300-GF6PTa
53443452 Glyma.18g247100-UDPGlyT
54462068 Glyma.18g259100-Myb30
54674592 Glyma.18g260900-EXP.A1
54874976 Glyma.18g263400-WRKY3
54973304 Glyma.18g264700-GalT13
55238800 Glyma.18g268600-AcylT
55506268 Glyma.18G272100-COBL4(TC210804)
56277508 Glyma.18g281800-HD
56333704 Sat_064
56972636 Glyma.18g290800-Act(U60503)
58018744 Stop
LAC 0.0
Satt163 4.2
Satt038 4.4
Satt275 6.4
Satt309 8.1
UBC440-400 22.7
Satt356 42.2
Sat_315 65.0
Sat_290 73.5
B314 109.0
Satt324 141.6
UBC494-1000 149.9
UBC494-1100 152.0
Satt394 157.8
UBC517-1500 173.0
UBC388-500 188.6
UBC353-1500 219.1
Satt288 252.9
COBL4 293.4
Sat_064 313.5
PH9W/2.20
C8W/2.04
C9H/4.98p
FM9W/4.51p
TM9H/3.62p
IND8W/2.24
H/L9R/1.95
H/L9H/1.94
L9W/2.01
IS8H/3.12p
L9W/2.01
HC9H/2.72
FPH9H/3.24p
TS9W/2.45
C9H/2.85
IND9H/1.98
IS8W/2.03
PH8W/2.13
HC8W/3.21p
HC9H/2.33
FPH8W/3.71p
FPH8H/2.22
DM8W/2.28

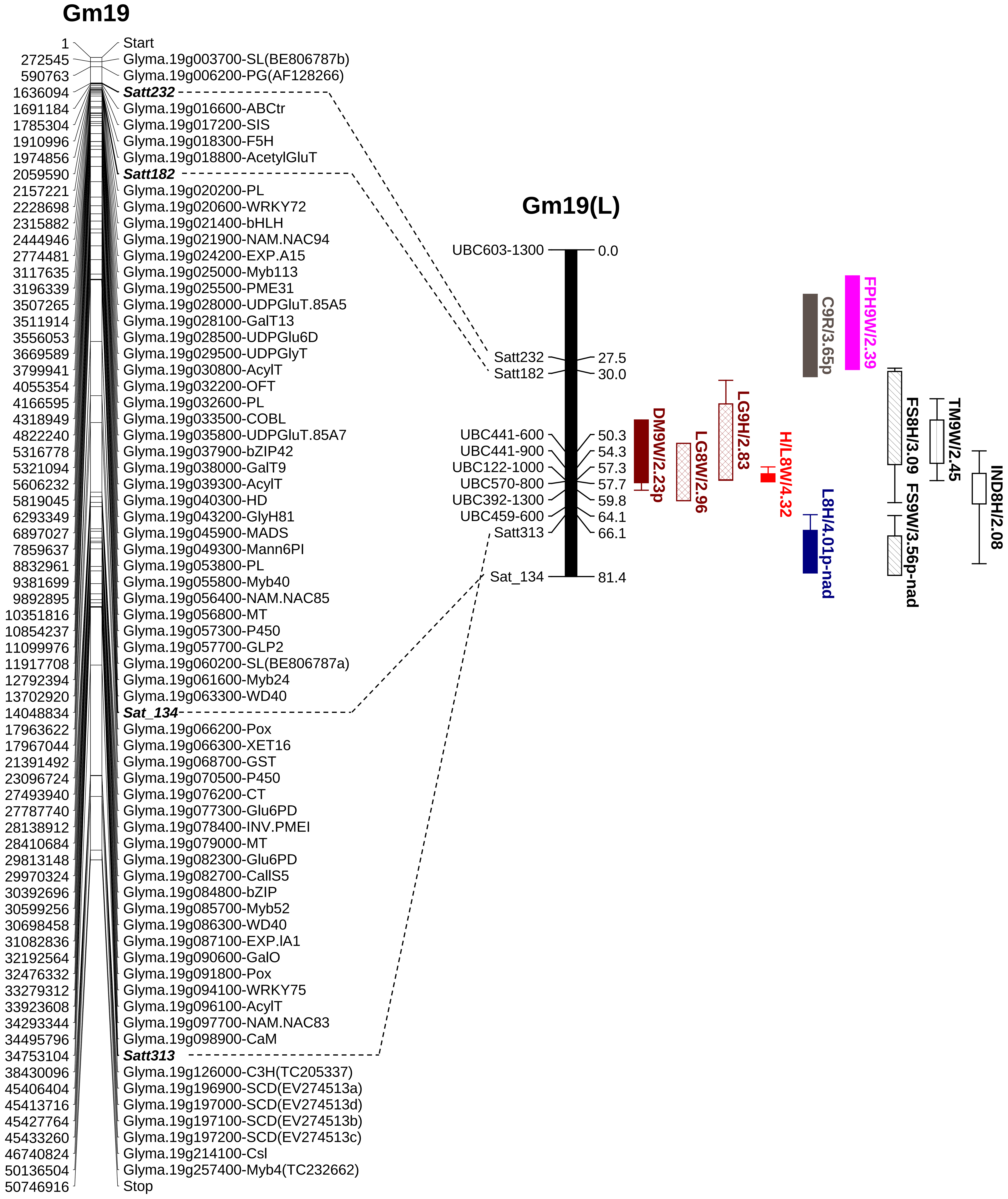
Gm19
1 Start
272545 Glyma.19g003700-SL(BE806787b)
590763 Glyma.19g006200-PG(AF128266)
1636094 Satt232
1691184 Glyma.19g016600-ABCtr
1785304 Glyma.19g017200-SIS
1910996 Glyma.19g018300-F5H
1974856 Glyma.19g018800-AcetylGluT
2059590 Satt182
2157221 Glyma.19g020200-PL
2228698 Glyma.19g020600-WRKY72
2315882 Glyma.19g021400-bHLH
2444946 Glyma.19g021900-NAM.NAC94
2774481 Glyma.19g024200-EXP.A15
3117635 Glyma.19g025000-Myb113
3196339 Glyma.19g025500-PME31
3507265 Glyma.19g028000-UDPGluT.85A5
3511914 Glyma.19g028100-GalT13
3556053 Glyma.19g028500-UDPGlu6D
3669589 Glyma.19g029500-UDPGlyT
3799941 Glyma.19g030800-AcylT
4055354 Glyma.19g032200-OFT
4166595 Glyma.19g032600-PL
4318949 Glyma.19g033500-COBL
4822240 Glyma.19g035800-UDPGluT.85A7
5316778 Glyma.19g037900-bZIP42
5321094 Glyma.19g038000-GalT9
5606232 Glyma.19g039300-AcylT
5819045 Glyma.19g040300-HD
6293349 Glyma.19g043200-GlyH81
6897027 Glyma.19g045900-MADS
7859637 Glyma.19g049300-Mann6PI
8832961 Glyma.19g053800-PL
9381699 Glyma.19g055800-Myb40
9892895 Glyma.19g056400-NAM.NAC85
10351816 Glyma.19g056800-MT
10854237 Glyma.19g057300-P450
11099976 Glyma.19g057700-GLP2
11917708 Glyma.19g060200-SL(BE806787a)
12792394 Glyma.19g061600-Myb24
13702920 Glyma.19g063300-WD40
14048834 Sat_134
17963622 Glyma.19g066200-Pox
17967044 Glyma.19g066300-XET16
21391492 Glyma.19g068700-GST
23096724 Glyma.19g070500-P450
27493940 Glyma.19g076200-CT
27787740 Glyma.19g077300-Glu6PD
28138912 Glyma.19g078400-INV.PMEI
28410684 Glyma.19g079000-MT
29813148 Glyma.19g082300-Glu6PD
29970324 Glyma.19g082700-CallS5
30392696 Glyma.19g084800-bZIP
30599256 Glyma.19g085700-Myb52
30698458 Glyma.19g086300-WD40
31082836 Glyma.19g087100-EXP.lA1
32192564 Glyma.19g090600-GalO
32476332 Glyma.19g091800-Pox
33279312 Glyma.19g094100-WRKY75
33923608 Glyma.19g096100-AcylT
34293344 Glyma.19g097700-NAM.NAC83
34495796 Glyma.19g098900-CaM
34753104 Satt313
38430096 Glyma.19g126000-C3H(TC205337)
45406404 Glyma.19g196900-SCD(EV274513a)
45413716 Glyma.19g197000-SCD(EV274513d)
45427764 Glyma.19g197100-SCD(EV274513b)
45433260 Glyma.19g197200-SCD(EV274513c)
46740824 Glyma.19g214100-Csl
50136504 Glyma.19g257400-Myb4(TC232662)
50746916 Stop
Gm19(L)
UBC603-1300 0.0
Satt232 27.5
Satt182 30.0
UBC441-600 50.3
UBC441-900 54.3
UBC122-1000 57.3
UBC570-800 57.7
UBC392-1300 59.8
UBC459-600 64.1
Satt313 66.1
Sat_134 81.4
FPH9W/2.39
C9R/3.65p
DM9W/2.23p
LG8W/2.96
LG9H/2.83
H/L8W/4.32
L8H/4.01p-nad
FS8H/3.09
FS9W/3.56p-nad
TM9W/2.45
IND8H/2.08

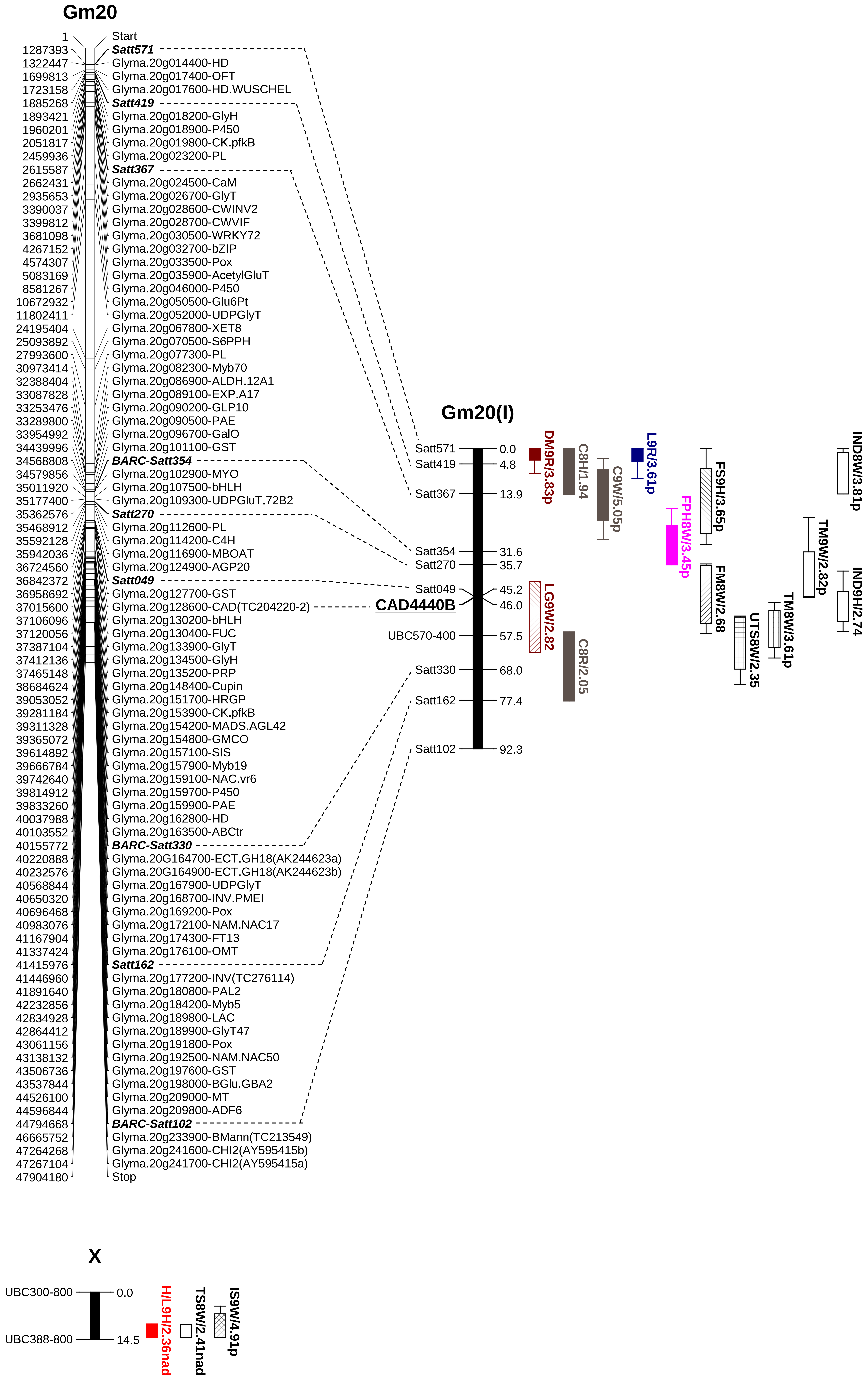
Gm20
1 Start
1287393 Satt571
1322447 Glyma.20g014400-HD
1699813 Glyma.20g017400-OFT
1723158 Glyma.20g017600-HD.WUSCHEL
1885268 Satt419
1893421 Glyma.20g018200-GlyH
1960201 Glyma.20g018900-P450
2051817 Glyma.20g019800-CK.pfkB
2459936 Glyma.20g023200-PL
2615587 Satt367
2662431 Glyma.20g024500-CaM
2935653 Glyma.20g026700-GlyT
3390037 Glyma.20g028600-CWINV2
3399812 Glyma.20g028700-CWVIF
3681098 Glyma.20g030500-WRKY72
4267152 Glyma.20g032700-bZIP
4574307 Glyma.20g033500-Pox
5083169 Glyma.20g035900-AcetylGluT
8581267 Glyma.20g046000-P450
10672932 Glyma.20g050500-Glu6Pt
11802411 Glyma.20g052000-UDPGlyT
24195404 Glyma.20g067800-XET8
25093892 Glyma.20g070500-S6PPH
27993600 Glyma.20g077300-PL
30973414 Glyma.20g082300-Myb70
32388404 Glyma.20g086900-ALDH.12A1
33087828 Glyma.20g089100-EXP.A17
33253476 Glyma.20g090200-GLP10
33289800 Glyma.20g090500-PAE
33954992 Glyma.20g096700-GalO
34439996 Glyma.20g101100-GST
34568808 BARC-Satt354
34579856 Glyma.20g102900-MYO
35011920 Glyma.20g107500-bHLH
35177400 Glyma.20g109300-UDPGluT.72B2
35362576 Satt270
35468912 Glyma.20g112600-PL
35592128 Glyma.20g114200-C4H
35942036 Glyma.20g116900-MBOAT
36724560 Glyma.20g124900-AGP20
36842372 Satt049
36958692 Glyma.20g127700-GST
37015600 Glyma.20g128600-CAD(TC204220-2)
37106096 Glyma.20g130200-bHLH
37120056 Glyma.20g130400-FUC
37387104 Glyma.20g133900-GlyT
37412136 Glyma.20g134500-GlyH
37465148 Glyma.20g135200-PRP
38684624 Glyma.20g148400-Cupin
39053052 Glyma.20g151700-HRGP
39281184 Glyma.20g153900-CK.pfkB
39311328 Glyma.20g154200-MADS.AGL42
39365072 Glyma.20g154800-GMCO
39614892 Glyma.20g157100-SIS
39666784 Glyma.20g157900-Myb19
39742640 Glyma.20g159100-NAC.vr6
39814912 Glyma.20g159700-P450
39833260 Glyma.20g159900-PAE
40037988 Glyma.20g162800-HD
40103552 Glyma.20g163500-ABCtr
40155772 BARC-Satt330
40220888 Glyma.20G164700-ECT.GH18(AK244623a)
40232576 Glyma.20G164900-ECT.GH18(AK244623b)
40568844 Glyma.20g167900-UDPGlyT
40650320 Glyma.20g168700-INV.PMEI
40696468 Glyma.20g169200-Pox
40983076 Glyma.20g172100-NAM.NAC17
41167904 Glyma.20g174300-FT13
41337424 Glyma.20g176100-OMT
41415976 Satt162
41446960 Glyma.20g177200-INV(TC276114)
41891640 Glyma.20g180800-PAL2
42232856 Glyma.20g184200-Myb5
42834928 Glyma.20g189800-LAC
42864412 Glyma.20g189900-GlyT47
43061156 Glyma.20g191800-Pox
43138132 Glyma.20g192500-NAM.NAC50
43506736 Glyma.20g197600-GST
43537844 Glyma.20g198000-BGlu.GBA2
44526100 Glyma.20g209000-MT
44596844 Glyma.20g209800-ADF6
44794668 BARC-Satt102
46665752 Glyma.20g233900-BMann(TC213549)
47264268 Glyma.20g241600-CHI2(AY595415b)
47267104 Glyma.20g241700-CHI2(AY595415a)
47904180 Stop
Gm20(I)
Satt571 0.0
Satt419 4.8
Satt367 13.9
Satt354 31.6
Satt270 35.7
Satt049 45.2
CAD4440B 46.0
UBC570-400 57.5
Satt330 68.0
Satt162 77.4
Satt102 92.3
DM9R/3.83p
C8H/1.94
C9W/5.05p
L9R/3.61p
FS9H/3.65p
FPH8W/3.45p
TM9W/2.82p
IND8W/3.81p
LG9W/2.82
C8R/2.05
FM8W/2.68
UTS8W/2.35
TM8W/3.61p
IND9H/2.74
X
UBC300-800 0.0
UBC388-800 14.5
H/L9H/2.36nad
TS8W/2.41nad
IS9W/4.91p

Table A in S1 File. Fiber (cell wall-related) gene-based PCR primers.

Protein	Accession	Primer Sequence 5′ to 3′	Primer Position	PCR (bp)
**Lignin**				
Phenylalanine ammonia lyase	X52953 (PAL1)	AAAATGACTCATGAGCCTACCA<br>AGTTCCTTCTGCAGAGCACCAC	44-574	531
		CAAACAGGGTGGTGCTCTG<br>TTGTACCACGAAGGTCCAAAC	545-2296	1752
		CAACAATGGATTGCCTTCAA<br>CAGAGAGAAAGATGGAAGTTGGA	3003-4073	1071
	TC225162 (PAL2)	CATTTTTAAACCTAGCTCCATCTCC<br>CAATAGGAGTTCCTTGGAAGTTACC	30-1289	1260
	AW351172 (PAL3)	ATCGGCGTCACTGGAAATC<br>GCACGAGGTCCACAGATTG	2-1212	1211
Cinnamate 4-hydroxylase	X92437 (C4H)	AATGGATCTCCTCCTTCTGGA<br>TCTTCGTTGATCTCGCCTTT	50-934	885
		CCAAGAGCACCAACAACAAT<br>AAACCGTGCATCACTTCAA	850-1750	901
4-coumarate:CoA ligase	AF279267 (4CL1	GGCACCTTCTCCACAAGAAA<br>GCTTCTTGAAGTTCCCCTCCT	38-1000	963
		CGTTGGTTAAGAGCGGAGAG<br>GGAACAAAAACCACAACAGG	904-1755	852
	AF002259 (4CL2)	CCTCCACAAATCAATCTCCAG<br>CGGACTTCAAGACTCTGATGG	8-1000	993
		GTCACTATTGCCCCTGTCGT<br>CTAGCGGGATCAAGCAACAG	901-1809	909
	AF002258 (4CL3)	CTGCTTCTCTTGATGCCAAAG<br>CCGACAGCACTAACCGTATTG	14-1000	987
		GTTAGTGCTGTCGGGAGCTG<br>CACAATACAGGCATGGTGGT	987-1900	914
	X69955 (4CL4)	CACATTCAGACACCACCATGA<br>GTCAAAATCCGCCACCATC	17-981	965
		GGCAAAGAATCCGATGGTG<br>CAGACATGGTGGTGGACAAT	951-1903	953
p-Coumarate 3-hydroxylas	TC205337 (C3H)	CACAGGCTCCACAAAACATC<br>ATGGCCCACTCAACTGAAAT	6-982	977
		CCATCATTGGTCTCCTTTGG<br>GTCCAATGCCATTCCGTTAC	910-1729	820
Cinnamyl alcohol dehydrogenase	TC204440 (CAD)	TTCTGTTCATCCTTTGCTTCC<br>CAGAAGAGCTTATCACAGTCACA	15-699	685
		GTCATCATGTGACTGTGATAAGCTC<br>TCTTTCAATGGCGGAACG	670-1414	745
	TC225589	GCTGCCGGTTATATTGCTTC<br>AACGAAACACCGTTGAGATTC	57-1055	999
	TC229375	AAGGAGGAAATGGAGGAAGC<br>CGCTAATTTCTCAAACGTTGC	47-1000	954
Cinnamoyl CoA reductase	TC226045 (CCR1)	TGTTCCGTTCTACTTTCCCTTC<br>ATTCCAAATCACTCCAACAGG	11-500	490
		CCACCGTGGAGAATTAGTTGA<br>TTCAACTCAACGAGGGGAAT	808-1252	445
	TC219427 (CCR2)	GAGGATTCGTAGCGTCTTGG<br>GAGAGCCAGGCTACTTGCAT	5-671	667
Sinapyl alcohol dehydrogenase	TC227395 (SAD)	GCAAATGAACTACCCGTCAAAG<br>TTCCCGGCCCTGCAATTA	49-958	910
Ferulate 5-hydroxylase	TC217041 (F5H)	GAGGGCGAAATCATCCAAAC<br>GACCCGCTTTTGATCTTCTG	13-1097	1085

		ACGGAAACGGTAGCGTCA<br>TGTTTCTGCACAAAAGCTCA	1023-1822	800
Caffeoyl-CoA 3-O-methyltransferase	TC228561 (CCoAMT)	GCAGGAGAGGCACTGTAAGTC<br>CGACACTCCCAGTTTCACATTAG	40-1002	963
	TC228993	ATACGCTGCAGTTTGATTGC<br>AACCCTCACTAAAGGGAATAAGC	14-983	970
	TC225342	CAGAGAATTCCAAAGACAGAGATG<br>AATCCTTCCAAGCAGACACG	19-903	885
	TC204613	GGGAAGCTCCAATCAACTCTT<br>CCACCCTCCTGATAAAGCAC	4-1094	1091
	TC225339	GATTCAAGACCGGCATGG<br>TCGGATACAATTCGGTGTCA	29-1136	1108
Caffeic acid O-methyltransferase	TC226262 (COMT)	CATGGGTTCAACAGGTGAGA<br>CTTTCGGAACACTCGCAAAC	25-800	776
		GGAATGACAGCCTTTGAGTACC<br>TTGGCAGTCTACATTGAACTTG	500-1268	769
	TC226264	GGACTTAAATCCCTGGTTGATG<br>AAGGCGGTATACATTGAACTTG	57-710	654
Aldehyde dehydrogenase	TC215334 (NAD/REF1)	ACACCTATTATGGAAGGTGTGC<br>AATAGCAGCACCGGCAGTT	46-1096	1051
		CACCATTGTTCTCAAAACAGC<br>CACAGTTCAATCATAACACTGC	961-2218	1258
	AY250704 (ADH)	GACTATACCGGCACCGAAGA<br>CCACCAATTTCAGCTCCATT	259-1431	1173
	BG405171	CGGCACGAGGGAGAGAACTA<br>GGAAGATGGGAACTCGAGAA	7-327	321
	CA799072	GGCCATATCATGTGCAAACA<br>GCATGTCATCCTTACCATTTGA	33-770	738
Pinoresinol-lariciresinol reductase	TC206041 (PLR)	GCACGAGTGAAGAACCAACA<br>CCGTGTTCCTGTTTGAATCAC	2-1035	1034
	AK244769	CAACTGAAGAACCAACAAGAGG<br>TCAACGTCTTCTTGGATGAGG	6-1225	1220
Secoisolariciresinol dehydrogenase	TC227077	GCTTAGCAGCGAGCTTATCC<br>TGGTCATTATTTGGCTGATG	93-1006	914
	V274513	TGTTCCTTCTATCCCTTTCTTTTG<br>ATCCTCAGCTTTAAGAGTGACACC	10-1479	1470
Polyphenol oxidase	CA784777 (PO)	GCAGCAATGGGTAATCCTTC<br>GGAATTTGCATCCCTTCTACAG	22-749	728
	TA63189	GAACGATCATGGCTTATATCTCC<br>TCGCACCAGAGACAACACTC	4-963	960
Sinapyl alcohol dehydrogenase	TC227395 (SAD)	GCAAATGAACTACCCGTCAAAG<br>TTCCCGGCCCTGCAATTA	49-958	910
Alcohol dehydrogenase	AF079058 (ADH1)	CCAGACCATCAAGTGCAAAG<br>TCCAAGTCCAAATATGGCAAC	8-1877	1870
		TGCTGCTCCACTTGACAAAG<br>GGGCTGTTCCTATCATCTTATC	1652-3166	1515
Hydroxycinnamoyl-CoA hydroxycinnamoyl transferase	TC215709 (HCT)	TTCAAATGTGGAGGGGTGTC<br>TGCAGCATACCATGTTGGTT	26-592	567
		GCAACACCATGTAGCAGACG<br>GAAGACGCTGAAGAGTATCTGGA	11-1243	1233
	AK286472	TGGTAACCATTGTGGGTTCTTAC<br>TGACTTTAACATGCCCAGAAAC	20-1486	1467
Peroxidase	L81148 (Pox)	CGTACGTGATATTATCTAGTGTCTCTC<br>GTTCAGATCACTTGATTTGTCTCC	21-1010	990
		TGGTCATACGTTTGGAAGAGC<br>GCAAAATCGAATTGACATGC	2694-3296	603
	AF019116	TTCTCTTGGCAATTAAGGTCTC<br>GGCAAATTCCGCAAAGAAC	37-988	1348

	AF145349	GGTGTATTTCATATACCGTTGC ATTTGGCCCATTTTCACAAC	16-968	953
Dirigent protein	TC231423 (DIRP)	GCACGAGGCAATTCTCACTT CCGTTCTCAATAATGGAACACA	8-632	625
Chalcone isomerase	AY595415 (Chi2)	ATGGCATTTCCGTCCGTAAC AGTTGCTTCAATTCCCAACG	4-885	880
Chalcone synthase	X54644 (Chs1)	GCATGCATAGCATCTACGTACC TGTCCAACCAAACAACTTGG	1-2026	2026
	X52097 (Chs4)	GCTTCATCACCCACTTATTCC CCGAAACCAAATAGCACACC	3-2108	2106
Dihydroflavonol reductase	TA56057 (Dfr1)	AACACTGGCGGAGAAAGAAG TTGAAGATAACCTGGCCTTAC	2536-4165	1630
	EF187612 (Dfr2)	GCTTGGCCCGTTCTTCTATC ATGGCTTCATCAAAGCTTCC	144-1850	1707
**Cellulose**				
Cellulose synthase	TC215803 (CesA1)	CCCTTGTAGCCAAAGCTCAG GATGGAAATCCAACCACGAG	13-995	983
	TC215151 (CesA3)	AACAGTAAAGCCTTGAGGGAAG GTCAGGAGACAGACAGCAGGT	59-999	941
	TC230955 (CesA)	GCATGGAAAGAAAGAGTTGATG CTGTGCCTTTGAAACAAGTCC	48-862	807
	TC206323 (CesA)	GCACGAGGTTATCTCTAACTTTC CAAACACCACCCACAAATAAC	1-895	895
	TC219882 (CesA)	CTCTTGATTCCTCCAACCAC GGCAGTTCCAACCAAAACAG	22-525	504
	TC216172 (CesA)	GAAGGGCTAGAAGGGTATGAGG CTCAAGAATCAAGTTCCAGTAGATG	18-1442	1425
Secondary xylem cellulose synthase	BE806202 (CesA)	GGTTTCTGATGCACTCAATGG CAGGAAATTTCATCCAATGC	1-423	423
COBRA	TC216062 (COB)	CTATAGAAGCCTATGATCCACTTGA CAGTTGGTTTCACAACTTTGG	32-532	501
	TC239239 (Cob2)	CAAACACGCCACTAGTCCAG GAAGCAGCAGAAGTGCTACC	124-998	875
COBRA-like 4	TC210804 (COBL4)	GTTGCTAACACTTGGCACCAC CTAGCCAGAAATTGTGAGTATGTG	31-731	701
Chitinase-like 1	TC216133 (CTL1)	CTTAGCCTTCCAGGCTGCA GCAATCCTTAATGATCAAATACTCAATTG	88-709	621
Endochitinase	CA799153	AAGATGAGTCCAACATGGGTTT CAGCCTGGAAGGCTAGAGTG	2-635	634
	AB000097 (acidic, class III)	TTCCAATACACATGGCTTCC CAACATGCTTCGATTATATTGGTTAC	29-1231	1203
	TA54696	GAAATTCTTATGCGTTGTTGTAGC CGTTTAAAACTCCTCCTAAAAGTG	14-1045	1032
	AK244623	AATCATTCCAATACACATGGCTTC AGGGAGTATTTTATTCTCAACATGC	26-1250	1225
Sucrose synthase	AF030231 (SS)	GTTCACAGTCTCCGTGAGAGG ATCAGATCTGGCTTGCCTTG	96-1297	1202
		CACGAGCTTGCCAAAGAGTT GGCACAGACTCAGCCAATTT	1257-2473	1217
Sugar transport protein	CA785365 (STP)	GGTCATGAACATGCAACAGAG TCCATCGATAAGCTCCTTGG	18-737	720
Sucrose-phosphate synthase	TC286435 (SPS)	CAGCTTTCATTGAGCCATTTG TCCGTAATAACTTGCGAAGC	1130-2490	1361
Sucrase-like	BE806787	CGTCAACGGCTTCAATCG TCGAAGCTCAATTTCCTCATTC	11-485	475

Pectinesterase	BE805968	AGTGTGCAAGCAGAAGATGC<br>CCATAATAAGAGGCCACAGGA	12-536	525
Pectinacetylesterase	CA784831	GGCTCTTCTTTCTGGATGCT<br>ATTGCCTTGTTCCCAATGAC	7-720	714
Pectin methyl esterase	BE806415	CTCAATCCACCACCACCAG<br>CCTGTGACAACACATCTGAGG	3-407	405
Pectate lyase	CA785370	GGGAACTCTCCGTTATGCAG<br>CCTTCTGACCTCCAATTCCA	20-745	726
	BF008833	GCACACAACACAAACACCAA<br>ATGGACCACCCGCAATGT	1-514	514
Homogalacturonan (pectin)	TC217754	AATTACGCAGCGATGAGGAT<br>AAGCCCTACTCAGATGGAATTG	126-1031	906
	AK244415	AGAACGGGCACACAAGAAAG<br>AACTTGAATGGTTGCCTTGC	11-1465	1455
Beta-1, 3-glucanase	CA784107	CCACTGCCACCTTTAACTCTTC<br>CAATTTGCTCCTCCTTGTCC	1-658	658
Alpha-l-arabinofura nosidase	CA800570	AGGAGAGACCTGGGCACTTT<br>TCCACAATCTTCATTCCCAAC	8-312	305
	BE806620	GCTGAGCTTGTGAGCAACAG<br>GCTGTCAGCATTTCACAAGG	26-576	551
Alpha-glucan phosphorylase	BE806173	GAGATGAGCCCTGAAGAACG<br>AGGTAATCACCCGACCCATA	26-576	551
Arabinose kinase	BE806770	CCGAAGCGTACTTACGGAGT<br>GGGTAGCTCGACGACGTATT	42-492	451
Ascorbate oxidase	BE806303	CTCTGCTTGATAGCGGTAGGA<br>CGTAATGGTAGGAGCCTTGC	2-493	492
Beta-D-glucan exohydrolase	BG507311	GCCGATAATTTGGGTTACCA<br>GATCTACAGATTTGAACCATGTCC	34-508	475
Beta-glucosidase	CA802003	CCGGTACAAGAGCGACATAAA<br>TTGAGCTTTGGTGAATGTGG	49-751	703
Beta-galactosidase (Hemicellulose)	CA785185	CTTGTCCCGGCAGAAATG<br>CCACACGTATTTGGTAAATTGC	13-614	602
	TC322276	CCCAACAAGAACACCAAACC<br>TTGCGTGTCTACAATTCTTTCC	8-1916	1909
	TC330147	TTCCTGACTGCAAGAACACTG<br>AATGGCTGCCTTTGAGAAAC	6-1639	1634
		AAACATGGAATGCTGGTGTTC<br>GGGTCTCCACCTAGCTCTTC	1377-2600	1224
Alpha-l-arabinofuranosidase (Hemicellulose)	TC307152	GTTCATATTGGTTTGCCTTGTC<br>GCACCCAATAGCTAGGAGTTCAG	147-1726	1581
	TC322471	GTGCTCTATGCCTTTGCGTTC<br>GTTTGCAGCATCACTTTTCACAGC	73-1425	1352
Xyloglucan endo transglycosylase	XE/TA43776	TGGAACAGTCACTGCCTATTATG<br>GATCACAAACAATGGAGTAGTGC	6-1673	1668
	XE/TA45693	GCCACAACGAGTTTGATTTTG<br>CAAGCAGAAACATGGAGGAC	1107-2516	1410
Cellulase	CA801638	TGCCAAGACCCAGATTGATT<br>TTAAAGAGACACACTCTCAAGTACCAC	5-656	652
Fructokinase	CA784817	CCCATGGCGTTGAACAAT<br>TCCAAGAGTGACAAGGAGCA	4-726	723
Fructose-1,6-bisph. aldolase	AJ005041	TGCCAATGCTGCCTACATC<br>AATGCCAAACCTACCCTCTTT	78-1160	1083
Glucan endo-1, 3-beta glucosidase	M37753	CTTCTTTCCTCAACCTTCTTTCTTC<br>TTCAAACTCTGGCTGCTTCT	1-1019	1019
Glucose-6-phosphate isomererase	BG507569	TCATCAGATTGCACAACTTGG<br>ACTGAATAATAACAGGCTGTCC	32-500	469

Invertase (beta-fructofuranosidase)	AJ717412 (INV-alkaline)	TTGAGAGGCAGAGGTCGTTT ATCCATTCGGGCAGGACT	258-1297	1040
	TC276114	TCCCTGATTTGGATGATTCC AATGTCATTTAGTTGTGGAAGTCG	30-980	951
Malate dehydrogenase	AY496910	TTGGGTATGCTCTTGTCCCTA TCTTCTCCAGTGATAGTTCCTTTG	102-1172	1071
Xylulose kinase	CA799181	CTTTGGCAACGGTGAAATCT TTCCATGCAAGAACTCAACATC	1-652	652
	TC296666	GAAATTCAGCACTGCACTGAG CAGCAAAGGGTTGAGGAGAG	1-1579	1579
Nucleotide sugar epimerase	CA784906	GCGACGTTCCGTTTACTCAC ATCAACGAAGGAACAACAGC	14-444	431
DTDP-glucose 4-6-dehydratase-like	BI945853	GATTCATTGGGTCTCACTTAGTTG TAGGGCCATCAAACAGTTCAG	1-641	641
UDP-glycosyl transferase	TC216848F	TTATCGCCTCAAGGATCTGC CAAAGAAGACACAAACTAGCACCT	27-1034	1008
Glycosyl transferase	TC203711	GCCTCCACTCTCGCTTTACT TACGACACTCGGCTTACCG	132-1302	1171
	TC322929	TGAACGGACACGTCAAAGTC CTCCTTCGGGTGAGTTAAGC	54-1955	1902
		TCAATCGCTTTGAAAGGAAG GGAGCAAGACTTGGAACATTG	1881-4096	2216
Glucosyl transferase	TC283117	TCTTTCTACTTGCTCCCACCTC AATGTCATGTATGCCAAGTGAC	13-1762	1750
	GT/TA59613	CGTGAACCAGAACCAGAATG AAGGGACATGTTTCAAAGGTTC	10-1656	1647
Galactosyl transferase	BM519727	TTTGCTGCTTCCTTACCTACATC CTGGACCGTTAGCATAGATGG	6-1824	1809
	TA70286	GTGCCGTATTTGGATAACTATG ACAGAACTTGCATCTGCATC	30-1460	1431
	TC217730	GGATTGCGGTGAATACTGG GCCCAAATACAAAGGAAGC	79-894	816
Endo-1,3;1,4-beta-D-glucanase	TC229099	TTCCGTGTTACTCAAGGCAAC TTGCTTCAACCTTTCTGGTG	34-670	637
Endopoly galacturonase	TC212447	CACATTGTCATCACTGGGTTG CTCCAGATTTCAGATTTCAGAGC	2-759	758
Polygalacturonase	F128266	TTCTGTGTGGTCTTCAAGCTG GTATGCCTGAAACTGCCACA	129-1150	1022
UDP-glucose pyrophosphorylase	BF009075	TAAATGGAGGCTTGGGAACA GCAATTTCCAGGAGCTGAAC	12-576	565
Xyloglucan endotransglycosylase	CA799236	TTCTTTCTCTCTCCTCTTTGTGC AGTCCTTGTAGGTGGCAACG	11-693	683
Glycogenin glucosyltransferase	BG507518	CACTGTGAGTACGTGGCAAT GCGGAACAAATTAGCAGGAC	42-497	456
Endo-xyloglucan transferase	D16455	TTTCTGTGTGGACGGTGTGT TTTGCACTCAGGAGGAGAGA	13-872	860
Endo-1,4-beta-glucanase	DQ340400	TCTCAAAGCCACTGCACATC GGCTCCGATTGCTCATAATC	143-1177	1035
GDP-D-Mannose 4,6-dehydratase	CA784580	AGCGCATCGACCACATATAC AAGCACCTTCTTGGCCTTG	17-668	652
Mannosyl mannosidase	TC278832	CAGCAGGCTACTCTCCCATC GCAGGAAGTATCTTCTATTCAATGC	1266-2423	1158
Phosphoglucomutase	TC322502	GTGGACCTGCACCAGAGG TTTCAAATTCAGATGTTTGGCTAC	2621-4422	1802
Prephenate dehydratase with ACT	EH262010	TGGGTGTGAGAGTGAGAGAGC TGAAGCTTTGCCTGAGAATG	11-1898	1888

Alpha-glucan phosphorylase	AGP-TC261561	CAACAAAGGTTGCTGTACAGTTG GCAAATTTCATGTTGCTTGTG	6-1077	1072
ABC transporter	BF009025	CCACCTGTTGAAGGAACGAC TTGCTGATACACACGCATGA	26-397	372
Glucose 6 phosphate/phosphate translocator	AY942816	TTCGCCTACCCTTTACAATGA TGAACTGGTGTGTGGAAGAT	2-1133	1132
Nonspecific lipid transfer protein	CA784306 (NLTP)	TGAGTTATGGCAAGCTTAATCG ACATGAGTGGAACCCGAGTC	22-497	476
**Cell wall proteins**				
Cell wall protein	CA801101	AGGGATTGGACCTGTTGGAT TGTGCACATGGAGCATACTT	58-408	351
Membrane associated protein	TA41557	TTCAAACAACTGCACCCAAG ATCCATCTACAAATTATAAGAGCTG	655-2054	1400
Membrane-related protein	BF009085	TTCTCCACCACCAGTTCCTC CAAATTCGCCTCCGTATGA	2-373	372
Integral membrane protein	TA44750	TCAGCAATTATCTGGTATCAATGG CATAATAGACTACCTCTGTGCTCTCC	1356-3206	1851
Late-embryogenesis abundant protein	TC303667	GAAGAACAAAGATGCAATCCTC TGGACCGTCAATGATGATG	45-1186	1142
Proline-rich APG-like protein	TA69674	GAGTTGGGAGCTAGAAGGGTTC AGCTAGTAGTGATGTTGCACTTG	11-1160	1150
Hydroxyproline-rich glycoprotein	L22030	TCACCGCCGTCTCCATATTA TGCAAACACAACAACAGGGTA	187-917	731
Proline-rich protein	J02746 (PRP)	CGAAGAGGTACGTGCCAAATTACATC CACTCTTGTCGTGGCCATAG	1-2087	2087
		ACCCATCATTGGGTGTGTGT CGATCTATTAACAGCAGCATGTAG	1085-2835	1751
Proline-rich protein 2	J05208 (PRP2)	GTATACAGTAAATCGCTACTTGAG GCAACCCCTTGCAATAATTTG	1-2051	2051
		CACTTGTGTGGACTTGTCAATG CCTTGTCATATGCAAAGAGATTG	1031-2763	1733
Repetitive proline-rich protein	X16574 (RPRP)	GATCGATCCTCTAGAGTCGAC ATTTCGTACAATTCGTGTGG	22-1661	1640
		ACGTGAGAAACATGGCTTCC CAACTTCGTAATATTGAACAAATTCACG	661-2811	2151
Glycine rich protein	AF169205 (GRP)	TGAGTACCGTTGCTTTGTCG GTTTCTCCAGCCTCCATCAC	18-480	463
Glycyne rich protein 10	TC208462 (GRP10)	CTGTGAAATCGCAAAGAAACAG ACCTTTGTAGGCATCGGAAA	62-793	732
Arabinogalactan protein	CA801105	TCAGTTGTTCACACCTCACTCAC TGAAAGGGACAGCCAAATTC	2-735	734
Germin-like protein	EU916269 (GLP)	CCCATCACTTCCTTCCAACTC GCAAGGAAGCAAGCATCTAATTTC	14-997	984
	TA51907	CCTTTTCAGTTTCCACTCATTG GCAAAATGCAACACGGTAAG	30-1274	1245
	CA800012	CATGTCACCAACAAACAACACT GAGCAAGCTTGGCCTTGAT	59-711	653
14-3-3-like protein	CA784856 (LA)	GGTGGAGTTCATGGAGAAGG CTGTTTCGGTGCTGCTTCTT	4-691	688
	CA785232 (LB)	CTCGCAAGCAAATCAGAACA CGCCCGTCATCTTCAAGTAG	1-433	433
	CA784740 (LC)	TGGAATTGACGGTAGAGGAG CCCAAATACCATCAGCTAAA	1-704	704

	BG405219 (LD)	CACGAGGCAGAGTGAGAAAG<br>TGATCAGCAGCTTCCTTCTTC	9-520	512
Stem-specific protein TSJT1	BF008688 (TSJT1)	TCTGCTGTTTACAACCTTCCA<br>TGATTAGCAACTTGGTCAGGAG	2-252	251
Phloem-specific lectin	BE806245	GGTGAGAATGGAGTTTCAAGG<br>GGCCACCAAATACATTGACC	1-560	560
Expansin	CA785153	CCCTCAACTCCATAAGCCACT<br>CTTTGGCCATTCAGGTAGGA	19-697	679
Extensin	CA784792	CCTCCTCCACCTAAGCCATAC<br>CAAGCGCAGCAGGTTGAA	30-454	425
**Other proteins**				
Actin	U60503	CCTACTGTCATACTCTTGTTGGTTT<br>TAGGGACAGTGTGGCTGACA	44-897	854
Actin depolymerizing factor-like protein	TA42046	TCAGAACCCTCGATCACTCC<br>TGAACAAGATGCCTTTGTATGC	37-1998	1962
Annexin	TC281997	TGTGTTGGTTTGGACATTGG<br>AAGAGGTGAAGCGCTGTGTC	5-1854	1850
Argonaute	Argon-TA68302	AGTGAATCACAATTCGAACAAG<br>AGCCCATGCGACTCAATTAC	1466-2819	1354
Calreticulin-1	AB196794 (Crt1)	CTCACGTTCATTTCTACACATCC<br>CGGAATATAACTTCCGCTCAC	6-1361	1356
Cucumisin	TC293154	TGCAGCAGCTGTTTACATCAAATC<br>AAAGCACTTGCCATCAATTCTG	1-780	780
Syntaxin	AF532627	CCTTCTTCGTCCCAGATCC<br>TTAATTAAAGAAACATGGAGAGAAC	1-1171	1771
Pattern formation protein EMB30	TC304111	GGCACATCTGAGGATGAGG<br>ACAAAGACCAATCCGGTAGC	12-1377	1366
Signal peptidase	TC280896	CGAAGAAGAGTGTGGACAGTAGC<br>TGAATTCCAACTATCATAGAGTGTCAG	1-780	780
Elongation factor	X56856 (EF)	GTGTGCTAAACATGCCCTTG<br>TCCAATCTTGTACACATCCTGA	177-1300	1124
**Regulatory proteins**				
LIM1	TC204443	TCTTGGTTGCACCTTGGTAAG<br>AAGCAGGAGATATAGCACAAACAC	119-833	715
	TC204441	TGTCCTGTTCTTTCCCTTGG<br>ACAGATGATGAAGATCCACACAG	140-990	951
Myb4	TC232662	TCTGAGACCCGATATAAAGAGAGG<br>TTCATCTGGCTTCTCCTCAAC	9-642	634
	NM_120023	GTCACCGTGCTGTGAGAAAG<br>TCCATTGCTCATGTCACTCC	324-1092	769
MADS box protein	MADS-TC298698	CTTCTCAAAGCGCAGAAATG<br>AATCAAAGCTACATCAATGGAC	2-782	781
KNAT7-1	TC274106-1F	GCCTTTGAGGAAAAGAAAGGA<br>GGCGAACTCTTTCCATCAAT	2-567	566
IFL1	TC271859	TTTCTTTCTTTCTCTCTCTCTCC<br>GTCACGGAACCAAGATGGAC	1-976	976
ATHB8	NM_119441	CGCAAATCTTGACTCTTCACG<br>GCAACCAACAAGGCTTATCC	46-1077	1032
ANACO12	NM_103011	ATGGCTGATAATAAGGTCAATCTTTC<br>CAGATAAATGAAGAAGTGGGTCTAAAG	1-1603	1603
NST2	At3g61910	CGTTCTATTTTCTTCTCCTTTTTC<br>ATTATCACAATCTGACCATTTATACCG	1-1380	1380
NST1	At2g4677-1	GTTACAATTAAATAAACCTCACATGC<br>TCATGAACGGTGACATCCTC	27-878	852
Myb85	At4g22680	GACATAACTTGCAAGCTGTTGAG<br>AAGGATACAAAGATATGCAGGAGAG	1-1848	1842

Myb46	At5g128706	AAATATGAGGAAGCCAGAGG<br>CTAATCATTACATGGCACCTCAAG	50-2219	2170
Myb63	At1g79180	GGAAGAGCACCTTGTTGTGAC<br>CTCTACTATCTCTCTACCTTTCCATC	10-1148	1139
Myb26	At3g13890	ATGGGTCATCACTCATGCTG<br>AAGAGATTGGCGACGATGAG	1-1261	1261
SND3	At1g28470	GGTAGGTCACTTGTATAGAGAGATCAG<br>TCAGTCATGTCTCGAGGTTAAAG	39-1889	1851
Myb103	At1g63910	ATGGGTCATCACTCATGCTG<br>GTGAAAATCCCGTTACAAACTC	1-1754	1754
SND2	At4g28500	ACTTGGTGCAATGACCGTAG<br>TATCCCGGTCTGATTGAACG	4-1211	1208
Myb20	At1g66230	GGTGGTTGCAAGGCTTACC<br>ACTAGAGTAACGAAGGGTATTTAGG	25-1186	1162
VND7	At1g71930	GTTTATAGGATCATCGTGGATGG<br>GCTTCGACCTCATTATAGCTTTG	47-1650	1604
VND6	At5g62380	ACATTCCTCCCGGTTATCG<br>AGCATTCATCGAAACCATTG	17-1118	1102

Table B in S1 File. Analysis of variance (Fisher test values) for agronomic, fiber compositional and composite mechanical traits.

Trait	Effect						
	Genotype (G)	Year (Y)	Location (L)	G x Y	G x L	Y x L	G x Y x L
Days to maturity	17.48**	160.98**	419.71**	1.40*	2.91**	832.02**	1.60*
Plant height	9.23**	140.73**	0.10	1.31	1.35	107.27**	0.90
Lodging	12.20**	5.16*	0.29	1.30	1.38*	42.32**	1.21
Height/Lodging	8.46**	23.28**	0.10	1.18	0.98	11.55**	1.07
Cellulose	1.91**	0.52	29.17*	0.99	1.33*	9.79**	1.14
Hemicellulose	1.14	20.55**	2.61	0.88	0.82	4.99*	0.96
Lignin	2.20**	50.24	5.57	1.59**	1.04	19.91**	1.29
Free phenolics	4.27**	125.21**	9.10	2.06**	3.99**	5.70**	3.27**
Flexural strength	5.67***	11.83**	24.45***	7.00***	7.88***	21.79**	6.55***
Flexural modulus	3.11***	0.13	13.50***	4.59***	3.81***	16.39***	3.19***
Ultimate tensile strength	7.62***	129.04***	1.80	7.21***	6.42***	0.23	5.87***
Tensile strength	6.45***	24.83***	5.25	5.33***	4.41***	0.07	5.08***
Tensile modulus	3.08***	9047**	0.43	4.76***	2.86***	3.96*	2.72***
Impact strength	2.24***	0.31	0.60	2.33***	1.74**	5.17*	1.72**

*Significant at P = 0.05
**Significant at P= 0.01
***Significant at P = 0.001

Table C in S1 File. Comparison of mechanical performance of stem fibers in soybean/polypropylene (SS/PP) composites with pure polypropylene (PP) and wheat straw/polypropylene (WS/PP) composites.

Trait	Year	Location	SS/PP						PP	WS/PP
			RILs[a]	Best performing lines[b]						
				RO7	RO58	RO139	RO147	RO167		
Flexural strength (MPa)	2008	Harrow	49.3**	51.6**	48.8**¶¶	50.7**	50.2**	51.5**	41.8	52.8
		Woodstock	49.2**	48.6**¶¶	49.0**¶¶	48.9**¶¶	49.9**	48.1**¶¶		
	2009	Harrow	49.3**	51.7**	45.8**¶¶	51.5**	50.9**	50.6**		
		Woodstock	48.6**	48.7**¶¶	49.9**	48.6**¶¶	48.7**¶¶	50.9**		
Flexural modulus (MPa)	2008	Harrow	1347**	1466**	1383**¶¶	1368**¶¶	1358**¶¶	1436**	1010	1565
		Woodstock	1349**	1327**¶¶	1430**	1363**¶	1431**	1305**¶		
	2009	Harrow	1361**	1474**	1236*¶¶	1416**	1443**	1388**		
		Woodstock	1327**	1289**¶	1394**	1330**¶	1397**	1426**		
Ultimate tensile strength (MPa)	2008	Harrow	35.7*	38.4¶¶	31.7**	36.7	35.5	36.0	36.8	34.2
		Woodstock	35.5	36.5	31.1**	35.1	36.6	35.7		
	2009	Harrow	34.7**	37.1	34.3	34.1	36.4	34.7		
		Woodstock	34.4**	34.3	37.2	35.5	34.1	35.5		
Tensile strength (MPa)	2008	Harrow	32.8	35.9	30.3*	32.6	32.8	33.8	33.9	32.9
		Woodstock	32.5	33.8	30.1	30.7	33.4	33.3		
	2009	Harrow	32.3*	35.8	32.4	32.6	33.3	31.8		
		Woodstock	31.2**	32.5	34.4	33.9	32.5	33.0		
Tensile modulus (MPa)	2008	Harrow	259*	277	177¶	260	247	285	222	276
		Woodstock	261*	272	173¶	288	262	233		
	2009	Harrow	256*	230	254	248	259	277		
		Woodstock	250*	216	318**	250	284	272		
Impact strength (J m^{-1})	2008	Harrow	26.5*	26.8	30.8	27.0	31.6	24.4	30.9	23.1
		Woodstock	26.8*	28.3	32.4	32.5	25.8	28.0		
	2009	Harrow	26.8*	24.7	27.0	29.5	24.8	33.2¶		
		Woodstock	26.0**	25.2	28.4	31.2	28.1	26.4		

[a]RILs population mean was compared with pure PP and WS/PP composites using mixed model procedure (contrast analysis) in SAS

[b]Means of five best performing RILs were compared with pure PP and WS/PP composites using mixed model procedure (adjust= Tukey) in SAS

*Significantly different from pure PP at P= 0.05

¶Significantly different from WS/PP at P= 0.05

Table D in S1 File. Soybean (*Glycine max* Wm82.a2.v1) sequence map (partial, Phytozome v9.1).

Chromosome Gm01 (1-56831624)[a]

Feature	Position	Annotation
Glyma.01g014000-Csl	1227738	Cellulose synthase-like
Glyma.01g020900-CAD	2104937	Cinnamyl alcohol dehydrogenase
Glyma.01g021000-CAD	2122589	Cinnamyl alcohol dehydrogenase
Glyma.01g031500-ALDH(CA799072)[b]	3278502	Aldehyde dehydrogenase
Glyma.01g039800-GalT(BM19727)	4321369	Galacturonosyltransferase
Glyma.01g091400-CHS4(X52097)	27621455	Chalcone synthase 4
Glyma.01g167500-MannD(CA784580)	50521867	GDP-mannose 4, 6 dehydratase
Glyma.01g189200-GlyT43(AI460843)	52433139	Glycosyl transferase family 43
BARC-Satt071	***53600489***	***SSR***[c]
Glyma.01g203400-GlyH14	53662610	Glycosyl hydrolase family 14
Glyma.01g208700-OFT	54037651	O-fucosyl transferase
Glyma.01g209500-PE	54103335	Pectin esterase
Glyma.01g211000-BFF.INV	54261857	Invertase/Glycosyl hydrolase family 32
Glyma.01g211500-Myb20(At1g66230)	54292771	Transcription factor AtMyb20
Glyma.01g213000-MatE	54426718	MATE efflux family protein
Glyma.01g216900-P450	54720766	Cytochrome P450
Glyma.01g217000-P450	54731408	Cytochrome P450
Satt147	***54828484***	***SSR***
Glyma.01g232400-4CL4(X69955)	55890929	4-coumarate:CoA ligase 4
Glyma.01g232500-Csl	55898581	Cellulose synthase-like

Chromosome Gm02 (1- 48577505)

Feature	Position	Annotation
Glyma.02g005500-MalD(AY496910)	597924	Malate dehydrogenase
Glyma.02g016400-E14BG.GlyH9(DQ340400)	1466563	Endo-1,4-β-gucanase/Glycosyl hydrolase family 9
Glyma.02g025000-GalT(BM519727)	2208388	Galacturonosyltransferase
Glyma.02g034000-ALDH(TC215334)	3157712	Aldehyde dehydrogenase
Glyma.02g080900-CesA(TC206323)	6970629	Cellulose synthase
Satt157	***9327815***	***SSR***
Glyma.02g103500-CCoAOMT	9836681	Caffeoyl-CoA O-methyltransferase
Glyma.02g108100-B13GalT	10325912	β-1,3-galacturonosyltransferase
Glyma.02g120300-FK.l2	11862175	Fructokinase- like 2
Glyma.02g123200-OFT	12289339	O-fucosyltransferase
Glyma.02g128200-MT	13093448	Methyltransferase
Glyma.02g133500-ADH.REF	13806600	Alcohol dehydrogenase
Glyma.02g143700-MT	14826269	Methyltransferase
Glyma.02g147800-HLH.TT8	15251037	Basic helix-loop-helix transcription factor AtTT8
Glyma.02g154400-GST	15832224	Glutathione S-transferase

Satt537	***15993654***	***SSR***
Glyma.02g156100-P450	16311871	Cytochrome P450
Glyma.02g158700-DFR.TT3	17453873	Dihydroflavonol 4-reductase AtTT3
Satt428	***17770710***	***SSR***
Glyma.02g162600-Myb	21485033	Myb-like transcription factor
Glyma.02g164500-MT	23438212	Methyltransferase
Glyma.02g169800-DIRP(TC231423)	26294277	Dirigent-like protein
Glyma.02g171600-Pox	26982395	Peroxidase
Glyma.02g172800-Act	27461138	Actin
Satt005	***27699285***	***SSR***
Glyma.02g176200-P450	28900535	Cytochrome P450
Satt600	***29355267***	***SSR***
Glyma.02g178100-Myb	29805316	Myb-like transcription factor
Satt604	***31234716***	***SSR***
Glyma.02g182500-AKR	31249609	Aldo/keto reductase
Satt141	***32004124***	***SSR***
Glyma.02g186800-GlyH3	34273084	Glycosyl hydrolase family 3
Satt189	***34993207***	***SSR***
Glyma.02g189600-MT	35576153	Methyltransferase
Satt350	***36833446***	***SSR***
Glyma.02g196100-COBL	37119590	COBRA-like extracellular glycosyl-phsphatidyl inositol-anchored protein
Sat_135	***37285448***	***SSR***
Sat_169	***37813855***	***SSR***
Satt644	***38221027***	***SSR***
Glyma.02g202500-ALDH	38754600	Aldehyde dehydrogenase
Glyma.02g205800-CesA	39076970	Cellulose synthase
Glyma.02g208000-UDPGlu4E(CA784906)	39276566	UDP-D-glucunorate 4-epimerase 1
Glyma.02g209000-Pox	39419801	Peroxidase
Glyma.02g218400-MatE	40642050	MATE efflux family protein
Glyma.02g222400-FBPA(AJ005041a)	40998557	Fructose-biphosphate aldolase
Glyma.02g224800-LAC	41222747	Laccase/Diphenol oxidase family protein
Sat_139	***41802760***	***SSR***
Glyma.02g231600-LAC	41882853	Laccase/Diphenol oxidase family protein
Glyma.02g236500-C4H(X92437)	42416141	Cinnamate 4-hydroxylase
Glyma.02g240400-SS.GlyT1	42892680	Sucrose synthase 6/Glycosyltransferase family 1
Satt172	***43443481***	***SSR***
Glyma.02g250000-EMB30(TC304111)	43747970	Sec7-domain-containing protein/Pattern formation protein
Glyma.02g250400-MT	43786923	Methyltransferase
Glyma.02g259300-Pox	44632522	Peroxidase
Glyma.02g261600-LAC	44758969	Laccase/Diphenol oxidase family protein
Glyma.02g265300-GRP	45028909	Glycine-rich protein family
Satt459	***45311085***	***SSR***
Glyma.02g286100-Csl	46696850	Cellulose synthase-like

Glyma.02g298300-ABCtr(BF009025c)	47557607	Multidrug resistance-associated protein 3/ABC transporter
Glyma.02g298400-ABCtr(BF009025a)	47564697	Multidrug resistance-associated protein 3/ABC transporter
Glyma.02g298500-ABCtr(BF009025b)	47580167	Multidrug resistance-associated protein 3/ABC transporter
Glyma.02g301200-Csl	47715921	Cellulose synthase-like
Glyma.02g303000-FBPA(AJ005041b)	47847723	Fructose-biphosphate aldolase
Glyma.02g309300-PAL3(AW351172)	48287101	Phenylalanine ammonia lyase 3

Chromosome Gm03 (1-45779781)

**Feature**	**Position**	**Annotation**
Glyma.03g014600-NADME	1465953	NAD-dependant malic enzyme
Glyma.03g016700-MT	1683395	Methyltransferase
Glyma.03g019300-MADS.AGL20	1952796	MADS box transcription factor/AGAMOUS-like
Glyma.03g021600-P450	2240686	Cytochrome P450
Glyma.03g024200-GlyH18	2607579	Glycosyl hydrolase family 18
Glyma.03g027700-MT	3035702	Methyltransferase
Glyma.03g028600-INV.PMEI	3119570	Invertase/Pectin methylesterase inhibitor superfamily protein
Satt159	***3197845***	***SSR***
Glyma.03g030300-P450	3356626	Cytochrome P450
Glyma.03g032500-UDPGlyT	3716149	UDP-Glycosyltransferase superfamily protein
Glyma.03g037900-Pox	4661075	Peroxidase
Glyma.03g045400-MT	5760642	Methyltransferase
Glyma.03g047600-Myb74	6047876	Myb transcription factor Myb74
Glyma.03g059700-ALDH.5F1	8688057	Aldehyde dehydrogenase 5F1
Glyma.03g061400-B13GluT	9286580	β-1, 3-glucosyltransferase
Glyma.03g654500-GlyH16	11450263	Glycosyl hydrolase family 16/Xyloglucan endotransglucosylase
Glyma.03g065900-HAD	12065850	Haloacid dehalogenase-like hydrolase (HAD) superfamily protein
Glyma.03g066600-AKR	13349361	Aldo/keto reductase
Glyma.03g072900-WD40	17628315	Transducin/WD40 repeat-like superfamily protein
Glyma.03g074400-Tub	18514546	Tubulin
Glyma.03g074900-OFT	18568298	O-fucosyltransferase family protein
Glyma.03g077800-P450	19287169	Cytochrome P450
Glyma.03g077900-LAC	19361933	Laccase/Diphenol oxidase family protein
Glyma.03g081900-Myb91	22433399	Myb transcription factor Myb91
Glyma.03g083000-GlyT14	23023402	Glycosyltransferase family 14/Core-2/I-branching β-1,6-N-acetylglucosaminyltrans.
Glyma.03g085000-P450	24502398	Cytochrome P450
Glyma.03g086600-Csl.C6	25553545	Cellulose synthase-like C6
Glyma.03g090800-Myb94	26974147	Myb transcription factor Myb94
Glyma.03g091300-ADH	27082164	Alcohol dehydroganease
Glyma.03g095400-MT	28125306	Methyltransferase
Glyma.03g095600-PLl.GlyH28	28259791	Pectin lyase-like superfamily protein/Glycosyl hydrolase family 28
Glyma.03g103400-INV.PMEI	29748622	Invertase/Pectin methylesterase inhibitor superfamily protein
Glyma.03g106600-P450	30584263	Cytochrome P450
Glyma.03g109100-WRKY	31002094	WRKY transciption factor

Glyma.03g111400-Csl	31631299	Cellulose synthase-like
Glyma.03g115300-P450	32313152	Cytochrome P450
Glyma.03g120000-ALAF(BE806620)	33134814	α-L-arabinofuranosidase 1
Glyma.03g122000-C3H(NM_180006)	33466244	*p*-coumarate 3-hydroxylase/Cytochrome P450 family 98
Glyma.03g132600-B13Glu.GlyH17(M37753c)	34749959	β-1,3-glucanase 1/Glycosyl hydrolase family 17
Glyma.03g132700-B13Glu.GlyH17(M37753a)	34758007	β-1,3-glucanase 1/Glycosyl hydrolase family 17
Glyma.03g132900-B13Glu.GlyH17(M37753b)	34783733	β-1,3-glucanase 1/Glycosyl hydrolase family 17
Glyma.03g181600-PAL1(X52953b)	39351927	Phenylalanine ammonia lyase 1-like
Glyma.03g181700-PAL1(X52953a)	39362677	Phenylalanine ammonia lyase 1
Glyma.03g217500-Csl	42137961	Cellulose synthase-like
Glyma.03g227400-GLP(EU916269)	42925859	Germin-like protein 5/Cupin
Glyma.03g227700-Myb26(TC276967)	42961283	Myb transcription factor Myb26

Chromosome Gm04 (1-52389146)

**Feature**	**Position**	**Annotation**
Glyma.04g004100-Myb4(NM_120023)	348291	Myb transcription factor AtMyb2
Glyma.04g004600-GlyH10	392963	Glycosyl hydrolase family 10
Glyma.04g008300-FBPA	632922	Fructose-biphosphate aldolase 1
Glyma.04g012300-NmrA	910831	NmrA-like negative trancriptional regulator family protein
Glyma.04g018300-OFT	1429143	O-fucosyltransferase family protein
Glyma.04g025700-MT	2075011	Methyltransferase
Glyma.04g028600-BGal.GlyH35	2318784	β-galactosidase 3/Glycosyl hydrolase family 35
Glyma.04g030400-Cell.GlyH5	2466301	Cellulase/ Glycosyl hydrolase family 5
Glyma.04g032600-SIS	2589457	Sugar isomerase (SIS) family protein
Glyma.04g035300-P450	2815996	Cytochrome P450 family 82
Glyma.04g042300-Myb73	3404360	Myb transcription factor Myb73
Glyma.04g046200-LIM(TC204443)	3692249	LIM domain-containing protein
Glyma.04g052100-P450	4228426	Cytochrome P450
Glyma.04g063800-CesA	5293426	Cellulose synthase
Glyma.04G067900-CesA(TC215803)	5686681	Cellulose synthase
Glyma.04g071500-Act	5949695	Actin-related protein
Glyma.04g076500-Csl.C5	6419020	Cellulose synthaselike C5/Glycosyltransferase family 2
Glyma.04g078700-MT	6608679	Methyltransferase
Glyma.04g082100-B13GluT.GlyT43	6930590	β-1,3-Glucuronyltransferase/Glycosyltransferase family 43
Glyma.04g085000-ATH8B(TC275879)	7226748	Homeobox gene 8
Glyma.04g086300-ME	7340889	Malic enzyme
Glyma.04g089000-MatE	7718059	MATE efflux family protein
Satt578	***7891669***	***SSR***
Glyma.04g092400-PGI(BG507569)	8143384	Phosphoglucose isomerase
Glyma.04g092600-1433LA(CA784856b)	8158031	14-3-3 protein-like A
Glyma.04g094100-OFT	8363989	O-fucosyltransferase
Glyma.04g097200-GST	8814118	Gluhathione S-transferase
Glyma.04g099500-Pox	9106497	Peroxidase

Glyma.04g099900-1433LA(CA784856a)	9132954	14-3-3 protein-like A
Glyma.04g100700-Pox	9267532	Peroxidase
Glyma.04g103100-MT	9554862	Methyltransferase
Glyma.04g107500-GST	11317835	Gluhathione S-transferase
Glyma.04g109600-ADH	11826869	Alcohol dehydrogenase
Glyma.04g110100-Myb119	12068771	Myb transcription factor Myb119
Glyma.04g110400-P450	12174525	Cytochrome P450
Glyma.04g111300-Cell.GlyH9	12306456	Cellulase/Endo-1,4-β-glucanase/Glycosyl hydrolase family 9
Glyma.04g121400-BGal.GlyH35	15251488	β-galactosidase 3/Glycosyl hydrolase family 35
Glyma.04g126500-ABCtr	17010198	ABC transporter
Glyma.04g131100-NmrA	18387518	NmrA-like negative transcriptional regulator family protein
Glyma.04g138200-GST	21394530	Gluhathione S-transferase
Glyma.05g141700-Myb305	23929298	Myb transcription factor Myb305
Glyma.04g142300-AcylT.NAT	24723349	Acyl-CoA N-acyltransferase (NAT) superfamily protein
Glyma.04g142700-Csl	24964360	Cellulose synthase-like
Glyma.04g143200-GlyH28	25762308	Glycosyl hydrolase family 28/Pectin lyase-like superfamily protein
Glyma.04g153700-CesA	35198918	Cellulose synthase
Glyma.04g160100-COBL(TC266672)	39659936	COBRA-like extracellular glycosyl-phsphatidyl inositol-anchored protein
Glyma.04g173700-Csl	43512293	Cellulose synthase-like
Glyma.04g187300-Myb103(EV271766)	45788984	Myb transcription factor Myb103
Glyma.04g227700-COMT(TC226264)	49704598	O-methyltransferase
Glyma.04g240800-ADH(AF079058)	50907553	Alcohol dehydrogenase
Glyma.04g255400-Csl	52186009	Cellulose synthase-like

Chromosome Gm05[d] (1-42234498)

Feature	Position	Annotation
Glyma.05g160000-Csl	35169849	Cellulose synthase-like
Glyma.05g187300-CesA	37355209	Cellulose synthase
Glyma.05g187700-CAD	37387615	Cinnamyl alcohol dehydrogenase

Chromosome Gm06 (1-51416486)

Feature	Position	Annotation
Glyma.06g015000-STP(CA785365a)	1127434	Sugar transport protein
Glyma.06g065000-CesA(BE806202)	4944119	Cellulose synthase
Glyma.06g069600-CesA	5338316	Cellulose synthase
Satt281	***6529270***	***SSR***
Satt422	***7233361***	***SSR***
Glyma.06g094400-1433LB(CA785232a)	7432085	14-3-3 protein-like B
Glyma.06g143300-EXP(CA785153a)	11662423	Expansin
Glyma.06g186300-ALDH(BG405171)	16211217	Aldehyde dehydrogenase 10A8
Satt277	***17218677***	***SSR***

Glyma.06g195000-EXP(CA785153b)	17424362	Expansin
Glyma.06g202400-F3'H	18785415	Flavonoid 3' hydroxylase/Cytochrome P450
Glyma.06g205400-COBL(TC266672)	19413460	COBRA-like extracellular glycosyl-phsphatidyl inositol-anchored protein
Glyma.06g205500-COBL4.IRX6	19443188	COBRA-like extracellular glycosyl-phsphatidyl inositol-anchored protein 4
Glyma.06g207300-GlyH28	20011530	Glycosyl hydrolase family 28/Pectin lyase-like superfamily protein
Glyma.06g213400-Myb	21679889	Myb-like transcription factor
Glyma.06g215200-P450	22278471	Cytochrome P450
Satt489	***23848501***	***SSR***
Glyma.06g221700-Pox	28139596	Peroxidase
Glyma.06g222500-ADH	29083216	Alcohol dehydrogenase
Glyma.06g225400-CesA(TC219882c)	31081545	Celulose synthase
Glyma.06g225500-CesA(TC219882a8)	31115900	Celulose synthase
Glyma.06g227300-P450	34434861	Cytochrome P450
Glyma.06g231500-MalD	36232185	Lactate/malate dehydrogenase family protein
Glyma.06g238500-P450	38999067	Cytochrome P450
Glyma.06g240600-GlyT28	39729921	Glycosyltransferase family 28
Glyma.06g248200-Myb305	41892792	Myb transcription factor Myb305
Satt079	***44503658***	***SSR***
Glyma.06g260300-AKR	44535490	Aldo/keto reductase family
Glyma.06g269300-ABCtr	45811991	ABC transporter family protein
Glyma.06g275900-Pox	46685943	Peroxidase
BARC-Satt307	***46820581***	***SSR***
Glyma.06g277100-E14BGlu.GlyH9	46831714	Endo-1,4-β-glucanase/Glycosyl hydrolase 9B18
Glyma.06g284300-LAC	47271762	Laccase/Diphenol oxidase family protein
Glyma.06g286700-OMT	47527698	O-methyltransferase family protein
Satt316	***48016322***	***SSR***
Glyma.06g292400-Cell.GlyH5	48130296	Cellulase/Glycosyl hydrolase family 5
Glyma.06g296300-CWall(CA801101)	48530486	Cell wall protein precursor
Glyma.06g302300-XET	49159813	Xyloglucan endo-transglycosylase 6 (XET) /Glycosyl hydrolase family 16
Glyma.06G302600-Pox(AF019116a)	49188759	Peroxidase
Glyma.06G302700-Pox(AF019116b)	49199850	Peroxidase
Glyma.06g307900-Csl	49664901	Cellulose synthase-like
Glyma.06g310800-P450	49935880	Cytochrome P450
Satt357	***50509593***	***SSR***
Glyma.06g316700-CesA(TC219882e)	50555072	Cellulose synthase
Glyma.06g324300-Csl	51189321	Cellulose synthase-like

Chromosome Gm07 (1-44630646)

**Feature**	**Position**	**Annotation**
Satt590	***1301315***	***SSR***
Glyma.07g018000-Aqp	1437693	Aquaporin (major intrinsic family protein)
Glyma.07g019100-ACoAS	1511438	Acyl-CoA synthetase
Glyma.07g020500-GlyT14	1603103	Glycosyltransferase family 14/ Core-2/I-branching β-1,6-N-acetylglucosaminyltrans.

Glyma.07g021600-HCT(TC215709)	1673827	Hydroxycinnamoyl-CoA shikimate/quinate hydroxycinnamoyl transferase
Glyma.07g022300-FT	1718519	Fucosyltransferase 1
Glyma.07g023700-CCR	1825609	Cinnamoyl CoA reductase 1
Glyma.07g024400-Aqp	1891263	Aquaporin (major intrinsic family protein)
Satt201	***2025244***	***SSR***
Glyma.07G026300-CCR2(TC219427)	2055071	Cinnamoyl CoA reductase 2
Glyma.07g028600-SK	2298893	Sugar kinase
Glyma.07g030400-GlyH17	2425523	Glycosyl hydrolase family family 17
Glyma.07g031800-CFiber	2511998	Cotton fiber expressed protein
Glyma.07g034500-GlyT1	2734294	Glycosyltransferase family 1
Glyma.07g037700-Myb32	3110543	Myb transcription factor Myb32
Glyma.07g038800-GLP	3199585	Germin-like protein 3/Cupin
Glyma.07g039800-F3'H.TT7	3276016	Flavonoid 3' hydroxylase AtTT7/Cytochrome P450fuperfamily
Glyma.07g046500-OFT	3894897	O-fucosyltransferse family protein
Glyma.07g048900-OMT	4156669	O-methyltransferase 1
Glyma.07g049900-GlyT1	4243258	Glycosyltransferase family 1
Glyma.07g050600-NAM.NST1(TC268990)	4330571	NAC (No Apical Meristem) domain transcription regulator superfamily protein
Glyma.07g051500-bHLH	4449602	Basic helix-loop-helix (bHLH) DNA-binding family protein/Myc
Glyma.07g052300-P450	4557779	Cytochrome P450
Satt567	***4559602***	***SSR***
Glyma.07g175200-CAD	31708738	Cinnamyl alcohol dehydrogenase
Glyma.07g202000-Csl	37098709	Cellulose synthease-like
Glyma.07g214700-CCoAOMT(TC204613)	38691727	Caffeoyl-CoA O-methyltransferase
Glyma.07g254600-UDPGlyT(TA59613a)	43138637	UDP-glycosyltransferase superfamily protein

Chromosome Gm08 (1-47837940)

**Feature**	**Position**	**Annotation**
Glyma.08g031300-Myb63(At1g79180b)	2473542	Myb transcription factor Myb63
Glyma.08g088400-CesA	6687539	Cellulose synthase
Glyma.08g109400-CHS(X54644a1)	8391364	Chalcone synthase
Glyma.08g109500-CHS(X54644a2)	8397944	Chalcone synthase
Glyma.08g110700-CHS4(X52097a)	8513952	Chalcone synthase 4
Glyma.08g117500-CesA	9065033	Cellulose synthase
Satt187	***9192408***	***SSR***
Glyma.08g120100-Aqp	9255018	Aquaporin (major intrinsic family protein)
Glyma.08g121200-XET	9361001	Xyloglucan endo-transgkycosylase (XET) /Glycosyl hydrolase family 16
Glyma.08g122600-AKR	9445153	Aldo/keto reductase family
Glyma.08g125100-P450	9605622	Cytochrome P450
Glyma.08g126500-CesA	9741612	Cellulose synthase
Glyma.08g130600-Cell.GlyH5	10025431	Cellulase/Glycosyl hydrolase family 5
Glyma.08g132300-GlyT17	10137243	Glycosyltransferase family 17/β-1,4-N-acetylglucosaminyltransferase family
Glyma.08g140400-P450	10742008	Cytochrome P450
Glyma.08g140500-F3'H.TT7	10748126	Flavonoid 3' hydroxylase AtTT7/Cytochrome P450

Glyma.08g140600-F5H	10751884	Ferulic acid 5-hydroxylase 1/Cytochrome P450
Glyma.08g145600-CesA	11066152	Cellulose synthase
Glyma.08g145900-CAD	11092669	Cinnamyl alcohol dehydrogenase
Glyma.08g149300-GlyH28	11441237	Glycosyltransferase family 28/Polygalacturonase (pectinase) family
Glyma.08g150500-BGlu.GlyH1	11575936	β-glucosidase family 17/Glycosyl hydrolase family 1
Glyma.08g162700-Pox	12736920	Peroxidase
Glyma.08g163200-Myb67	12800182	Myb transcription factor Myb67
Glyma.08g168000-Pox	13238337	Peroxidase
Glyma.08g168100-Myb63(At1g79180a)	13246042	Myb transcription factor AtMyb63
Glyma.08g173400-NAM.NAC1	13802693	NAC domain containing protein 1
Glyma.08g174600-GST	13954182	Glutathione S-transferase
Glyma.08g175700-OMT	14032115	O-methyltransferase
Glyma.08g179600-Pox	14396789	Peroxidase
Glyma.08g180900-BDXyl.GlyH3	14516542	β-D-xylosidase 4/Glycosyl hydrolase family 3
Glyma.08g181100-NAM.ANAC104	14538880	NAC domain protein ANAC104/Xylem NAC domain 1
Glyma.08g182100-P450	14605001	Cytochrome P450
Glyma.08g182300-F16BP	14620291	Fructose-1 6-bisphosphatase
Sat_129	***14660743***	***SSR***
Glyma.08g187500-GlyT8	15017217	Glycosyltransferase family 8
Satt089	***15233696***	***SSR***
Glyma.08g220200-HCT(AK286844)	17889781	Hydroxycinnamoyl-CoA shikimate/quinate hydroxycinnamoyl transferase
Glyma.08g275700-CAD	36660183	Cinnamyl alcohol dehydrogenase
Glyma.08g276100-CAD	36778270	Cinnamyl alcohol dehydrogenase
Glyma.08g280500-CAD	38102015	Cinnamyl alcohol dehydrogenase
Glyma.08g330600-Csl	44828516	Cellulose sunthase-like
Glyma.08g330700-Csl	44841021	Cellulose sunthase-like

Chromosome Gm09 (1-50189764)

**Feature**	**Position**	**Annotation**
Glyma.09g017000-GlyT8(AK244415)	1320193	Glycosyltransferase family 8/Galacturonosyltransferase 4
Glyma.09g018500-E14Glu.GlyH9(TC225216)	1452953	Endo-1,4-βglucanase/Glycosyl hydrolase 9A1
Glyma.09g022300-Pox(L81148)	1786058	Peroxidase
Glyma.09g022900-Pox	1830536	Peroxidase
Glyma.09g023411-BGEH.GlyH3(BG507311)	1871307	β-glucan exohydrolase/Glycosyl hydrolase family 3
Satt349	***2399604***	***SSR***
Glyma.09g029400-P450	2404388	Cytochrome P450
Glyma.09g029900-ACoAS	2475075	Acyl-CoA synthetase
Glyma.09g032100-Myb78	2673509	Myb transcription factor Myb78
Glyma.09g033700-INV.PMEI	2822619	Invertase/Pectin methylesterase inhibitor superfamily protein
Glyma.09g036000-ALDH	3014591	Aldehyde dehydrogenase
Glyma.09g036200-INV.PMEI	3032023	Invertase/Pectin methylesterase inhibitor superfamily protein
Glyma.09g037100-GlyH17	3093741	Glycosyl hydrolase family 17
Glyma.09g038500-CTL2	3219573	Chitinase-like protein 2

Glyma.09g041600-PG	3509893	Polygakacturonase 4/Glycosyl hydrolase family 28
Glyma.09g042700-MatE	3653337	MATE efflux family protein
Glyma.09g044500-ABCtr	3801066	ABC transporter
Glyma.09g048400-Pox	4181332	Peroxidase
Glyma.09g048700-P450	4210666	Cytochrome P450
Glyma.09g051100-CesA(TC215151b)	4454515	Cellulose synthase
Glyma.09g057100-Pox	5155538	Peroxidase
Glyma.09g060200-bHLH	5571201	Basic helix-loop-helix (bHLH) DNA-binding superfamily/Myc
Glyma.09g061300-OFT	5791276	O-fucosyltransferase family protein
Glyma.09g062600-XET(TA43776)	5956760	Xyloglucan endo-transglycosylase 6 (XET) /Glycosyl hydrolase family 16
Glyma.09g062900-BGal(TC322276)	6004419	β-galactosidase
Glyma.09g063300-CFiber	6074289	Cotton fiber expressed protein
Glyma.09g065800-Pox	6415366	Peroxidase
Glyma.09g069200-OFT	7043737	O-fucosyltransferase family protein
Glyma.09g070300-ALDH(AY250704)	7178794	Aldehyde dehydrogenase
Glyma.09g071500-GlyH28	7309029	Glycosyl hydrolase family 28/Pectin lyase-like superfamily
Glyma.09g072700-INV.PMEI	7619132	Invertase/Pectin methylesterase inhibitor superfamily protein
Glyma.09g073600-SS	7809852	Sucrose synthase
Sat_119	***7879934***	***SSR***
Glyma.09g092700-PRP(J02746)	12673090	Proline rich protein/Extensin-like protein repeat
Glyma.09g093000-RPRP(X16574)	12819908	Repetitive proline rich protein
Glyma.09g103000-CesA(TC264440)	19046132	Cellulose synthase
Glyma.09g119000-Csl	28442074	Cellulose synthase-like
Glyma.09g201100-CAD	42535148	Cinnamyl alcohol dehydrogenase
Glyma.09g201200-CAD	43238381	Cinnamyl alcohol dehydrogenase
Glyma.09g208200-Csl	43253360	Cellulose synthase-like
Glyma.09g278000-NLTP(CA784306)	49336440	Non specific lipid transfer protein

Chromosome Gm10 (1-51566898)

Feature	Position	Annotation
Satt358	***1018481***	***SSR***
Glyma.10g012100-INV.PMEI	1116279	Invertase/Pectin methylesterase inhibitor superfamily protein
Glyma.10g013900-BXyl.GlyH3	1235166	β-xylosidase 2/Glycosyl hydrolase family 3
BARC-Sat_132	***1303506***	***SSR***
Glyma.10g014600-Myb12	1304361	Myb domain protein 12
Glyma.10g015000-CFiber	1334304	Cotton fiber expressed protein
Glyma.10g015900-GlyT8	1401653	Glycosyltransferase family 8
Glyma.10g016100-GlyH28	1448652	Glycosyl hydrolase family 28/Pectin lyase-like superfamily
Glyma.10g016900-PG	1490555	Polygalacturonase 4/Glycosyl hydrolase family 28
Glyma.10g017000-E14BGlu	1495686	Endo-1,4-β-glucanast/Glycosyl hydrolase family 9
Glyma.10g017800-Tub	1562191	Tubulin
Glyma.10g019000-ABCtr	1633934	ABC transporter
Glyma.10g019900-GST	1736235	Glutathione S-transferase

Glyma.10g022500-Pox	1953439	Peroxidase
Glyma.10g026000-bHLH.TT8	2262144	Basic helix-loop-helix (bHLH) DNA-binding superfamily/Myc
Satt445	***2662079***	***SSR***
Glyma.10g039600-Csl	3498156	Cellulose synthase-like
Glyma.10g058200-PAL2(TC225162)	5328963	Phenyalanine ammonia lyase 2
Satt188	***30401139***	***SSR***
Glyma.10g119400-Cuc(TC293154)	30453912	Cucumisin/Subtilisin-like serine endopeptidase family protein
Glyma.10g120100-S6PPH	30587542	Sucrose-6F-phosphate phosphohydrolase
Glyma.10g122300-EXP	31342355	Expansin
Glyma.10g124800-AKR	32580232	Aldo/keto reductase family
Glyma.10g125000-PL	32669281	Pectate lyase
Glyma.10g126400-MADS.AGL6	33568320	MADS box protein/AGAMOUS 6
Glyma.10g127100-GlyT14	33821417	Glycosyltransferase 14/Core-1/I-branching β-1,6-N-acetylglucosaminyltrans.
Glyma.10g128800-B12AGT	34258487	β-1,2-N-acetylglucosaminyltransferase II
Glyma.10g129800-WD40	34791494	Transducin family protein/WD40 repeat family protein
Glyma.10g132200-Myb79	35607026	Myb domain protein 79
Satt173	***36986811***	***SSR***
Glyma.10g139700-GLP(TA51907a)	37334283	Germin-like protein/Cupin
Glyma.10g142100-PAE(CA784831)	37558420	Pectinacetlesterase family protein
Glyma.10g147600-CLT(AB196794)	37786291	Calreticulin
Glyma.10g172100-PCTP(BF009085)	40573282	Phosphatidylcholine transfer protein
Glyma.10g189300-Csl	42237298	Cellulose synthase-like
Glyma.10g214700-INV(AJ717412)	44674211	Invertase
Glyma.10g216400-NAM.VND7.BT026447(At1g71930)	44817031	NAM protein/vascular related NAC-domain protein 7
Glyma.10g217800-FT	44944812	Fucosyltransferase
Glyma.10g219100-LAC	45096674	Laccase/Diphenol oxidase family protein
Glyma.10g219600-NAM.NAC017	45126440	NAM protein NAC017
Glyma.10g219800-Pox	45139887	Peroxidase
Glyma.10g223000-INV.PMEI	45400648	Invertase/Pectin methylesterase inhibitor superfamily protein
Glyma.10g223500-Csl	45445164	Cellulose synthase-like
Glyma.10g227700-CT	45817689	Chitinase A/Glycosyl hydrolase 18
Glyma.10g230200-WRKY27	46024155	WRKY DNA-binding protein
Glyma.10g231100-GlyH28	46085150	Glycosyl hydrolase family 28/Pectin lyase-like superfamily
Glyma.10g232200-MatE	46170280	MATE efflux family protein
Glyma.10g234700-P450	46404476	Cytochrome P450
Glyma.10g236600-Myb45	46549220	Myb domain protein 45
Satt243	***46657863***	***SSR***
Glyma.10g239000-MatE	46755702	MATE efflux family protein
Glyma.10g240300-Pox	46878808	Peroxidase
Glyma.10g240500-MADS(TC298698)	46884602	MADS box protein
Glyma.10g240800-CK.pfkB	46921096	pfkB family carbohydrate kinase
Glyma.10g243700-AKR	47213119	Aldo/keto reductase family
Glyma.10g245900-BGal	47426255	β-galactosidase

Glyma.10g251700-XET	47980543	Xyloglucan endo-transglycosylase 6 (XET) /Glycosyl hydrolase family 16
Glyma.10g262400-CAD(TC20444-1)	48108667	Cinnamyl alcohol dehydrogenase
Glyma.10g260900-FUC	48615657	1,2-α-L fucosidase/Glycosyl hydrolase
Glyma.10g262400-CAD	48707980	Cinnamyl alcohol dehydrogenase
Glyma.10g264600-GlyT14	48877329	Glycosyltransferase 14/Core-1/I-branching β-1,6-N-acetylglucosaminyltrans.
Glyma.10g273100-MBOAT	49554256	Membrane bound O-acyl transferase (MBOAT) family protein
Glyma.10g275600-C4H	49830805	Cinnamate 4-hydroxylase/Cytochrome P450
Glyma.10g277200-PL	49938040	Pectate lyase
Glyma.10g291100-GST	50911211	Glutathione S-transferase
Glyma.10g292200-CHI.TT5	51006927	Chalcone-flavanone isomerase
Glyma.10g296400-P450	51323582	Cytochrome P450

Chromosome Gm11 (1-34766867)

Feature	Position	Annotation
Glyma.11g010400-Csl	725625	Cellulose synthase-like
Glyma.11g021100-Cell.E14BGlu(U00730)	1503824	Endo-1,4-β-glucanast/Glycosyl hydrolase family 9
Glyma.11g021600-Myb103(AY519564)	1538603	Myb domain protein 103
Glyma.11g027100-HD.KNAT7(TC274106)	1947017	KNOTTED-like homeobox 7 (KNAT7)
Glyma.11g030200-Myb85(At4g22680a)	2188990	Myb domain protein 85
Glyma.11g054500-CCoAOMT(TC225342)	4104145	Caffeoyl-CoA O-methyltransferase
Glyma.11g059200-PE(BE805968a)	4474609	Pectin esterase/Multicopper oxidase
Glyma.11g074100-F5H(TC217041)	5535750	Ferulic acid 5-hydroxylase/Cytochrome P450
Glyma.11g108900-CK.pfkB(BG507617)	8299154	pfkB family carbohydrate kinase
Glyma.11g112600-XK(CA799181)	8600895	Xylulose kinase 1/Sugar kinase
Satt197	***8898878***	***SSR***
Glyma.11g117200-Myb6	8920043	Myb domain protein 6
Glyma.11g119600-STP	9127497	Sugar transport protein
Glyma.11g122700-P450	9354319	Cytochrome P450
Glyma.11g124500-CT	9466533	Chitinase
Glyma.11g130100-BGlu	9899417	β-glucosidase 17/Glycosyl hydrolase family 1
Glyma.11g133300-ALDH	10188790	Aldehyde dehydrogenase
Glyma.11g137500-LAC	10456742	Laccase/Diphenol oxidase family protein
Glyma.11g139100-EXP	10591261	Expansin
Glyma.11g145800-HD.IFL1(TC271859)	11229131	Homeobox-leucine zipper family protein/Lipid binding START domain-containing
Glyma.11g147900-BGal	11401551	β-galactosidase 8/Glycosyl hydrolase family 2
Glyma.11g150800-OMT	11842709	O-methyltransferase
Glyma.11g151800-Csl	12025822	Cellulose synthase-like
Glyma.11g154900-EXT	12643089	Extensin
Glyma.11g155700-MOG	12781732	Mannosyl oligosaccharide glucosidase
Glyma.11g157800-PL.GlyH28	14096320	Glycosyl hydrolase family 28/Pectin lyase-like superfamily
Glyma.11g159700-MADS.AGL80	14448495	MADS box protein/AGL80
Glyma.11g161800-Pox	14983400	Peroxidase
Glyma.11g163300-WRKY33	15312631	WRKY DNA-binding protein 33

Glyma.11g165100-GlyH17	15722988	Glycosyl hydrolase family 17
Glyma.11g166600-SHT	16173269	Spermidine hydroxycinnamoyl transferase
Sat_095	***16478273***	***SSR***
Glyma.11g167400-bZIP	16491689	Basic-leucine zipper (bZIP) transcription factor
Glyma.11g168800-GNAT	17551481	Acetyltransferase (GNAT) family
BARC-Satt583	***18261607***	***SSR***
Glyma.11g172000-FBP	18690585	Fructose-2,6-biphosphatase
Glyma.11g174600-ALDH	19129385	Aldehyde dehydrogenase 10A9
Glyma.11g175900-P450	20343531	Cytochrome P450
Glyma.11g176400-GlyH3	21280974	Glycosyl hydrolase family 3
Glyma.11g182000-NAM.NAC090	24908076	NAM protein NAC090
Glyma.11g183200-GlyH17	25070908	Glycosyl hydrolase family 17
Glyma.11g186400-MADS.AGL80	25583936	MADS box protein/AGL80
Glyma.11g189600-Csl.A02(BG507518)	26169102	Cellulose synthase-like
Glyma.11g193600-XET	26698651	Xyloglucan endo-transglycosylase 6 (XET) /Glycosyl hydrolase family 16
Glyma.11g194500-4CL	26790441	Acyl-CoA synthetase
Glyma.11g197100-Pox	27196300	Peroxidase
Glyma.11g210600-PL	30326671	Pectate lyase
Glyma.11g212400-NAM.NAC028	30521509	NAM protein NAC028
Glyma.11g212700-SS	30547238	Sucrose synthase
Glyma.11g215800-Myb20	30994324	Myb domain protein 20
Glyma.11g216400-GST	31056913	Glutathione S-transferase
Glyma.11g223700-GPAT	31857860	Glucose-1-phosphate adenyltransferase family protein
Glyma.11g226900-FBP	32185432	Fructose-1, 6-biphosphatase
Sat_123	***32194459***	***SSR***
Glyma.11g227100-FBP	32199169	Fructose-1, 6-biphosphatase
Glyma.11g228900-Pox	32424578	Peroxidase
Glyma.11g231300-bHLH	32690044	Basic helix-loop-helix (bHLH) DNA-binding superfamily/Myc
Glyma.11g232400-MT	32780782	Methyltransferase
Glyma.11g233400-LAC	32873226	Laccase/Diphenol oxidase family protein
Glyma.11g243700-OFT	33750461	O-fucosyltransferase family protein
Glyma.11g244900-PL	33856985	Pectate lyase
Satt453	***34173104***	***SSR***

Chromosome Gm12 (1-40091314)

Feature	Position	Annotation
Glyma.12g004900-NAM.VND7.At5g62380(BT026510)	376442	NAC (No Apical Meristem) domain transcription regulator superfamily protein
Glyma.12g017600-Csl	1232608	Cellulose synthase-like
Glyma.12g019700-CCR(TC276003)	1380962	Cinnamoyl CoA reductase
Satt353	***1682474***	***SSR***
Glyma.12g023300-MCO	1711347	SKU5 similar5/Multicopper oxidase
Glyma.12g023600-Aqp	1733920	Aquaporin (major intrinsic family protein)
Glyma.12g024000-INV	1748002	Invertase

Glyma.12g027200-B14BGlu.GlyH9	1961942	Glycosyl hydrolase family 9
Glyma.12g029600-P450	2208804	Cytochrome P450
Glyma.12g030700-DnaJ	2284184	DnaJ heat shock protein
Glyma.12g032200-Myb32	2416369	Myb domain protein 32
Glyma.12g032300-Pox	2420748	Peroxidase
Glyma.12g032600-BGal.GlyH35	2460810	β-galactosidase/ Glycosyl hydrolase family 35
Glyma.12g033100-WD40	2498908	transducin family protein/WD40 repeat family protein
Glyma.12g036700-AKR	2665303	Aldo/keto reductase family
Glyma.12g036900-SNEr	2671112	Sugar nucleotide epimerase related
Glyma.12g037400-FBPA	2709372	Fructose biphosphatase aldolase
Glyma.12g038500-XK	2776328	Xylulose kinase 1/Sugar kinase
Glyma.12g039600-CaM	2864627	Calmodulin binding protein
Glyma.12g042400-GPAT	3059633	Glucose-1-phosphate adenylyltransferase family protein
Glyma.12g042700-NmrA	3085800	NmrA-like negative transcriptional regulator family protein
Glyma.12g042900-Myb	3114697	Myb domain protein
Glyma.12g043000-GRP(AF169205)	3122454	Glycine-rich protein
Glyma.12g043800-GlyH17	3175262	Glycosyl hydrolase family 17
Glyma.12g057900-Myb46.At5g12870(BT002549a)	4209199	Myb domain protein 46
Glyma.12g069600-AGP(CA801105b)	5085478	FASCICLIN-like arabinogalactan protein
Glyma.12g080100-XET(TCC2266542b)	6295519	Xyloglucan endo-transglycosylase 6 (XET) /Glycosyl hydrolase family 16
Glyma.12g096900-Csl	8237882	Cellulose synthase-like
Glyma.12g173800-AGP(CA801105a)	33069515	FASCICLIN-like arabinogalactan protein
Glyma.12g191500-Csl	35302118	Cellulose synthase-like
Glyma.12g191700-Csl	35324238	Cellulose synthase-like
Glyma.12g191800-Csl	35354173	Cellulose synthase-like
Glyma.12g192000-Csl	35364680	Cellulose synthase-like
Glyma.12g192100-Csl	35369755	Cellulose synthase-like
Glyma.12g215800-CCR	37494356	Cinnamoyl CoA reductase
Glyma.12g237000-CesA(TC270909)	39586913	Cellulose synthase
Glyma.12g237500-Myb46.At5g12870(BT002549b)	39643857	Myb domain protein 46

Chromosome Gm13 (1-45874162)

Feature	Position	Annotation
Satt569	***12294072***	***SSR***
Glyma.13g040900-GST	13017722	Glutathione S-transferase
Glyma.13g044100-AcylT	13758232	HXXXD-type acyltransferase family protein
Glyma.13g045700-Mann6PI	13984814	Mannose-6-phosphate isomerase
Glyma.13g046400-P450	14071533	Cytochrome P450
Glyma.13g048600-GlyH81	14456516	Glycosyl hydrolase family 81
Glyma.13g050400-Myb83	14652395	Myb domain protein 83
Glyma.13g051600-P450	14805645	Cytochrome P450
Glyma.13g052100-MADS.AGL8	14902730	MADS box protein/AGL8
Glyma.13g053300-COBL	15064460	COBRA-like extracellular glycosyl-phsphatidyl inositol-anchored protein

Glyma.13g054000-AcylT	15126544	HXXXD-type acyltransferase family protein
Glyma.13g055300-OFT	15237358	O-fucosyltransferase family protein
Glyma.13g056600-UDPGlyT	15417552	UDP-glycosyltransferase
Glyma.13g056900-WD40	15439351	Transducin family protein/WD40 repeat family protein
Glyma.13g057800-GlyT35	15537334	Glycosyltransferase 35
Glyma.13g060900-PME31	15841355	Pectin methylesterase 31
Glyma.13g061400-UDPGlyT74	15869684	UDP-glycosyltransferase 74
Glyma.13g062000-NAM.NAC100	15953721	NAC (No Apical Meristem) domain transcription regulator superfamily protein
Glyma.13g064700-PL	16424596	Pectate lyase
Satt252	***16454986***	***SSR***
Glyma.13g066900-MT	16662928	Methyltransferase
Glyma.13g067900-GluT8	16787469	Glucosyltransferase family protein 8
Glyma.13g068500-P450	16851649	Cytochrome P450
Glyma.13g069400-Pox	16923989	Peroxidase
Glyma.13g070300-Csl.C4	17038776	Cellulose synthase-like C4
Glyma.13g070600-OMT	17100410	O-methyltransferase
Glyma.13g073200-GST	17473325	Glutathione S-transferase
Glyma.13g073400-Myb33	17553306	Myb domain protein 33
Glyma.13g074800-CaM	17765288	Calmodulin binding protein
Satt160	***17875691***	***SSR***
Glyma.13g080200-PL	18671747	Pectate lyase
Glyma.13g082300-FLS1	19154278	Flavonol synthase 1
Glyma.13g083000-OFT	19257367	O-fucosyltransferase family protein
Glyma.13g084600-GalT	19574202	Galacturonosyltransferase
Glyma.13g086400-MADS.AGL104	19857225	MADS box protein/AGL104
BARC-Sat_039	***19958451***	***SSR***
Glyma.13g126000-Csl	23922920	Cellulose synthase-like
Glyma.13g161600-SPS(TC286435)	27715861	Sucrose phosphate synthase/Glycosyltransferase family 1
Glyma.13g174300-Csl	28844973	Cellulose synthase-like
Glyma.13g174700-NAM.SND2.DQ056658(At4g28500a)	28912927	NAC (No Apical Meristem) domain transcription regulator superfamily protein
Glyma.13g202500-CesA(TC270909)	31619752	Cellulose synthase
Glyma.13g203800-DFR	31789318	Dihydroflavonol 4-reductase
Glyma.13g203900-DFR	31793697	Dihydroflavonol 4-reductase
BARC-Sat_120	***32029622***	***SSR***
Glyma.13g206700-Glu6Pt	32050467	Glucose-6-phosphate/phosphate translocator
Glyma.13g210100-HRGP	32392402	Hydroxyproline-rich glycoprotein family protein
Glyma.13g213100-WD40	32664808	transducin family protein/WD40 repeat family protein
Glyma.13g217400-P450	33038297	Cytochrome P450
Glyma.13g217900-AMann3	33096818	α-manosidase 3
Glyma.13g218300-GlyH	33167113	Glycosyl hydrolase family 28/Pectin lyase-like superfamily
Glyma.13g221000-GalT	33409269	Galacturonosyltransferase
Glyma.13g226000-AGP11	33868588	FASCICLIN-like arabinogalactan protein 11
Glyma.13g226800-Myb6	33942335	Myb domain protein 6

Glyma.13g228200-OFT	34046523	O-fucosyltransferase family protein
Glyma.13g232400-AcylT	34353109	HXXXD-type acyltransferase family protein
Glyma.13g234700-NAM.SND3.NM_102615(At1g28470)	34537748	NAC domain transcription regulator superfamily protein SND3
Glyma.13g255300-CAD	36108674	Cinnamyl alcohol dehydrogenase
Glyma.13g270600-1433LD(BG405219b)	37265741	14-3-3 protein-like D
Glyma.13g285700-CCR(TC229375)	38657018	Cinnamoyl CoA reductase
Glyma.13g290900-1433LD(BG405219a)	39120795	14-3-3 protein-like D
Glyma.13g294000-NAM.SND2.DQ056658(At4g28500c)	39352609	NAC domain transcription regulator superfamily protein SND2
Glyma.13g309300-PPD(EH262010)	40514843	Prephenate dehydratease
Glyma.13g310300-Csl	40581901	Cellulose synthase-like
BARC-Sat_090	***42696818***	***SSR***
Glyma.13g333200-Myb48	42713045	Myb domain protein 48
Glyma.13g336600-EXP.A4	42991934	Expansin
Glyma.13g339400-UDPGlyT	43148955	UDP-glycosyltransferase
Glyma.13g340000-ALDH.3F1	43201486	Aldehyde dehydrogenase
Glyma.13g343000-BGal32	43447268	β-galactosidase
Glyma.13g343500-OMT	43488830	O-methyltransferase
Glyma.13g344500-CK.pfkB	43554239	pfkB family carbohydrate kinase
Glyma.13g346100-Pox	43666720	Peroxidase
Glyma.13g349300-GlyH32	43903578	Glycosyl hydrolase family 32
Glyma.13g350700-BGal(TC330147)	44006167	β-galactosidase
Glyma.13g355600-DFRl	44407834	Dihydroflavonol 4-reductase-like
Glyma.13g360500-UDPGlyT	44752357	UDP-glycosyltransferase
BARC-Sat_074	***44850255***	***SSR***
Glyma.13g364700-PL	45100703	Pectate lyase
Glyma.13g365600-GlyH	45166941	Glycosyl hydrolase
Glyma.13g367400-HRGP	45303060	Hydroxyproline-rich glycoprotein family protein
Glyma.13g369800-CCR	45526207	Cinnamoyl CoA reductase
Glyma.13g370100-WRKY40	45564565	WRKY DNA-binding protein 40
Glyma.13g370400-FT2	45595215	Fucosyltransferase 2
Glyma.13g371000-HCT	45626397	Hydroxycinnamoyl-CoA shikimate/quinate hydroxycinnamoyl transferase
Glyma.13g372000-4CL2(AF002259)	45701238	4-coumarate:CoA ligase 2

Chromosome Gm14 (1-49042192)

Feature	Position	Annotation
Glyma.14g012800-Csl	962290	Cellulose synthase-like
Glyma.14g012900-Csl	971984	Cellulose synthase-like
Glyma.14g029200-Csl	2136060	Cellulose synthase-like
Glyma.14g064600-Cuc(TC293154)	5184454	Cucumisin/Subtilisin-like serine endopeptidase family protein
Glyma.14g072700-DFR1(AF167556)	6111578	Dihydroflavonol 4-reductase 1
BARC-Satt070	***17406311***	***SSR***
Glyma.14g121200-ADH1(AF079058a)	17460374	Alcohol dehydrogenase 1
Glyma.14g122400-P450	17832823	Cytochrome P450

Glyma.14g123400-AcylT.NAT	18516789	Acyl-CoA N-acyltransferase (NAT) superfamily protein
Glyma.14g127400-AGO	20399619	Argonaute family protein
Glyma.14g130300-B13G(CA784107)	22190211	β-1,3-glucanase
Glyma.14g132800-MT	22783132	Methyltransferase
Glyma.14g133200-AGP	22963748	FASCICLIN-like arabinogalactan protein
Glyma.14g134300-Pox	23354149	Peroxidase
Glyma.14g134700-CaM	23511818	Calmodulin binding protein
Glyma.14g135400-WRKY11	23729201	WRKY DNA-binding protein 11
Glyma.14g136900-Csl.C5	24378248	Cellulose synthase-like C5
Glyma.14g138300-ArabK(BE806770)	25798322	Arabinose kinase
Glyma.14g139800-ABCtr	27322688	ABC transporter
Glyma.14g140100-NAM.NAC38	27453859	NAC domain transcription regulator superfamily protein NAC38
Glyma.14g143400-HD	29170614	Homeodomain-like superfamily protein
Glyma.14g143600-GST	29278743	Glutathione S-transferase
Glyma.14g143800-GlyS	29323994	Glycogen/starch synthase
Glyma.14g147600-BGlu31	31451522	β-glucosidase 31
Glyma.14g149200-PL	32211624	Pectate lyase
Glyma.14g151500-UDPGluT.85A5	32771539	UDP-glucosyltransferase 85A5
Glyma.14g151900-WallKin2	32912345	Wall-associated kinase 2
Glyma.14g152100-ALDH.3H1	32968712	Aldehyde dehydrogenase
Glyma.14g154400-Myb5	33570229	Myb domain protein 5
Glyma.14g155200-GST	33752073	Glutathione S-transferase
Glyma.14g156400-ADH1(AF079058b)	34149029	Alcohol dehydrogenase 1
Glyma.14g156500-OMT	34251490	O-methyltransferase
Glyma.14g157600-WallKin2	34850397	Wall-associated kinase 2
Glyma.14g159600-PME	36170123	Pectin methylesterase
Glyma.14g160700-bHLH	37455117	Basic helix-loop-helix (bHLH) DNA-binding superfamily/Myc
Glyma.14g161000-AGP16	37916706	FASCICLIN-like arabinogalactan protein 16
Glyma.14g158100-MatE	37998078	MATE efflux family protein
Glyma.14g161500-GST	38193541	Glutathione S-transferase
Glyma.14g163200-GRP	39995831	Glycine-rich protein
Glyma.14g163300-FLS1	40082511	Flavonol synthase 1
Glyma.14g163900-OFT	40415756	O-fucosyltransferase family protein
Glyma.14g166300-GalT	41122482	Galacturonosyltransferase
Glyma.14g166400-UDPGlyT	41133077	UDP-glycosyltransferase
Glyma.14g167300-Gly3PAcylT3	41382512	Glycerol-3-phosphate acyltransferase 3
Glyma.14g168700-MADS.AGL104	41782810	MADS box protein/AGl104
Glyma.14g169900-Myb43	42018793	Myb domain protein 43
Glyma.14g170900-GlyH	42298358	Glycosyl hydrolase
Glyma.14g174500-OMT	43066238	O-methyltransferase
Glyma.14g175000-UDPGluT.85A2	43225518	UDP-glucosyltransferase 85A2
Glyma.14g175200-HRGP	43263918	Hydroxyproline-rich glycoprotein family protein
Glyma.14g177300-OFT	43678017	O-fucosyltransferase family protein

Glyma.14g179600-HD	44161231	Homeodomain-like superfamily protein
Glyma.14g181500-bHLH	44432316	Basic helix-loop-helix (bHLH) DNA-binding family protein/Myc
Glyma.14g183800-MADS.AGL16	44735942	MADS box protein/AGL16
Glyma.14g186100-WRKY70	45021831	WRKY DNA-binding protein 70
Satt534	***45041539***	***SSR***
Glyma.14g187400-WD40	45209096	transducin family protein/WD40 repeat family protein
Glyma.14g189300-NAM.NAC9	45407343	NAC domain transcription regulator superfamily protein NAC9
Glyma.14g191500-LAC	45616466	Laccase/Diphenol oxidase family protein
Glyma.14g191900-GlyH	45655896	Glycosyl hydrolase
Glyma.14g192500-P450	45715135	Cytochrome P450
Glyma.14g193500-CK.pfkB	45834143	pfkB family carbohydrate kinase
Satt063	***45993714***	***SSR***
Glyma.14g195700-Fruc2	46106790	Fructose 2
Glyma.14g197200-bZIP	46214720	Basic-leucine zipper (bZIP) transcription factor
Sat_424	***46281808***	***SSR***
Glyma.14g210700-UDPGluPP(BF009075)	47577189	UDP-glucose pyrophosphorylase
Glyma.14g221200-CAD	48627244	Cinnamyl alcohol dehydrogenase
Glyma.14g223000-AscO(BE806303)	48811743	Ascorbate oxidase/Multicopper oxidase

Chromosome Gm15 (1-51756343)

Feature	Position	Annotation
Glyma.15G003600-CCR(TC226045)	332065	Cinnamoyl CoA reductase
Glyma.15g025500-Myb26(AY519588a)	2064944	Myb domain protein 26
Glyma.15g026300-Lox3.X06928	2123754	Lipoxygenase 3
Glyma.15g026700-EXP.A6	2156473	Expansin
Glyma.15g028200-Pox	2254166	Peroxidase
Glyma.15g029800-FruKin(CA784817)	2387957	O-methyltransferase
Glyma.15g030800-OMT	2466762	O-methyltransferase
Glyma.15g031300-BGlu17	2515687	β-glucosidase 17/Glycosyl hydrolase family 1
Glyma.15g034400-ALDH.3F1	2740960	Aldehyde dehydrogenase
Glyma.15g034500-Myb46(TC250734)	2754696	Myb domain protein 46
Glyma.15g035000-UDPGlyT	2796277	UDP-glycosyltransferase
Glyma.15g040000-UDPXylS5	3158838	UDP-XYL synthase
Glyma.15g044600-WD40	3556280	transducin family protein/WD40 repeat family protein
Glyma.15g049000-bZIP	3866108	Basic-leucine zipper (bZIP) transcription factor
Glyma.15g049100-AcylT	3869436	HXXXD-type acyltransferase family protein
Glyma.15g050800-Pox(AF145349)	3997913	Peroxidase
Glyma.15g051200-XylemNAC	4025824	Xylem NAC domain transcription regulator superfamily protein
Glyma.15g051500-BXyl4	4044021	β-xylosidase 4
Glyma.15g053600-CaM	4209474	Calmodulin binding protein
Glyma.15g054500-UDPGluT.85A2	4274887	UDP-glucosyltransferase 85A2
Glyma.15g057400-HRGP	4428040	Hydroxyproline-rich glycoprotein family protein
Glyma.15g058900-ALDH.6B2	4545863	Aldehyde dehydrogenase

Glyma.15g059500-CAD	4583265	Cinnamyl alcohol dehydrogenase
Glyma.15g059600-MADS.AGL21	4590780	MADS box protein/AGL21
Glyma.15g066800-Myb102	5091082	Myb domain protein 102
Glyma.15g067700-CaM	5161602	Calmodulin binding protein
Satt212	***5240509***	***SSR***
Glyma.15g073100-SSP.TSJT(BF008688)	5604443	Stem-specific protein TSJT
Glyma.15g157100-CesA(TC216172)	13179217	Cellulose synthase
Glyma15g174000-OMT	16104022	O-methyltransferase
Glyma.15g175900-HRGP	16582087	Hydroxyproline-rich glycoprotein family protein
Glyma.15g180200-OFT	17389227	O-fucosyltransferase family protein
Glyma.15g182600-SS(AF030231)	17910131	Sucrose synthase
Glyma.15g184300-INV.PMEI	18368379	Invertase/Pectin methylesterase inhibitor superfamily protein
Glyma.15g186300-WRKY	19145004	WRKY DNA-binding protein
Glyma.15g189000-Pox	19863193	Peroxidase
Glyma.15g196100-PL	22509381	Pectate lyase
Glyma.15g199700-RPRP(X16574)	23940170	Repetitive proline rich protein
Glyma.15g200400-GalT	24455818	Galacturonosyltransferase
Glyma.15g203300-STP	25837465	Sugar transport protein
Glyma.15g206100-CaM	28952274	Calmodulin binding protein
Glyma.15g207100-INV.PMEI	29507153	Invertase/Pectin methylesterase inhibitor superfamily protein
Satt185	***30238139***	***SSR***
Glyma.15g212500-WallKin4	33148044	Wall-associated kinase 4
Glyma.15g212600-GST	33402757	Glutathione S-transferase
Glyma.15g214000-GalO	34266754	Galactose oxidase/Kelch repeat superfamily protein
Glyma.15g218700-WD40	37153830	transducin family protein/WD40 repeat family protein
Glyma.15g220200-HD.GLB2	39672697	Homeodomain-like superfamily protein GLB2
Glyma.15g220700-OMT	39762795	O-methyltransferase
Glyma.15g223500-INV.PMEI	40623676	Invertase/Pectin methylesterase inhibitor superfamily protein
Glyma.15g226200-BGal9	41548120	β-galactosidase 9
Glyma.15g231000-CallS5	43330808	Callose synthase 5
Glyma.15g234600-CaM	44117564	Calmodulin binding protein
Glyma.15g234700-UDPGluT.85A3	44130360	UDP-glucosyltransferase 85A3
Glyma.15g235600-GlyH2	44320970	Glycosyl hydrolase family 2
Glyma.15g236400-HD	44538692	Homeodomain-like superfamily protein
Glyma.15g240900-GalT4	45739945	Galacturonosyltransferase 4
Glyma.15g241000-MRP	45743394	Multidrug resistance-associated protein
Glyma.15g241100-OMT	45747635	O-methyltransferase
Glyma.15g244600-Pox	46599133	Peroxidase
Glyma.15g245800-GluS4	46829270	Glucan synthase-like 4
Glyma.15g250900-OMT	47906601	O-methyltransferase
Glyma.15g251500-GST	47966984	Glutathione S-transferase
Glyma.15g254000-NAM.NAC1	48295488	NAC domain transcription regulator superfamily protein NAC1
Glyma.15g256700-UDPGlyT	48612677	UDP-glycosyltransferase

Glyma.15g259400-Myb63	49185213	Myb domain protein 63
Glyma.15g259500-Pox	49199706	Peroxidase
Glyma.15g263500-ALDH.5F1	49664672	Aldehyde dehydrogenase
Glyma.15g263700-HD	49732962	Homeodomain-like superfamily protein
Glyma.15g264100-NAM.NAC20	49820324	NAC domain transcription regulator superfamily protein NAC20
Glyma.15g265700-BHA2	50090344	β-hexoaminidase 2
Glyma.15g268800-GluS3	50529595	Glucan synthase-like 3
Glyma.15g269400-GlyH28	50637589	Glycosyl hydrolase family 28/Pectin lyase-like superfamily
Sat_124	***50728020***	***SSR***
Glyma.15g270400-OFT	50743973	O-fucosyltransferase family protein
Glyma.15g270900-BGlu	50801893	β-glucosidase
Glyma.15g271000-BGlu(CA802003a)	50823428	β-glucosidase
Glyma.15g275000-CesA	51338547	Cellulose synthase

Chromosome Gm16 (1-37887014)

Feature	Position	Annotation
Glyma.16g019400-NAM.SND1(NM_103011a)	1725643	NAC domain transcription regulator superfamily protein SND1
Satt285	***2827903***	***SSR***
Sct_046	***2919448***	***SSR***
Glyma.16g043200-NAM.NAC012(EU661913a)	4037637	NAC domain transcription regulator superfamily protein NAC012
Glyma.16g045000-XET(D16455a1)	4272035	Xyloglucan endo-transglycosylase 6 (XET) /Glycosyl hydrolase family 16
Glyma.16g075400-STX(AF532627)	7599961	Syntaxin, cell membrane protein
Glyma.16g081000-Csl	8655827	Cellulose synthase-like
Glyma.16g096300-CAD	18250367	Cinnamyl alcohol dehydrogenase
Glyma.16g103900-PLR(AK244769)	21014276	Pinoresinol reductase
Glyma.16g107600-PE	21879441	Pectin esterase
Glyma.16g109100-STP	23030070	Sugar transport protein
Glyma.16g114000-GlyH	25247813	Glycosyl hydrolase
Glyma.16g128700-F3H	28077595	Flavanone 3-hydroxylase
Glyma.16g128800-CHI	28110324	Chalcone-flavanone isomerase
Glyma.16g130200-NAM.SND1(NM_103011b)	28340797	NAC domain transcription regulator superfamily protein SND1
Glyma.16g131200-F5H1	28550384	Ferulic acid 5-hydroxylase 1
Glyma.16g131800-BGal3	28739594	β-galactosidase 3
Glyma.16g132000-GRP	28762615	Glycine-rich protein
Glyma.16g133000-Pox	28949595	Peroxidase
Glyma.16g140900-INV.PMEI	29867884	Invertase/Pectin methylesterase inhibitor superfamily protein
Glyma.16g142700-LIM	30141616	LIM domain-containing protein
Glyma.16g150300-XET(D16455b)	31069867	Xyloglucan endo-transglycosylase 6 (XET) /Glycosyl hydrolase family 16
Glyma.16g150600-AcylT	31096633	HXXXD-type acyltransferase family protein
Glyma.16g151500-NAM.NAC012(EU661913b)	31198908	NAC domain transcription regulator superfamily protein NAC012
Glyma.16g151900-GlyH17	31232074	Glycosyl hydrolase family 17
Glyma.16g152200-HD	31289941	Homeodomain-like superfamily protein
Glyma.16g153600-AGP14	31420465	FASCICLIN-like arabinogalactan protein 14

Glyma.16g154600-FT13	31484987	Fucosyltransferase 13
Glyma.16g155400-AcylT.NAT	31566993	Acyl-CoA N-acyltransferase (NAT) superfamily protein
Glyma.16g162900-XET8	32171560	Xyloglucan endo-transglycosylase 6 (XET) /Glycosyl hydrolase family 16
Glyma.16g164200-Pox	32307939	Peroxidase
Glyma.16g164600-INV.PMEI	32358400	Invertase/Pectin methylesterase inhibitor superfamily protein
Glyma.16g165100-WD40	32435021	transducin family protein/WD40 repeat family protein
Glyma.16g165900-CesA	32487231	Cellulose synthase
Glyma.16g168600-P450	32850506	Cytochrome P450
Glyma.16g172400-MRP	33304594	Multidrug resistance-associated protein
Glyma.16g173000-CT	33365497	Chitinase
Glyma.16g175600-UDPGlyT.88A1	33709232	UDP-glycosyltransferase 88A1
Glyma.16g175700-CesA5	33715208	Cellulose synthase
Glyma.16g175800-GlyH32	33717020	Glycosyl hydrolase family 32
Glyma.16g176700-WRKY27	33803200	WRKY DNA-binding protein 27
Glyma.16g178700-HD	33941617	Homeodomain-like superfamily protein
Glyma.16g179000-PL	33962757	Pectate lyase
Glyma.16g179100-BHX	33969618	β-hydroxylase
Glyma.16g180200-MatE	34077613	MATE efflux family protein
Glyma.16g180500-AcylT	34105756	HXXXD-type acyltransferase family protein
Glyma.16g180600-HRGP	34129875	Hydroxyproline-rich glycoprotein family protein
Glyma.16g182600-P450	34364037	Cytochrome P450
Glyma.16g189400-Myb18	35199461	Myb domain protein 18
Glyma.16g197200-OMT	35854742	O-methyltransferase
Glyma.16g200300-Pox	36137903	Peroxidase
Satt431	***36221174***	***SSR***
Glyma.16g201300-bHLH	36279459	Basic helix-loop-helix (bHLH) DNA-binding superfamily/Myc
Glyma.16g201800-AcylT	36334150	HXXXD-type acyltransferase family protein
Glyma.16g205300-GRP	36621271	Glycine-rich protein
Glyma.16g207500-Pox	36728513	Peroxidase
Glyma.16g209800-AGal2	36908675	α-galactosidase 2
Glyma.16g210000-Aqp	36924622	Aquaporin (major intrinsic family protein)
Glyma.16g210200-HRGP	36933402	Hydroxyproline-rich glycoprotein family protein
Glyma.16g210300-P450	36943073	Cytochrome P450
Glyma.16g210700-OMT	36976580	O-methyltransferase
Glyma.16g212000-UDPGluT.71B6	37055038	UDP-glucosyltransferase 71B6
Glyma.16g217200-SS6	37414228	Sucrose synthase 6
Glyma.16g217400-NAM.SND1(NM_103011d)	37450061	NAC domain transcription regulator superfamily protein SND1
Glyma.16g217800-HD.GLB11	37499534	Homeodomain-like superfamily protein GLB11
Glyma.16g218900-Myb52	37627774	Myb domain protein 52
Glyma.16g219800-WRKY70	37703370	WRKY transcription factor
Glyma.16g220300-GlyH	37739581	Glycosyl hydrolase
Glyma.16g220400-AGal1	37746011	α-galactosidase 1

Chromosome Gm17 (1-41641366)

Feature	Position	Annotation
Glyma.17g064400-4CL1(AF279267b)	4952729	4-coumarate:CoA ligase-like 1
Glyma.17g064500-4CL1(AF279267c)	4974218	4-coumarate:CoA ligase-like 1
Glyma.17g064600-4CL1(AF279267a)	4988012	4-coumarate:CoA ligase 1
Glyma.17g072200-CesA	5646263	Cellulose synthase
BARC-Satt002	***8817625***	***SSR***
Glyma.17g112400-MannT	8864215	β-1,4-mannosyl-glycoprotein/β-1,4-N-acetylglucosaminyltransferase
Glyma.17g118100-P450	9302935	Cytochrome P450
Glyma.17g119200-Myb103	9414944	Myb domain protein 103
Glyma.17g120800-GlyH17	9619511	Glycosyl hydrolase family 17
Sat_092	***9687613***	***SSR***
Glyma.17g125200-P450	9975868	Cytochrome P450
Glyma.17g126500-PL(CA785370)	10078819	Pectate lyase
Glyma.17g128000-MalS	10234685	Malate synthase
Glyma.17g132700-MADS.AGL19	10684402	MADS box protein/AGL19
Glyma.17g133100-EXP.A7	10722246	Expansin
Glyma.17g133800-Myb42	10773369	Myb domain protein 42
Glyma.17g133900-P450	10783983	Cytochrome P450
Glyma.17g134300-UDPGlu4E	10826187	UDP-D-glucuronate 4-epimerase 1
Glyma.17g137400-INV.PMEI	11095069	Invertase/Pectin methylesterase inhibitor superfamily protein
Glyma.17g137500-UDPGlyT	11097469	UDP-glycosyltransferase
Glyma.17g138100-NAM.NAC007	11141925	NAC domain transcription regulator superfamily protein NAC007
Glyma.17g138500-GlyH	11222613	Glycosyl hydrolase
Glyma.17g142600-OFT	11589146	O-fucosyltransferase family protein
Glyma.17g144000-ACoAO	11759547	Acyl-CoA oxidase
Glyma.17g144500-GST	11839475	Glutathione S-transferase
Glyma.17g146900-EXP.B1	12114008	Expansin
Glyma.17g151200-INV.PMEI	12606606	Invertase/Pectin methylesterase inhibitor superfamily protein
Glyma.17g152600-AcylT	12715563	HXXXD-type acyltransferase family protein
Glyma.17g154100-NAM.NAC71	12962459	NAC domain transcription regulator superfamily protein NAC71
Glyma.17g156200-HK	13283810	Hexokinase
Glyma.17g157400-XET	13439996	Xyloglucan endo-transglycosylase 6 (XET) /Glycosyl hydrolase family 16
Glyma.17g158000-Myb84	13527627	Myb domain protein 84
Glyma.17g158900-bHLH	13673011	Basic helix-loop-helix (bHLH) DNA-binding superfamily/Myc
Satt389	***13771477***	***SSR***
Glyma.17g160500-HD	13938649	Homeodomain-like superfamily protein
Glyma.17g161700-AcylT.NAT	14112331	Acyl-CoA N-acyltransferase (NAT) superfamily protein
Glyma.17g162300-P450	14363606	Cytochrome P450
Glyma.17g162700-GST	14474135	Glutathione S-transferase
Glyma.17g166500-CesA	15209217	Cellulose synthase
Glyma.17g167100-Myb70	15312448	Myb domain protein 70
Glyma.17g168900-WRKY15	15629891	WRKY DNA-binding protein 15

Glyma.17g171100-CCoAOMT(TC225339)	16172938	Caffeoyl-CoA O-methyltransferase
Glyma.17g173100-bHLH	17848992	Basic helix-loop-helix (bHLH) DNA-binding superfamily/Myc
Glyma.17g173200-DFR	17882585	Dihydroflavonol 4-reductase
Satt311	***18065453***	***SSR***
Glyma.17g174400-GST	18153382	Glutathione S-transferase
Glyma.17g177800-Pox	19135422	Peroxidase
Glyma.17g178500-HD	19324290	Homeodomain-like superfamily protein
Glyma.17g180800-bHLH	20729812	Basic helix-loop-helix (bHLH) DNA-binding superfamily/Myc
Glyma.17g182400-HK	22439701	Hexokinase
Glyma.17g185000-NAM.NAC83	23531754	NAC domain transcription regulator superfamily protein NAC83
Glyma.17g186600-EF(X56856)	23719358	Elongation factor Tu domain 2
Glyma.17g188200-GlyH	24384072	Glycosyl hydrolase
Glyma.17g188500-bZIP	24483766	Basic-leucine zipper (bZIP) transcription factor
Glyma.17g190100-AcetylGluT	26560251	Core-2/I-branching β-1,6-N-acetylglucosaminyltransferase
Glyma.17g194400-AK	29127921	Arabinose kinase
Glyma.17g196700-Csl.C5	30779213	Cellulose synthase-like C5
Glyma.17g197500-WRKY11	31139783	WRKY DNA-binding protein 11
Glyma.17g198700-Pox	31473297	Peroxidase
Glyma.17g199400-GST	31585092	Glutathione S-transferase
Glyma.17g201400-MT	32021667	Methyltransferase
Glyma.17g202000-AGP	32165828	FASCICLIN-like arabinogalactan protein
Glyma.17g202500-GlyH17	32240216	Glycosyl hydrolase family 17
Glyma.17g203000-OFT	32368590	O-fucosyltransferase family protein
Glyma.17g206400-AcylT	33851280	HXXXD-type acyltransferase family protein
Glyma.17g209100-P450	34364400	Cytochrome P450
Glyma.17g212500-GalT	35167055	Galacturonosyltransferase
Sat_001	***36455269***	***SSR***
Glyma.17g217000-GlyH	36553695	Glycosyl hydrolase
Glyma.17g217200-AcylT	36624152	HXXXD-type acyltransferase family protein
Satt301	***36718722***	***SSR***
Glyma.17G234700-LIM(TC204441)	38994529	LIM domain-containing protein
Glyma.17g252200-DFR2(EF187612)	40652090	Dihydroflavonol 4-reductase 2
Glyma.17g260100-CAD	41442836	Cinnamyl alcohol dehydrogenase

Chromosome Gm18 (1-58018742)

Feature	Position	Annotation
BARC-Satt163	***997970***	***SSR***
Glyma.18g014900-HD6	1050631	Homeodomain-like superfamily protein 6
Glyma.18g016100-UDPGluT.85A3	1152808	UDP-glucosyltransferase 85A3
Satt275	***1239847***	***SSR***
Glyma.18g017600-ABCtr	1248018	ABC transporter
Satt038	***1344090***	***SSR***
Glyma.18g021800-Pox	1605820	Peroxidase

Glyma.18g023600-LAC	1723409	Laccase/Diphenol oxidase family protein
Satt309	***1736692***	***SSR***
Glyma.18g024300-GlyH85	1795058	Glycosyl hydrolase family 85
Glyma.18g028300-P450	2124636	Cytochrome P450
Glyma.18g030700-Cell2	2355461	Cellulase
Glyma.18g038600-CK.pfkB	3109693	pfkB family carbohydrate kinase
Glyma.18g043000-HD	3626320	Homeodomain-like superfamily protein
BARC-Satt356	***3666591***	***SSR***
Glyma.18g043700-GST	3734876	Glutathione S-transferase
Glyma.18g053300-MADS.AGL80	4615120	MADS box protein/AGL80
Glyma.18g055600-Pox	4841840	Peroxidase
Glyma.18g056600-WRKY	4952759	WRKY DNA-binding protein
Glyma.18g057200-LAC(AF527604)	5045036	Laccase/Diphenol oxidase family protein
Glyma.18g059500-HCT(AK286472e)	5271846	Hydroxycinnamoyl-CoA shikimate/quinate hydroxycinnamoyl transferase
Sat_315	***5348175***	***SSR***
Glyma.18g060200-AcylT.NAT	5366551	Acyl-CoA N-acyltransferase (NAT) superfamily protein
Glyma.18g060600-F26BP	5457321	Fructose-2, 6-biphosphatase
Sat_290	***5496681***	***SSR***
BARCSOYSSR_18_0314	***5634544***	***SSR***
Glyma.18g065100-LAC	5925631	Laccase/Diphenol oxidase family protein
Satt324	***5927346***	***SSR***
Glyma.18g065200-HD	5946779	Homeodomain-like superfamily protein
Glyma.18g067200-AGluP(BE806173)	6220528	α-glucan phosphorylase
Glyma.18g071600-Myb103	6727657	Myb domain protein 103
Glyma.18g080100-P450	7673760	Cytochrome P450
Glyma.18g089000-MBOAT	8845472	Membrane bound O-acyl transferase (MBOAT) family protein
Glyma.18g096500-CaM	9889095	Calmodulin binding protein
BARC-Satt394	***10003685***	***SSR***
Glyma.18g097600-MatE	10136404	MATE efflux family protein
Glyma.18g098100-CesA	10187377	Cellulose synthase
Glyma.18g102700-Glu6Pt(AY942816)	10931521	Glucose-6-phosphate/phosphate translocator
Glyma.18g103400-HCT(AK286472b)	11116646	Hydroxycinnamoyl-CoA shikimate/quinate hydroxycinnamoyl transferase
Glyma.18g103500-HCT(AK286472a)	11234541	Hydroxycinnamoyl-CoA shikimate/quinate hydroxycinnamoyl transferase
Glyma.18g103600-HCT(AK286472c)	11264448	Hydroxycinnamoyl-CoA shikimate/quinate hydroxycinnamoyl transferase
Glyma.18g104000-HCT(AK286472g)	11360219	Hydroxycinnamoyl-CoA shikimate/quinate hydroxycinnamoyl transferase
Glyma.18g104100-HCT(AK286472d)	11399570	Hydroxycinnamoyl-CoA shikimate/quinate hydroxycinnamoyl transferase
Glyma.18g106500-XET15	11926798	Xyloglucan endo-transglycosylase 15 (XET) /Glycosyl hydrolase family 16
Glyma.18g110700-NAM.NAC42	13087248	NAC domain transcription regulator superfamily protein NAC42
Glyma.18g113100-HCT(AK286472f)	13453544	Hydroxycinnamoyl-CoA shikimate/quinate hydroxycinnamoyl transferase
Glyma.18g120200-CT	15009600	Chitinase
Glyma.18g123400-STP	15878055	Sugar transport protein
Glyma.18g131600-Pox	18418395	Peroxidase
Glyma.18g135100-PFK	19604222	Phosphofructokinase family protein

Glyma.18g137300-ALDH.2B4	20178242	Aldehyde dehydrogenase
Glyma.18g143800-MADS.AGL29	23164282	MADS box protein/AGL29
Glyma.18g153500-EXP.A4	30102640	Expansin
Glyma.18g175300-PL	41509717	Pectate lyase
Glyma.18g177000-CAD	42208498	Cinnamyl alcohol dehydrogenase
Glyma.18g177400-LAC	42324003	Laccase/Diphenol oxidase family protein
Glyma.18g179800-PAE	43340286	Pectinacetlesterase family protein
Glyma.18g181700-EXP.A4	43752350	Expansin
Glyma.18g183300-GlyH82	44194502	Glycosyl hydrolase family 82
Glyma.18g183700-LAC	44276577	Laccase/Diphenol oxidase family protein
Glyma.18g194200-Myb113	46166132	Myb domain protein 113
Glyma.18g211700-UDPGlu4E	49818868	UDP-D-glucuronate 4-epimerase
Glyma.18g215600-GlyH	50266705	Glycosyl hydrolase
Glyma.18g218400-P450	50535728	Cytochrome P450
Glyma.18g219500-OFT	50634153	O-fucosyltransferase family protein
Glyma.18g223300-WD40	51002335	transducin family protein/WD40 repeat family protein
BARC-Satt288	***51127072***	***SSR***
Glyma.18g224200-GalT1	51178324	Galacturonosyltransferase 1
Glyma.18g224500-MADS.AGL20	51203136	MADS box protein/AGL20
Glyma.18g225000-PO(CA784777b)	51307307	Polyphenol oxidase
Glyma.18g225100-PO(CA784777a)	51316886	Polyphenol oxidase
Glyma.18g235700-CallS5	52422195	Callose synthase 5
Glyma.18g236700-PL	52558809	Pectate lyase
Glyma.18g243300-GF6PTa	53151480	Glutamine-fructose-6-phosphate transaminase (isomerizing)/sugar binding
Glyma.18g247100-UDPGlyT	53443452	UDP-glycosyltransferase
Glyma.18g259100-Myb30	54462068	Myb domain protein 30
Glyma.18g260900-EXP.A1	54674591	Expansin
Glyma.18g263400-WRKY3	54874974	WRKY DNA-binding protein 3
Glyma.18g264700-GalT13	54973306	Galacturonosyltransferase 13
Glyma.18g268600-AcylT	55238799	HXXXD-type acyltransferase family protein
Glyma.18G272100-COBL4(TC210804)	55506268	COBRA-like extracellular glycosyl-phsphatidyl inositol-anchored protein
Glyma.18g281800-HD	56277508	Homeodomain-like superfamily protein
Sat_064	***56333703***	***SSR***
Glyma.18g290800-Act(U60503)	56972637	Actin

Chromosome Gm19 (1-50746916)

Feature	Position	Annotation
Glyma.19g003700-SL(BE806787b)	272545	Sucrase/ferredoxin-like family protein
Glyma.19g006200-PG(AF128266)	590763	Polygalacturonase 4/Glycosyl hydrolase family 28
Satt232	***1636094***	***SSR***
Glyma.19g016600-ABCtr	1691184	ABC transporter
Glyma.19g017200-SIS	1785304	Sugar isomerase (SIS) family protein
Glyma.19g018300-F5H	1910996	Ferulic acid 5-hydroxylase 1/Cytochrome P450

Glyma.19g018800-AcetylGluT	1974856	
Satt182	***2059590***	***SSR***
Glyma.19g020200-PL	2157221	Pectate lyase
Glyma.19g020600-WRKY72	2228698	WRKY DNA-binding protein 72
Glyma.19g021400-bHLH	2315882	Basic helix-loop-helix (bHLH) DNA-binding superfamily/Myc
Glyma.19g021900-NAM.NAC94	2444946	NAC domain transcription regulator superfamily protein NAC94
Glyma.19g024200-EXP.A15	2774481	Expansin
Glyma.19g025000-Myb113	3117635	Myb domain protein 113
Glyma.19g025500-PME31	3196339	Pectin methylesterase 31
Glyma.19g028000-UDPGluT.85A5	3507265	UDP-glucosyltransferase 85A5
Glyma.19g028100-GalT13	3511914	Galacturonosyltransferase 13
Glyma.19g028500-UDPGlu6D	3556053	UDP-glucose 6-dehydrogenase family protein
Glyma.19g029500-UDPGlyT	3669589	UDP-glycosyltransferase
Glyma.19g030800-AcylT	3799941	HXXXD-type acyltransferase family protein
Glyma.19g032200-OFT	4055354	O-fucosyltransferase family protein
Glyma.19g032600-PL	4166595	Pectate lyase
Glyma.19g033500-COBL	4318949	COBRA-like extracellular glycosyl-phsphatidyl inositol-anchored protein
Glyma.19g035800-UDPGluT.85A7	4822240	UDP-glucosyltransferase 85A7
Glyma.19g037900-bZIP42	5316778	Basic-leucine zipper (bZIP) transcription factor 42
Glyma.19g038000-GalT9	5321094	Galacturonosyltransferase 9
Glyma.19g039300-AcylT	5606232	HXXXD-type acyltransferase family protein
Glyma.19g040300-HD	5819045	Homeodomain-like superfamily protein
Glyma.19g043200-GlyH81	6293349	Glycosyl hydrolase family 81
Glyma.19g045900-MADS	6897027	MADS box protein
Glyma.19g049300-Mann6PI	7859637	Mannose-6-phosphate isomerase
Glyma.19g053800-PL	8832961	Pectate lyase
Glyma.19g055800-Myb40	9381699	Myb domain protein 40
Glyma.19g056400-NAM.NAC85	9892895	NAC domain transcription regulator superfamily protein NAC85
Glyma.19g056800-MT	10351816	Methyltransferase
Glyma.19g057300-P450	10854237	Cytochrome P450
Glyma.19g057700-GLP2	11099976	Germin-like protein 2/Cupin
Glyma.19g060200-SL(BE806787a)	11917708	Sucrase/ferredoxin-like family protein
Glyma.19g061600-Myb24	12792394	Myb domain protein 24
Glyma.19g063300-WD40	13702920	transducin family protein/WD40 repeat family protein
Sat_134	***14048834***	***SSR***
Glyma.19g066200-Pox	17963622	Peroxidase
Glyma.19g066300-XET16	17967045	Xyloglucan endo-transglycosylase 16 (XET) /Glycosyl hydrolase family 16
Glyma.19g068700-GST	21391493	Glutathione S-transferase
Glyma.19g070500-P450	23096723	Cytochrome P450
Glyma.19g076200-CT	27493941	Chitinase
Glyma.19g077300-Glu6PD	27787739	Glucose-6-phosphate dehydrogenase
Glyma.19g078400-INV.PMEI	28138911	Invertase/Pectin methylesterase inhibitor superfamily protein
Glyma.19g079000-MT	28410684	Methyltransferase

Glyma.19g082300-Glu6PD	29813147	Glucose-6-phosphate dehydrogenase
Glyma.19g082700-CallS5	29970325	Callose synthase 5
Glyma.19g084800-bZIP	30392696	Basic-leucine zipper (bZIP) transcription factor
Glyma.19g085700-Myb52	30599257	Myb domain protein 52
Glyma.19g086300-WD40	30698458	transducin family protein/WD40 repeat family protein
Glyma.19g087100-EXP.lA1	31082836	Expansin
Glyma.19g090600-GalO	32192563	Galactose oxidase/Kelch repeat superfamily protein
Glyma.19g091800-Pox	32476331	Peroxidase
Glyma.19g094100-WRKY75	33279312	WRKY DNA-binding protein 75
Glyma.19g096100-AcylT	33923607	HXXXD-type acyltransferase family protein
Glyma.19g097700-NAM.NAC83	34293345	NAC domain transcription regulator superfamily protein NAC83
Glyma.19g098900-CaM	34495795	Calmodulin binding protein
Satt313	***34753106***	***SSR***
Glyma.19g126000-C3H(TC205337)	38430097	*p*-coumarate 3-hydroxylase/Cytochrome P450 family 98
Glyma.19g196900-SCD(EV274513a)	45406403	Short chain dehydrogenase
Glyma.19g197000-SCD(EV274513d)	45413716	Short chain dehydrogenase
Glyma.19g197100-SCD(EV274513b)	45427765	Short chain dehydrogenase
Glyma.19g197200-SCD(EV274513c)	45433259	Short chain dehydrogenase
Glyma.19g214100-Csl	46740824	Cellulose synthase-like
Glyma.19g257400-Myb4(TC232662)	50136504	Myb domain protein 4

Chromosome Gm20 (1-47904181)

**Feature**	**Position**	**Annotation**
Satt571	***1287393***	***SSR***
Glyma.20g014400-HD	1322447	Homeodomain-like superfamily protein
Glyma.20g017400-OFT	1699813	O-fucosyltransferase family protein
Glyma.20g017600-HD.WUSCHEL	1723158	Homeodomain-like superfamily protein WUSCHEL
Satt419	***1885268***	***SSR***
Glyma.20g018200-GlyH	1893421	Glycosyl hydrolase
Glyma.20g018900-P450	1960201	Cytochrome P450
Glyma.20g019800-CK.pfkB	2051817	pfkB family carbohydrate kinase
Glyma.20g023200-PL	2459936	Pectate lyase
Satt367	***2615587***	***SSR***
Glyma.20g024500-CaM	2662431	Calmodulin binding protein
Glyma.20g026700-GlyT	2935653	Glycosyltransferase
Glyma.20g028600-CWINV2	3390037	Cell wall invertase
Glyma.20g028700-CWVIF	3399812	Cell wall/vacuolar inhibitor of fructosidase
Glyma.20g030500-WRKY72	3681098	WRKY DNA-binding protein 72
Glyma.20g032700-bZIP	4267152	Basic-leucine zipper (bZIP) transcription factor
Glyma.20g033500-Pox	4574307	Peroxidase
Glyma.20g035900-AcetylGluT	5083169	Core-2/I-branching β-1,6-N-acetylglucosaminyltransferase
Glyma.20g046000-P450	8581267	Cytochrome P450
Glyma.20g050500-Glu6Pt	10672932	Glucose-6-phosphate/phosphate translocator

Glyma.20g052000-UDPGlyT	11802411	UDP-glycosyltransferase
Glyma.20g067800-XET8	24195403	Xyloglucan endo-transglycosylase 8 (XET) /Glycosyl hydrolase family 16
Glyma.20g070500-S6PPH	25093893	Sucrose-6F-phosphate phosphohydrolase
Glyma.20g077300-PL	27993600	Pectate lyase
Glyma.20g082300-Myb70	30973414	Myb domain protein 70
Glyma.20g086900-ALDH.12A1	32388405	Aldehyde dehydrogenase
Glyma.20g089100-EXP.A17	33087828	Expansin
Glyma.20g090200-GLP10	33253475	Germin-like protein 10/Cupin
Glyma.20g090500-PAE	33289799	Pectinacetlesterase family protein
Glyma.20g096700-GalO	33954993	Galactose oxidase/Kelch repeat superfamily protein
Glyma.20g101100-GST	34439997	Glutathione S-transferase
BARC-Satt354	***34568806***	***SSR***
Glyma.20g102900-MYO	34579855	Myosin
Glyma.20g107500-bHLH	35011922	Basic helix-loop-helix (bHLH) DNA-binding superfamily/Myc
Glyma.20g109300-UDPGluT.72B2	35177398	UDP-Glucosyltransferase 72B2
Satt270	***35362576***	***SSR***
Glyma.20g112600-PL	35468911	Pectate lyase
Glyma.20g114200-C4H	35592130	Cinnamate 4-hydroxylase
Glyma.20g116900-MBOAT	35942037	Membrane bound O-acyl transferase (MBOAT) family protein
Glyma.20g124900-AGP20	36724562	FASCICLIN-like arabinogalactan protein 20
Satt049	***36842373***	***SSR***
Glyma.20g127700-GST	36958692	Glutathione S-transferase
Glyma.20g128600-CAD(TC204220-2)	37015598	Cinnamyl alcohol dehydrogenase
Glyma.20g130200-bHLH	37106096	Basic helix-loop-helix (bHLH) DNA-binding superfamily/Myc
Glyma.20g130400-FUC	37120058	α-L-fucosidase
Glyma.20g133900-GlyT	37387102	Glycosyltransferase
Glyma.20g134500-GlyH	37412134	Glycosyl hydrolase
Glyma.20g135200-PRP	37465148	Proline rich protein/Extensin-like protein repeat
Glyma.20g148400-Cupin	38684625	Cupin family protein
Glyma.20g151700-HRGP	39053053	Hydroxyproline-rich glycoprotein family protein
Glyma.20g153900-CK.pfkB	39281184	pfkB family carbohydrate kinase
Glyma.20g154200-MADS.AGL42	39311328	MADS box protein/AGL42
Glyma.20g154800-GMCO	39365070	Glucose-methanol-choline (GMC) oxidoreductase family protein
Glyma.20g157100-SIS	39614892	Sugar isomerase (SIS) family protein
Glyma.20g157900-Myb19	39666784	Myb domain protein 19
Glyma.20g159100-NAC.vr6	39742639	Vascular-related NAC domain transcription regulator
Glyma.20g159700-P450	39814911	Cytochrome P450
Glyma.20g159900-PAE	39833259	Pectinacetlesterase family protein
Glyma.20g162800-HD	40037989	Homeodomain-like superfamily protein
Glyma.20g163500-ABCtr	40103550	ABC transporter
BARC-Satt330	***40155771***	***SSR***
Glyma.20G164700-ECT.GH18(AK244623a)	40220886	Endo-chitinase
Glyma.20G164900-ECT.GH18(AK244623b)	40232577	Endo-chitinase

Glyma.20g167900-UDPGlyT	40568843	UDP-glycosyltransferase
Glyma.20g168700-INV.PMEI	40650318	Invertase/Pectin methylesterase inhibitor superfamily protein
Glyma.20g169200-Pox	40696468	Peroxidase
Glyma.20g172100-NAM.NAC17	40983077	NAC domain transcription regulator superfamily protein NAC17
Glyma.20g174300-FT13	41167903	Fucosyltransferase 13
Glyma.20g176100-OMT	41337426	O-methyltransferase
Satt162	***41415974***	***SSR***
Glyma.20g177200-INV(TC276114)	41446962	Invertase
Glyma.20g180800-PAL2	41891638	Phenylalanine ammonia lyase 2
Glyma.20g184200-Myb5	42232856	Myb domain transcriprion factor 5
Glyma.20g189800-LAC	42834930	Laccase/Diphenol oxidase family protein
Glyma.20g189900-GlyT47	42864412	Glycosyltransferase family 47
Glyma.20g191800-Pox	43061155	Peroxidase
Glyma.20g192500-NAM.NAC50	43138131	NAC domain transcription regulator superfamily protein NAC50
Glyma.20g197600-GST	43506736	Glutathione S-transferase
Glyma.20g198000-BGlu.GBA2	43537844	β-glucosidase
Glyma.20g209000-MT	44526100	Methyltransferase
Glyma.20g209800-ADF6	44596844	Actin depolymerizing factor
BARC-Satt102	***44794667***	***SSR***
Glyma.20g233900-BMann(TC213549)	46665754	Glycoside hydrolase family protein 2
Glyma.20g241600-CHI2(AY595415b)	47264269	Chalcone-flavanone isomerase
Glyma.20g241700-CHI2(AY595415a)	47267106	Chalcone-flavanone isomerase

[a]Chromosome size
[b]Sequence used to design gene-based primers
[c]Simple sequence repeat (microsatellite) markers mapped in the RG10 x OX948 linkage map (bold, italic)
[d]Chromosome Gm05 (A1) does not exist in the RG10 x OX948 linkage map
